# Comparison of Phenotype and Genotype Virulence and Antimicrobial Factors of *Salmonella* Typhimurium Isolated from Human Milk

**DOI:** 10.3390/ijms24065135

**Published:** 2023-03-07

**Authors:** Joanna Pławińska-Czarnak, Karolina Wódz, Magdalena Guzowska, Elżbieta Rosiak, Tomasz Nowak, Zuzanna Strzałkowska, Adam Kwieciński, Piotr Kwieciński, Krzysztof Anusz

**Affiliations:** 1 Department of Food Hygiene and Public Health Protection, Institute of Veterinary Medicine, Warsaw University of Life Sciences, ul. Nowoursynowska 159, 02-776 Warsaw, Poland; 2 Laboratory of Molecular Biology, Vet-Lab Brudzew, ul. Turkowska 58c, 62-720 Brudzew, Poland; 3 Department of Physiological Sciences, Faculty of Veterinary Medicine, Warsaw University of Life Sciences, ul. Nowoursynowska 159, 02-776 Warsaw, Poland; 4 Department of Food Gastronomy and Food Hygiene, Institute of Human Nutrition Sciences, Warsaw University of Life Sciences-SGGW, ul. Nowoursynowska 166, 02-787 Warsaw, Poland

**Keywords:** *Salmonella enterica* subsp. *enterica serovar* Typhimurium, WGS, antimicrobial resistant gene, SPI

## Abstract

*Salmonella* is a common foodborne infection. Many serovars belonging to *Salmonella enterica* subsp. *enterica* are present in the gut of various animal species. They can cause infection in human infants via breast milk or cross-contamination with powdered milk. In the present study, *Salmonella* BO was isolated from human milk in accordance with ISO 6579-1:2017 standards and sequenced using whole-genome sequencing (WGS), followed by serosequencing and genotyping. The results also allowed its pathogenicity to be predicted. The WGS results were compared with the bacterial phenotype. The isolated strain was found to be *Salmonella enterica* subsp. *enterica* serovar Typhimurium 4:i:1,2_69M (*S.* Typhimurium 69M); it showed a very close similarity *to S. enterica* subsp. *enterica* serovar Typhimurium LT2. Bioinformatics sequence analysis detected eleven SPIs (SPI-1, SPI-2, SPI-3, SPI-4, SPI-5, SPI-9, SPI-12, SPI-13, SPI-14, C63PI, CS54_island). Significant changes in gene sequences were noted, causing frameshift mutations in *yeiG*, *rfbP*, *fumA*, *yeaL*, *ybeU* (insertion) and *lpfD*, *avrA*, *ratB*, *yacH* (deletion). The sequences of several proteins were significantly different from those coded in the reference genome; their three-dimensional structure was predicted and compared with reference proteins. Our findings indicate the presence of a number of antimicrobial resistance genes that do not directly imply an antibiotic resistance phenotype.

## 1. Introduction

*Salmonella enterica* subsp. *enterica* serovar Typhimurium is a major cause of gastroenteritis and bacteraemia in humans [1,2] and has been included in the group of invasive non-typhoidal *Salmonella* (iNTS), frequently associated with human and animal diseases [3]. Infection with iNTS can be acquired through contaminated food and water and contact with animals, especially reptiles and amphibians. After infection, the incubation period is short, and gastrointestinal disease often develops within hours or days, during which *S*. Typhimurium develops alongside intestinal inflammation and has been found to survive and multiply in macrophages.

The invasiveness, virulence, and pathogenicity of *Salmonella* spp. have been attributed to genes located on *Salmonella* pathogenicity islands (SPI) [4]. In *Salmonella*, pathogenic islands such as SPI1 and SPI 2 include genes that are responsible for host-cell invasion, phagocytic cell inactivation, apoptosis, and alteration of intracellular transport pathways [5]. Following successful infection, some animals may remain asymptomatic for many months and may shed bacteria in their stools [6,7].

*Salmonella* Typhimurium is frequently connected with outbreaks [8]. One such outbreak associated with chocolate products occurred in 2022, during which 453 people, mainly children, from 14 European countries, Canada, and the United States fell ill [9].

Cases of *Salmonella* spp. infection have been reported in infants following transmission from breastmilk [10,11,12,13,14]. However, a greater risk of *Salmonella* spp. infection, like *Cronobacter sakazakii*, is associated with contact between breast milk and PIF (powdered infant formula) via surfaces, bottles, and utensils [15]. Recent publications highlight the clinical importance of the presence of *Salmonella enterica* subsp. *enterica* in human milk [16,17,18]. This is an area of particular concern considering the growing use of human milk banks: specialized laboratories operating in hospitals that process milk from donors for infants who cannot be fed with their own mothers’ milk [15].

There have been numerous reports related to the emergence of antibiotic resistance in *Salmonella* Typhimurium [19,20], and the presence of genes enabling it to multiply in macrophages and use, for example, iron [7]. Following reports of *Salmonella* occurring in newborns as a zoonosis [21], the aim of the present study was to provide a detailed phenotypic and genotypic analysis of *Salmonella* Typhimurium isolated from human milk and determine its potential for invasiveness, pathogenicity, and antibiotic resistance using whole-genome sequencing.

## 2. Results

### 2.1. Identification of the Isolated Strain

A *Salmonella* strain was isolated from human milk in accordance with PN-EN ISO 6579-1:2017-04 [22]. The strain was grown on differential solid media for *Salmonella* spp. (i.e., on XLD and BGA medium). The tested strain of *Salmonella* indicated characteristic growth on the differential media: convex colonies with a black center and a pink-red halo on a pink-red medium on the XLD agar and pinkish-white colonies on a pinkish-red medium on the BGA agar (Figure S1A,B).

Serological and biochemical analysis indicated that the isolated strain was *Salmonella enterica* subsp. *enterica* serovar O:4 (BO) with no characteristic phenotypical antigenic phase. Positive results were obtained for 14 biochemical reactions: H_2_S production, D-glucose, D-mannitol, D-maltose, D-mannose, gamma-glutamyl-transferase, ornithine decarboxylase, lysine decarboxylase, fermentation/glucose, alpha-galactosidase, phosphatase, coumarate, O/129 resistance, L-malate assimilation, lLATa—L-lactate assimilation. The results of all biochemical reactions are presented in Supplemental Materials, Figure S2.

An automatic analysis based on these findings classified the strains in the following order: *Salmonella* group; *Salmonella* spp.; *S*. Paratyphi B; *S*. Typhimurium; *S. enterica* subsp. *enterica*; *S*. Enteritidis; *S*. Pataryphi C.

Subsequent PCR analysis narrowed this classification to *Salmonella enterica* subsp. *enterica* serovar Typhimurium.

#### 2.1.1. Whole-Genome Sequencing (WGS) and Bioinformatic Analysis

##### The Genome Assembly and Annotation

Reference-based genome assembly (reference genome ASM694v2) was performed with Bowtie 2 v. 2.4.5 [23]. In total, 83 contigs were obtained for *Salmonella enterica* subsp. *enterica* Typhimurium str. 69M (*S*. Typhimurium 69M), with the result demonstrating 99.7% similarity to *S. enterica* subsp. *enterica serovar* Typhimurium strain LT2. The assembled genome was submitted for comprehensive analysis at the on-line PATRIC service [24,25]; based on a comparison with other genomes within this same species, this genome appears to be of good quality (Coarse Consistency 99.7%, Fine Consistency 99.3%, CheckM Completeness 100%).

**Figure 1:**
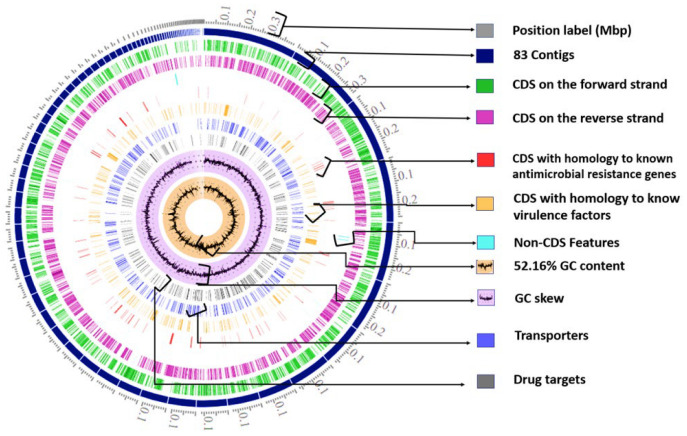
A circular graphical display of the distribution of the genome annotations of *Salmonella enterica* subsp. *enterica* Typhimurium 69M. The colors of the descriptive of the CDS elements of the *Salmonella enterica* subsp. *enterica* Typhimurium 69M were changed for the transparency of the view: grey for position label (MBP), navy for the contigs, green for the CDS on the forward strand, violet for the CDS on the reverse strand, turquoise RNA genes, red for the CDS with homology to known antimicrobial resistance genes, orange for the CDS with homology to know virulence factors, blue for the transporters genes, black for the drugs target genes, black line on orange background GC content, and black on purple GC skew (done with PATRIC-BV-BRC, https://www.bv-brc.org, accessed on 17 November 2022).

The genome encompassed 83 contigs, with a total length of 4,784,350 bp, and one plasmid IncFIB(S). The mean G+C content was 52.16%. The arrangement of the individual elements of the *S*. Typhimurium 69M genome is given in Figure 1; this includes the assembled contigs, coding DNA sequences (CDS), CDS on the forward strand, CDS on the reverse strand, RNA genes, CDS with homology to known antimicrobial resistance genes, CDS with homology to known virulence factors, GC content, and GC skew.

The annotation included 529 hypothetical proteins and 4235 proteins with functional assignments. Those with functional assignments included 1283 with Enzyme Commission (EC) numbers [26], 1048 with Gene Ontology (GO) assignments [27], and 900 proteins that were mapped to KEGG pathways [28]. PATRIC analysis indicated that the genome has 4647 proteins belonging to genus-specific protein families (PLFams) and 4673 belonging to cross-genus protein families (PGFams) [29].

**Figure 2:**
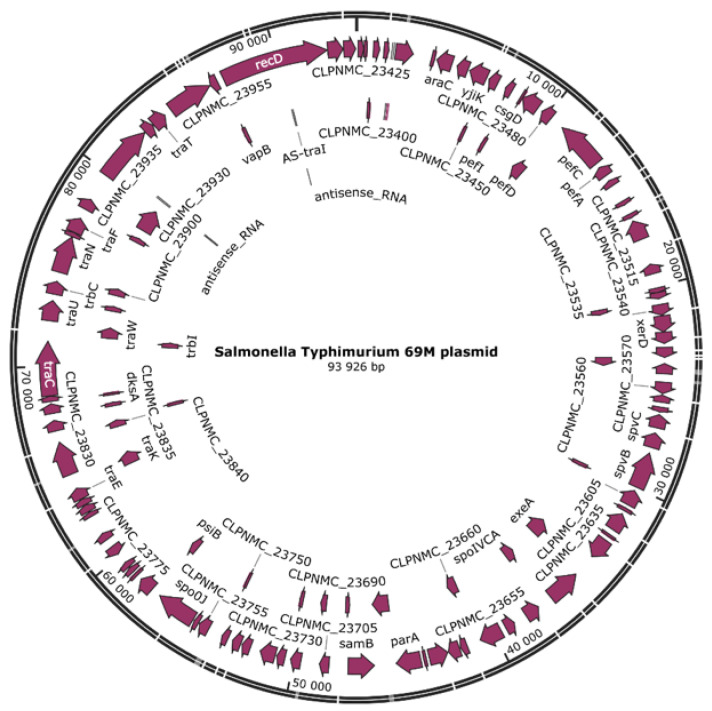
A circular graphic of the IncFIB(S) plasmid found in the *Salmonella* Typhimurium 69M sequence. The length of the plasmid is 93,926 bp, and the virulence genes carried by the IncFIB(S) are marked with maroon arrows (created in SnapGene 6.0.3 (www.snapgene.com, accessed on 2 February 2023)).

Plasmid IncFIB(S) were observed. These carried critical virulence genes (*spvB* and *spvC*) and typical determinants for *Salmonella* pathogenicity island 1 (SPI-1) and SPI-2. They also harbored genes associated with fimbriae (*pefA*, *pefC*, *pefD*, *pefI*) and *vapB* (type II toxin-antitoxin system VapB family antitoxin) (Figure 2).

#### 2.1.2. Serotype Determination Using Whole-Genome Sequencing Data

Genotypic serotyping based on multidirectional WGS analysis showed that O antigen prediction is 4, H1 antigen prediction (*fliC*)—i, H2 antigen prediction (*fljB*)—1,2. The strain was tentatively classified as *Salmonella enterica* subsp. *enterica* (subspecies I) serotype Typhimurium 4:i:1,2 and named as *Salmonella enterica* subsp. *enterica* Typhimurium 4:i:1,2_69M) [30,31].

### 2.2. Analysis for Virulence and Antibiotic Resistance Genes

#### 2.2.1. Salmonella Pathogenic Island (SPI)

The sequence of *S.* Typhimurium 69M was determined using SPIFinder 2.0, the online bioinformatics tool of the Center for Genomic Epidemiology. The results identified eleven SPIs (SPI-1, SPI-2, SPI-3, SPI-4, SPI-5, SPI-9, SPI-12, SPI-13, SPI-14, C63PI, CS54_island) with very high similarity (i.e., 98.58% to 100%) to the reference genome AE006468.2; it also identified other close matches regarding genes and regions of *Salmonella* spp. given in the NCBI Nucleotide Database, including JN673272, Z95891, AJ000509, Y13864, and AJ576316 (Table S1) [32]. All identified parts of the SPIs and their genes, together with their position in the contig and their accession number, are presented in Table 1.

**Figure 3:**
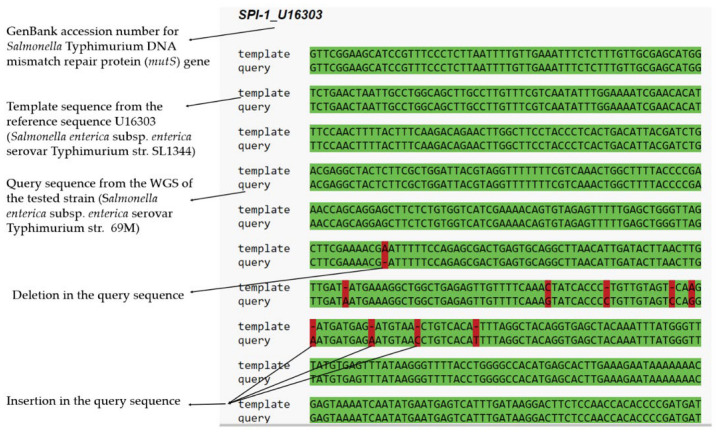
Fragment of SPI 1 with the *mutS* gene. Analysis performed by SPIFinder 2.0. alignment of the *mutS* gene; query (*Salmonella* Typhimurium 69M from human milk) and template (U16303—*Salmonella enterica* subsp. *enterica* serovar Typhimurium str. SL1344). The query sequence was found to demonstrate the greatest similarity (99.43%) with the *Salmonella* Typhimurium DNA mismatch repair protein (*mutS*) gene sequence (U16303).

The *S*. Typhimurium 69M sequences were compared to the reference strain *Salmonella* Typhimurium LT2 genome. The results of fragment of SPI 1 with the *mutS* gene are given in Figure 3, with 100% matches marked in green, and differences such as deletions, insertions, and SNPs in red. Ten single nucleotide insertions and single nucleotide polymorphisms (SNPs) were detected in the *mutS* gene (Figure 3). Despite these differences, the protein sequences generated by the *mutS* gene of the test strain demonstrated 98% and 100% similarity with the reference strains U16303 and *S*. Typhimurium LT2 (ProgramBlast 2 sequences [33]). The entire SPIfinder2.0 analysis is presented in the Supplementary Materials as Table S1.

**Figure 4:**
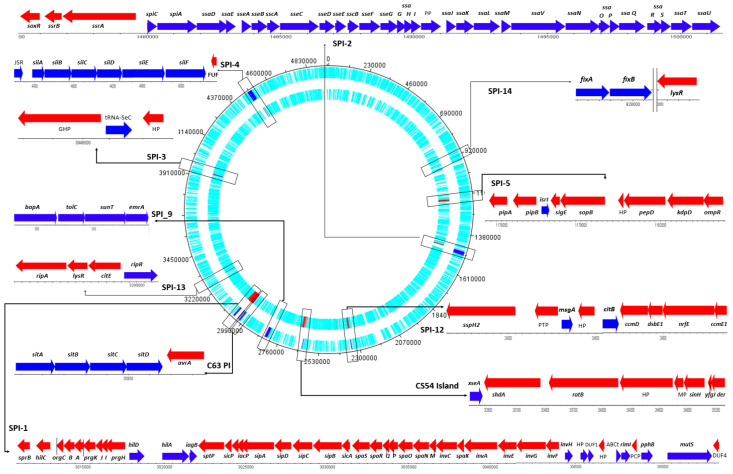
The black line indicates the position on the genome (Kbp); the *Salmonella* Pathogenity Islands are presented in color: the red arrows indicate genes on the reverse strand and the blue arrows indicate those on the forward strand: SPI-1 includes 38 genes and 5 putative protein genes (*sprB, hilC, orgC, orgB, orgA, prgK, prgJ, prgI*, *prgH, hilD, hilA, iagB, sptP, sicP, iacP, sipA, sipD, sipC, sipB, sicA, spaS, spaR, spaQ, spaP, spaO, spaN, spaM, invC, spaK, invA, invE, invG, invF, invH, InvR, rimI, pphB, mutS*, ABCt—ABC transporter, PCP—putative cytoplasmic protein, DUF1—DUF1493 domain-containing protein, DUF4—DUF4440 domain-containing protein); SPI-2 includes 32 genes and 1 putative protein gene (*soxR, ssrB, ssrA, spiC, spiA, ssaD, ssaE, sseA, sseB, sscA, sseC, sseD, sseE, sscB, sseF, sseG, ssaG, ssaH, ssaI, ssaJ*, PIP, *ssaK, ssaL, ssaM, ssaV, ssaN, ssaO, ssaP, ssaQ, ssaR, ssas, ssaT, ssaU)* and (on the position from 2424353 to 2425810) *nuoN;* SPI-3 includes 3 putative protein genes (GHP, tRNA-SeC, HP as Ybl27 hypothetical protein); SPI-4 includes 7 genes and 2 putative protein genes *(ssb1*, JSR, *siiA, siiB, siiC, siiD, siiE, siiF*, FUF), JSR-JUMPstart RNA, and FUF—family of unknown function; SPI-5 includes 8 genes and 2 putative protein genes (tRNA-Ser, *pipA, pipB, isrI, sigE, sopB*, HP, *pepD, kdpD, ompR*); SPI-9 includes 5 genes (*bapA, isrL, tolC, sunT, emrA*); SPI-12 includes 7 genes and 2 putative protein genes (*sspH2*, PTP, *isrG, msgA*, AH, *citB, ccmD, dsbE1, nrfE*); SPI-13 includes 4 genes (*ripA, lysR, citE, ripR*); SPI-14 includes 2 genes, *traX* and *fixA*; the CS54 island includes 5 genes and 2 putative protein genes *(shdA, ratB*, HP, MP, *sinH, yfgJ, der*); C63 PI includes 5 genes (*sitA, sitB, sitC, sitD, avrA*); HP-hypothetical protein, PIP—pathogenicity island protein, AH—acyloxyacyl hydrolase, GPH—glycoside–pentoside–hexuronide family transporter.

The gene sequences obtained from *S.* Typhimurium 69M, particularly those within the identified SPIs, were analyzed using DNAPlotter [34]. It was found that the strain contains 221 genes responsible for virulence factors and antimicrobial resistance. The genes are located within 11 *Salmonella* pathogenicity islands (Figure 4). SPI-1 includes 42 genes; SPI-2, 33 genes; SPI-3, three genes; SPI-4, nine genes; SPI-5, 10 genes; SPI-9, five genes; SPI-12, eight genes; SPI-13, four genes; and SPI-14, two genes (*fixB* and *fixA*). In addition, the CS54 island includes seven genes, and C63-PI includes five genes. The location of individual pathogenicity islands and their genes, along with their arrangement on the forward and the reverse strands, are given in Figure 4.

The WGS analysis showed differences in the arrangement of 14 genes. Most of these, including *pipB* (SPI-2 type III secretion system effector PipB) and *sspH2* (SPI-2 type III secretion system effector E3 ubiquitin transferase SspH2), were transferred from SPI-1 to SPI-5 and SPI-2 to SPI-12 (Figure 4). The relevant SPIs are presented in Table S2, together with their component genes, gene length, strand direction, locus, and a brief description of the product and function. 

#### 2.2.2. Multiple Sequence Alignment (MSA) and Single-Nucleotide Polymorphism (SNP)

**Figure 5:**
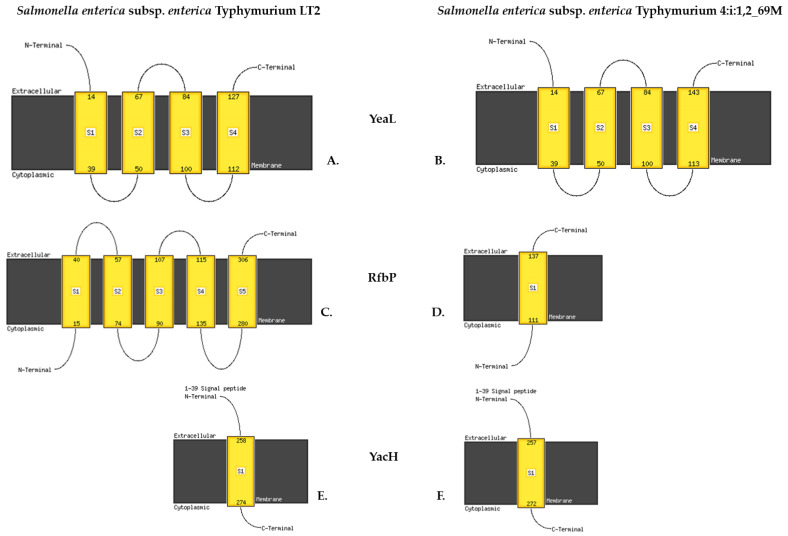
Predicted transmembrane helices. Functional (*Salmonella enterica* subsp. *Enterica* Typhimurium LT2, left panel) and truncated (*Salmonella enterica* subsp. *Enterica* Typhimurium 4:i:1,2_69M, right panel) proteins. YeaL UPF0756 membrane protein (**A**). YeaL LT2 (148 aa), (**B**). YeaL 69M (134 aa); RfbP O-antigen transfer protein (**C**). RfbP LT2 (476 aa), (**D**). RfbP 69M (307 aa); YacH membrane protein (**E**). YacH LT2 (540 aa), (**F**). YacH 69M (285 aa). Created by Phyre2, Protein Homology/analogY Recognition Engine V 2.0, London, UK.

Changes occurring in individual genes were identified using the multiple sequence alignment (MSA) and single-nucleotide polymorphism (SNP) service of the BV-BRC. In total, 700 changes were detected. These included 53 insertions and 24 deletions, of which 36 were classified as having a high impact. These are given in Table 2, with all raw results in Table S3. SNP effects were marked based on the degree of their effect on the encoded protein. Only those genes marked as High SNP Impact Effect, i.e., a destructive effect on the protein, were selected for further analysis (Figure 5). Significant changes were observed in a number of genes due to frameshift variants; these included insertions, such as in *yeiG*, *rfbP*, *fumA*, *yeaL*, and *ybeU*, and deletions, as in *lpfD*, *avrA*, *ratB*, and *yacH*. A stop-gain mutation was observed in *shdA* and *ybfE* and a stop loss-mutation in *yciW.* The most important changes observed in the selected genes are given in Table 2, together with a description of their effect and the possible differences to the *S.* Typhimurium LT2 referential genes. 

Insertions were noted in the *yeaL* and *yeiG* genes of *S.* Typhimurium 69M. These changes cause a frame shift, creating a premature STOP codon and resulting in the loss of a large part of the protein (UPF0756 membrane protein YeaL UniProtKB/Swiss-Prot: Q8ZPW7 [36]). More specifically, the YeaL protein contains 148 aa in the reference strain (NP_460246.2), but only 134 aa in *S*. Typhimurium 69M: gtg cgg Val, Arg (VR), Val130, Arg131 (Val130_Arg131) Val Arg Ser Ala His STOP (TGA) (Figure 5).

The *rfbP* gene of *Salmonella enterica* serovar Typhimurium has two functions: it is involved in the first step of O-antigen synthesis, i.e., the galactosyltransferase [GT] function, and in a later step (the T function as O-antigen transfer protein).

This was first thought to be the flipping of the O-antigen subunit on undecaprenyl-phosphate galactosephosphotransferase (EC 2.7.8.6) from the cytoplasm to the periplasmic space of the cytoplasmic membrane [37].

The detected insertion within the first half of the *rfbP* gene would result in a frameshift mutation leading to the creation of a STOP codon 25 codons below the frameshift. This would shorten the open reading frame to only 307 codons, compared to 476 codons in the reference-type *rfbP* gene.

Insertion 390_391insC, Val130_Arg131fs, creates a new STOP codon in the *yeaL* gene, resulting in the formation of a truncated Yeal multi-pass membrane protein and the disruption of its S4 and C-terminal parts. Similarly, insertion 67_68insT Ile23_Phe24fs causes a premature STOP codon in undecaprenyl-phosphate galactosephosphotransferase/O-antigen transfer protein gene *rfbP*; this results in the formation of a truncated RfbP protein and loss of transmembrane helix structure (Figure 5).

Insertion 720_721insG Arg240_Lys241fs in *yeiG* (S-formylglutathione hydrolase gene) also creates a new STOP codon and the formation of a truncated protein. In addition, insertion 139_140insG Ile47_Asp48fs adds a new STOP codon, resulting in the formation of truncated YbeU protein (Figure S3). In Figure S3 protein model with premature STOP codon in *yeaL*, *rfbP*, *yacH*, *avrA*, and *ratB* were present.

Insertion 1631_1632insC, Gly544_Ala545fs, deletes the STOP codon in the *fumA* gene, leading to the formation of a longer protein than in *Salmonella* Typhimurium LT2 (Figure S4). Table S4. Secondary structure of protein with insertions and deletions in gene of *Salmonella* Typhimurium LT2 and *Salmonella* Typhimurium 69 were described in Table S4.

**Figure 6:**
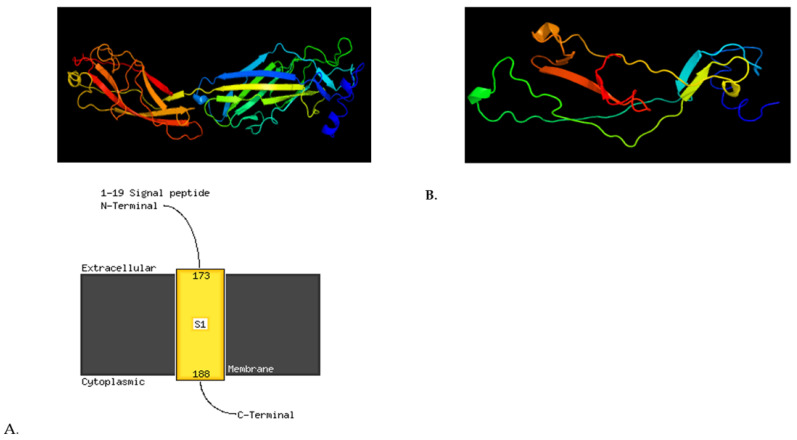
3D viewing of the protein model with premature STOP codon. (**A**). IpfD LT2 (359 aa); (**B**). IpfD 69M (134 aa), the truncated IpfD protein with loss of transmembrane structure. Created by Phyre2, Protein Homology/analogY Recognition Engine V 2.0, London, UK.

A deletion was found in the gene encoding the long polar fimbrial operon protein (*ipfD*), i.e., 400_409delTTTGAGAATG, Phe134fs, which adds a new STOP codon. This results in the production of a truncated IpfD protein and loss of its transmembrane structure. Deletion 5807delT Met1936fs in *ratB*, encoding an outer membrane protein/colonization factor, creates a new STOP codon and potentially leads to the synthesis of a truncated protein (Figure 6). Deletion 850_857delGATCATCA Asp284fs creates a new STOP codon in the outer membrane gene (*yacH*) resulting in a truncated YacH protein sequence (Figure 5).

Deletion 755delA Glu252fs in *avrA* again results in the creation of a premature STOP codon. This leads to the formation of a truncated Type III secretion injected virulence protein (YopP, YopJ), thought to be an inner-membrane protein (Table 2).

#### 2.2.3. Antimicrobal Resistance Genes

The isolated strain turned out to be sensitive to many of the tested antibiotics: ampicillin ≤ 2, amoxicillin with clavulanic acid ≤ 2, cephalexin ≤ 4, cephalotin ≤ 2, cefoperazone ≤ 4, ceftiofur ≤ 1, cefquinone ≤ 0.5 imipenem ≤ 0.25, neomycin ≤ 2, flumequin ≤ 1, enrofloxacin ≤ 0.12, marbofloxacin ≤ 0.5 tetracycline ≤ 1 florfenicol 4, trimethoprim/sulfamethoxazole ≤ 20. *Salmonella enterica* subsp. *enterica* Typhimurium 4:i:1,2_69M showed phenotypic resistance only to gentamicin.

The phenotype of *S*. Typhimurium was determined using VITEK2 software v 8.02, Biomerieux, Marcy-l’Étoile, France: β-lactams, wild; aminoglycosides, resistant to Ami mesh (AAC (6′)) wild; quinolones, wild resistant/partially resistant; tetracyclines, wild; phoenixols, wild–resistant; trimethoprim, wild–resistant. 

In the *Enterobacteriaceae*, resistance to aminoglycoside (gentamycin) is associated with AAC(6′), an aminoglycoside acetyltransferase encoded in the plasmid. The aac(6′)-I-cr variant gene can induce resistance against aminoglycoside and fluoroquinolone simultaneously. Our results also indicate the presence of the AcrAD-TolC efflux pump system, associated with aminoglycoside efflux. Although aminoglycoside resistance can also be caused by mutations within the *gidB* gene, causing changes in the structure of 16s rRNA, no such genetic changes were present in the investigated strain.

Bioinformatic analysis identified genes involved in, broadly understood, antimicrobial resistance involving various mechanisms (Table 3) [24].

*Salmonella* Typhimurium 69M has both *marA*, inducing MDR efflux pump AcrAB, and *marB*, coding the repressor of the mar operon marRAB, which regulates *marA* expression. Although *S*. Typhimurium 69M strain carries the *gyrB* gene, associated with fluoroquinolone resistance, it appears to be sensitive to fluoroquinolone. In addition, the investigated genome contains *inhA* and *kasA*, associated with resistance to isoniazid.

Other AMR mechanisms were also detected. These include AcrAB-TolC, a tripartite efflux system that confers resistance to tetracycline, chloramphenicol, ampicillin, nalidixic acid, and rifampin. This was accompanied by the AcrEF-TolC efflux pump system, involved in resistance to fluoroquinolones, and the MdtABC-TolC multidrug efflux system. The latter includes the *macA* gene, encoding the MacA membrane fusion protein, which forms an antibiotic efflux complex with MacB and TolC. Detailed functions of individual genes are provided in Tables S3 and S4.

## 3. Discussion

Human milk from the Women’s Milk Bank is not available to private individuals: it is illegal to purchase breast milk or give it to a child outside the hospital premises. The milk collected by milk banks is intended primarily for premature babies or for sick infants as part of nutritional therapy. Therefore, it is very important that potential donors are screened for pathogens, including *Salmonella enterica* subsp. *enterica* serotype Typhimurium [15].

The investigated *Salmonella* strain from human milk could not be serotyped with classical methods according to the White–Kauffmann–Le Minor scheme; this approach is time-consuming and complicated, as it requires above one hundred and fifty specific antisera and well-trained personnel to interpret the results.

For the genus *Salmonella*, 47 O serogroups and 114 H antigens have been described according to the Kauffmann–White–Le Minor scheme [38,39]. The O antigen (polysaccharide O) is part of the lipopolysaccharide (LPS) component of the outer membrane. It is necessary for the survival of the bacteria and plays a role in the virulence of *Salmonella* spp. [39].

Often, precise identification is not possible due to the lack of well-expressed flagellar antigens, as was the case in our study. This problem is becoming increasingly common [1,22,40,41], and as such, a number of European countries have included WGS in their screening protocols, such as the UK Standards for Microbiology Investigations [42]. 

In the present study, the biochemical tests and serotyping of isolated pure colonies allowed only the strain to be identified to a subspecies, i.e., *Salmonella enterica* subsp. *enterica*. Subsequent PCR analysis indicated that the strain belonged to the Typhimurium serovar. However, the WGS data confirmed the presence of genes encoding the lipopolysaccharide (O antigen; encoded by *rfb* genes) and flagellar antigens (phases 1 and 2 of H antigen, encoded by *fliC* and *fljB*). The detected antigenic profile was characteristic for the strain of *S.* Typhimurium (4:i:1,2): O antigen 4, H1 antigen I, and H2 antigen 1,2, despite *Salmonella* Typhimurium 69M not revealing any flagellar antigens when tested with H sera.

RfbP belongs to a large family of bacterial membrane proteins required for initiation of O antigen synthesis, and which catalyze the transfer of galactose-1-phosphate to undecaprenyl phosphate (Und-P) [43]. The *rfpB* gene is involved in lipopolysaccharide biosynthesis; it encodes galactosyl transferase, which catalyzes the transfer of galactose to undecaprenol phosphate, which is involved in the initial step in O-polysaccharide synthesis. As noted by Kong et al. (2011), *Salmonella* with mutated *wbaP* (*rfbP*) were significantly attenuated compared to wild-type strains when administered orally to BALB/c mice and were less invasive in host tissues; the mutants also demonstrated substantially reduced bacterial motility [44]. Wand et al. reported the presence of a secondary translation starting within the *rfbP* gene, resulting in the synthesis of a polypeptide with GT activity [37]. These results indicate that the N- and C-terminal parts of RfbP are the T and GT functional domains, respectively.

The genes affected by the changes within the tested *Salmonella* Typhimurium 69M strain are believed to be responsible for the chemotaxis and motility of the bacterial cell, as well as its adhesion and colonization factors. One good example is the *bapA* gene; this encodes the BapA protein, which forms a biofilm together with cellulose, fimbriae, and the *lpfD* colonization factor, fimbrial gene *lpfD*, encoding the adhesin at the tip of the Lpf fimbriae [45].

The *fumA* gene (fumarase A) is responsible for switching flagellar rotation from one direction to another and is hence an essential part of bacterial chemotaxis. In cytoplasm-free bacterial envelopes containing CheY, fumarate has been shown to restore the ability of flagella to switch directions; it also increases the probability of reversal in intact cells. Fumarate acts as a switching factor, presumably by lowering the activation energy of switching; thus, fumarate modulates bacterial flagellar rotation during chemotaxis and plays a role in bacteria metabolism [46]. The encoded protein, FumA (fumarase A), plays an essential role in bacterial chemotaxis through switching the direction of flagellar rotation. Fumarate acts as a switching factor, presumably by lowering the activation energy of switching. Thus fumarate and some of its metabolites may serve as a connection point between the bacterial metabolic state and chemotactic behavior [46].

The outer membrane protein yacH—CpxR is a conserved sensing system, i.e., regulated by genes associated with virulence; they are known to contribute to the resistance of *E. coli* to cationic antimicrobial peptide stress. In CpxR, extracellular protein transcription is reduced upon exposure to a sublethal dose of the cationic antimicrobial insect peptide cecropin A. Single-deletion strains (ΔyacH) demonstrated better survival than wild-type strains after protamine challenge, suggesting that these target genes contribute to resistance to protamine in *E. coli* [47,48]. CpxRA is a two-component system that monitors envelope perturbations and responds by altering the gene expression profile to allow *Salmonella* to survive under harmful conditions. Therefore, CpxRA activation is likely to contribute to *Salmonella* gut infection. However, the role of the CpxRA-mediated envelope stress response in *Salmonella*-induced diarrhea is unclear. In *S. enterica* subsp. *enterica* serovar Typhimurium, it has been found that CpxRA is not needed for the induction of colitis, but is required for gut colonization [49].

In BALB/c mice, a strain of *Salmonella* Typhimurium with a deletion in the *ratB* gene was not able to colonize the cecum, but was still noted in Peyer’s patches, the mesenteric lymph nodes, and spleen. In addition, mutations in *shdA*, *ratB*, and *sivH* resulted in a reduced ability to colonize intestinal tissues. The genes were encoded on the CS54 island and appear to be required for optimal colonization in the mouse cecum [50]. In contrast, despite large deletions and premature codon arrest, *shdA* from *S*. Typhi (*shdA*_STy_) remained fully functional and was found to allow adherence and invasion in a fibronectin-producing epithelial cell line [51].

A significant change was found in *yeiG*, encoding S-formylglutathione hydrolase (esterase), which plays a role in L-glutamine production. The change resulted in alanine replacement in the protein YeiG, demonstrating that Ser145, Asp233, and His256 are essential for protein activity: the residues represent a serine hydrolase catalytic triad in the protein. The enzyme also appears to contribute to the detoxification of formaldehyde, and may be involved in the degradation of methylglyoxal and/or other aldehydes [52,53].

The *yeaL* gene is responsible for the synthesis of the transmembrane protein in *inter alia E. coli*, *Salmonella enterica* subsp. *Enterica*, *Klebsiella pneumoniae*, and *Yersinia enterocolitica* [54]. The gene is also believed to involved in cell-wall biogenesis, which is required by *S*. Typhimurium to survive after desiccation [55].

Our findings indicate that 80.44% of *Salmonella enterica* subsp. *Enterica* showed aminoglycoside resistance facilitated by AAC(6′)-Ib-cr. The gene encodes an aminoglycoside acetyltransferase, which acetylates an amino group at position 6′ in aminoglycoside. Despite the presence of a number of potential antibiotic resistant mechanisms, *S*. Typhimurium 69M was still sensitive to most antibiotics.

Moreover, one plasmid, IncFIB(S), carrying virulence genes *spvB* and *spvC*, associated with fimbriae (*pefA*, *pefC*, *pefD*, *pefI*) and *vapB* (type II toxin-antitoxin system VapB family antitoxin), was observed. The SpvB protein exhibits a cytotoxic effect on host cells and is required for delayed cell death by apoptosis following intracellular infection. The SpvC protein demonstrates phosphothreonine lyase activity and has been shown to inhibit MAP kinase signaling [56]. Interestingly, strains isolated from HIV positive patients, usually carry *spv* genes, strongly suggesting that CD4+ T lymphocytes are required to control disease caused by *spv*-positive *Salmonella*.

The IncFIB(S) plasmid belongs to the IncF family, which is widely distributed throughout the *Enterobacteriaceae*, in particular, *Salmonella enterica* subsp. *Enterica*. These plasmids carry a variety of virulence factors, such as AMR genes and adhesion factors [57]. In *S.* Typhimurium, resistance genes are carried predominantly on IncFII(S)/IncFIB(S)/IncQ1-type plasmids. The detected IncFIB(S) plasmid affects the virulence of this serovar but is not involved in antibiotic resistance because of the absence of the AMR gene (*bla_TEM_*, *tet(A)*, *dfrA15*, *sul1*, *catA1*, *strA/strB*, *addA1*).

In the presence of antibiotic stress, *Salmonella* overexpresses the global activator protein MarA, which induces MDR efflux pump AcrAB, and downregulates the synthesis of the porin OmpF. In addition, *S.* Typhimurium 69M showed the presence of the *marR* gene encoding the MarR protein; this regulates the expression of marA, the activator of multidrug efflux pump AcrAB.

In the stress-response pathways, *Salmonella* Typhimurium alternates sensitivity to triclosan due to point mutations in the *gyrA* gene; similarly, point mutations in *fabI* used in lipid metabolism and fatty acid biosynthesis are disturbed by this biocide.

In addition to direct AMR mechanisms, the *Salmonella* genome expresses AcrAB-TolC, a tripartite efflux system that spans the cell membrane (AcrB) and the outer-membrane (TolC) and is linked together in the periplasm by AcrA. This efflux pump confers resistance to tetracycline, chloramphenicol, ampicillin, nalidixic acid, and rifampin. The cells also expressed AcrAD-TolC, associated with efflux of aminoglycosides, and the AcrEF-TolC efflux pump system, associated with resistance to fluoroquinolones.

The replacement of classical *Salmonella* spp. serotyping methods with molecular biology methods, especially WGS, has been extensively discussed in previous studies [58]. Our present analysis of the *Salmonella enterica* subsp. *enterica* serotype Typhimurium 4:i:1.2_69M sequence revealed the presence of numerous antimicrobial resistance genes. However, the strain turned out to be sensitive to all antibiotics, except for gentamicin, to which the *Enterobacteriacea* demonstrate natural resistance. This probably indicates that the strain was not subject to environmental pressure in terms of the presence of antibiotics. However, there is the genetic potential for activation of these genes. In the environment, such a strain may serve as an effective donor of antibiotic resistance genes for other bacteria.

## 4. Materials and Methods

*Salmonella* spp. was isolated in accordance with PN-EN ISO 6579-1:2017-04 [59]. A human milk sample was preincubated in buffered peptone water (BPW GRASO, Gdansk, Poland) diluted 1:9 and then transferred to the following media: MSRV agar (modified semi-solid Rappaport–Vassiliadis (MSRV) agar, GRASO, Gdansk, Poland) and the Muller–Kauffmann tetrathionate–novobiocin (MKTTn) broth (GRASO, Gdansk, Poland). The potential *Salmonella* colonies were transferred from the selective enrichment media, viz. XLD agar (xylose lysine deoxycholate agar, GRASO, Gdansk, Poland) and BGA agar (Brilliant Green agar, OXOID, Hampshire, United Kingdom), to non-selective nutrient agar (GRASO, Gdansk, Poland). The temperature range and incubation time used for the above-mentioned media was described previously [60].

### 4.1. Serological Testing

Serotyping was performed by slide agglutination according to the White–Kauffmann–Le Minor scheme [61]. Commercial H poly antisera were used to verify the genus of *Salmonella enterica* (IBSS Biomed, Lublin, Poland), O group antisera to determine the O group, (IBSS Biomed, Lublin, Poland), and H phase and H factor antisera to determine the H phase and H factor (IBSS Biomed, Lublin, Poland, Bio-Rad, Chercules, CA, USA) [62].

### 4.2. Biochemical Strain Identification

The colonies demonstrating morphology typical of *Salmonella* spp. on selective agars were subjected to biochemical identification using VITEK2 COMPACT automated system for bacterial identification and VITEK^®^ 2 GN cards (Biomerieux, Marcy-l’Étoile, France). *E. coli* ATCC 25922, *Salmonella* Typhimurium ATCC 14028, *Salmonella* Enteritidis ATCC 13076, and *P. aeruginosa* ATCC 27853 were used as reference strains. Tests were performed according to the manufacturer’s instructions.

### 4.3. Confirmation of Salmonella Identification with Molecular Biology Methods

DNA for Real-Time PCR was extracted from bacterial cells using a Kylt DNA Extraction-Mix II kit (Anicon, Emstek, Germany). A Kylt *Salmonella* spp. kit (Anicon, Emstek, Germany) was used to detect *Salmonella* spp., and a Spp-Se-St PCR kit (BioChek, Reeuwijk, The Netherlands) to detect *Salmonella* Enteritidis and *Salmonella* Typhimurium. Both Real-Time PCR tests were performed according to the manufacturer’s instructions using an Applied Biosystems 7500 Fast Real-Time PCR System (Thermo, Waltham, MA, USA).

### 4.4. Whole-Genome Sequencing

The whole genome and library gene preparation were sequenced using Illumina DNA Prep, (M) Tagmentation (Illumina, San Diego, CA, USA, Nexter DNA CD Indexes (Illumina, San Diego, CA, USA), PhiX Control v3 (Illumina), and MiSeq Reagent Kit v2 (300-cycles, Illumina) on Illumina MiSeq (Illumina, San Diego, CA, USA). All procedures were performed according to the manufacturer’s instructions.

Sequencing data from the Illumina MiSeq platform were extracted. The quality of the raw sequence reads was assessed with FastQC (v0.11.5) [63]. Low-quality sequences and adapters were removed with Trimmomatic (v0.36) with the following parameters: ILLUMINACLIP:adapters.fasta:2:30:10 LEADING:3 TRAILING:3 SLIDINGWINDOW:4:15 MINLEN:36; adapterst.fasta contained a sequence for Nextera_XT adapters (CTGTCTCTTATACACATCT) [64]. The sequence reads were assembled into the reference genome of *Salmonella enterica* subsp. *enterica* serovar Typhimurium str. LT2 (ASM694v2) with Bowtie 2 v. 2.4.5 [23]. SAM files were converted to BAM files with SAMtools [65] and annotated with Bakta v. 1.5.0. [66].

The general information about the assembly quality and gene content of *Salmonella enterica* subsp. *enterica* serovar Typhimurium 69M isolates and genomic components was obtained using the genomics tools of the Bacterial and Viral Bioinformatics Resource Center (BV-BRC, https://www.bv-brc.org accessed on 9 July 2022).

The serotypes of the isolated *S*. Typhimurium 69M strain were identified using a web-based tool: SeqSero 1.2 (https://cge.food.dtu.dk/services/SeqSero/ accessed on 9 July 2022) [30]. Bakta was used for rapid and standardized annotation of the bacterial genome and plasmids; this tool provides dbxref-rich, sORF-including, and taxon-independent annotations in machine-readable JSON and bioinformatics standard file formats for automated analysis of whole-genome sequences (Bakta version 1.4.2 was installed via BioConda with its native database publicly hosted at Zenodo [66]).

The *Salmonella* Pathogenicity Islands were predicted using the SPIFinder online search tool (https://cge.cbs.dtu.dk/services/SPIFinder accessed on 9 July 2022). Known or potential virulence factors were predicted in silico using the Virulence Factor Database (VFDB) (http://www.mgc.ac.cn/cgi-bin/VFs/v5/main.cgi accessed on 19 November 2022). The result was visualized by DNAPlotter [34], BV-BRC, Phyre2 web portal for protein modeling, prediction and analysis (http://www.sbg. bio.ic.ac.uk/phyre2 accessed on 19 November 2022), and plasmid graphics (SnapGene version 6.2) [67].

### 4.5. Antimicrobial Sensitivity Testing: Phenotypic Antibiotic Resistance

The antimicrobial susceptibility studies were performed as described previously [60] using a 96-well MICRONAUT plate in a VITEK2 Compact reader-incubator module, and AST-GN96 cards for gram-negative bacteria (BioMérieux, Marcy-l’Étoile, France). The AST card is a miniaturized and abbreviated version of the doubling dilution technique used to determine MICs by microdilution. The MICs were interpreted according to Clinical and Laboratory Standards Institute (CLSI) and FDA breakpoints (CLSI M100-ED28, 2018).

## 5. Conclusions

*Salmonella* serovars are typically classified based on serotyping according to the Kauffman and White classification scheme. However, some isolates require several passages through semi-solid media to enhance motility and flagellar antigenic expression. Some strains, like the *S*. Typhimurium 69M described herein, do not express serotype H antigens; *S*. Typhimurium 69M possesses a nine-nucleotide deletion (TTTGAGAATG, Phe134fs) in the *ipfD* gene (long polar fimbrial protein gene, part of the fimbrial operon), which creates a premature STOP codon. Pseudogene formation was also evident in a number of host adhesion factors. Two fimbrial genes including *lpfD* (encoding the tip adhesin of the Lpf fimbriae) and *fimH* (encoding the adhesin of the mannose-specific type 1 fimbriae) are inactive in the Sparrow MpSTM strain [45]. It was predicted that the mutated IpfD was truncated, i.e., with only 134 aa; this would result in the loss of its transmembrane structure, and thus potentially limit the value of traditional serotyping. In such cases, WGS *Salmonella* subtyping may ultimately prove to be more reliable and efficient. Although WGS serotyping requires higher technical and informational capacities, and remains expensive, it is clearly a very useful technique for both identifying *Salmonella* spp. strains and predicting the potential development of antibiotic resistance.

## Figures and Tables

**Table 1:** *Salmonella* Pathogenicity Islands (SPIs) detected in the genome of *Salmonella enterica* subsp. *enterica* Typhimurium isolated from human milk (*S*. Typhimurium 69M) and their references in GeneBank.

SPI	Identity %	Query/Template Length	Position in Contig	Genes	Organism	Accession Number
C63PI	100	4000/4000	3,006,066..3,010,065	*sitA*, *sitB*, *sitC*, *sitD*	*Salmonella-enterica*-Typhimurium-SL1344	AF128999
CS54_island	99.92	25255/25252	2,627,012..2,652,263	*xse*, *shdA*, *ratC*, *ratB*, *ratA*, *sinI*, *sinH*, *yfgK*	*Salmonella-enterica*-Typhimurium-ATCC_14028	AF140550
SPI-1	99.96	2705/2705	3,010,641..3,013,345	*sprA*, *sprB*	*Salmonella-enterica*-Typhimurium-SL1344	AF148689
SPI-1	99.77	440/440	3,015,995..3,016,434	*prgH*, *prgI*, *prgJ*	*Salmonella-enterica*-Gallinarum-SGB_1	AY956822
SPI-1	100	430/430	3,018,105..3,018,534	*hilD*	*Salmonella-enterica*-Gallinarum-SGE_2	AY956823
SPI-1	100	470/470	3,026,813..3,027,282	*sipD*	*Salmonella-enterica*-Gallinarum-SGB_4	AY956824
SPI-1	99.28	415/415	3,038,600..3,039,014	*invA*	*Salmonella-enterica*-Gallinarum-SGB_8	AY956825
SPI-1	98.84	259/257	3,039,894..3,040,152	*invA*	*Salmonella-enterica*-Typhimurium-J4STEHO	JN982040
SPI-1	99.43	3155/3141	3,049,734..3,052,886	*mutS*	*Salmonella-enterica*-Typhimurium-SL1344	U16303
SPI-12	97.64	9345/11075	2,342,056..2,351,393	*sspH2*, *isrG*, *citB*, *ccmD*, *dsbE1*, *nrfE*, *ccmE1*	*Salmonella-enterica*-Choleraesuis-SC_B67	NC_006905
SPI-13	99.41	338/338	3,279,563..3,279,900	*gacD*	*Salmonella-enterica*-Gallinarum-SGD_3	AY956832
SPI-13	100	404/404	3,278,852..3,279,255	*gtrA*	*Salmonella-enterica*-Gallinarum-SGG_1	AY956833
SPI-13	100	341/341	3,277,144..3,277,484	*gtrB*	*Salmonella-enterica*-Gallinarum-SGA_10	AY956834
SPI-14	100	501/501	926,728..927,228	*gpiA*	*Salmonella-enterica*-Gallinarum-SGA_8	AY956835
SPI-14	99.55	441/441	32,304..932,744	*gpiB*	*Salmonella-enterica*-Gallinarum-SGC_8	AY956836
SPI-2	98.90	637/637	1,497,019..1,497,655	*ssaO*, *ssaP*	*Salmonella-enterica*-Gallinarum-SGB_10	AY956826
SPI-2	100	642/642	1,489,709…1,490,350	*ssaG*, *ssaH*, *ssaI*, *ssaJ*	*Salmonella-enterica*-Gallinarum-SGC_2	AY956827
SPI-2	100	395/396	1,494342..1,494,736	*ssaV*	*Salmonella-enterica*-Gallinarum-SGC_9	AY956828
SPI-2	99.76	425/425	1,487,684..1,488,108	*sscB*, *sseF*	*Salmonella-enterica*-Gallinarum-SGH_1	AY956829
SPI-2	99.82	547/547	1,477,547..1,478,093	*ssrA*	*Salmonella-enterica*-Gallinarum-SGD_8	AY956830
SPI-2	100	384/384	1,480,008..1,480,391	*spiC*	*Salmonella-enterica*-Typhimurium-St11	JN673272
SPI-2	100	1252/1252	1,475,967..1,477,218	*ssrA*, *ssrB*	*Salmonella-enterica*-Typhimurium	Z95891
SPI-3	100	738/738	3,965,704..3,966,441	*mgtC*	*Salmonella-enterica*-Typhimurium-14028s	AJ000509
SPI-3	100	1514/1514	3,948,169..3,949,682	*selC*	*Salmonella-enterica*-Typhimurium-14028s	Y13864
SPI-4	100	24660/24660	4,476,929..4,501,588	*siiABCDEF*	*Salmonella-enterica*-Typhimurium-ST4/74	AJ576316
SPI-5	100	9069/9069	1,175,309..1,184,377	*Ser_trna*, *pipA*, *pipB*, *isrI*, *sigE*, *sopB*, *pepD*, *kdpD*, *ompR*	*Salmonella*-Typhimurium-LT2	NC_003197
SPI-9	98.58	12647/15696	2,831,233..2,843,879	*bapA*, *isrL*, *tolC*, *sunT*, *emrA*	*Salmonella*-Typhi-CT18	NC_003198

**Table 2:** The selected genes with their mutations and potential effects in the identified *Salmonella* Typhimurium 4:i:1,2_69M sequence; the table also compares the findings with referential genes from *Salmonella enterica* subsp. *enterica* serovar Typhimurium str. LT2.

Pos	Ref	Var	Type	Ref_nt	Var_nt	Ref_nt_pos_change	Ref_aa_pos_change	Locus Tag	Gene Name	Function	snpEff Type	snpEff Impact
1357016	CGC	CGGC	Insertion	gtgcgg	gtGCCGg	390_391insC	Val130_Arg131fs	STM1280	yeaL	UPF0756 membrane protein YeaL	Frameshift variant	HIGH
1543816	GGC	GCGC	Insertion	ggcgct	GCGCgct	1631_1632insC	Gly544_Ala545fs	STM1468	fumA	Fumarate hydratase class I, aerobic (EC 4.2.1.2)	Frameshift variant	HIGH
1794104	A	G	Nonsyn	tga	tgG	981A>G	Ter327Trpext *?	STM1701	yciW	Uncharacterized protein YciW	stop_lost	HIGH
184611	GTGATGATCG	GG	Deletion	gacgatcatcac	gaCC	850_857delGATCATCA	Asp284fs	STM0157	yacH	Uncharacterized protein YacH	Frameshift variant	HIGH
2162286	TAAAAAAAATCAA	TAAAAAAAAATCAA	Insertion	ttgattttttttaat	TTGATTTTTTTTTAat	67_68insT	Ile23_Phe24fs	STM2082	rfbP	Undecaprenyl-phosphate galactosephosphotransferase (EC 2.7.8.6)	Frameshift variant	HIGH
2292159	CGC	CGGC	Insertion	cgcaaa	CGGCaaa	720_721insG	Arg240_Lys241fs	STM2194	yeiG	S-formylglutathione hydrolase (EC 3.1.2.12)	Frameshift variant	HIGH
2633672	G	A	Nonsyn	cga	Tga	28C>T	Arg10 *	STM2513	shdA	AIDA autotransporter-like protein	stop_gained	HIGH
2635902	CAT	CT	Deletion	atg	AG	5807delT	Met1936fs	STM2514	ratB	Putative outer membrane protein	Frameshift variant	HIGH
3010007	GTTTCA	GTTCA	Deletion	aatgaaacg	aaTGAACg	755delA	Glu252fs	STM2865	avrA	Type III secretion injected virulence protein (YopP, YopJ, induces apoptosis, prevents cytokine induction, inhibits NFkb activation)	Frameshift variant	HIGH
3823687	TACATTCTCAAA	TA	Deletion	tttgagaatgta	TA	400_409delTTTGAGAATG	Phe134fs	STM3637	lpfD	Protein LpfD	Frameshift variant	HIGH
720479	TGGGGGAT	TGGGGGGAT	Insertion	tgggggatt	TGGGGGGATt	139_140insG	Ile47_Asp48fs	STM0657	ybeU	hypothetical protein	Frameshift variant	HIGH
757823	G	A	Nonsyn	cag	Tag	280C>T	Gln94 *	STM0695	ybfE	Uncharacterized protein YbfE	stop_gained	HIGH

Ref: *Salmonella enterica* subsp. *enterica* serovar Typhimurium str. LT2, Var: *Salmonella* Typhimurium 4:i:1,2_69M, Ref_nt: reference nucleotide, Var_nt: variant nucleotide, Ref_nt_pos_change: reference nucleotide position change, Ref_aa_pos_change: reference amino acid position change, * Nonsyn: nonsynonymous substitution is a nucleotide mutation that alters the aminoacide sequence of a protein. This variant is defined as an alter in the DNA coding nucleotide that affects gene expression and protein production in different form [35].

**Table 3:** The multi-drug resistance virulence profile of *Salmonella enterica* subsp. *enterica Typhimurium* 4:i:1,2_69M isolated from human milk.

AMR Mechanism	Genes
Antibiotic activation enzyme	*KatG*
Antibiotic inactivation enzyme	*AAC(6’)-Ic,f,g,h,j,k,l,r-z*
Antibiotic resistance gene cluster, cassette, or operon	*MarA, MarB, MarR*
Antibiotic target in susceptible species	*Alr, Ddl, dxr, EF-G, EF-Tu, folA, Dfr, folP, gyrA, gyrB, inhA, fabI, Iso-tRNA, kasA, MurA, rho, rpoB, rpoC, S10p, S12p*
Antibiotic target protection protein	*BcrC*
Efflux pump conferring antibiotic resistance	*AcrAB-TolC, AcrAD-TolC, AcrEF-TolC, AcrZ, EmrAB-TolC, MacA, MacB, MdfA/Cmr, MdtABC-TolC, MdtL, MdtM, MexPQ-OpmE, OprM/OprM family, SugE, TolC/OpmH*
Gene conferring resistance via absence	*gidB*
Protein altering cell wall charge conferring antibiotic resistance	*GdpD, PgsA*
Regulator modulating expression of antibiotic resistance genes	*AcrAB-TolC, EmrAB-TolC, H-NS, OxyR*

## Data Availability

The data presented in this study are available in Supplementary Materials.

## Supplementary Materials

The following supporting information can be downloaded at: https://www.mdpi.com/article/10.3390/ijms24065135/s1. References [68,69,70,71,72,73,74,75,76,77,78,79,80,81,82,83,84,85,86,87,88,89,90,91,92,93,94,95,96,97,98,99,100,101,102,103,104,105,106,107,108,109,110,111,112] are cited in the supplementary materials.
