# Supplementary material for: Comparison of Phenotype and Genotype Virulence and Antimicrobial Factors of Salmonella Typhimurium Isolated from Human Milk
Source: Int J Mol Sci. 2023 Mar 7;24(6):5135. doi: 10.3390/ijms24065135 (PMC10048834; doi:10.3390/ijms24065135)
Supplement: Supplementary file 1 [file ijms-24-05135-s001.zip › Suplement_tab_S4_fig_S1_S2_S3_S4.pdf]

Supplement

Table S4. Secondary structure of protein with insertions and deletions in *Salmonella* Typhimurium LT2 and *Salmonella* Typhimurium 69 based on reports from Phyre2, Protein Homology/analogY Recognition Engine V 2.0, UK.

Protein (LT2/69M aa)		LT2			69M	
	α- helix %	β-strand %	TM-helix %	α- helix %	β-strand %	TM-helix %
YeaL (148/134)	84	0	62	85	0	57
FumA (548/>548)	36	24	-	37	24	-
RfbP (307/245)	56	10	23	47	17	9
YeiG (285/245)	35	18	-	31	18	-
YbeU (231/209)	70	0	-	72	0	-
lpfD (359/134)	6	52	4	13	40	0
AvrA (302/278)	51	13	-	48	13	-
RatB (2435/1974)	2	54	-	2	50	-
YacH (540/285)	23	5	3	46	6	6

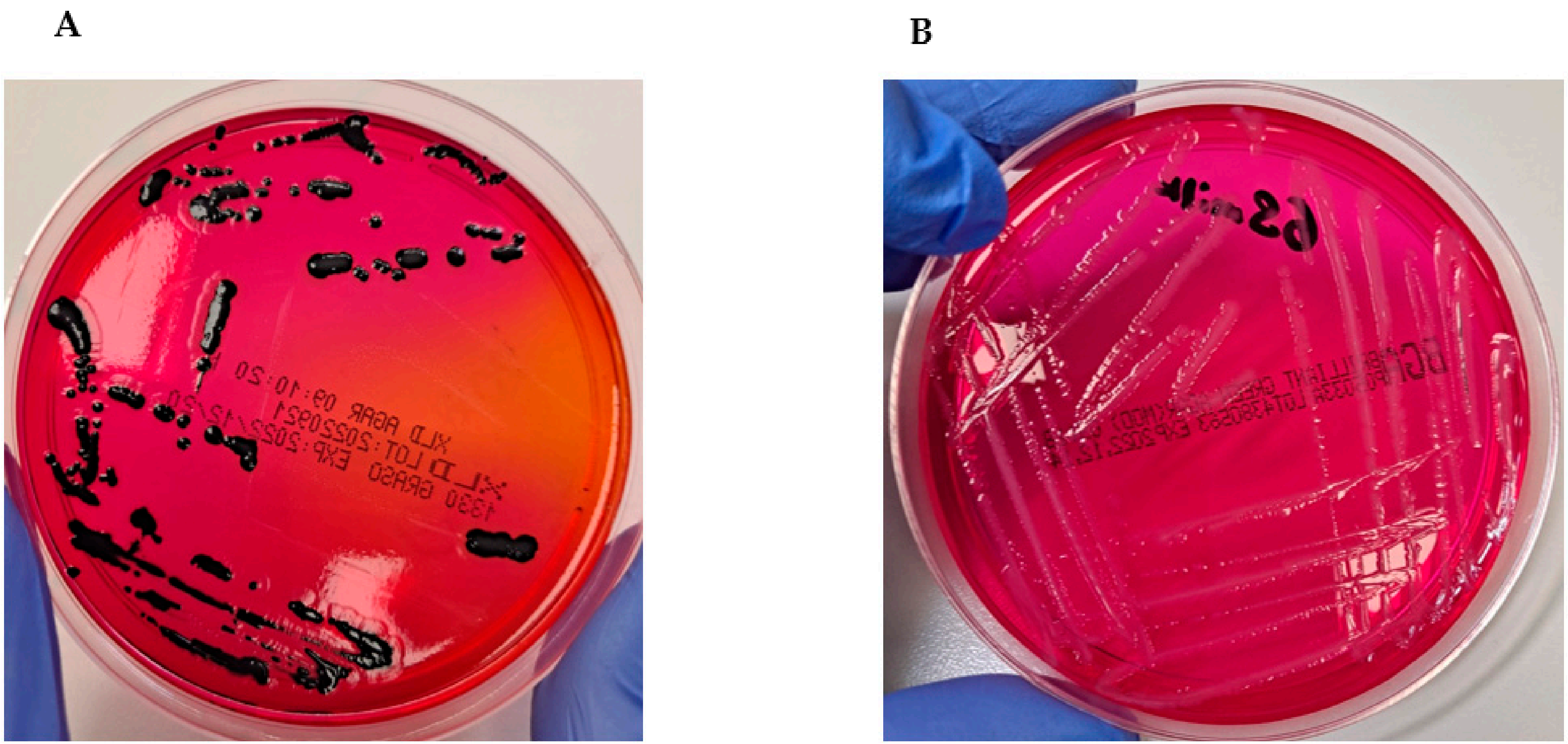

Figure S1. **A** . Characteristic growth of the tested Salmonella spp. strain (isolated from human milk) on XLD agar as convex colonies with a black center and a pink-red surrounded halo on a pink-red medium**.** B . Brilliant Green Agar (BGA) a selective and differential medium for the isolation of Salmonella. Characteristic growth of tested strain Salmonella spp. (isolated from human milk) colonies were as pinkish-white colonies on the pinkish-red medium

XLD Agar (Xylose Lysine Deoxycholate Agar) is one of the mandatory media in accordance with PN-EN ISO 6579-1:2017-04 Food chain microbiology - Horizontal method for detection, enumeration and serotyping of Salmonella - Part 1: Detection of Salmonella spp.

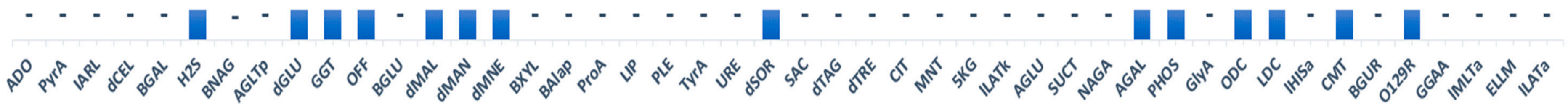

Figure S2. A summary of the results of biochemical reactions for the isolated strain of *Salmonella* spp. in the VITEK test. A positive result is marked in blue and a negative result is marked as a minus. BGAL/ONPG - beta-galactosidase, H2S - H2S production, dGLU/GLU - D-glucose, dMAN/MAN - D-mannitol, URE – urease, dSOR/SOR - D-sorbitol, CIT - citrate (sodium), ODC - ornithine decarboxylase, LDC - lysine decarboxylase, APPA - Ala-Phe-Pro-arylamidase, ADO – adonitol, PyrA - L-pyrrolydonyl-arylamidase, IARL - L-arabitol, dCEL - D-cellobiose, BNAG - beta-n-acetyl-glucosaminidase, AGLTp - Glutamyl Arylamidase pNA, GGT - gamma-glutamyl-transferase, OFF - fermentation/ glucose, BGLU - beta-glucosidase, dMAL - D-maltose, dMNE - D-mannose, BXYL - beta-xylosidase, BAlap - beta-alanine arylamidase pNA, PrpA - L-Proline arylamidase, LIP – lipase, PLE – palatinose, TyrA - Tyrosine arylamidase, dTAG - D-tagatose, dTRE - D-trehalose, MNT – malonate, 5KG - 5-keto-d-gluconate, lLATk - L-lactate alkalinisation, AGLU - alpha-glucosidase, SUCT - succinate alkalinisation, NAGA - beta-N-acetyl-galactosaminidase, AGAL - alpha-galactosidase, PHOS – phosphatase, GlyA - glycine arylamidase, lHISa - L-histidine assimilation, CMT – coumarate, BGUR - beta-glucuronidase, O129R - O/129 resistance (comp.vibrio.), GGAA - Glu-Gly-Arg-arylamidase, lMLTa - L-malate assimilation, ELLM – ellman, lLATa - L-lactate assimilation

Salmonella Typhimurium LT2

Salmonella Typhimurium 69M

A. 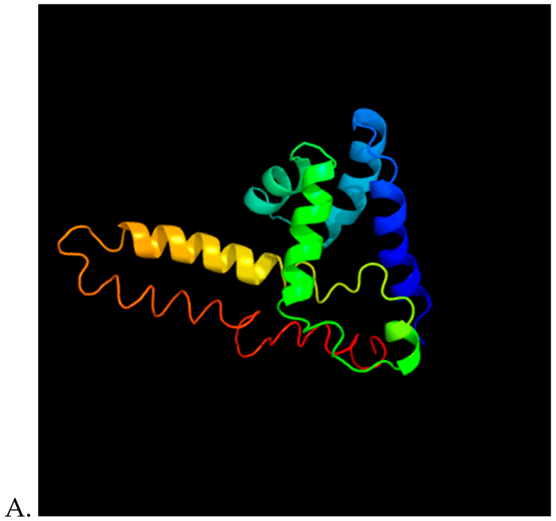

B. 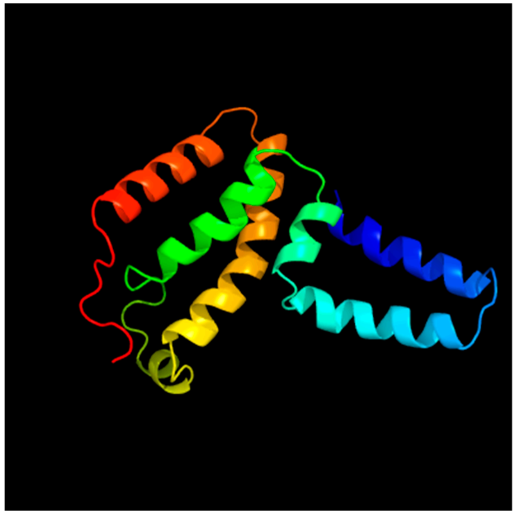

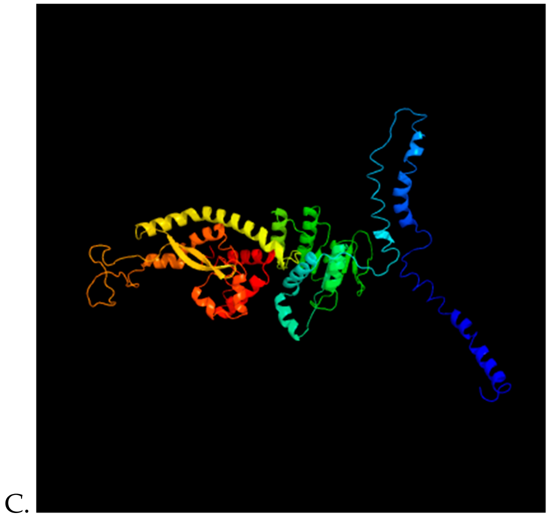
C.
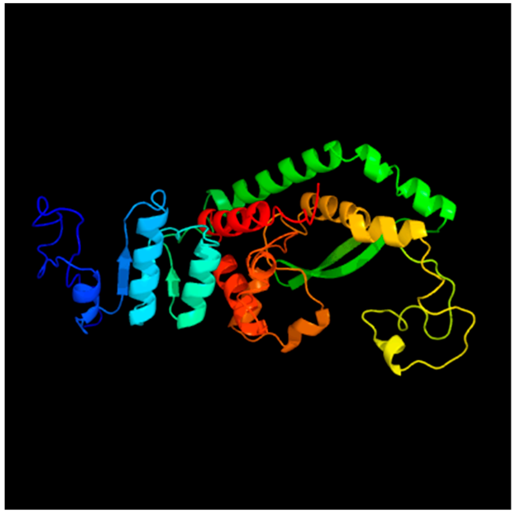
D.
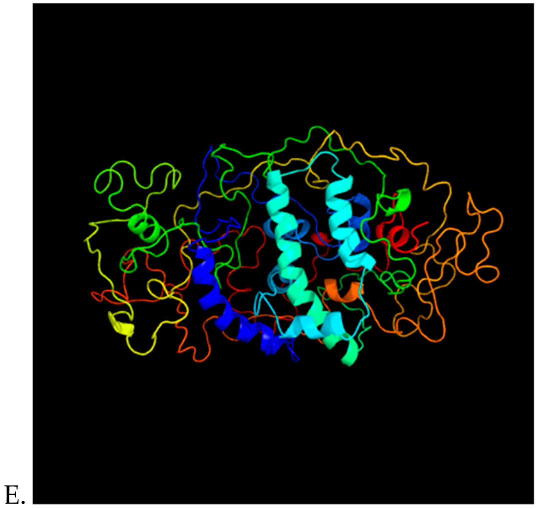
E.
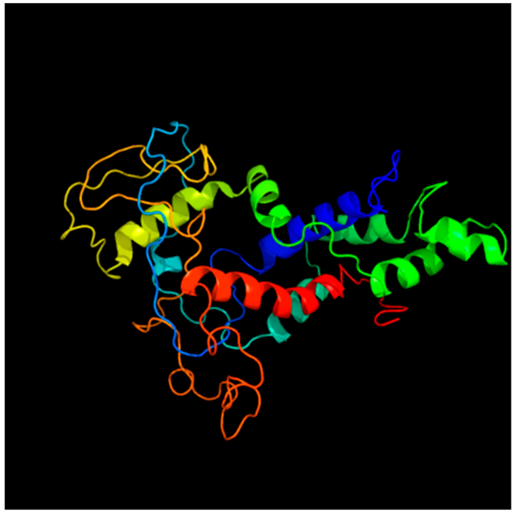
F.

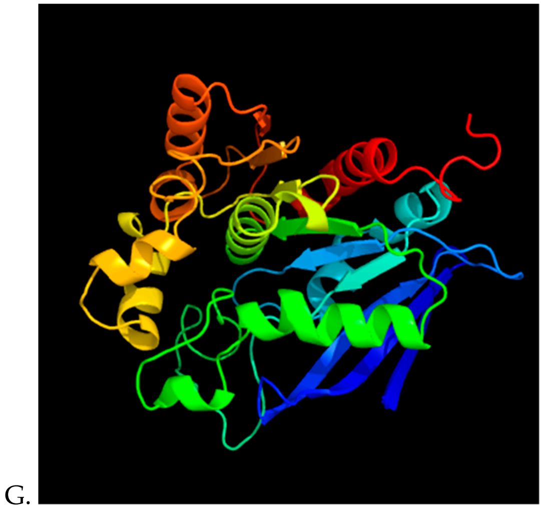
G.

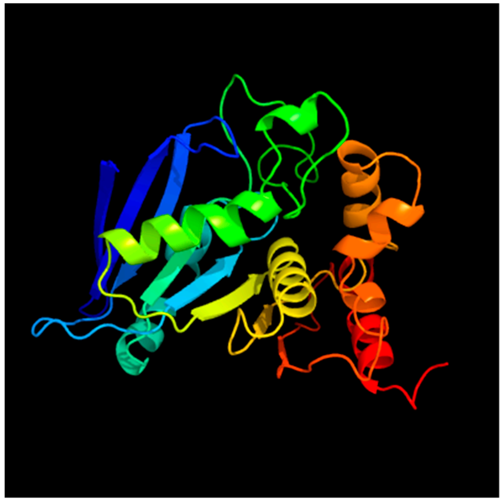
H.

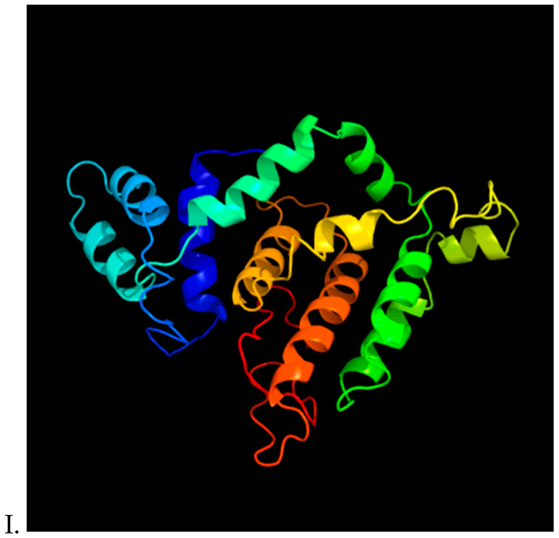
I.

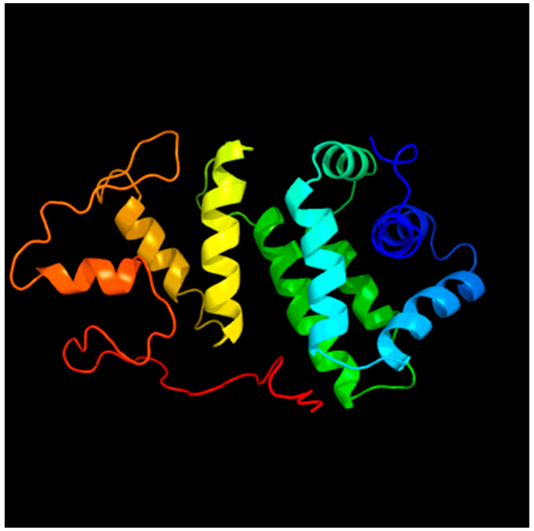
J.

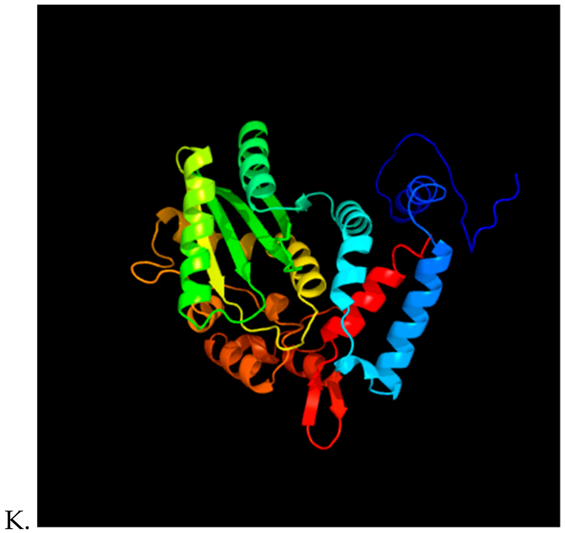
K.
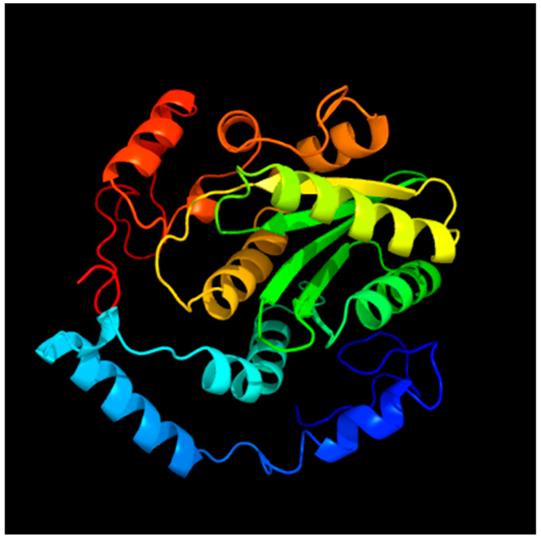
L.
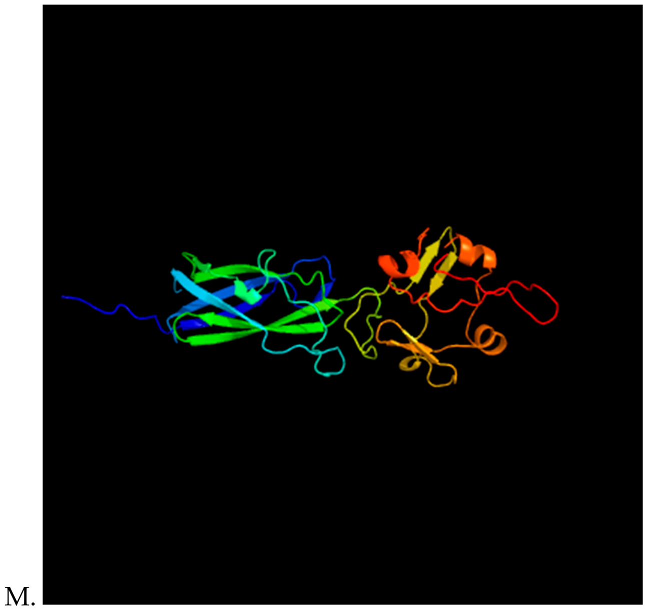
M.
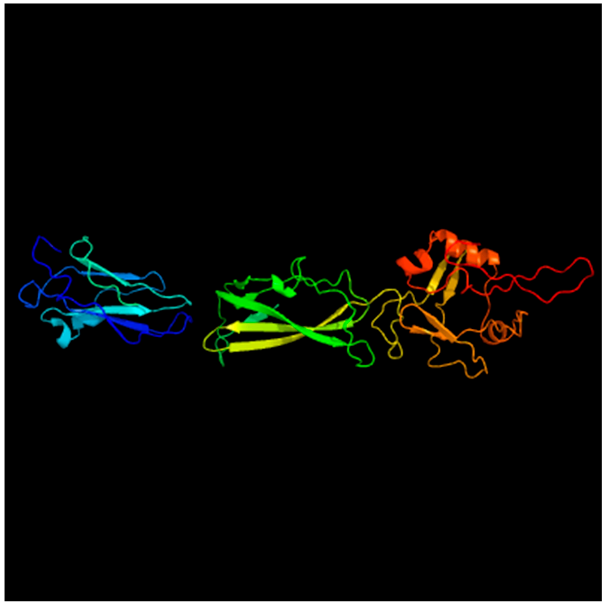
N.

Figure S3. 3D viewing of the protein model with premature STOP codon. Normal (LT2, left panel) and truncated (69M, right panel) protein. A. YeaL LT2 (148 aa), B. YeaL 69M (134 aa), C. RfbP LT2 (476 aa), D. RfbP 69M (307 aa), E. YacH LT2 (540 aa), F. YacH 69M (285 aa), G. YeiG LT2 (285 aa), H. YeiG 69M (245 aa), I. ybeU LT2 (231 aa), J. ybeU 69M (209 aa), K. AvrA LT2 (302 aa), L. AvrA 69M (278 aa). M. RatB LT2 (2435 aa), N. RatB 69M (1974 aa). Created by Phyre2, Protein Homology/analogY Recognition Engine V 2.0, UK.

A. 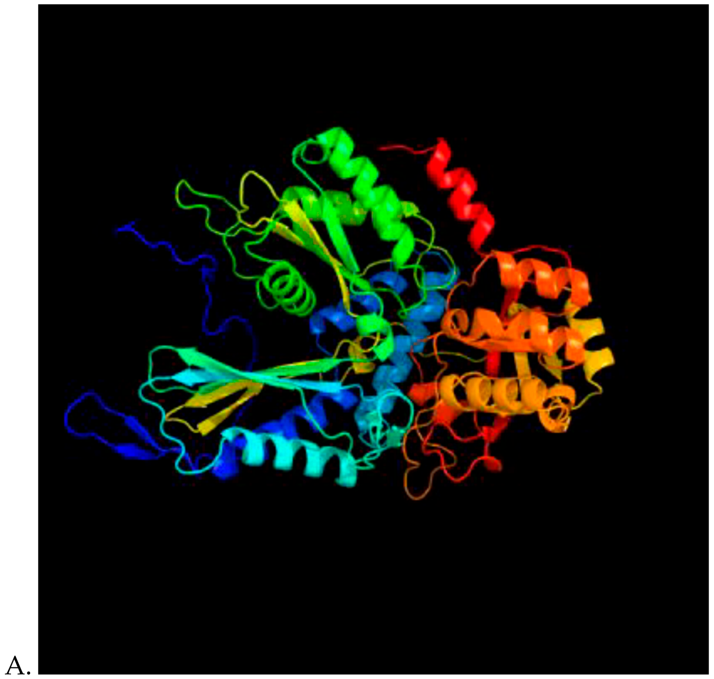

B. 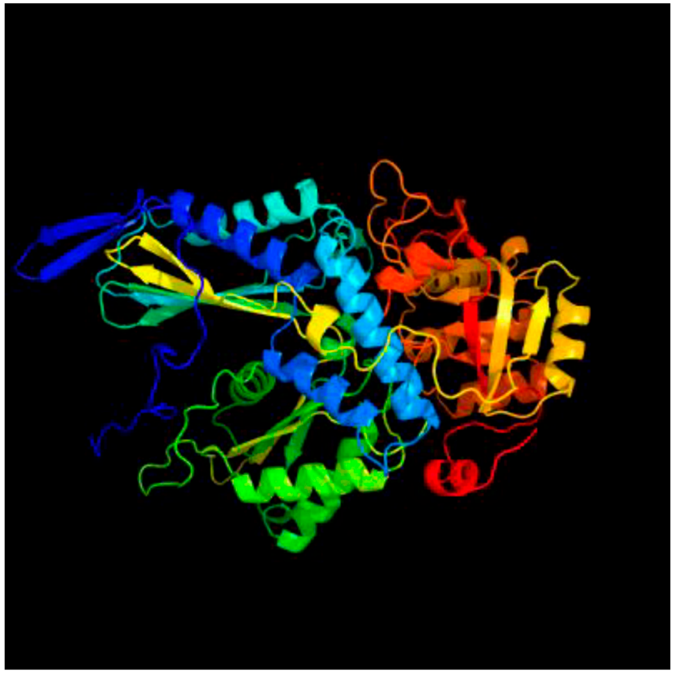

Figure S4. 3D viewing of the protein model with deleted STOP codon. A. FumA LT2 (548 aa), B. FumA 69M (> 548 aa). Created by Phyre2 software.
