# Supplementary material for: Comparison of Phenotype and Genotype Virulence and Antimicrobial Factors of Salmonella Typhimurium Isolated from Human Milk
Source: Int J Mol Sci. 2023 Mar 7;24(6):5135. doi: 10.3390/ijms24065135 (PMC10048834; doi:10.3390/ijms24065135)
Supplement: Supplementary file 1 [file ijms-24-05135-s001.zip › Tabel S3.pdf]

Table S3. Identified antimicrobial resistances genes and their functions in WGS *Salmonella enterica* subsp. *enterica* Typhimurium 4:i:1,2_69M.

Operon/Category	Gene	Function	References
Antibiotic activation enzyme	*katG*	Encodes the bifunctional catalase-peroxidase enzyme that converts isoniazid to an active form. In *katG* mutations are associated with high-level isoniazid resistance, aminoglycoside antibiotic resistance.	[68,69]
marRAB operon	*marA*	Activates expression of genes required for DNA repair and lipid trafficking. Consequently, the *mar* locus reduces quinolone-induced DNA damage and the ability of tetracyclines to traverse the outer membrane. May be a transcriptional activator of genes involved in the multiple antibiotic resistance (Mar) phenotype. It can also activate genes such as sodA, zwf and micF.	[70-72]
marRAB operon	*marB*	*marA, marB., marR* - tetracycline, chloramphenicol, salicylate, resistance to disinfectants such as pine oil, and resistance to weak acids.	[71]
marRAB operon	*marR*	Repressor of the marRAB operon which is involved in the activation of both antibiotic resistance and oxidative stress genes. Binds to the marO operator/promoter site.	[71]
	alr	Isomerizes L-alanine to D-alanine which is then oxidized to pyruvate by DadA.	[73,74]
	ddlA	Cell wall formation.	[75]
	dxr	Catalyzes the NADPH-dependent rearrangement and reduction of 1-deoxy-D-xylulose-5-phosphate (DXP) to 2-C-methyl-D-erythritol 4-phosphate (MEP).	[76]
Antibiotic target in susceptible species	*EF-G (fusA)*	Catalyzes the GTP-dependent ribosomal translocation step during translation elongation. During this step, the ribosome changes from the pre-translocational (PRE) to the post-translocational (POST) state as the newly formed A-site-bound peptidyl-tRNA and P-site-bound deacylated tRNA move to the P and E sites, respectively. Catalyzes the coordinated movement of the two tRNA molecules, the mRNA and conformational changes in the ribosome. Resistance to fusidic acid.	[77]
Antibiotic target in susceptible species	*EF-tu (tufA)*	This protein promotes the GTP-dependent binding of aminoacyl-tRNA to the A-site of ribosomes during protein biosynthesis. Resistance to the antibiotic mocimycin (kirromycin).	[78]
Antibiotic target in susceptible species	*folA*	Key enzyme in folate metabolism. Catalyzes an essential reaction for de novo glycine and purine synthesis, and for DNA precursor synthesis, resistance to trimethoprim.	[79]
Antibiotic target in susceptible species	*folP*	Catalyzes the condensation of para-aminobenzoate (pABA) with 6-hydroxymethyl-7,8-dihydropterin diphosphate (DHPt-PP) to form 7,8-dihydropteroate (H2Pte), the immediate precursor of folate derivatives. resistance to cotrimoxazole, sulfonamide.	[80]
Antibiotic target in susceptible species	*gyrA*	A type II topoisomerase that negatively supercoils closed circular double-stranded (ds) DNA in an ATP-dependent manner to modulate DNA topology and maintain chromosomes in an underwound state, and also catalyzes the interconversion of other topological isomers of double-stranded DNA rings, including catenanes and knotted rings. Replenishes negative supercoiling downstream of highly transcribed genes to help control overall chromosomal supercoiling density. Negative supercoiling favors strand separation, and DNA replication, transcription, recombination and repair, all of which involve strand separation. Type II topoisomerases break and join 2 DNA strands simultaneously in an ATP-dependent manner. Quinolone resistance-determining region (QRDR), resistance to second and third generation fluoroquinolones, nalidixic acid and triclosan, resistance to ciprofloxacin.	[81]
Antibiotic target in susceptible species	*gyrB*	A type II topoisomerase that negatively supercoils closed circular double-stranded (ds) DNA in an ATP-dependent manner to modulate DNA topology and maintain chromosomes in an underwound state, and also catalyzes the interconversion of other topological isomers of double-stranded DNA rings, including catenanes and knotted rings. Replenishes negative supercoiling downstream of highly transcribed genes to help control overall chromosomal supercoiling density. Negative supercoiling favors strand separation, and DNA replication, transcription, recombination and repair, all of which involve strand separation. Type II topoisomerases break and join 2 DNA strands simultaneously in an ATP-dependent manner. Fluoroquinolone resistance, resistance to nalidixic	[82]

		acid and various fluoroquinolones, resistance to aminocoumarins, a group of gyrase inhibitors which include novobiocin and coumermycin.	
Antibiotic target in susceptible species	*fabI*	The antibiotic diazaborine interferes with the activity by binding to the protein. Catalyzes the reduction of a carbon-carbon double bond in an enoyl moiety that is covalently linked to an acyl carrier protein (ACP). Involved in the elongation cycle of fatty acid which are used in the lipid metabolism and in the biotin biosynthesis. type II fatty acid synthase system as the specific cellular target for these antibacterials. (a new antibiotic Fabimycin should affect fabI). fabI alone does not mediate high-level triclosan resistance in *Salmonella* Typhimurium - active efflux of triclosan via AcrAB-TolC confers intrinsic resistance.	[83]
Antibiotic target in susceptible species	*murA*	Cell wall formation. Adds enolpyruvyl to UDP-N-acetylglucosamine. Resistance to Fosfomycin.	[84]
Antibiotic target in susceptible species	*rho*	Facilitates transcription termination by a mechanism that involves Rho binding to the nascent RNA, activation of Rho's RNA-dependent ATPase activity, and release of the mRNA from the DNA template. Mutant background showed an unusual susceptibility to kanamycin. The Rho mutants have altered morphology, distinct cell surface, and increased levels of lipopolysaccharide in their outer membrane, which might have rendered the TolC efflux pumps inefficient. Regulates the broad-spectrum antibiotic susceptibility of E. coli through multipartite pathways in a TolC-dependent manner.	[85]
Antibiotic target in susceptible species	*rpoB*	Rifampin resistance-determining region (RRDR), rifampicin, rifabutin, rifalazil, rifapentine and rifaximin, but also resistance *inhA, mabA-inhA* operon, encoding a target for isoniazid and ethionamide.	[86]
	rpoC	DNA-dependent RNA polymerase catalyzes the transcription of DNA into RNA using the four ribonucleoside triphosphates as substrates. Chromosomal supercoiling density is controlled by a combination of RNA polymerase and DNA gyrase activity; negative supercoiling increases upstream of transcribed regions while it decreases downstream, DNA gyrase introduces negative supercoils into underwound DNA. Quaternary ammonium compound efflux SMR transporter BcrC.	[87]
	bcrC	Quaternary ammonium compound efflux SMR transporter BcrC.	[88]
AcrA-AcrB-AcrZ-TolC is drug efflux protein complex	*acrA*	MDR efflux pump AcrAB transcriptional activator RobA, Putative AcrA protein.	[89-91]
AcrA-AcrB-AcrZ-TolC is drug efflux protein complex	*acrB*	Efflux pump membrane transporter, AcrA-AcrB-AcrZ-TolC is a drug efflux protein complex with a broad substrate specificity. This protein binds to AcrB and is required for efflux of some but not all substrates, suggesting it may influence the specificity of drug export.	[72,89,90]
AcrA-AcrB-AcrZ-TolC is drug efflux protein complex	*acrZ*	Multidrug efflux pump accessory protein AcrZ.	[89,90]
AcrA-AcrB-AcrZ-TolC is drug efflux protein complex	*tolC*	Outer membrane channel.	[89,90]
	acrE	Transmembrane protein affecting septum formation and cell membrane.	[72,92]
	acrF	Efflux pump membrane transporter.	[72]
	emrA	Multidrug resistance secretion protein, membrane fusion protein that transports the substrates across the inner and outer membrane.	[93]
	emrB	Putative MFS superfamily multidrug transport protein.	[93]
MacAB-TolC system	*macA*	Macrolide export protein MacA, natural resistance to macrolides (i.e. erythromycin, tylosin).	[91]
MacAB-TolC system	*macB*	Macrolide export ATP-binding/permease protein MacB. MacB is a non-canonical ABC transporter that contains transmembrane domains (TMD), which form a pore in the inner membrane, and an ATP-binding domain (NBD), which is responsible for energy generation. Confers resistance against macrolides.	[91]
	mdfA	Multidrug translocase, multidrug (Mdr) transporters.	[94]
	cmr	Multidrug pump Cmr.	[95,96]
mdt operon	*mdtA*	Multidrug resistance protein MdtA.	[97]

mdt operon	*mdtB*	Multidrug resistance protein MdtB.	[97]
mdt operon	*mdtC*	Multidrug resistance protein MdtC	[97]
mdt operon	*mdtL*	Multidrug resistance protein MdtL	[98]
mdt operon	*mdtM*	Multidrug resistance protein MdtM	[99]
	phoE (ompE)	Outer membrane porin PhoE. Uptake of inorganic phosphate, phosphorylated compounds, and some other negatively charged solutes. Outer membrane phosphate channel.	[100]
	oprM	AdeC/adeK/oprM family multidrug efflux complex outer membrane factor.	[101]
	gdx (sugE)	Guanidinium exporter. Guanidinium ion exporter. Couples guanidinium export to the proton motive force, exchanging one guanidinium ion for two protons. Quaternary ammonium salt-resistance protein-encoding genes.	[91]
	skp (ompH)	Chaperone protein Skp. Molecular chaperone that interacts specifically with outer membrane proteins, thus maintaining the solubility of early folding intermediates during passage through the periplasm.	[102]
	gidB (rsmG)	Ribosomal RNA small subunit methyltransferase G. Specifically methylates the N7 position of guanine in position 527 of 16S rRNA. Streptomycin resistance. Gene conferring resistance via absence.	[103]
	pgsA	CDP-diacylglycerol--glycerol-3-phosphate 3-phosphatidyltransferase. This protein catalyzes the committed step to the synthesis of the acidic phospholipids.	[104-106]
	hilD	DNA-binding protein H-NS. Binds tightly to dsDNA. Has a strong preference for DNA that has been recently acquired by horizontal gene transfer, binding strongly to Salmonella pathogenicity islands 1 and 2 (SPI1 and SPI2). It plays a role in the thermal control of pili production.	[107]
	oxyR	DNA-binding transcriptional regulator OxyR.	[108,109]
	AAC(6′)-Ib-cr	Resistance to ciprofloxacin, aac(6′)-Ib-cr is the most prevalent plasmid-mediated fluoroquinolone (FQ) resistance mechanism in Enterobacteriaceae.	[110,111]
	HN-S	Regulator modulating expression of antibiotic resistance genes.	[112]

References:

68. Ando, H.; Kondo, Y.; Suetake, T.; Toyota, E.; Kato, S.; Mori, T.; Kirikae, T. Identification of katG mutations associated with high-level isoniazid resistance in Mycobacterium tuberculosis. *Antimicrob. Agents Chemother.* **2010**, *54*, 1793–1799, doi:10.1128/AAC.01691-09.

69 Loewen, P.C.; De Silva, P.M.; Donald, L.J.; Switala, J.; Villanueva, J.; Fita, I.; Kumar, A. KatG-Mediated Oxidation Leading to Reduced Susceptibility of Bacteria to Kanamycin. *ACS Omega* **2018**, *3*, 4213–4219, doi:10.1021/acsomega.8b00356.

70. Holden, E.R.; Webber, M.A. MarA, RamA, and SoxS as Mediators of the Stress Response: Survival at a Cost. *Front. Microbiol.* **2020**, *11*, 1–10, doi:10.3389/fmicb.2020.00828.

71. Sharma, P.; Haycocks, J.R.J.; Middlemiss, A.D.; Kettles, R.A.; Sellars, L.E.; Ricci, V.; Piddock, L.J.V.; Grainger, D.C. The multiple antibiotic resistance operon of enteric bacteria controls DNA repair and outer membrane integrity. *Nat. Commun.* **2017**, *8*, 1–11, doi:10.1038/s41467-017-01405-7.

72. Eaves, D.J.; Ricci, V.; Piddock, L.J. V soxS in Salmonella enterica Serovar Typhimurium : Role in Multiple Antibiotic Resistance Serovar Typhimurium : Role in Multiple Antibiotic Resistance. *Antimicrob. Agent Chemother.* **2004**, *48*, 1145–1150, doi:10.1128/AAC.48.4.1145.

73. Galakatos, N.G.; Daub, E.; Botstein, D.; Walsh, C.T. Alanine Racemase from. **1986**, 3255–3260.

74. Ray, S.; Das, S.; Panda, P.K.; Suar, M. Identification of a new alanine racemase in Salmonella Enteritidis and its contribution to pathogenesis. *Gut Pathog.* **2018**, *10*, 1–17, doi:10.1186/s13099-018-0257-6.

75. Zawadzke, L.E.; Bugg, T.D.H.; Walsh, C.T. Existence of Two D-Alanine:D-Alanine Ligases in Escherichia coli: Cloning and Sequencing of the ddlA Gene and Purification and Characterization of the DdlA and DdlB Enzymes. *Biochemistry* **1991**, *30*, 1673–1682, doi:10.1021/bi00220a033.

76. Brown, A.C.; Parish, T. Dxr is essential in Mycobacterium tuberculosis and fosmidomycin resistance is due to a lack of uptake. *BMC Microbiol.* **2008**, *8*, 1–9, doi:10.1186/1471-2180-8-78.

77. Macvanin, M.; Björkman, J.; Eriksson, S.; Rhen, M.; Andersson, D.I.; Hughes, D. Fusidic Acid-Resistant Mutants of Salmonella enterica Serovar Typhimurium with Low Fitness In Vivo are Defective in RpoS Induction. *Antimicrob. Agents Chemother.* **2003**, *47*, 3743–3749, doi:10.1128/AAC.47.12.3743-3749.2003.

78. Harvey, K.L.; Jarocki, V.M.; Charles, I.G.; Djordjevic, S.P. The diverse functional roles of elongation factor tu (Ef-

tu) in microbial pathogenesis. *Front. Microbiol.* **2019**, *10*, 1–19, doi:10.3389/fmicb.2019.02351.
79. Manna, M.S.; Tamer, Y.T.; Gaszek, I.; Poulides, N.; Ahmed, A.; Wang, X.; Toprak, F.C.R.; Woodard, D.N.R.; Koh, A.Y.; Williams, N.S.; et al. A trimethoprim derivative impedes antibiotic resistance evolution. *Nat. Commun.* **2021**, *12*, 1–10, doi:10.1038/s41467-021-23191-z.
80. Vedantam, G.; Guay, G.G.; Austria, N.E.; Doktor, S.Z.; Nichols, B.P. Characterization of mutations contributing to sulfathiazole resistance in Escherichia coli. *Antimicrob. Agents Chemother.* **1998**, *42*, 88–93, doi:10.1128/aac.42.1.88.
81. Weigel, L.M.; Steward, C.D.; Tenover, F.C. gyrA mutations associated with fluoroquinolone resistance in eight species of Enterobacteriaceae. *Antimicrob. Agents Chemother.* **1998**, *42*, 2661–2667, doi:10.1128/aac.42.10.2661.
82. Feng, X.; Zhang, Z.; Li, X.; Song, Y.; Kang, J.; Yin, D.; Gao, Y.; Shi, N.; Duan, J. Mutations in gyrB play an important role in ciprofloxacin-resistant pseudomonas aeruginosa. *Infect. Drug Resist.* **2019**, *12*, 261–272, doi:10.2147/IDR.S182272.
83. Webber, M.A.; Randall, L.P.; Cooles, S.; Woodward, M.J.; Piddock, L.J.V. Triclosan resistance in Salmonella enterica serovar Typhimurium. *J. Antimicrob. Chemother.* **2008**, *62*, 83–91, doi:10.1093/jac/dkn137.
84. Takahata, S.; Ida, T.; Hiraishi, T.; Sakakibara, S.; Maebashi, K.; Terada, S.; Muratani, T.; Matsumoto, T.; Nakahama, C.; Tomono, K. Molecular mechanisms of fosfomycin resistance in clinical isolates of Escherichia coli. *Int. J. Antimicrob. Agents* **2010**, *35*, 333–337, doi:10.1016/j.ijantimicag.2009.11.011.
85. Hafeezunnisa, M.; Sen, R. The Rho-Dependent Transcription Termination Is Involved in Broad-Spectrum Antibiotic Susceptibility in Escherichia coli. *Front. Microbiol.* **2020**, *11*, 1–20, doi:10.3389/fmicb.2020.605305.
86. Siu, G.K.H.; Zhang, Y.; Lau, T.C.K.; Lau, R.W.T.; Ho, P.L.; Yew, W.W.; Tsui, S.K.W.; Cheng, V.C.C.; Yuen, K.Y.; Yam, W.C. Mutations outside the rifampicin resistance-determining region associated with rifampicin resistance in Mycobacterium tuberculosis. *J. Antimicrob. Chemother.* **2011**, *66*, 730–733, doi:10.1093/jac/dkq519.
87. De Vos, M.; Müller, B.; Borrell, S.; Black, P.A.; Van Helden, P.D.; Warren, R.M.; Gagneux, S.; Victor, T.C. Putative compensatory mutations in the rpoc gene of rifampin-resistant mycobacterium tuberculosis are associated with ongoing transmission. *Antimicrob. Agents Chemother.* **2013**, *57*, 827–832, doi:10.1128/AAC.01541-12.
88. Radeck, J.; Gebhard, S.; Orchard, P.S.; Kirchner, M.; Bauer, S.; Mascher, T.; Fritz, G. Anatomy of the bacitracin resistance network in Bacillus subtilis. *Mol. Microbiol.* **2016**, *100*, 607–620, doi:10.1111/mmi.13336.
89. Du, D.; Wang, Z.; James, N.R.; Voss, J.E.; Klimont, E.; Ohene-agyei, T.; Venter, H.; Chiu, W.; Luisi, B.F. Structure of the AcrAB – TolC multidrug efflux pump. *Nature* **2014**, doi:10.1038/nature13205.
90. Atac, N.; Kurt-Azap, O.; Dolapci, I.; Yesilkaya, A.; Ergonul, O.; Gonen, M.; Can, F. The Role of AcrAB–TolC Efflux Pumps on Quinolone Resistance of E. coli ST131. *Curr. Microbiol.* **2018**, *75*, 1661–1666, doi:10.1007/s00284-018-1577-y.
91. Lu, Y.; Wen, Y.; Hu, G.; Liu, Y.; Beier, R.C.; Hou, X. Genomic sequence analysis of the multidrug-resistance region of avian Salmonella enterica serovar Indiana strain MHYL. *Microorganisms* **2019**, *7*, doi:10.3390/microorganisms7080248.
92. Alav, I.; Bavro, V.N.; Blair, J.M.A. Interchangeability of periplasmic adaptor proteins AcrA and AcrE in forming functional efflux pumps with AcrD in Salmonella enterica serovar Typhimurium. *J. Antimicrob. Chemother.* **2021**, *76*, 2558–2564, doi:10.1093/jac/dkab237.
93. Alenazy, R. Antibiotic resistance in Salmonella: Targeting multidrug resistance by understanding efflux pumps, regulators and the inhibitors. *J. King Saud Univ. - Sci.* **2022**, *34*, 102275, doi:10.1016/j.jksus.2022.102275.
94. Yardeni, E.H.; Zomot, E.; Bibi, E. The fascinating but mysterious mechanistic aspects of multidrug transport by MdfA from Escherichia coli. *Res. Microbiol.* **2018**, *169*, 455–460, doi:10.1016/j.resmic.2017.09.004.
95. Nilsen, I.W.; Bakke, I.; Vader, A.; Olsvik, Ø.; El-Gewely, M.R. Isolation of cmr, a novel Escherichia coli chloramphenicol resistance gene encoding a putative efflux pump. *J. Bacteriol.* **1996**, *178*, 3188–3193, doi:10.1128/jb.178.11.3188-3193.1996.
96. Ayhan, D.H.; Tamer, Y.T.; Akbar, M.; Bailey, S.M.; Wong, M.; Daly, S.M.; Greenberg, D.E.; Toprak, E. Sequence-Specific Targeting of Bacterial Resistance Genes Increases Antibiotic Efficacy. *PLoS Biol.* **2016**, *14*, 1–18, doi:10.1371/journal.pbio.1002552.
97. Nishino, K.; Nikaido, E.; Yamaguchi, A. Regulation of multidrug efflux systems involved in multidrug and metal resistance of Salmonella enterica serovar typhimurium. *J. Bacteriol.* **2007**, *189*, 9066–9075, doi:10.1128/JB.01045-07.
98. Jibril, A.H.; Okeke, I.N.; Dalsgaard, A.; Menéndez, V.G.; Olsen, J.E. Genomic analysis of antimicrobial resistance and resistance plasmids in salmonella serovars from poultry in Nigeria. *Antibiotics* **2021**, *10*, 1–22, doi:10.3390/antibiotics10020099.
99. Holdsworth, S.R.; Law, C.J. Functional and biochemical characterisation of the Escherichia coli major facilitator superfamily multidrug transporter MdtM. *Biochimie* **2012**, *94*, 1334–1346, doi:10.1016/j.biochi.2012.03.001.
100. Spierings, G.; Elders, R.; van Lith, B.; Hofstra, H.; Tommassen, J. Characterization of the Salmonella typhimurium phoE gene and development of Salmonella-specific DNA probes. *Gene* **1992**, *122*, 45–52, doi:10.1016/0378-1119(92)90030-S.

101. Nishino, K.; Latifi, T.; Groisman, E.A. Virulence and drug resistance roles of multidrug efflux systems of Salmonella enterica serovar Typhimurium. *Mol. Microbiol.* **2006**, *59*, 126–141, doi:10.1111/j.1365-2958.2005.04940.x.
102. Kapach, G.; Nuri, R.; Schmidt, C.; Danin, A.; Ferrera, S.; Savidor, A.; Gerlach, R.G.; Shai, Y. Loss of the Periplasmic Chaperone Skp and Mutations in the Efflux Pump AcrAB-TolC Play a Role in Acquired Resistance to Antimicrobial Peptides in Salmonella typhimurium. *Front. Microbiol.* **2020**, *11*, doi:10.3389/fmicb.2020.00189.
103. Rodríguez-García, Á.; Mares-Alejandre, R.E.; Muñoz-Muñoz, P.L.A.; Ruvalcaba-Ruiz, S.; González-Sánchez, R.A.; Bernáldez-Sarabia, J.; Meléndez-López, S.G.; Licea-Navarro, A.F.; Ramos-Ibarra, M.A. Molecular analysis of streptomycin resistance genes in clinical strains of mycobacterium tuberculosis and biocomputational analysis of the mtgidb l101f variant. *Antibiotics* **2021**, *10*, doi:10.3390/antibiotics10070807.
104. Sulaiman, J.E.; Long, L.; Wu, L.; Qian, P.Y.; Lam, H. Comparative proteomic investigation of multiple methicillin-resistant Staphylococcus aureus strains generated through adaptive laboratory evolution. *iScience* **2021**, *24*, 102950, doi:10.1016/j.isci.2021.102950.
105. Yang, B.; Yao, H.; Li, D.; Liu, Z. The phosphatidylglycerol phosphate synthase PgsA utilizes a trifurcated amphipathic cavity for catalysis at the membrane-cytosol interface. *Curr. Res. Struct. Biol.* **2021**, *3*, 312–323, doi:10.1016/j.crstbi.2021.11.005.
106. Tran, T.T.; Mishra, N.N.; Seepersaud, R.; Diaz, L.; Rios, R.; Dinh, A.Q.; Garcia-de-la-Maria, C.; Rybak, M.J.; Miro, J.M.; Shelburne, S.A.; et al. Mutations in cdsA and pgsA Correlate with Daptomycin Resistance in Streptococcus mitis and S. Oralis. *Antimicrob. Agents Chemother.* **2019**, *63*, 1–6, doi:10.1128/AAC.01531-18.
107. Kalafatis, M.; Slauch, J.M. Long-distance effects of H-NS binding in the control of hilD expression in the salmonella SPI1 locus. *J. Bacteriol.* **2021**, *203*, doi:10.1128/JB.00308-21.
108. Johnson, J.R.; Clabots, C.; Rosen, H. Effect of inactivation of the global oxidative stress regulator oxyR on the colonization ability of Escherichia coli O1:K1:H7 in a mouse model of ascending urinary tract infection. *Infect. Immun.* **2006**, *74*, 461–468, doi:10.1128/IAI.74.1.461-468.2006.
109. Anand, A.; Chen, K.; Catoiu, E.; Sastry, A. V.; Olson, C.A.; Sandberg, T.E.; Seif, Y.; Xu, S.; Szubin, R.; Yang, L.; et al. OxyR Is a Convergent Target for Mutations Acquired during Adaptation to Oxidative Stress-Prone Metabolic States. *Mol. Biol. Evol.* **2020**, *37*, 660–666, doi:10.1093/molbev/msz251.
110. Robicsek, A.; Strahilevitz, J.; Jacoby, G.A.; Macielag, M.; Abbanat, D.; Chi, H.P.; Bush, K.; Hooper, D.C. Fluoroquinolone-modifying enzyme: A new adaptation of a common aminoglycoside acetyltransferase. *Nat. Med.* **2006**, *12*, 83–88, doi:10.1038/nm1347.
111. Wong, M.H.Y.; Chan, E.W.C.; Liu, L.Z.; Chen, S. PMQR genes oqxAB and aac(6')Ib-cr accelerate the development of fluoroquinolone resistance in salmonella Typhimurium. *Front. Microbiol.* **2014**, *5*, 1–7, doi:10.3389/fmicb.2014.00521.
112. Nishino, K.; Yamaguchi, A. Role of Histone-Like Protein H-NS in Multidrug Resistance of Escherichia coli. **2004**, *186*, 1423–1429, doi:10.1128/JB.186.5.1423.
