# Supplementary material for: Comparison of Phenotype and Genotype Virulence and Antimicrobial Factors of Salmonella Typhimurium Isolated from Human Milk
Source: Int J Mol Sci. 2023 Mar 7;24(6):5135. doi: 10.3390/ijms24065135 (PMC10048834; doi:10.3390/ijms24065135)
Supplement: Supplementary file 1 [file ijms-24-05135-s001.zip › Table S1. SPI strain of Salmonella enterica subsp. enterica Typhimurium 4i1,2_69M using Center for Genomic Epidemiology.pdf]

Center for Genomic Epidemiology

Detection: ***Salmonella Pathogenicity Islands***

Salmonella Pathogenicity Islands									
SPI	Identity	Query / Template length	Contig	Position in contig	Organism	Insertion Site	Category Function	Note	Accession number
C63PI	100	4000 / 4000	69M_SET_cns	3006066..3010065	Salmonella-enterica-Typhimurium-SL1344	fhlA	1		AF128999
CS54_island	99.92	25255 / 25252	69M_SET_cns	2627012..2652263	Salmonella-enterica-Typhimurium-ATCC_14028	xseA-yfgK	29		AF140550
Not_named	100	330 / 330	69M_SET_cns	1482746..1483075	Salmonella-enterica-Enteritidis-CMCC50041		26		JQ071613
SPI-1	99.96	2705 / 2705	69M_SET_cns	3010641..3013345	Salmonella-enterica-Typhimurium-SL1344	fhlA/mutS	5	N.B. This record is part of a PAI which includes several records: U16278,U16303	AF148689
SPI-1	99.77	440 / 440	69M_SET_cns	3015995..3016434	Salmonella-enterica-Gallinarum-SGB_1	Not_published	5		AY956822
SPI-1	100	430 / 430	69M_SET_cns	3018105..3018534	Salmonella-enterica-Gallinarum-SGE_2	Not_published	5		AY956823
SPI-1	100	470 / 470	69M_SET_cns	3026813..3027282	Salmonella-enterica-Gallinarum-SGB_4	Not_published	5		AY956824
SPI-1	99.28	415 / 415	69M_SET_cns	3038600..3039014	Salmonella-enterica-Gallinarum-SGB_8	Not_published	5		AY956825
SPI-1	98.84	259 / 257	69M_SET_cns	3039894..3040152	Salmonella-enterica-Typhimurium-J4STEHO		27		JN982040
SPI-1	99.43	3155 / 3141	69M_SET_cns	3049734..3052886	Salmonella-enterica-Typhimurium-SL1344	fhlA/mutS	5	N.B. This record is part of a PAI which includes several records: U16278,AF148689	U16303
SPI-12	97.64	9345 / 11075	69M_SET_cns	2342056..2351393	Salmonella-enterica-Choleraesuis-SC_B67	tRNA-pro	8	gi|62178570:2354604-2365678 - go to http://www.paidb.re.kr/about_paidb.php for link to partial flatfile	NC_006905
SPI-13	99.41	338 / 338	69M_SET_cns	3279563..3279900	Salmonella-enterica-Gallinarum-SGD_3	tRNA-pheV	9		AY956832
SPI-13	100	404 / 404	69M_SET_cns	3278852..3279255	Salmonella-enterica-Gallinarum-SGG_1	tRNA-pheV	11		AY956833

SPI-13	100	341 / 341	69M_SET_cns	3277144..3277484	Salmonella-enterica-Gallinarum-SGA_10	tRNA-pheV	10		AY956834
SPI-14	100	501 / 501	69M_SET_cns	926728..927228	Salmonella-enterica-Gallinarum-SGA_8	Not_published	12		AY956835
SPI-14	99.55	441 / 441	69M_SET_cns	932304..932744	Salmonella-enterica-Gallinarum-SGC_8	Not_published	13		AY956836
SPI-2	98.9	637 / 637	69M_SET_cns	1497019..1497655	Salmonella-enterica-Gallinarum-SGB_10	tRNA-valV	14		AY956826
SPI-2	100	642 / 642	69M_SET_cns	1489709..1490350	Salmonella-enterica-Gallinarum-SGC_2	tRNA-valV	14		AY956827
SPI-2	100	395 / 396	69M_SET_cns	1494342..1494736	Salmonella-enterica-Gallinarum-SGC_9	tRNA-valV	14		AY956828
SPI-2	99.76	425 / 425	69M_SET_cns	1487684..1488108	Salmonella-enterica-Gallinarum-SGH_1	tRNA-valV	14		AY956829
SPI-2	99.82	547 / 547	69M_SET_cns	1477547..1478093	Salmonella-enterica-Gallinarum-SGD_8	tRNA-valV	14		AY956830
SPI-2	100	384 / 384	69M_SET_cns	1480008..1480391	Salmonella-enterica-Typhimurium-St11		23		JN673272
SPI-2	100	1252 / 1252	69M_SET_cns	1475967..1477218	Salmonella-enterica-Typhimurium-	tRNA-valV	14	N.B. This record is part of a PAI which includes several records: AJ224892,AJ224978,U51927,X99944,X99945,Y09357	Z95891
SPI-3	100	738 / 738	69M_SET_cns	3965704..3966441	Salmonella-enterica-Typhimurium-14028s	tRNA_selC	15	N.B. This record is part of a PAI which includes several records: AF106566,Y13864	AJ000509
SPI-3	100	1514 / 1514	69M_SET_cns	3948169..3949682	Salmonella-enterica-Typhimurium-14028s	tRNA_selC	15	N.B. This record is part of a PAI which includes several records: AF106566,AJ000509	Y13864
SPI-4	100	24660 / 24660	69M_SET_cns	4476929..4501588	Salmonella-enterica-Typhimurium-ST4/74	Not_published	17		AJ576316
SPI-5	100	9069 / 9069	69M_SET_cns	1175309..1184377	Salmonella-Typhimurium-LT2	tRNA-serT	18	gi|16763390:1175321-1184389 - go to http://www.paidb.re.kr/about_paidb.php for link to partial flatfile	NC_003197
SPI-9	98.58	12647 / 15696	69M_SET_cns	2831233..2843879	Salmonella-Typhi-CT18	Not_published	22	gi|16758993:2743495-2759190 - go to http://www.paidb.re.kr/about_paidb.php for link to partial flatfile	NC_003198

extended output

C63PI_AF128999

```
template   TTTTGCGCGTAGGCGAACAGCGCGTCGTAATGCCCCTGCACCGTTGACGCCTGTGGGCAC
query      TTTTGCGCGTAGGCGAACAGCGCGTCGTAATGCCCCTGCACCGTTGACGCCTGTGGGCAC
```

template TGGCTTTCATACAGCGCGATCATTTTTTTGATCGTCAGTTTTTCACGAGCGATACGTTTA
query TGGCTTTCATACAGCGCGATCATTTTTTTGATCGTCAGTTTTTCACGAGCGATACGTTTA

template CCAGGCATCGTGCTCTCTCCGAACATTAAGGTGTATTTATTTTACACCTTATCCCTCTTT
query CCAGGCATCGTGCTCTCTCCGAACATTAAGGTGTATTTATTTTACACCTTATCCCTCTTT

template AGCACTATCACTGCATATTGTCGCCATTTCGCAAATAAGAATTATTTTCATTTATCCATA
query AGCACTATCACTGCATATTGTCGCCATTTCGCAAATAAGAATTATTTTCATTTATCCATA

template CCTTGTGCTATATAACATAGCAAAGGCTATATTCGATGATTAATTAACCACATTGTTGCG
query CCTTGTGCTATATAACATAGCAAAGGCTATATTCGATGATTAATTAACCACATTGTTGCG

template AGGGATACTATGACGAATCTACATCGTCTGAAAACACTCCTGATTGCCGGTATTGTCGCG
query AGGGATACTATGACGAATCTACATCGTCTGAAAACACTCCTGATTGCCGGTATTGTCGCG

template ATACTCGCGCTATCGCCAGCCTATGCAAAAGAGAAATTTAAAGTTATCACCACGTTTACC
query ATACTCGCGCTATCGCCAGCCTATGCAAAAGAGAAATTTAAAGTTATCACCACGTTTACC

template GTTATTGCCGACATGGCGAAAAACGTGGCGGGCGACGCAGCGGAAGTCAGCTCGATTACC
query GTTATTGCCGACATGGCGAAAAACGTGGCGGGCGACGCAGCGGAAGTCAGCTCGATTACC

template AAACCCGGCGCTGAAATCCATGAGTATCAGCCAACGCCCGGCGATATTAAACGAGCGCAG
query AAACCCGGCGCTGAAATCCATGAGTATCAGCCAACGCCCGGCGATATTAAACGAGCGCAG

template GGGGCACAGCTTATCCTCGCGAATGGTCTGAACCTGGAGCGATGGTTCGCCCGCTTTTAT
query GGGGCACAGCTTATCCTCGCGAATGGTCTGAACCTGGAGCGATGGTTCGCCCGCTTTTAT

template CAGCACCTTTCCGGCGTGCCGGAAGTCGTCGTCTCCACCGGCGTCAAACCGATGGGCATT
query CAGCACCTTTCCGGCGTGCCGGAAGTCGTCGTCTCCACCGGCGTCAAACCGATGGGCATT

template ACCGAAGGCCCGTATAACGGTAAACCGAACCCGCACGCCTGGATGTCGGCAGAAAACGCG
query ACCGAAGGCCCGTATAACGGTAAACCGAACCCGCACGCCTGGATGTCGGCAGAAAACGCG

template CTGATTTATGTCGATAACATTCGCGACGCCCTGGTGAAGTACGATCCGGATAATGCGCAG
query CTGATTTATGTCGATAACATTCGCGACGCCCTGGTGAAGTACGATCCGGATAATGCGCAG

template ATCTATAAGCAAAATGCCGAACGCTATAAAGCGAAAATTCGCCAGATGGCCGATCCGTTG
query ATCTATAAGCAAAATGCCGAACGCTATAAAGCGAAAATTCGCCAGATGGCCGATCCGTTG

template CGTGCCGAACTGGAAAAAATTCCCGCCGATCAGCGCTGGTTGGTCACCAGTGAAGGCGCG
query CGTGCCGAACTGGAAAAAATTCCCGCCGATCAGCGCTGGTTGGTCACCAGTGAAGGCGCG

template TTCTCTTACCTGGCGCGCGATAACGACATGAAAGAGCTTTATCTCTGGCCAATTAACGCC
query TTCTCTTACCTGGCGCGCGATAACGACATGAAAGAGCTTTATCTCTGGCCAATTAACGCC

template GATCAACAGGGGACGCCAAAACAGGTGCGTAAAGTGATTGATACCATTAAAAAGCACCAT
query GATCAACAGGGGACGCCAAAACAGGTGCGTAAAGTGATTGATACCATTAAAAAGCACCAT

template ATTCCCGCCATCTTTAGCGAGAGTACGGTTTCCGATAAACCGGCCCGTCAGGTCGCGCGT
query ATTCCCGCCATCTTTAGCGAGAGTACGGTTTCCGATAAACCGGCCCGTCAGGTCGCGCGT

template GAATCCGGCGCGCATTATGGCGGCGTGCTGTATGTCGATTCTCTGAGCGCCGCTGACGGC
query GAATCCGGCGCGCATTATGGCGGCGTGCTGTATGTCGATTCTCTGAGCGCCGCTGACGGC

template CCTGTGCCAACCTATCTGGATCTGCTGCGCGTCACGACCGAAACCATCGTCAACGGCATT
query CCTGTGCCAACCTATCTGGATCTGCTGCGCGTCACGACCGAAACCATCGTCAACGGCATT

template AACGACGGACTGAGGAGTCAACAATGAGTCAATCTGCGATTACCGTTGATCAAGTCACGG
query AACGACGGACTGAGGAGTCAACAATGAGTCAATCTGCGATTACCGTTGATCAAGTCACGG

template TGACGTATCGCAACGGTCATACCGCCCTACGGGACGCCACTTTTCAGGTGCCTGGCGGTT
query TGACGTATCGCAACGGTCATACCGCCCTACGGGACGCCACTTTTCAGGTGCCTGGCGGTT

template CAATCGCCGCGCTGGTAGGCGTAAATGGTTCCGGTAAGTCTACGTTATTTAAAGCGTTAA
query CAATCGCCGCGCTGGTAGGCGTAAATGGTTCCGGTAAGTCTACGTTATTTAAAGCGTTAA

template TGGGCTTTGTGCATCTTGCGCAGGGCGATATCACTATCCTGCAACAGTCAGTCAACAAGG
query TGGGCTTTGTGCATCTTGCGCAGGGCGATATCACTATCCTGCAACAGTCAGTCAACAAGG

template CACTGAAAAAAAACCTCATTGCTTATGTTCCGCAATCGGAAGAAGTGGACTGGTCATTTC

```
query     CACTGAAAAAAAACCTCATTGCTTATGTTCCGCAATCGGAAGAAGTGGACTGGTCATTTC

template  CGGTGCTGGTAGAAGATGTGGTGATGATGGGACGCTACGGCCATATGGGCTGGCTACGCC
query     CGGTGCTGGTAGAAGATGTGGTGATGATGGGACGCTACGGCCATATGGGCTGGCTACGCC

template  GTCCCACTGCGCATGATCACGCCTGTGTTGACGCCGCGCTGGCGCGGGTGGATATGCAGG
query     GTCCCACTGCGCATGATCACGCCTGTGTTGACGCCGCGCTGGCGCGGGTGGATATGCAGG

template  AGTATCGTCACAGGCAGATTGGCGAGCTGTCCGGCGGGCAGAAAAAACGTGTGTTTCTTG
query     AGTATCGTCACAGGCAGATTGGCGAGCTGTCCGGCGGGCAGAAAAAACGTGTGTTTCTTG

template  CCAGGGCCATCGCTCAGGATGGACAGGTGATCCTGCTGGATGAACCTTTTACCGGCGTGG
query     CCAGGGCCATCGCTCAGGATGGACAGGTGATCCTGCTGGATGAACCTTTTACCGGCGTGG

template  ATGTAAAAACCGAAGCGCGAATCATCGATCTACTGCGGGAACTGCGCGATGAAGGCCGCA
query     ATGTAAAAACCGAAGCGCGAATCATCGATCTACTGCGGGAACTGCGCGATGAAGGCCGCA

template  CGATGCTGGTCTCAACGCATAATCTCGGCTCCGTCACCGAGTTTTGCGACTATACGGTAA
query     CGATGCTGGTCTCAACGCATAATCTCGGCTCCGTCACCGAGTTTTGCGACTATACGGTAA

template  TGATTAAAGGAACCGTACTGGCCAGCGGCCCTACCGAAACCACGTTTACCGCCGCGAATC
query     TGATTAAAGGAACCGTACTGGCCAGCGGCCCTACCGAAACCACGTTTACCGCCGCGAATC

template  TGGAACAGGCGTTTAGCGGCGTTCTACGTCATATCGCGTTAAGCGGAGGTGAAGAGCACA
query     TGGAACAGGCGTTTAGCGGCGTTCTACGTCATATCGCGTTAAGCGGAGGTGAAGAGCACA

template  TCATCACCGATGACGAGCGCCCCTTTATTTCCCGGCGCGTGGCCAGCGGAGGAAAATCGT
query     TCATCACCGATGACGAGCGCCCCTTTATTTCCCGGCGCGTGGCCAGCGGAGGAAAATCGT

template  CATGAACTGGCTTGTTGAGCCGTTTGGCTACCAGTATATGCTCAACGCGATGTGGGTCTC
query     CATGAACTGGCTTGTTGAGCCGTTTGGCTACCAGTATATGCTCAACGCGATGTGGGTCTC

template  CGCGATGGTGGGCGGTCTGTGCGCGTTTCTCTCCTGCTATTTGATGCTCAAAGGCTGGTC
query     CGCGATGGTGGGCGGTCTGTGCGCGTTTCTCTCCTGCTATTTGATGCTCAAAGGCTGGTC

template  GCTCATTGGCGATGCGCTATCTCACTCTATTGTGCCTGGCGTCGCTGGCGCCTGGATGTT
query     GCTCATTGGCGATGCGCTATCTCACTCTATTGTGCCTGGCGTCGCTGGCGCCTGGATGTT

template  AGGGCTGCCCTTCTCGCTCGGCGCGTTTCTTTCCGGCGGACTGGCGGCAGGCAGTATGCT
query     AGGGCTGCCCTTCTCGCTCGGCGCGTTTCTTTCCGGCGGACTGGCGGCAGGCAGTATGCT

template  CTTTCTTAACCAACGCTCACGCCTGAAAGAAGACGCGATTATCGGGCTTATCTTCTCCTC
query     CTTTCTTAACCAACGCTCACGCCTGAAAGAAGACGCGATTATCGGGCTTATCTTCTCCTC

template  TTTTTTTGGCGTCGGCCTTTTTATGGTGTCGCTCAATCCGATGTCGGTGAATATCCAGAC
query     TTTTTTTGGCGTCGGCCTTTTTATGGTGTCGCTCAATCCGATGTCGGTGAATATCCAGAC

template  GATTATTCTCGGCAACGTGCTGGCGATCGCACCAGCGGATATTGCGCAACTGGCGATTAT
query     GATTATTCTCGGCAACGTGCTGGCGATCGCACCAGCGGATATTGCGCAACTGGCGATTAT

template  CGGCGCAGTCTCGCTGACGATTCTGCTGTTAAAGTGGAAAGATCTGATGGTCGTCTTTTT
query     CGGCGCAGTCTCGCTGACGATTCTGCTGTTAAAGTGGAAAGATCTGATGGTCGTCTTTTT

template  CGATGAAACCCATGCGCGTTCTATCGGGCTTAATCCGGGTCGACTAAAGCTGTTGTTTTT
query     CGATGAAACCCATGCGCGTTCTATCGGGCTTAATCCGGGTCGACTAAAGCTGTTGTTTTT

template  CACTCTATTGTCCGTCTCTACCGTGGCGGCCCTGCAAACCGTTGGGGCGTTCCTGGTGAT
query     CACTCTATTGTCCGTCTCTACCGTGGCGGCCCTGCAAACCGTTGGGGCGTTCCTGGTGAT

template  CTGTCTCGTCGTCACGCCGGGCGCGACGGCCTGGTTACTCACCGACCGTTTTCCACGTCT
query     CTGTCTCGTCGTCACGCCGGGCGCGACGGCCTGGTTACTCACCGACCGTTTTCCACGTCT

template  GCTCATGATTGCCGTCGTGATTGGCAGCCTGACCAGTTTTCTGGGCGCATGGCTTAGCTA
query     GCTCATGATTGCCGTCGTGATTGGCAGCCTGACCAGTTTTCTGGGCGCATGGCTTAGCTA

template  CTGGCTGGATGGCGCCACGGGTGGAATTATTGTCGTCATGCAAACCTTACTGTTCATCAC
query     CTGGCTGGATGGCGCCACGGGTGGAATTATTGTCGTCATGCAAACCTTACTGTTCATCAC

template  AGCCTTTATTTTCGCCCCGAAACACGGTCTGTTAGCCAACCGTCGACGCGCCCGCCTGCA
query     AGCCTTTATTTTCGCCCCGAAACACGGTCTGTTAGCCAACCGTCGACGCGCCCGCCTGCA
```

```
template    GAAGGAGCCGACATGTTCTTAACAACGTTACTGGAACCCTTTCAGTTTGATTTTATGGTG
query       GAAGGAGCCGACATGTTCTTAACAACGTTACTGGAACCCTTTCAGTTTGATTTTATGGTG

template    AACGCCCTTATGGTTTCGGTCATTGTGGCGATTCCCTGCGCCTTACTGTCCGTGTTTTTA
query       AACGCCCTTATGGTTTCGGTCATTGTGGCGATTCCCTGCGCCTTACTGTCCGTGTTTTTA

template    GTGTTAAAAGGCTGGGCGTTAATGGGCGATGCAATGAGTCATGCGGTATTCCCCGGGGTC
query       GTGTTAAAAGGCTGGGCGTTAATGGGCGATGCAATGAGTCATGCGGTATTCCCCGGGGTC

template    GTGCTGGCATATATTGTGGGGATCCCCCTGGCGATTGGCGCTTTCATTGCCGGATTATTT
query       GTGCTGGCATATATTGTGGGGATCCCCCTGGCGATTGGCGCTTTCATTGCCGGATTATTT

template    TGCGCTATCGCCACCGGCTATCTGGACGATAACAGCCGCATCAAACGTGATACGGTGATG
query       TGCGCTATCGCCACCGGCTATCTGGACGATAACAGCCGCATCAAACGTGATACGGTGATG

template    GGCATCGTCTTTTCCGGGATGTTTGGCGCAGGTCTGGTGCTCTATGTTTCTATTCAATCG
query       GGCATCGTCTTTTCCGGGATGTTTGGCGCAGGTCTGGTGCTCTATGTTTCTATTCAATCG

template    GAAGTGCATCTGGATCACATCCTGTTTGGGGATATGCTGGGCGTATCGCTGGGGGATATC
query       GAAGTGCATCTGGATCACATCCTGTTTGGGGATATGCTGGGCGTATCGCTGGGGGATATC

template    GTACAAACGTCAGTTATTGCACTGGGTATTGCGTTAATCATAGGGCTGAAATGGAAAGAT
query       GTACAAACGTCAGTTATTGCACTGGGTATTGCGTTAATCATAGGGCTGAAATGGAAAGAT

template    CTCCTGCTACACGCTTTTGACCCGCACCAGGCGAAAGCCAGCGGGCTGAATACCACACTA
query       CTCCTGCTACACGCTTTTGACCCGCACCAGGCGAAAGCCAGCGGGCTGAATACCACACTA

template    CTGCATTATGGCCTGCTATGTATGATTGCGCTGACTATCGTCGCCACGCTGAAATCGGTG
query       CTGCATTATGGCCTGCTATGTATGATTGCGCTGACTATCGTCGCCACGCTGAAATCGGTG

template    GGCATTATTCTGTCAATCTCACTGCTTATCGCCCCCGGCGCAATTGCCATTTTGCTGACG
query       GGCATTATTCTGTCAATCTCACTGCTTATCGCCCCCGGCGCAATTGCCATTTTGCTGACG

template    CGACGCTTTGCGCGCGCGCTGGGATTAGCGGTGAGTCTGTCAGTTATTACCGCTTTTGCG
query       CGACGCTTTGCGCGCGCGCTGGGATTAGCGGTGAGTCTGTCAGTTATTACCGCTTTTGCG

template    GGCGTTTATCTGTCTTTTTATCTTGATAGCGCGCCAGCGCCGACCATCGTGGTATTATTT
query       GGCGTTTATCTGTCTTTTTATCTTGATAGCGCGCCAGCGCCGACCATCGTGGTATTATTT

template    GCCATCGTGTTTATTGCGGCATTTATTTACGCAACATGGCGCGACAGGCGTAATGAAATC
query       GCCATCGTGTTTATTGCGGCATTTATTTACGCAACATGGCGCGACAGGCGTAATGAAATC

template    GTACCAGAGGCGCAAGGTTAACACTTTTTTGCCTGCTATTCAGGAAACATACGGTCATAA
query       GTACCAGAGGCGCAAGGTTAACACTTTTTTGCCTGCTATTCAGGAAACATACGGTCATAA

template    TATAGAGTAGTCTTATGGCGCTGGAAGGATTTCCTCTGGCAGGCAACCTTATAATTTCAT
query       TATAGAGTAGTCTTATGGCGCTGGAAGGATTTCCTCTGGCAGGCAACCTTATAATTTCAT

template    TACGGTTTAAGTAAAGACTTATATTCAGCTATCCTTTTTTTATGAGCGGATATAGAGAGT
query       TACGGTTTAAGTAAAGACTTATATTCAGCTATCCTTTTTTTATGAGCGGATATAGAGAGT

template    TTTTTATCGTTTAGCATAACGGCATTGTTATCGAATCGCTCATAAAGCGTTTCATTCTTT
query       TTTTTATCGTTTAGCATAACGGCATTGTTATCGAATCGCTCATAAAGCGTTTCATTCTTT

template    TTGTTTACTATACTGCTTCCCGCCGCCGGATTGGCCTCCA
query       TTGTTTACTATACTGCTTCCCGCCGCCGGATTGGCCTCCA
```

CS54_island_AF140550

```
template    TGGCCCGTCAGCAAACCGCGCTGGAACGTCTACGCCAACGGATGGGATTTGCGCTTGAGG
query       TGGCCCGTCAGCAAACCGCGCTGGAACGTCTACGCCAACGGATGGGATTTGCGCTTGAGG

template    CCAGGATTAAGCAGGCCAATCAACGCCAACAGTGCGTAAGTCAACGCCTGAGCCAGCAAA
query       CCAGGATTAAGCAGGCCAATCAACGCCAACAGTGCGTAAGTCAACGCCTGAGCCAGCAAA

template    ATCCGCAGCCGCGCATTCACCGCGCGCAGTCGCGCATCCAACAGCTTGAATATCGTCTGA
query       ATCCGCAGCCGCGCATTCACCGCGCGCAGTCGCGCATCCAACAGCTTGAATATCGTCTGA
```

```
template   CGGAGAATATCCGCTCACGGTTAAGCGAACAGCGCGAGCGTTTTGGCAACGCGGTCACGC
query      CGGAGAATATCCGCTCACGGTTAAGCGAACAGCGCGAGCGTTTTGGCAACGCGGTCACGC

template   ATCTGGAAGCGGTCAGCCCGTTAGCCACGCTGGCGCGCGGCTATACGGTTTCGACGACAA
query      ATCTGGAAGCGGTCAGCCCGTTAGCCACGCTGGCGCGCGGCTATACGGTTTCGACGACAA

template   CGGACGGCAAGGTGCTGAAAAAGATCAAACAGGTCAAAGCAGGCGATATCATGACCACGA
query      CGGACGGCAAGGTGCTGAAAAAGATCAAACAGGTCAAAGCAGGCGATATCATGACCACGA

template   GACTGGAGGACGGCTGGCTTGAAAGTGAAGTCAAATCCGTCACGCCGGGCACGTAATCCA
query      GACTGGAGGACGGCTGGCTTGAAAGTGAAGTCAAATCCGTCACGCCGGGCACGTAATCCA

template   ACACTCCCCCACCCTTTGCAGCGCGATGTTGGGTTTCGACTTCGCTTCAGCCAACCTACG
query      ACACTCCCCCACCCTTTGCAGCGCGATGTTGGGTTTCGACTTCGCCTCAGCCAACCTACG

template   CGCCACGTCGGATAAGGTGTAGCCATAATCTGACGATCAACCGGTTTGTCATTCGCCTCA
query      CGCCACGTCGGATAAGGTGTAGCCATAATCTGACGATCAACCGGTTTGTCATTCGCCTCA

template   AAACCGGGCAGGGAACACCCGCCCGGTTTTGTCTAACTTACCAGTTATAGCGCAGATTGA
query      AAACCGGGCAGGGAACACCCGCCCGGTTTTGTCTAACTTACCAGTTATAGCGCAGATTGA

template   GGCTACCGTTCAGATCGCCAAAGTCATTGCTGCCAGTCTGCCCGGCAACCTGAGCGCGAA
query      GGCTACCGTTCAGATCGCCAAAGTCATTGCTGCCAGTCTGCCCGGCAACCTGAGCGCGAA

template   CGCTCCACTGCTTATCGATATCTGCCTGCAGACCCACTTTCAGCTCCGCACGGTTAGCCG
query      CGCTCCACTGCTTATCGATATCTGCCTGCAGACCCACTTTCAGCTCCGCACGGTTAGCCG

template   GAAGATCCTGTTTCACCGTAGCATCATCAAACGATACCGACACATCATCGCTGGTGTGCA
query      GAAGATCCTGTTTCACCGTAGCATCATCAAACGATACCGACACATCATCGCTGGTGTGCA

template   GCCAGTTAGCTTCAGCAAACGGCTGGATAACCGTACGGCTGCCTTTGTACAGCTTGCCGT
query      GCCAGTTAGCTTCAGCAAACGGCTGGATAACCGTACGGCTGCCTTTGTACAGCTTGCCGT

template   CAACACGCAGACCCAGACGCGTTGTCCAACTGTCGCCATCCTGACCATCGATCCGGGTGC
query      CAACACGCAGACCCAGACGCGTTGTCCAACTGTCGCCATCCTGACCATCGATCCGGGTGC

template   CGTAGTTATCCTTCACGCTATCCGCTGAGTAATTCTGCCAGACAACCTGCGCCTGCGGCG
query      CGTAGTTATCCTTCACGCTATCCGCTGAGTAATTCTGCCAGACAACCTGCGCCTGCGGCG

template   TCAGACTGACAGTATTACCGTTGCTAAGCGCAATATCGTAGCGATAACCAGTTTCCAGCG
query      TCAGACTGACAGTATTACCGTTGCTAAGCGCAATATCGTAGCGATAACCAGTTTCCAGCG

template   AGACGGCGTTAGCGGTTGAATCATAAGATTCAGATCCCGCATCACCGCTCTCTACGCTGT
query      AGACGGCGTTAGCGGTTGAATCATAAGATTCAGATCCCGCATCACCGCTCTCTACGCTGT

template   TGTTGTAGAAACCATATTGGTACCAGCTGTCGACATACGCGCCGCTATGCGTTTGGGCAT
query      TGTTGTAGAAACCATATTGGTACCAGCTGTCGACATACGCGCCGCTATGCGTTTGGGCAT

template   CGGCAAACCAGGTGGCATAGACGCCAAGGTTGTAGCCGTCTACGTTGCCGCTACTGGTGA
query      CGGCAAACCAGGTGGCATAGACGCCAAGGTTGTAGCCGTCTACGTTGCCGCTACTGGTGA

template   ACTGGCTACCGTCTGCGCCACGGTTACCGGTGCTGTCGGTATCGGCGTTGATGTAGCTCG
query      ACTGGCTACCGTCTGCGCCACGGTTACCGGTGCTGTCGGTATCGGCGTTGATGTAGCTCG

template   CCATGACGCCAACGGTAACGCTCTGCTGGCCGTTACCCCAGGCCAGAATGTCGCCGCCTA
query      CCATGACGCCAACGGTAACGCTCTGCTGGCCGTTACCCCAGGCCAGAATGTCGCCGCCTA

template   ACTGGAACTGGGAGTAGTTGCTGTCCATATCGATATTGCCGCTGACAGCCTCGGATTCCG
query      ACTGGAACTGGGAGTAGTTGCTGTCCATATCGATATTGCCGCTGACAGCCTCGGATTCCG

template   CTTTACCCGCTTTGAAGCGCGCCCATACGCTGCCATCGGCATTACGATACTGGCTGCCCT
query      CTTTACCCGCTTTGAAGCGCGCCCATACGCTGCCATCGGCATTACGATACTGGCTGCCCT

template   CGCGGTCATAGAGGGTCTGCATTTGCAGGTTGCGCGCCATCCACTGGTTGCCCATGTACG
query      CGCGGTCATAGAGGGTCTGCATTTGCAGGTTGCGCGCCATCCACTGGTTGCCCATGTACG

template   CGCCGATATCCGCACGGTACTGCGGTGCGACATCGCCGCCGTGGTCCGGAGGCGTGACAT
query      CGCCGATATCCGCACGGTACTGCGGTGCGACATCGCCGCCGTGGTCCGGAGGCGTGACAT
```

```
template  CGCCGCCGTCGCCGCCGTCGTCCGGAGGCGTGACATTACCGCCATCGCCGCCGTCGTCCG
query     CGCCGCCGTCGCCGCCGTCGTCCGGAGGCGTGACATTACCGCCATCGCCGCCGTCGTCCG

template  GAGGCGTGACATTACCGCCATCGTCACCGCCATCCGAAGGCGTGTCATCACCGCCGCTGT
query     GAGGCGTGACATTACCGCCATCGTCACCGCCATCCGAAGGCGTGTCATCACCGCCGCTGT

template  CGTCATCATCAGACTGTGCTTTTGACGCCAGATACCAGTCGTTGTCACTTTCATTCCAGT
query     CGTCATCATCAGACTGTGCTTTTGACGCCAGATACCAGTCGTTGTCACTTTCATTCCAGT

template  ACAGACGGTAGGTATACAGACCGGCGTTAACCTCATTTTTTAGCTCAAACGCATCGCTGG
query     ACAGACGGTAGGTATACAGACCGGCGTTAACCTCATTTTTTAGCTCAAACGCATCGCTGG

template  TCGATACGCCGCCAACATCCACCACTTCGATACCGTTAGTGGTCTGTGCTCCATCGCCGA
query     TCGATACGCCGCCAACATCCACCACTTCGATACCGTTAGTGGTCTGTGCTCCATCGCCGA

template  TGCCGTTGATGTACAGATCGGTGGTGCCGGAAGCATCGCCGGTAATGACCAACTTATCGG
query     TGCCGTTGATGTACAGATCGGTGGTGCCGGAAGCATCGCCGGTAATGACCAACTTATCGG

template  TTGCCGAATCATCGTCGCCCAGGACGGTATTCAGGTAAAGCGAGCCGCCGTTGCCGGTGT
query     TTGCCGAATCATCGTCGCCCAGGACGGTATTCAGGTAAAGCGAGCCGCCGTTGCCGGTGT

template  AATTGCCATCGACATAAAGCGTATTGCCAGGCGTGCTGGAGCTGCTACCACTGGTCATTG
query     AATTGCCATCGACATAAAGCGTATTGCCAGGCGTGCTGGAGCTGCTACCACTGGTCATTG

template  TCCCCATGTTGATCAGATCGCCATTGAAGGTGAAGATAGCGCCTGTTTCCTCGCTGTTGC
query     TCCCCATGTTGATCAGATCGCCATTGAGGGTGAAGATAGCGCCTGTTTCCTCGCTGTTGC

template  CGAAGACCAGCGTCCCTTCGTTGGTCACCTTACCGTTAACGGTCGTACCATTGCTGCCGC
query     CGAAGACCAGCGTCCCTTCGTTGGTCACCTTACCGTTAACGGTCGTACCATTGCTGCCGC

template  CGAAGGTCGCCCCGGAGGCGACGTTCACCGCCTGCTGGCTGCCCATCTCGCCAATCGAGC
query     CGAAGGTCGCCCCGGAGGCGACGTTCACCGCCTGCTGGCTGCCCATCTCGCCAATCGAGC

template  CGTCTCCGGACAGCCATAGCGTGCCTTCCTGTACGTTGGTATCGCCGCTATAAGTGTTCG
query     CGTCTCCGGACAGCCATAGCGTGCCTTCCTGTACGTTGGTATCGCCGCTATAGGTGTTCG

template  CGCCGCTCAGCGTCAGTTTACCCGCGCCCAGTTTGGTCAGCGATTTACCGTCCCAGTCGC
query     CGCCGCTCAGCGTCAGTTTACCCGCGCCCAGTTTGGTCAGCGATTTACCGTCCCAGTCGC

template  CGTTGCCGGTTTTATCGCCCAGTACGCTGGTGACTTCAAAACTGTCGCCTGCGCCCAGGG
query     CGTTGCCGGTTTTATCGCCCAGTACGCTGGTGACTTCAAAACTGTCGCCTGCGCCCAGGG

template  TAAACGTGCCGTGTGCTGGCGTCGCTGACGTGGCGGTCGCATTCCAGCTCAGCCCTTCGG
query     TAAACGTGCCGTGTGCTGGCGTCGCTGACGTGGCGGTCGCATTCCAGCTCAGCCCTTCGG

template  TAAGCAGATATTCTGACGCATCTTCAGGATTGATTGTCCCGGTTACGGTCAGATAGTCCG
query     TAAGCAGATATTCTGACGCATCTTCAGGATTGATTGTCCCGGTTACGGTCAGATAGTCCG

template  GCTTGTCAGTGAGGTTCATCGTCAGGCTGGTAAAGTCGCCGCTGATATCGCTGGTCATAT
query     GCTTGTCAGTGAGGTTCATCGTCAGGCTGGTAAAGTCGCCGCTGATATCGCTGGTCATAT

template  CCATCAGTTTAAAGGACTGGAACTCCGCGTCGGAGGCGCGCGCTTGCAGGCTGGCGTCGC
query     CCATCAGTTTAAAGGACTGGAACTCCGCGTCGGAGGCGCGCGCTTGCAGGCTGGCGTCGC

template  TGACGACCAAATCGCCGCCAAGCGTGGCGCTGCTGCCGGTAATCAGCGGTTGCACTGAGT
query     TGACGACCAAATCGCCGCCAAGCGTGGCGCTGCTGCCGGTAATCAGCGGTTGCACTGAGT

template  CACTATTCAGCTCAACGTTCAGCGTACTGCCATCTTGCTGAATTAACGCGCTGTCGCCGA
query     CACTATTCAGCTCAACGTTCAGCGTACTGCCATCTTGCTGAATTAACGCGCTGTCGCCGA

template  GATCCAACGAGGTGCCGAGCGCCAGTTGGGTCGTCGCGCCGCTATGAGTGGTGATGCCGC
query     GATCCAACGAGGTGCCGAGCGCCAGTTGGGTCGTCGCGCCGCTATGAGTGGTGATGCCGC

template  CCACCGCGCTGACTTCACCGTCTGCGTCCAGCACCAGCGTGGCATTATTGTCTACCAGAC
query     CCACCGCGCTGACTTCACCGTCTGCGTCCAGCACCAGCGTGGCATTATTGTCTACCAGAC

template  CGGTGCCCAGCGCCGTCGTACTGGCGGCGACCAGCGTACCTTCATTGATGGTGGTGCCGC
query     CGGTGCCCAGCGCCGTCGTACTGGCGGCGACCAGCGTACCTTCATTGATGGTGGTGCCGC

template  CGCTGTAGTCGTTGTCGCCAGAGAGGATCAGCGTGCCTGCGCCTTCTTTGGTCAGCGATT
```

```
query       CGCTGTAGTCGTTGTCGCCAGAGAGGATCAGCGTGCCTGCGCCTTCTTTGGTCAGCGATT

template    TACCGTCCCATCTGCTACCCGGATCCAGCGTATCGTCAACGTCTGTCAGTGTAGCCGCAA
query       TACCGTCCCATCTGCTACCCGGATCCAGCGTATCGTCAACGTCTGTCAGTGTAGCCGCAA

template    CGTTAAAGCTGCCGTCAGGGTCGCTCAGGGTAAAGGTGCCGTGGGCGTCGGTAGTGGCGT
query       CGTTAAAGCTGCCGTCAGGGTCGCTCAGGGTAAAGGTGCCGTGGGCGTCGGTAGTGGCGT

template    TGTCTCTGTCCGCATACCAGGAGAGGGAGGCCGTCAGATCATAGTGGGTGTTGTCTGCTT
query       TGTCTCTGTCCGCATACCAGGAGAGGGAGGCCGTCAGATCATAGTGGGTGTTGTCTGCTT

template    CATCGACTTTTCCGTCGATAGTCAGGAAGTCAACATCTTCTCTATTCATTCCCGCGACGG
query       CATCGACTTTTCCGTCGATAGTCAGGAAGTCAACATCTTCTCTATTCATTCCCGCGACGG

template    TCAGATCGTCGAAATCGGTCGTAATGGCGCTGTCGCTGCCAATCAGCGTGTATGTGTATG
query       TCAGATCGTCGAAATCGGTCGTAATGGCGCTGTCGCTGCCAATCAGCGTGTAGGTGTATG

template    CCTCAGGCGTCCAGCTGTCTGTAACGCTGCCAATGCCGGTAACATTCAGCGTGCCGCCCA
query       CCTCAGGCGTCCAGCTGTCTGTAACGCTGCCAATGCCGGTAACATTCAGCGTGCCGCCCA

template    GCGCGACGCTGTCGGCGGTAATCACGGCGTCGTCGGTGGCCGCGCCGAGGTCAATACTTA
query       GCGCGACGCTGTCGGCGGTAATCACGGCGTCGTCGGTGGCCGCGCCGAGGTCAATACTTA

template    GCGTACTGCCGTCTTCCTGGGTGAACTGGCCCGCGTCAAGGGTGCTGCCCGCTGCCAGCG
query       GCGTACTGCCGTCTTCCTGGGTGAACTGGCCCGCGTCAAGGGTGCTGCCCGCTGCCAGCG

template    CGGTAGTGGCGCCGCTATGGGTGGTGACGTTCGCAAGCTCAAATGCGCCGTTGGCATCCA
query       CGGTAGTGGCGCCGCTATGGGTGGTGACGTTCGCAAGCTCAAATGCGCCGTTGGCATCCA

template    GTATCAGCGTACCGCTGTTGTCGATATTGCCGCTGCCCAGCGCGTTGACGTTGGCGGCAA
query       GTATCAGCGTACCGCTGTTGTCGATATTGCCGCTGCCCAGCGCGTTGACGTTGGCGGCAA

template    TCAGCGTGCCGTCAGTGATAGTGGTGGCGCCGGAATAGCTGTTGTCGCCGCCTAACGTCA
query       TCAGCGTGCCGTCAGTGATAGTGGTGGCGCCGGAATAGCTGTTGTCGCCGCCTAACGTCA

template    GCTCGCCGGTACCGGTTTTGACCAGCGAACCGCTGCCGGAAAGCGTATTTTCCAGCTCGC
query       GCTCGCCGGTACCGGTTTTGACCAGCGAACCGCTGCCGGAAAGCGTATTTTCCAGCTCGC

template    CCTCACCGACCTGAAGCACACCGCTGTTGGCAACAGCTCCGGTACCCAGCGAATCCGCAT
query       CCTCACCGACCTGAAGCACACCGCTGTTGGCAACAGCTCCGGTACCCAGCGAATCCGCAT

template    GGTCAGCCGTCAGCGTACCGCCGATAATGGTGGTGCCGCCGGAGTAACTGTTATCGCCGC
query       GGTCAGCCGTCAGCGTACCGCCGATAATGGTGGTGCCGCCGGAGTAACTGTTATCGCCGC

template    TTAACGTCAGTTCGCCCGTACCGGTTTTGACCAGCGAACCGCTGCCGGAAAGCGTATTTT
query       TTAACGTCAGTTCGCCCGTACCGGTTTTGACCAGCGAACCGCTGCCGGAAAGCGTATTTT

template    CCAGCTCGCCCTCGCCGACCTGAAGCACACCGCTGTTGGCAACAGCTCCGGTACCCAGCG
query       CCAGCTCGCCCTCGCCGACCTGAAGCACACCGCTGTTGGCAACAGCTCCGGTACCCAGCG

template    AATCCGCATGGTCAGCCGTCAGCGTACCGCCGATAATGGTGGTGCCGCCGGAGTAACTGT
query       AATCCGCATGGTCAGCCGTCAGCGTACCGCCGATAATGGTGGTGCCGCCGGAGTAACTGT

template    TATCGCCGCTTAACGTCAGTTCGCCCGTACCGGTTTTGACCAGCGAACCGCTGCCGGAAA
query       TATCGCCGCTTAACGTCAGTTCGCCCGTACCGGTTTTGACCAGCGAACCGCTGCCGGAAA

template    GCGTATTTTCCAGCTCGCCCTCGCCGACCTGAAGCACGCCGCTGTTGGCAACAGCTCCGG
query       GCGTATTTTCCAGCTCGCCCTCGCCGACCTGAAGCACGCCGCTGTTGGCAACAGCTCCGG

template    TACCCAGCGAATCCGCATGGTCAGCCGTCAGCGTACCGCCGATAATGGTGGTGCCGCCGG
query       TACCCAGCGAATCCGCATGGTCAGCCGTCAGCGTACCGCCGATAATGGTGGTGCCGCCGG

template    AGTAATCGTTGCCGCCGCTTAACGTCAGCTCGCCGGTACCGGTTTTCACCAGCGAACCGC
query       AGTAATCGTTGCCGCCGCTTAACGTCAGCTCGCCGGTACCGGTTTTCACCAGCGAACCGC

template    TGCCGGAAAGCGTATTTTCCAGATCGCCTTCGCCCACCTTAAGCACGCCGCTGTTATCGA
query       TGCCGGAAAGCGTATTTTCCAGATCGCCTTCGCCCACCTTAAGCACGCCGCTGTTATCGA

template    TATCGCCGCTGCCCAGCGAATCCGCATGGTCAGCCGTCAGCGTACCGCCGGTAATGGTGG
query       TATCGCCGCTGCCCAGCGAATCCGCATGGTCAGCCGTCAGCGTACCGCCGGTAATGGTGG
```

```
template   TGCCGCCGGAGTAAGTGTTGTCGCCGCTTAGCGTCAGTTCGCCTGTGCCGGTTTTCACCA
query      TGCCGCCGGAGTAAGTGTTGTCGCCGCTTAGCGTCAGCTCGCCTGTGCCGGTTTTCACCA

template   GCGAGCCGCTGCCGGAAAGGATGTTTTCCAGCTCGCCTTCGCCCACCTTAAGCACGCCGC
query      GCGAGCCGCTGCCGGAAAGGATGTTTTCCAGCTCGCCTTCGCCCACCTTAAGCACGCCGC

template   TGTTGTCGATATCGCCGCTGCCCAGCGAATCCGCATGGTCAGCCGTCAGCGTACCGCCGG
query      TGTTGTCGATATCGCCGCTGCCCAGCGAATCCGCATGGTCAGCCGTCAGCGTACCGCCGG

template   AAATAGTGGTGCCGCCGCTGTAGTTGTTATCGCCGGTCACGATCAGCTCATCGCTACCGC
query      AAATAGTGGTGCCGCCGCTGTAGTTGTTATCGCCGGTCACGATCAGCTCATCGCTACCGC

template   TTTTGACAATTTGGCCCTCGCCGGTCACGTTGTTATCCAGCGTGCCCTGGCTCAGCGCAA
query      TTTTGACAATTTGGCCCTCGCCGGTCACGTTGTTATCCAGCGTGCCCTGGCTCAGCGCAA

template   GCGTTGCGCTTTCTGCAATCGTCACATCGCCAGTGCCGAGCGCGGCGGCGTCTTTGGCGG
query      GCGTTGCGCTTTCTGCAATCGTCACATCGCCAGTGCCGAGCGCGGCGGCGTCTTTGGCGG

template   CCAGAATGCCGCCGTCAATTTCAGTATCGCCGTAGTCGTTGCCGGTGTTGCTGAGGATCA
query      CCAGAATGCCGCCGTCAATTTCAGTATCGCCGTAGTCGTTGCCGGTGTTGCTGAGGATCA

template   GCGTGCCGTCGCCTTGTTTGGTTAGCTTGCTGCCGTTCCAGTTGGAGGTCGCCGTGGTTT
query      GCGTGCCGTCGCCTTGTTTGGTTAGCTTGCTGCCGTTCCAGTTGGAGGTCGCCGTGGTTT

template   CGTCCAGTTCGCTGGTGACGGTAAAGGTACTGCCTGCATCAACGGTAAAAGTGCCATGCG
query      CGTCCAGTTCGCTGGTGACGGTAAAGGTACTGCCTGCATCAACGGTAAAAGTGCCATGCG

template   CAGCGCGCGCAGAATTTGCGCCCGCGTACCAACTCAAGCCCGTAGACAACTCGTAGTTGG
query      CAGCGCGCGCAGAATTTGCGCCCGCGTACCAACTCAAGCCCGTAGACAACTCGTAGTTGG

template   TATTATCATTCGCATCAACGCCCGCGTTGATCGTCAGATAATCCGGCATAGCGGAGGTGT
query      TATTATCATTCGCATCAACGCCCGCGTTGATCGTCAGATAATCCGGCATAGCGGAGGTGT

template   CAGCATCCAGTGTTACGCTGTCAAAATCGGTAGTGATTGCAGTTTGCGAATCAATCAGCG
query      CAGCATCCAGTGTTACGCTGTCAAAATCGGTAGTGATTGCAGTTTGCGAATCAATCAGCG

template   TGATCGATCCAAAGTGTTCGTCGGTTTCGGGCGCGACAAAGGTGGCCGTTGAGCTAAGGT
query      TGATCGATCCAAAGTGTTCGTCGGTTTCGGGCGCGACAAAGGTGGCCGTTGAGCTAAGGT

template   TCAGCGTCCCGTCCAGCGTGACGTTGGCGGCTTTGATAATTGGCGCGCTGTTGGCGGTAT
query      TCAGCGTCCCGTCCAGCGTGACGTTGGCGGCTTTGATAATTGGCGCGCTGTTGGCGGTAT

template   CCAGCGTCGCGTTTATCTGGCTGCCGTCCTGCAAGGTCAGGGTATTGTCCACCGTCAGCA
query      CCAGCGTCGCGTTTATCTGGCTGCCGTCCTGCAAGGTCAGGGTATTGTCCACCGTCAGCA

template   GCGTACCTGTCGAACCGCTCGCGTTGCCCAGCGTAATATCGCCGTCGTTGGTCAGATTGC
query      GCGTACCTGTCGAACCGCTCGCGTTGCCCAGCGTAATATCGCCGTCGTTGGTCAGATTGC

template   CGACGGTTGACGGGTCGGTCATGTTCCACTGGCTGGTGTCGTCGAGCGACAGCGTAGTAA
query      CGACGGTTGACGGGTCGGTCATGTTCCACTGGCTGGTGTCGTCGAGCGACAGCGTAGTAA

template   ACATCGCATCTACACTACCGGTTAACGTGGACCCCTGGCTTAAAGAGAGCGATGACGATG
query      ACATCGCATCTACACTACCGGTTAACGTGGACCCCTGGCTTAAAGAGAGCGATGACGATG

template   ACGCGTCAGTCTGGACAATGTTGCCGGTCAGCATCGTTTTTTCGGCCTGAATAGTCGCTT
query      ACGCGTCAGTCTGGACAATGTTGCCGGTCAGCATCGTTTTTTCGGCCTGAATAGTCGCTT

template   TGCTGCCTAATTTGATCGCGGTCGGCGCCGTAAAGGTGCTGTTGGTGAGCTCAATGGGGT
query      TGCTGCCTAATTTGATCGCGGTCGGCGCCGTAAAGGTGCTGTTGGTGAGCTCAATGGGGT

template   CGTTAATGGTCGTGCTGCCATCAATGTTCAGTGCGACAGTTTTTGAGGTGACGTTTGCGT
query      CGTTAATGGTCGTGCTGCCATCAATGTTCAGTGCGACAGTTTTTGAGGTGACGTTTGCGT

template   CGCTGACCGTCAGCGCGTTGAGATCCTCATACCCGTCGAGTTTTCTTTTGCTGGTTATAA
query      CGCTGACCGTCAGCGCGTTGAGATCCTCATACCCGTCGAGTTTTCTTTTGCTGGTTATAA

template   AGATACCGTCGCCGTCGGCGTTGATGGTGGCGCCGTCGATCCTGGCGTCCGACGCCAGAA
query      AGATACCGTCGCCGTCGGCGTTGATGGTGGCGCCGTCGATCCTGGCGTCCGACGCCAGAA
```

```
template   TATACACGCCCGTACCGGCAGAGGTAATGTTGCCGCCTGTAATATCCAGGGTAGTGGCGT
query      TATACACGCCCGTACCGGCAGAGGTAATGTTGCCGCCTGTAATATCCAGGGTAGTGGCGT

template   TATTGGCCTGTACCGCCGCGCCGCTGGCATTGGTATGTGTTATGGTCGCGTTGGTGAGAT
query      TATTGGCCTGTACCGCCGCGCCGCTGGCATTGGTATGTGTTATGGTCGCGTTGGTGAGAT

template   TAAGAGCGGCCTTATTGGTACCCTGGATGGTGCTCATCGTACCTGCAGAGCTGACCGAGC
query      TAAGAGCGGCCTTATTGGTACCCTGGATGGTGCTCATCGTACCTGCAGAGCTGACCGAGC

template   TATCAGACAGGTTCAGGGTGGAACCCTCGTCTGTCGCGGTGTTTCCGGCAACAACGTTTA
query      TATCAGACAGGTTCAGGGTGGAACCCTCGTCTGTCGCGGTGTTTCCGGCAACAACGTTTA

template   TCTGGCCTTGCGCCAGGGTGATTGTGCTACCGTCCAGCACGTTCAGCGTACTGCCTTGTT
query      TCTGGCCTTGCGCCAGGGTGATTGTGCTACCGTCCAGCACGTTCAGCGTACTGCCTTGTT

template   GCACCTGCGCGCTGCTGCTGTTAGCTGCCTCAACGATGGTACCGTCCAGCGTTGCTTCTG
query      GCACCTGCGCGCTGCTGCTGTTAGCTGCCTCAACGATGGTACCGTCCAGCGTTGCTTCTG

template   AACCCGTATACAACGTCATTACGCCGAAATTGCCGTCAATCGTTAGCGTACTGTCTGTTA
query      AACCCGTATACAACGTCATTACGCCGAAATTGCCGTCAATCGTTAGCGTACTGTCTGTTA

template   ACCTCAGTGTGCTGCCATCCGCTGCGGAAATACCCTGGGTATAAATACCCGTAGTGGTTA
query      ACCTCAGTGTGCTGCCATCCGCTGCGGAAATACCCTGGGTATAAATACCCGTAGTGGTTA

template   TCTTCGCGTCGGTAAGCTCAAGGATAGCGTAATGATCCAGCGAAATGGCGTTGGAAACAT
query      TCTTCGCGTCGGTAAGCTCAAGGATAGCGTAATGATCCAGCGAAATGGCGTTGGAAACAT

template   ACTTACCGCCATCCGGATCATCAGCCCCGCCTCCGCTTGTTTCAATGACGAGCCCCGTCC
query      ACTTACCGCCATCCGGATCATCAGCCCCGCCTCCGCTTGTTTCAATGACGAGCCCCGTCC

template   CTGTGACCGTGGTGGGCTGCCAGCCGACGGTGGCGACACCCGAGACATATAGCGCCGCGC
query      CTGTGACCGTGGTGGGCTGCCAGCCGACGGTGGCGACACCCGAGACATATAGCGCCGCGC

template   CTTCATCGGTGCCCGAATAGCTTATCGGCGGCGTTGACGCATCCAGAACGTGGGGCTGCG
query      CTTCATCGGTGCCCGAATAGCTTATCGGCGGCGTTGACGCATCCAGAACGTGGGGCTGCG

template   CATCAGCATATGATACCTGATTATCGGCCATAGCCAGCGGGCTGAAAAATCCGAGCGCCG
query      CATCAGCATATGATACCTGATTATCGGCCATAGCCAGCGGGCTGAAAAATCCGAGCGCCG

template   CAGCCACGGCGCAAGCCAGCGCCGCGCGCGTTGCTTTTATTATTTGTCCATATTCGGACT
query      CAGCCACGGCGCAAGCCAGCGCCGCGCGCGTTGCTTTTATTATTTGTCCATATTCGGACT

template   TTTGGTTTGGAGATATGGGTTTCACTCGCGCATACTTGCGCTTTAACTTTGCTGTGGCCT
query      TTTGGTTTGGAGA-ATGGGTTTCACTCGCGCATACTTGCGCTTTAACTTTGCTGTGGCCT

template   CACGCCAGTTCTGAAGCCACGACATACATATTTCGCACGGCGCTCCAGACAATACTGTAA
query      CACGCCAGTTCTGAAGCCACGACATACATATTTCGCACGGCGCTCCAGACAATACTGTAA

template   GTCCTGTTCAATTTTTAAATTTCCCTTTAATAACGTTCACGAAGCAAACTGGATTTTGAT
query      GTCCTGTTCAATTTTTAAATTTCCCTTTAATAACGTTCACGAAGCAAACTGGATTTTGAT

template   TGGGCTGCAAATAGCCTTTAAATGCTACATTATCAATGAGTTAAATAATAAATAACAAAA
query      TGGGCTGCAAATAGCCTTTAAATGCTACATTATCAATGAGTTAAATAATAAATAACAAAA

template   ATACAGCTCTATGAATGGTTGCAATCACCTGAAACAATATATTATCCAAGCCCAAATTAT
query      ATACAGCTCTATGAATGGTTGCAATCACCTGAAACAATATATTATCCAAG-CCAAATTAT

template   TAACTAATTTCAAGGCTTATTTCAATGCCAGGTCAATTTCTGGTTGATTTATAATTGATA
query      TAACTAATTTCAAGGCTTATTTCAATGCCAGGTCAATTTCTGGTTGATTTATAATTGATA

template   TAAATACATTCATCCATTTAATAAATATTTATATCGTGAATAACATTTAATAACTTCATA
query      TAAATACATTCATCCATTTAATAAATATTTATATCGTGAATAACATTTAATAACTTCATA

template   TATTGAAATAAATAAATGCGTGAAACAAAAAGGTGTGATTATCATGCGACACCTGGCGTT
query      TATTGAAATAAATAAATGCGTGAAACAAAAAGGTGTGATTATCATGCGACACCTGGCGTT

template   ATTTTAAATATACTCGTCATACTTCAAGTTGCATGTGCTGCGGCTGCGTTCCCTCACCCC
query      ATTTTAAATATACTCGTCATACTTCAAGTTGCATGTGCTGCGGCTGCGTTCCCTCACCCC

template   AGTCACTTACTTTAGTAAGCTCCCGGGGATTCGCTCGCTTGCCGCCTTCCTGCAACTCGA
```

```
query     AGTCACTTACTTTAGTAAGCTCCCGGGGATTCGCTCGCTTGCCGCCTTCCTGCAACTCGA

template  ATTATTTAGAGTATATACGTAGCGCCAGATGCCGGGTTGCAGAA-GGAGACGGCGCTTTT
query     ATTATTTAGAGTATATACGTAGCGCCAGATGCCGGGTTGCAGAAGGGAGACGGCGCTTTT

template  ACACACGCGTGGCAAACGGTGGTGACGCCAATTAGCAGAACATATACTTTTCAATGAATG
query     ACACACGCGTGGCAAACGGTGGTGACGCCAATTAGCAGAACATATACTTTTCAATGAATG

template  CTACTTTCACTTTTTCATGAAGCTCAATTGAACGTGAAAAGCAGATTATTTTCGTGCCAG
query     CTACTTTCACTTTTTCATGAAGCTCAATTGAACGTGAAAAGCAGATTATTTTCGTGCCAG

template  ACGCATAATACGTTACTCCACGCACGTCGCATAACCCGCTGAGCCTGCCGGCTTAATGGC
query     ACGCATAATACGTTACTCCACGCACGTCGCATAACCCGCTGAGCCTGCCGGCTTAATGGC

template  GCAGTTTTGACTCTTGGTATTCTCATCTACGCCGCAATACATGCCGCCGCTTGAGGTTGA
query     GCAGTTTTGACTCTTGGTATTCTCATCTACGCCGCAATACATGCCGCCGCTTGAGGTTGA

template  TTTAGATAAATAGGGATAGCCCTGCGTCGGCCAGCCTAACGAGCTAATTGAATGATACAG
query     TTTAGATAAATAGGGATAGCCCTGCGTCGGCCAGCCTAACGAGCTAATTGAATGATACAG

template  CGTCAACAGGTCGTTATTATCCGGTGCTTTGCTAAGGCTACCGCACTGTTTGGCAATATC
query     CGTCAACAGGTCGTTATTATCCGGTGCTTTGCTAAGGCTACCGCACTGTTTGGCAATATC

template  CCATTTCGTGTTGTCCACCGTATGCGCCATCGCCCAGTACTCGTTATTTGCATGCACTCC
query     CCATTTCGTGTTGTCCACCGTATGCGCCATCGCCCAGTACTCGTTATTTGCATGCACTCC

template  GTTGGTAAATACTGGCGTTACGCCTGACAGCATCTCAGCCTGAAGCCACGGACGATGCAG
query     GTTGGTAAATACTGGCGTTACGCCTGACAGCATCTCAGCCTGAAGCCACGGACGATGCAG

template  CGTTTTTCCGTTGACGGATGTCGTATCCGACATATTTCCCCAGAACAACGCTTTATCCGT
query     CGTTTTTCCGTTGACGGATGTCGTATCCGACATATTTCCCCAGAACAACGCTTTATCCGT

template  ATCCGGGCTGGTAAGTACCATAAAAATCACATCCAGCGATGCGTGAAGAGTCGTATTGTC
query     ATCCGGGCTGGTAAGTACCATAAAAATCACATCCAGCGATGCGTGAAGAGTCGTATTGTC

template  CGTCACTTTCACCGTGAGTGGCGTTTTCAACCCCAGGCTTTTATCCTGATTAAGCGTAAA
query     CGTCACTTTCACCGTGAGTGGCGTTTTCAACCCCAGGCTTTTATCCTGATTAAGCGTAAA

template  CGTTGCCGTACCGTCGCTGCCGGTAGTGCCGGTAAAGACAATCCCGGTGGTGGTCATACT
query     CGTTGCCGTACCGTCGCTGCCGGTAGTGCCGGTAAAGACAATCCCGGTGGTGGTCATACT

template  TTGGCTGGCGCTTGCGGGAGTCAGCTCTTTAAGCATGAGATCGTCTGCTCCGGCATCCGC
query     TTGGCTGGCGCTTGCGGGAGTCAGCTCTTTAAGCATGAGATCGTCTGCTCCGGCATCCGC

template  TGCCACGTCGCCATCCGTTATCACCATACCTGCCCGATTTTTCGAATCGCCACGTGACAA
query     TGCCACGTCGCCATCCGTTATCACCATACCTGCCCGATTTTTCGAATCGCCACGTGACAA

template  CGTAAAACCGACGTTGGCGACCGGGTTACCGCTGCTATCTTTTACCGTCACCGTTAACGG
query     CGTAAAACCGACGTTGGCGACCGGGTTACCGCTGCTATCTTTTACCGTCACCGTTAACGG

template  CATCGCGCTTCCCTTCGCCACTACCGCCGCATTTTTCGCGCTGTCCATCGCCGTCGAGGT
query     CATCGCGCTTCCCTTCGCCACTACCGCCGCATTTTTCGCGCTGTCCATCGCCGTCGAGGT

template  CAGCGTAATACTGGCAGGAGTAGAAGTACGCGGCTCGACCAGACACACCTGAGCGCCGGT
query     CAGCGTAATACTGGCAGGAGTAGAAGTACGCGGCTCGACCAGACACACCTGAGCGCCGGT

template  GGCAGTCGATGAACTGGCGGTGCCCTTACCGCCGCTATTCAGGTTTATAAATTGGTAATA
query     GGCAGTCGATGAACTGGCGGTGCCCTTACCGCCGCTATTCAGGTTTATAAATTGGTAATA

template  TCCGGTATTCGGCGCACGATCCGCCGCCCACCAGGATTTGTTCCCCGCCACCGGCCAGCC
query     TCCGGTATTCGGCGCACGATCCGCCGCCCACCAGGATTTGTTCCCCGCCACCGGCCAGCC

template  GTAATTTGTACCGATTGCGCCGTTAGGATTGTCGCTATACAACGTTTGCAGATCGCCCGA
query     GTAATTTGTACCGATTGCGCCGTTAGGATTGTCGCTATACAACGTTTGCAGATCGCCCGA

template  CAACGGCTGGTATTCCGCATCACAGCCGGTTGCGCCCGCTTTTTCAGTGTTCGCTGCGGT
query     CAACGGCTGGTATTCCGCATCACAGCCGGTTGCGCCCGCTTTTTCAGTGTTCGCTGCGGT

template  GACTAAGGGCCATGTTTCATTGTTAACCAGGTAAGTACCGCTATTCGACGTCATCTCCGC
query     GACTAAGGGCCATGTTTCATTGTTAACCAGGTAAGTACCGCTATTCGACGTCATCTCCGC
```

```
template  CGCCAGCAGCGGACGCCGGAACTTCACGCCCGCGCTGTTGGTTACGGTTTCCGGCATGTG
query     CGCCAGCAGCGGACGCCGGAACTTCACGCCCGCGCTGTTGGTTACGGTTTCCGGCATGTG

template  TCCCCAGTATTTAGCTTTATCACTGTCCGGGCTGGTAATGACGGTGAAAATGACGTCCAT
query     TCCCCAGTATTTAGCTTTATCACTGTCCGGGCTGGTAATGACGGTGAAAATGACGTCCAT

template  ATCGCTAACTGTCAGCGGATCGTCGACAAGCATCGCCTGTAGCGGTGTTCTCAGGCCACG
query     ATCGCTAACTGTCAGCGGATCGTCGACAAGCATCGCCTGTAGCGGTGTTCTCAGGCCACG

template  GGAATCATCCTGCGTCACTTCGAGTTGCGCTTTACCATCCTCATCCGTAATGCCATACCA
query     GGAATCATCCTGCGTCACTTCGAGTTGCGCTTTACCATCCTCATCCGTAATGCCATACCA

template  ATAACTTGTGCACGAAGATACCGTGCAGTTAAACGTCATTGAGCCCGTAGACGGCGCAAC
query     ATAACTTGTGCACGAAGATACCGTGCAGTTAAACGTCATTGAGCCCGTAGACGGCGCAAC

template  CGATGTGAGTACC-TGCCTCCGCCGGAACTGCCATCAACGACACCCGCCCGGTCTATCGA
query     CGATGTGAGTACCATGCCTCCGCCGGAACTGCCATCAACGACACCCGCCCGGTCTATCGA

template  AAGCGTTCGTCCCAAACGGATAGCGGTATTGGGCTGCGGCGTGCCGGAACCATCCCTCAC
query     AAGCGTTCGTCCCAAACGGATAGCGGTATTGGGCTGCGGCGTGCCGGAACCATCCCTCAC

template  AGTGACGGTCAACGGCAGGCTCTCGCCCTTCTTCGCCACTGGAGCGCCTTTATCCGCATC
query     AGTGACGGTCAACGGCAGGCTCTCGCCCTTCTTCGCCACTGGAGCGCCTTTATCCGCATC

template  CATGTCGCTGGAAGTGAGTTCTATCGACAAGTCCCGGCGCTTTGCCAGACAAAGCTGCGC
query     CATGTCGCTGGAGGTGAGTTCTATCGACAAGTCCCGGCGCTTTGCCAGACAAAGCTGCGC

template  CGTGGCGGTATTGGCTTTGGTTGTCAGCGTATTATTATCGCTGAGTCTGACATACTGAAA
query     CGTGGCGGTATTGGCTTTGGTTGTCAGCGTATTATTATCGCTGAGTCTGACATACTGAAA

template  CTGACTGTCTGAGTGCGTGGCATCCGGTACTGAGTTACCCGCCCCCCAATATTTTCCTGA
query     CTGACTGTCTGAGTGCGTGGCATCCGGTACTGAGTTACCCGCCCCCCAATATTTTCCTGA

template  TGCCACCGGTAGGCCAAGCGTTGTCGTGAGTGCGCCATTGGGATAATCGGTATATAGCGT
query     TGCCACCGGTAGGCCAAGCGTTGTCGTGAGTGCGCCATTGGGATAATCGGTATATAGCGT

template  CTGTAAATCATTTACCGTCGGCAACCCCAGCCGATCGCACGGGCTGGCGGTGTCCTGATA
query     CTGTAAATCATTTACCGTCGGCAACCCCAGCCGATCGCACGGGCTGGCGGTGTCCTGATA

template  CATATTCGGATAACGGCTCCAGGTATACCAGGTCTCGTTATTTTCCGTATAACCCGACGT
query     CATATTCGGATAACGGCTCCAGGTATACCAGGTCTCGTTATTTTCCGTATAACCCGACGT

template  GTCCGCCGTTGAAGAAAGTTCAGCGCGCAGCAGCGGTCGCTTAAATTCAACCCCCTTACT
query     GTCCGCCGTTGAAGAAAGTTCAGCGCGCAGCAGCGGTCGCTTAAATTCAACCCCCTTACT

template  GTTGGTAAAGGTTTCCGGCATATGGCCCCAGTATTTGGCCTTATCGCTGTCCGGGCTGGT
query     GTTGGTAAAGGTTTCCGGCATATGGCCCCAGTATTTGGCCTTATCGCTGTCCGGGCTGGT

template  CACTACGGTAAAGATTGCGTCTTTGCTGTCTGTCAGGTTGCTGCCGTCCATCGTGGCGGC
query     CACTACGGTAAAGATTGCGTCTTTGCTGTCTGTCAGGTTGCTGCCGTCCATCGTGGCGGC

template  GCTGATAGTGGTTTTTAAACCGGCGGTACTGTCCTGCGCAAGGGTAAATGTCACCTGTCC
query     GCTGATAGTGGTTTTTAAACCGGCGGTACTGTCCTGCGCAAGGGTAAATGTCACCTGTCC

template  CTGGGCATCAGTTTGCGCATACAGATATTTCGCCGAGGTATCCAGCAGGTATGTCGCCAG
query     CTGGGCATCAGTTTGCGCATACAGATATTTCGCCGAGGTATCCAGCAGGTATGTCGCCAG

template  GCCGGACGGCATCACATTACGCAGCGTAATATCGTCTGTGATGCTTGAGGACGATGTGGC
query     GCCGGACGGCATCACATTACGCAGCGTAATATCGTCTGTGATGCTTGAGGACGATGTGGC

template  GCTGTTGGCGCGATTGTACGAAGATCCGCGGCTGATTTTCACCATCACGTTTTTCATCGG
query     GCTGTTGGCGCGATTGTACGAAGATCCGCGGCTGATTTTCACCATCACGTTTTTCATCGG

template  CTGACCTGCGGCGTCTTTCACGTCGATGGTGGCGGCCATCTGCTCGCCCTTCTTCGCCTT
query     CTGACCTGCGGCGTCTTTCACGTCGATGGTGGCGGCCATCTGCTCGCCCTTCTTCGCCTT

template  TGCGCTTCCGGTGGTTTCATCCTGGCCTGCCAGCGTCAGCGTCAGGCTGGCGGCGGCGCT
query     TGCGCTTCCGGTGGTTTCATCCTGGCCTGCCAGCGTCAGCGTCAGGCTGGCGGCGGCGCT
```

```
template    GCGCGGCGAGGCAAGACAGGTCTGCATGTAGTACATCGAGGTGCTGGTCGTCGTTTTGAC
query       GCGCGGCGAGGCAAGACAGGTCTGCATGTAGTACATCGAGGTGCTGGTCGTCGTTTTGAC

template    ATCATTGCCGTCGTTAAACAGGTTGACCGCCGCATAGAGCGACTTCGTGCCGTCTGACGA
query       ATCATTGCCGTCGTTAAACAGGTTGACCGCCGCATAGAGCGACTTCGTGCCGTCTGACGA

template    TATCACGTAATCCCCCGCCCACCAGTAGCGGCTGACGTTGTTGTTGCCTGAACTGGTCAT
query       TATCACGTAATCCCCCGCCCACCAGTAGCGGCTGACGTTGTTGTTGCCTGAACTGGTCAT

template    CGGCCAGCCGTTACGGCTGCGCATGGCGTTATCCGGGTATGTCCCGTACAGTGTCTGGAG
query       CGGCCAGCCGTTACGGCTGCGCATGGCGTTATCCGGGTATGTCCCGTACAGTGTCTGGAG

template    GTTGGCTACGGTCGGTAGCGTGTCGCGCCCACAGCCGCCACCGGTGCCGTTCCAGGCTTT
query       GTTGGCTACGGTCGGTAGCGTGTCGCGCCCACAGCCGCCACCGGTGCCGTTCCAGGCTTT

template    ATCGACGGTTATCATGCCCCAGGTTTCGCCTTTGTCGTAGTAGCCGTTGGCGATGGTCAG
query       ATCGACGGTTATCATGCCCCAGGTTTCGCCTTTGTCGTAGTAGCCGTTGGCGATGGTCAG

template    CGTTTTGCCGGGCGTAGCGCTAAACTCGCTGGCCAGCAGCGGACGCCTGTACGCCACGCC
query       CGTTTTGCCGGGCGTAGCGCTAAACTCGCTGGCCAGCAGCGGACGCCTGTACGCCACGCC

template    GCTGCTGTCGGTCAGCGTTTCCGCCATATGGCCCCAGTAGTTGGCCTTCGGCGTGTCCGG
query       GCTGCTGTCGGTCAGCGTTTCCGCCATATGGCCCCAGTAGTTGGCCTTCGGCGTGTCCGG

template    ACTGGTGACCACGGTAAAGATGACCGGCAGCGGCGCGCTCGTTACGCCGTTGTGTTCTAT
query       ACTGGTGACCACGGTAAAGATGACCGGCAGCGGCGCGCTCGTTACGCCGTTGTGTTCTAT

template    CGCCGCCGTCAGCGGCGTTTTAAAGCCCGCGCCGCTGTCCTGGCTGATATTCAGCGTCAG
query       CGCCGCCGTCAGCGGCGTTTTAAAGCCCGCGCCGCTGTCCTGGCTGATATTCAGCGTCAG

template    TGTGCCGTCGGCGCCCGTGGCGGTGTAATAGTTAGCGCCGCTGCTACGCCCGTCGCTGTG
query       TGTGCCGTCGGCGCCCGTGGCGGTGTAATAGTTAGCGCCGCTGCTACGCCCGTCGCTGTG

template    GTTTATCTGTATATCCGCCGCCGCCGAGCTATTCACCTCCTGATTCGCGCGCCCGACGCT
query       GTTTATCTGTATATCCGCCGCCGCCGAGCTATTCACCTCCTGATTCGCGCGCCCGACGCT

template    GTCGCCGCGCGTAAAGATAACCGGCGTGTCGGCGGCCGGGTTGCCCTGGGCATCCCGCGT
query       GTCGCCGCGCGTAAAGATAACCGGCGTGTCGGCGGCCGGGTTGCCCTGGGCATCCCGCGT

template    GGTGATGATGACCGGGATGGTCTCGCCGACTTTCGCTTTGGCGAATCCGTCGCTGTCGTT
query       GGTGATGATGACCGGGATGGTCTCGCCGACTTTCGCTTTGGCGAATCCGTCGCTGTCGTT

template    ATACACAGGCGAAGTGAGCGTTATCTGCGTCGCCGCCGGATGGGCGTTTTGCAGGCAGAC
query       ATACACAGGCGAAGTGAGCGTTATCTGCGTCGCCGCCGGATGGGCGTTTTGCAGGCAGAC

template    AAAATAGAGTTGTTCATTCCACAATAGCGAACCGACGCCGCCGTCGCGCAGATTAATCGA
query       AAAATAGAGTTGTTCATTCCACAATAGCGAACCGACGCCGCCGTCGCGCAGATTAATCGA

template    CTTAACGTAGCGGTTTTGCGTACTCCGGCTCAAATCGGCGGTGCTGTCCTGGTAGTTTTT
query       CTTAACGTAGCGGTTTTGCGTACTCCGGCTCAAATCGGCGGTGCTGTCCTGGTAGTTTTT

template    ACCGTCCAGCGGCCAGCCCTGCTGCGTATTCATGGCGCCGCCGGGCCTGGCGTCGTACAG
query       ACCGTCCAGCGGCCAGCCCTGCTGCGTATTCATGGCGCCGCCGGGCCTGGCGTCGTACAG

template    CGATTGCATATCGGCAAGGGTCGGCACGTATCCCGCGCCGCAGGCGTCGTTCGCCATTTC
query       CGATTGCATATCGGCAAGGGTCGGCACGTATCCCGCGCCGCAGGCGTCGTTCGCCATTTC

template    AAAATCGACGATTCCCCACGCTTCGTTGTTTTCCGTATTGTCGCCGTGGGTAACGCCGCT
query       AAAATCGACGATTCCCCACGCTTCGTTGTTTTCCGTATTGTCGCCGTGGGTAACGCCGCT

template    GGCGATTTCAGCCGATAACAGCGGGCGCTTAAACACCTCGCCGTTGGCCGCCGTCGTCGT
query       GGCGATTTCAGCCGATAACAGCGGGCGCTTAAACACCTCGCCGTTGGCCGCCGTCGTCGT

template    TTCCGGCATATGGCCCCACATTTTGGCTTTGTCGCTGTCCGGGCTGGTGACCACGGTGAA
query       TTCCGGCATATGGCCCCACATTTTGGCTTTGTCGCTGTCCGGGCTGGTGACCACGGTGAA

template    AATAGTGTCGATGCTGGCCGTCGCGCTGGCGTTGTCGTACAGCGCCGCCGTAATCGCGAC
query       AATAGTGTCGATGCTGGCCGTCGCGCTGGCGTTGTCGTACAGCGCCGCCGTAATCGCGAC

template    TTTCGTCCCGTGGGTGTCCGGGCGGGTGACGTTGATGATTTTGCTGCCGTTTTCACCGGT
```

```
query       TTTCGTCCCGTGGGTGTCCGGGCGGGTGACGTTGATGATTTTGCTGCCGTTTTCACCGGT

template    CATGCCGCCGATTTTGGTGGCGGTGTCGTTGAGCGAATCGCCGTCGATCACCACCGCCGA
query       CATGCCGCCGATTTTGGTGGCGGTGTCGTTGAGCGAATCGCCGTCGATCACCACCGCCGA

template    GACGATGCCGTCGCCGCTACCGGCGATATGTTTCTCCCCCTGGCGGGTATAGCCGTCGCC
query       GACGATGCCGTCGCCGCTACCGGCGATATGTTTCTCCCCCTGGCGGGTATAGCCGTCGCC

template    GCGGCTTAACACGAACGGCGCTTGCGGCAGCGGATTGCCGCTGGCGTCTTTTACCGTCAC
query       GCGGCTTAACACGAACGGCGCTTGCGGCAGCGGATTGCCGCTGGCGTCTTTTACCGTCAC

template    TTTAAGCTGGAGCGTATCGCCCTTTTTCACCTTCACCGCGTGTTCGCTGTTTGCGTTATA
query       TTTAAGCTGGAGCGTATCGCCCTTTTTCACCTTCACCGCGTGTTCGCTGTTTGCGTTATA

template    CCACAATGCCGCATTCACCGGCTCAATGGTGATAGACGCCGCCGTCGGATTTTTGCTGAC
query       CCACAATGCCGCATCCACCGGCTCAATGGTGATAGACGCCGCCGTCGGATTTTTGCTGAC

template    CAGACAGCTCACGTAGTCCGACGCATCGCCGGAAGCGGTATAGCCGCCCGCAGCCAGTGA
query       CAGACAGCTCACGTAGTCCGACGCATCGCCGGAAGCGGTATAGCCGCCCGCAGCCAGTGA

template    TACCGCCTGCCATGTAGCGGCGCTGGCGAAGGTGGATGACCAGTAGTCATACTCAGTCGG
query       TACCGCCTGCCATGTAGCGGCGCTGGCGAAGGTGGATGACCAGTAGTCATACTCAGTCGG

template    CCAGCCGGATACGGTGTGGATTTTATTGCCGTCGCTGGAGGCATACAGTGCGGCAAGCTG
query       CCAGCCGGATACGGTGTGGATTTTATTGCCGTCGCTGGAGGCATACAGTGCGGCAAGCTG

template    ATCGGCGCGCGGCAGATGGCCGGCCTCGCAGCCCCCGGCGTTGGGGTTATTCAGCGCGTC
query       ATCGGCGCGCGGCAGATGGCCGGCCTCGCAGCCCCCGGCGTTGGGGTTATTCAGCGCGTC

template    GGCGTGGCTGACACGCGCCCAGGTTTCGTTATCCTGCTCCTGGGTACGGGTGGCGGCAGT
query       GGCGTGGCTGACACGCGCCCAGGTTTCGTTATCCTGCTCCTGGGTACGGGTGGCGGCAGT

template    CGCTTCCGCCGCCAGCTTTGGCCGCTGGAATTTCATATCACCCACGGTGAGCGTGTCCGC
query       CGCTTCCGCCGCCAGCTTTGGCCGCTGGAATTTCATATCACCCACGGTGAGCGTGTCCGC

template    CATATGACCCCACATCTGCGCCTCCGGCGTATCGGGGCTGGTGGCAACGGTAAATTTCAC
query       CATATGACCCCACATCTGCGCCTCCGGCGTATCGGGGCTGGTGGCAACGGTAAATTTCAC

template    GCTGCGGTTGACGGGCGTGGTAATCAGTGAGTTGACCGGCAGTACGCTCAGCGGCGTCAG
query       GCTGCGGTTGACGGGCGTGGTAATCAGTGAGTTGACCGGCAGTACGCTCAGCGGCGTCAG

template    CAAGCCCACGCCCTGCGGCTGTTCAATCACCAGCGTGGCGTTGCCCTCGGCATCGGTCAT
query       CAAGCCCACGCCCTGCGGCTGTTCAATCACCAGCGTGGCGTTGCCCTCGGCATCGGTCAT

template    GCCCTGGTACGTCATCGACGCCTGGGAAGAGCCGAAAGGCGTTCCGCCCATCACCAGCGT
query       GCCCTGGTACGTCATCGACGCCTGGGAAGAGCCGAAAGGCGTTCCGCCCATCACCAGCGT

template    TCCGTCGCTGGGATCGGTAAAGCCCGTGGTGGCGTTGTCGCGATTTTTGCCGTGCGACAT
query       TCCGTCGCTGGGATCGGTAAAGCCCGTGGTGGCGTTGTCGCGATTTTTGCCGTGCGACAT

template    GGTGACGGTAAAGCTGGCGTTAGGCACGCTTAACCCATTCAGCGCATTGACGGAATGGAC
query       GGTGACGGTAAAGCTGGCGTTAGGCACGCTTAACCCATTCAGCGCATTGACGGAATGGAC

template    GTTCATCGTGATTTGTTCGCCGACTTTGGCTACCGCATTGCGTAACGACTCATCGCCATT
query       GTTCATCGTGATTTGTTCGCCGACTTTGGCTACCGCATTGCGTAACGACTCATCGCCATT

template    AAAGTAAACGTCCAGACTGGTCACCAACTCATTGCCGGAGCAGGAGAGATAGTTCGGTTC
query       AAAGTAAACGTCCAGACTGGTCACCAACTCATTGCCGGAGCAGGAGAGATAGTTCGGTTC

template    GGTGCCGCTGAACGTGCTGTCCGCACCGGTCGCCAGGTTGACGTGCGCGGTGGTGGAACC
query       GGTGCCGCTGAACGTGCTGTCCGCACCGGTCGCCAGGTTGACGTGCGCGGTGGTGGAACC

template    ATCAGTATCCGCCGCGATATAGCTGTGTTTCTGCGTCGGCCAGCCGTAGGTCGTTTCCAT
query       ATCAGTATCCGCCGCGATATAGCTGTGTTTCTGCGTCGGCCAGCCGTAGGTCGTTTCCAT

template    CGCGTTGCCGGAATGGGCCTGATACAGCGTATCCAGCAACAGTTGCCCCGGCACCTGGCC
query       CGCGTTGCCGGAATGGGCCTGATACAGCGTATCCAGCAACAGTTGCCCCGGCACCTGGCC

template    GGTACCGCACTGGTTCGTCGCCGCCGTTACCGAGTTAAACGTCGCCCAGTATTCGTTATT
query       GGTACCGCACTGGTTCGTCGCCGCCGTTACCGAGTTAAACGTCGCCCAGTATTCGTTATT
```

```
template  CTCAAATTCGGAACCGGTATCCTGCGCGGCTTCGTCCGCCAGCAGCGGGCGTTTGTAGAT
query     CTCAAATTCGGAACCGGTATCCTGCGCGGCTTCGTCCGCCAGCAGCGGGCGTTTGTAGAT

template  ATTGCCCGCTTCCACCACGCCCTGCATATGGCCCCACATGCGCGCCTGCGCCACGTCCGG
query     ATTGCCCGCTTCCACCACGCCCTGCATATGGCCCCACATGCGCGCCTGCGCCACGTCCGG

template  GCTGGTGACGACGGTAAAAATCACGTTCTGCGTCGCTTCCGTGCCGTCGAACAGCACCAC
query     GCTGGTGACGACGGTAAAAATCACGTTCTGCGTCGCTTCCGTGCCGTCGAACAGCACCAC

template  GCGCACCGGGGTTAATACGCCCGCCCCCTTGTCCTGAGTCAACACCAGCGAGAACTGACC
query     GCGCACCGGGGTTAATACGCCCGCCCCCTTGTCCTGAGTCAACACCAGCGAGAACTGACC

template  GTTGGCGTCGGTGACGCCGTGATAGTGACCGTCGCTGCCTTCCAGATTGGTGATAATTAC
query     GTTGGCGTCGGTGACGCCGTGATAGTGACCGTCGCTGCCTTCCAGATTGGTGATAATTAC

template  CGGATCATCCGTCCACTGATGCCCGCTATCAATATCGGCGTTCTTTTGATTCTGGCGATT
query     CGGATCATCCGTCCACTGATGCCCGCTATCAATATCGGCGTTCTTTTGATTCTGGCGATT

template  TTGCTCGTCGCCCAGGTAGACGTTAAAGTAGCGATAAGGCAGCGGTTTATTGGTGGCGCT
query     TTGCTCGTCGCCCAGGTAGACGTTAAAGTAGCGATAAGGCAGCGGTTTATTGGTGGCGCT

template  GTCGGTGACGGTGATCTTCATGTTGATCTCTTCGCCCACCTTCACTTTCGCCGCGTTGAC
query     GTCGGTGACGGTGATCTTCATGTTGATCTCTTCGCCCACCTTCACTTTCGCCGCGTTGAC

template  CGTTGAATCAAAATTATCGGCGCTAATCACTAATTTCGGCGTGACCGCAGGCGGGGCTTT
query     CGTTGAATCAAAATTATCGGCGCTAATCACTAATTTCGGCGTGACCGCAGGCGGGGCTTT

template  ATCCACGCAGCTAACCAGCGATTGCGTGGCGTCCGGCATCTGCGTAAGGGCGCGGCTGCG
query     ATCCACGCAGCTAACCAGCGATTGCGTGGCGTCCGGCATCTGCGTAAGGGCGCGGCTGCG

template  CATATCAATGCTGATATGGTCAGTGGCAGTCGATCCCTGGGAGGAGGACCAGTACTCTTC
query     CATATCAATGCTGATATGGTCAGTGGCAGTCGATCCCTGGGAGGAGGACCAGTACTCTTC

template  GTCACCGGATGTCAGCCCTACCGGCCAGCCGAGGGTGGTGGCCAGATCGCCGCGCGCAAT
query     GTCACCGGATGTCAGCCCTACCGGCCAGCCGAGGGTGGTGGCCAGATCGCCGCGCGCAAT

template  TTTTAACCCTTCCAGTTGGCGTGCATCCGGCAGAATATCGCAGTGCGCGTCGGCGTGGCC
query     TTTTAACCCTTCCAGTTGGCGTGCATCCGGCAGAATATCGCAGTGCGCGTCGGCGTGGCC

template  CCAGTTGAACTGCGCCCAGGTTTCATTGTTGATGCTGGCGGTGCGGTCTTCACTGTCGGT
query     CCAGTTGAACTGCGCCCAGGTTTCATTGTTGATGCTGGCGGTGCGGTCTTCACTGTCGGT

template  TTCCGCCGCCAGTTTGGGGCGGTAAAAGGTGTAGCTTGCGCCGTCCACCGTGGCGGTGGA
query     TTCCGCCGCCAGTTTGGGGCGGTAAAAGGTGTAGCTTGCGCCGTCCACCGTGGCGGTGGA

template  CGTCTCTCCCATATGACCGTACATGTTGGCGCTGCTGCTGTCCGGGCTGGTGAGGGTGGT
query     CGTCTCTGCCATATGACCGTACATGTTGGCGCTGCTGCTGTCCGGGCTGGTGAGGGTGGT

template  AAAAATCACGTCCACGCTGGCTTTCAGCGCTGGCGCGCTCGACGGGTGCGCTATCAGCGG
query     AAAAATCACGTCCACGCTGGCTTTCAGCGCTGGCGCGCTCGACGGGTGCGCTATCAGCGG

template  CGTTTTAACGCCCGGGCCGTTCGCCTGGGTGACGACCACGGTCGCCACGCCCTCGGCGTT
query     CGTTTTAACGCCCGGGCCGTTCGCCTGGGTGACGACCACGGTCGCCACGCCCTCGGCGTT

template  GGTCGTACCGTGATATTCCGTGGCGGTGGTGGTCAGTTCGGTGTTGCCGACGCGCACCGG
query     GGTCGTACCGTGATATTCCGTGGCGGTGGTGGTCAGTTCGGTGTTGCCGACGCGCACCGG

template  CGCGGCATTGTTTACCACGCCCTGGCGGTTTTGCGCGTCGCCGCGGGTAATCACGAAATC
query     CGCGGCATTGTTTACCACGCCCTGGCGGTTTTGCGCGTCGCCGCGGGTAATCACGAAATC

template  AACGTTGCCCTCCGGTTCGCCGCCGCAGTCTTTGGTCGTTACCGTCAGCATGATGCTTTC
query     AACGTTGCCCTCCGGTTCGCCGCCGCAGTCTTTGGTCGTTACCGTCAGCATGATGCTTTC

template  GCCCGCTTTGACCTTCGCGGCGTTGAGCGACTCATCCAGATGATCGGCGGCGAGCGTTAC
query     GCCCGCTTTGACCTTCGCGGCGTTGAGCGACTCATCCAGATGATCGGCGGCGAGCGTTAC

template  CGGGTCGCCCACGGCGGGCGCGATATCCACGGCAAACTTTTGATCAAGCACTTCGACCGC
query     CGGGTCGCCCACGGCGGGCGCGATATCCACGGCAAACTTTTGATCAAGCACTTCGACCGC
```

```
template   CGTGTTATAAGGCACGCCGGTGCGGGTAAGCAACCACAGCGGCGGTATGGCGGTTGAGGT
query      CGTGTTATAAGGCACGCCGGTGCGGGTAAGCAACCACAGCGGCGGTATGGCGGTTGAGGT

template   ATCCAGTTGCGACCAGACCACCAGATGGCGGCCCGCCAGATTCTGCGCGCAGAAACTTAT
query      ATCCAGTTGCGACCAGACCACCAGATGGCGGCCCGCCAGATTCTGCGCGCAGAAACTTAT

template   ATTGCTGACGGGTTGTGGATCAAGCGGCGTGTCCGGCGTGGCGGCGTCGGCCCACACCAG
query      ATTGCTGACGGGTTGTGGATCAAGCGGCGTGTCCGGCGTGGCGGCGTCGGCCCACACCAG

template   CGTAACGGCGGGCGCGCGTTCAGCCTGCCAGGCCAGCGGCGGCACGGCGAAGATATCGCC
query      CGTAACGGCGGGCGCGCGTTCAGCCTGCCAGGCCAGCGGCGGCACGGCGAAGATATCGCC

template   TTCGGTGTCGTGCGGATTGGTTAAGGTCAGCGCGCTGCCGACATCACTGGTGCTGAACTG
query      TTCGGTGTCGTGCGGATTGGTTAAGGTCAGCGCGCTGCCGACATCACTGGTGCTGAACTG

template   GCTGGGCTTCGCGGAGTAGTCAACAGGATTCGCCGCATCAGAGCGCAACTGCACGCTGCC
query      GCTGGGCTTCGCGGAGTAGTCAACAGGATTCGCCGCATCAGAGCGCAACTGCACGCTGCC

template   CTGATATACCGGGATCGTTACACTGTCGGCCTGAGGCGGAGTGCCATTAATAGCCGCAGT
query      CTGATATACCGGGATCGTTACACTGTCGGCCTGAGGCGGAGTGCCATTAATAGCCGCAGT

template   TTGCTCAGTGAGTTCCTGCCATGCGCCGCTTCTTCGCACGGCCTGCGCGAAGAAAGGCGT
query      TTGCTCAGTGAGTTCCTGCCATGCGCCGCTGCTTCGCACGGCCTGCGCGAAGAAAGGCGT

template   TAAACTGAGGGCGCATAGCAGCAGCCAGGGTAAAAAACCGACACACTTTTTCGATTTCAC
query      TAAACTGAGGGCGCATAGCAGCAGCCAGGGTAAAAAACCGACACACTTTTTCGATTTCAC

template   TGACGTACCTGAGAAAAGTCCATTTCGTTGCAGTTCATTCCATGAATACAAAAACGTAAA
query      TGACGTACCTGAGAAAAGTCCATTTCGTTGCAGTTCATTCCATGAATACAAAAACGTAAA

template   AATACATCGCTACCGGAACAGCGCCAGCTACAAGGGATACCCAGGTTGGGCGCCGCAAAG
query      AATACATCGCTACCGGAACAGCGCCAGCTACAAGGGATACCCAGGTTGGGCGCCGCAAAG

template   CGTCACCCAACATTCAAACGCGCTCCCGGCCCCATTCCCCATTCACTACGGCACCCGGCA
query      CGTCACCCAACATTCAAACGCGCTCCCGGCCCCATTCCCCATTCACTACGGCACCCGGCA

template   CGAGCCTAAGCCCGAGGTCGTCGCTTTTTCACGAGTACAGGTTGTCTGGCCGGTCGTTTC
query      CGAGCCTAAGCCCGAGGTCGTCGCTTTTTCACGAGTACAGGTTGTCTGGCCGGTCGTTTC

template   GTTAAATGAACAGTAGTACTTATTGCTTTGACCTTCATCTTGCGACACATAGTCCCAGGA
query      GTTAAATGAACAGTAGTACTTATTGCTTTGACCTTCATCTTGCGACACATAGTCCCAGGA

template   TGATGTCGTCGGCCATTGCAACGTGCCGATAACGCTCTGCAACGCTTTCAGATCGTCCAG
query      TGATGTCGTCGGCCATTGCAACGTGCCGATAACGCTCTGCAACGCTTTCAGATCGTCCAG

template   CGAGGCTATATTTTCCTGCGATCCGCAAAGATGCGTCAACGACGTTTCCACGTGTTCGGA
query      CGAGGCTATATTTTCCTGCGATCCGCAAAGATGCGTCAACGACGTTTCCACGTGTTCGGA

template   CTGATAATAATAGTTCGTCCCCCAGAATTCATTATTAAACGGCCAGGTATCCCGGGGCGC
query      CTGATAATAATAGTTCGTCCCCCAGAATTCATTATTAAACGGCCAGGTATCCCGGGGCGC

template   GGCAGAGAGCTCGGCTTTCAGGTACGGGCGGCGCAGCGTAACGCCGTTCACCTGCACGGT
query      GGCAGAGAGCTCGGCTTTCAGGTACGGGCGGCGCAGCGTAACGCCGTTCACCTGCACGGT

template   ATCCGGCATATGACCCCAGTATGACGCATACGGCGTATCCGGGCTGGTTTGTACCGTAAA
query      ATCCGGCATATGACCCCAGTATGACGCATACGGCGTATCCGGGCTGGTTTGTACCGTAAA

template   GATTGCATCTACGCTCGCCGCCTCATCAAATTGATTCGCAAACGCCGCCGTGACAGACGT
query      GATTGCATCTACGCTCGCCGCCTCATCAAATTGATTCGCAAACGCCGCCGTGACAGACGT

template   TTTTAACCCCAGCGATTTATCCTGAGTTAACGTAAACCGCAACTTACCGTCATCGCCGGT
query      TTTTAACCCCAGCGATTTATCCTGAGTTAACGTAAACCGCAACTTACCGTCATCGCCGGT

template   GAAGCCAGACCATTTTGTGCTGAGGCCATTCTGGTTATTAAACGCCACTGCCGTACCCGC
query      GAAGCCAGACCATTTTGTGCTGAGGCCATTCTGGTTATTAAACGCCACTGCCGTACCCGC

template   AGGTGCAACCGGCGCCAGCATCATATTCGCCGGCGAAGTTGTAAACGGAACCGGCTCGCC
query      AGGTGCAACCGGCGCCAGCATCATATTCGCCGGCGAAGTTGTAAACGGAACCGGCTCGCC

template   AATCACGCCTGGTTCAATGTATTGCTTATCCACCGCACCGCGCGAATAAGCGTAATCACG
```

```
query       AATCACGCCTGGTTCAATGTATTGCTTATCCACCGCACCGCGCGAATAAGCGTAATCACG

template    CGTCAACAGTACGGCCACCCCGGCCTGCGGCTGCCCGGCGTCGTTAGTCACCGTTACCGT
query       CGTCAACAGTACGGCCACCCCGGCCTGCGGCTGCCCGGCGTCGTTAGTCACCGTTACCGT

template    CATCGGTATCGTTTCGCCTTTTTTCGCCACGGCCGCCGATTTATCGGCGTTCCATGCGTC
query       CATCGGTATCGTTTCGCCTTTTTTCGCCACGGCCGCCGATTTATCGGCGTTCCATGCGTC

template    GGAAGAGAGCGCGATATTCAGCCCGCTGCGCGATGTTGTCAGGCAGAGTTGTAAGTAGTA
query       GGAAGAGAGCGCGATATTCAGCCCGCTGCGCGATGTTGTCAGGCAGAGTTGTAAGTAGTA

template    GTTACCCGGGTTTTCCGACATGCTGTAGTTTTTCCCCGTCTTGAGATCTTTATACTGCCA
query       GTTACCCGGGTTTTCCGACATGCTGTAGTTTTTCCCCGTCTTGAGATCTTTATACTGCCA

template    GTAAAGGGTTGAGCCTTTCAGCAGGCTATCCCCCGCCCACCACCGCTTCCCGACGGGCAA
query       GTAAAGGGTTGAGCCTTTCAGCAGGCTATCCCCCGCCCACCACCGCTTCCCGACGGGCAA

template    TCCAATATCGGTCGCCACCTTGCCGCTTGGGTGATCGTTGTACAGCGCAGTGAAATCATC
query       TCCAATATCGGTCGCCACCTTGCCGCTTGGGTGATCGTTGTACAGCGCAGTGAAATCATC

template    TATCGCAGCCATCTGGTTCATCGGACATCCGCCGTAATTACCGGTATTAAAACTATTCAC
query       TATCGCAGCCATCTGGTTCATCGGACATCCGCCGTAATTACCGGTATTAAAACTATTCAC

template    CATGATCCAGTTTTCATTGGTTTCGAAATAGGTATCCGTGTGCGCCTTCGATGAGGGCTC
query       CATGATCCAGTTTTCATTGGTTTCGAAATAGGTATCCGTGTGCGCCTCCGATGAGGGCTC

template    GCCTGCCACCAGCGGGCGCTTAAACTCCGCACCGTTGCTGGCGGTGAAGGTCTCCGGCAT
query       GCCTGCCACCAGCGGGCGCTTAAACTCCGCACCGTTGCTGGCGGTGAAGGTCTCCGGCAT

template    ATGGCCGTACATGTTGGCCTTGTCGCTGTCCGGGCTGGTGAGGACGGTGAACACCACCGG
query       ATGGCCGTACATGTTGGCCTTGTCGCTGTCCGGGCTGGTGAGGACGGTGAACACCACCGG

template    CAGCGATGAGGTCGCCGTCGGCGTATCGTTCAGATTCGCGGTCAGCTGGTTTTTCAACCC
query       CAGCGATGAGGTCGCCGTCGGCGTATCGTTCAGATTCGCGGTCAGCTGGTTTTTCAACCC

template    TATGCCGCCGGGTTCGGCAAGCGTCAGGGCCAGCGTACCATCCGCGCCGGTGGCGCCGGT
query       TATGCCGCCGGGTTCGGCAAGCGTCAGGGCCAGCGTACCATCCGCGCCGGTGGCGCCGGT

template    CAGAACCGATGTGGCATAGTAAAACTGTTGCTGCGCACCGGTGAGCGGCGTCACCGTCAT
query       CAGAACCGATGTGGCATAGTAAAACTGTTGCTGCGCACCGGTGAGCGGCGTCACCGTCAT

template    GTTGGTGCCGTTAGTGGCTACAAATTTATTATAGTCAGGGTTGCGCGCCTTCCCGGCGTC
query       GTTGGTGCCGTTAGTGGCTACAAATTTATTATAGTCAGGGTTGCGCGCCTTCCCGGCGTC

template    ACGTTTAAGCGAAAAAGTCGCATAAGGCACCGGGTTGCCCATCGCATCGGTAGTGGTGAT
query       ACGTTTAAGCGAAAAAGTCGCATAAGGCACCGGGTTGCCCATCGCATCGGTAGTGGTGAT

template    GAGTAGCGGGATCGACTCGCTGTTTTTCGCCTTCACCGCCTGAATAGCGTCATCCATCGC
query       GAGTAGCGGGATCGACTCGCTGTTTTTCGCCTTCACCGCCTGAATAGCGTCATCCATCGC

template    CTGCGGGGAGGTGAGGGTAATCTGCGTGGCCTGCGGCAGCGGCGTCGTGGCGCAAATTTG
query       CTGCGGGGAGGTGAGGGTAATCTGCGTGGCCTGCGGCAGCGGCGTCGTGGCGCAAATTTG

template    ATACTGCCAGGCAGTGCTGGTCGCGCTGTTTTCTGACCGACGCGAACCATCAGCCAGATC
query       ATACTGCCAGGCAGTGCTGGTCGCGCTGTTTTCTGACCGACGCGAACCATCAGCCAGATC

template    GACTGCGTAATAGAAGTCAAGTTTTGCCCCGCTGGAAAATGACGTCGAGTTCGATCCGCT
query       GACTGCGTAATAGAAGTCAAGTTTTGCCCCGCTGGAAAATGACGTCGAGTTCGATCCGCT

template    CCAGTAAGAATAGTAGACCGGCCAGCCCTGGGCGGTATTTATCGTTCGGCTCGGGTATTT
query       CCAGTAAGAATAGTAGACCGGCCAGCCCTGGGCGGTATTTATCGTTCGGCTCGGGTATTT

template    GCTGTAGAGCGAATCCAGCGCCTCAACGGCAGGGTAATAGCCCGCCGCGCAACGGGCCGG
query       GCTGTAGAGCGAATCCAGCGCCTCAACGGCAGGGTAATAGCCCGCCGCGCAACGGGCCGG

template    ATTGGCCCCTTCACTGGCTGGCCCATGAAAAACCGCCCAGGTTTCGTTGGTTTCTTGCTT
query       ATTGGCCCCTTCACTGGCTGGCCCATGAAAAACCGCCCAGGTTTCGTTGGTTTCTTGCTT

template    AGACGCAGTATTGTCCGTACTGGACAGCTCAGCGTACAGCAGCGGGCGCTGGTACACCGC
query       AGACGCAGTATTGTCCGTACTGGACAGCTCAGCGTACAGCAGCGGGCGCTGGTACACCGC
```

```
template    GCCATCCGCCGCCGTCAGCGATGGCGCCATATGCCCCCACATGCGGGCAACGCTGACATC
query       GCCATCCGCCGCCGTCAGCGATGGCGCCATATGCCCCCACATGCGGGCAACGCTGACATC

template    CGGGCTGGTGACGACGGTAAAGATAGTGTCGATGCTGGCACTCACGGCGGCATTTTCATA
query       CGGGCTGGTGACGACGGTAAAGATAGTGTCGATGCTGGCACTCACGGCGGCATTTTCATA

template    CAGCGTCGCGTTAATCGCGGTGCGGGTGCCGTGGGTGTCCGGGCGGGTGACGCTGATGAT
query       CAGCGTCGCGTTAATCGCGGTGCGGGTGCCGTGGGTGTCCGGGCGGGTGACGCTGATGAT

template    ACGGGTGCCGTCAGTGCCGGTCTGCGACCCCATTTTGGTGGTGGTCCATGCCAGCGATTC
query       ACGGGTGCCGTCAGTGCCGGTCTGCGACCCCATTTTGGTGGTGGTCCATGCCAGCGATTC

template    GCCGTCGATCACCACGGGCGTCACGATGCCTTGCAGATCGTCACCGTCCTGAGCGGTATA
query       GCCGTCGATCACCACGGGCGTCACGATGCCTTGCAGATCGTCACCGTCCTGAGCGGTATA

template    CTTTTCGCCCCGGCGATCGTAACCGTCGCCGCGGGTGAGCACAAACGGCGCTTCCGGGAT
query       CTTTTCGCCCCGGCGATCGTAACCGTCGCCGCGGGTGAGCACAAACGGCGCTTCCGGGAT

template    CGGGTTGCCGCTGACGTCCTTCACCGTCACCTTGAGCTGCATCGTCTCGCCCTTTTTCAC
query       CGGGTTGCCGCTGACGTCCTTCACCGTCACCTTGAGCTGCATCGTCTCGCCCTTTTTCAC

template    TTTGACCGCGTGGACGTCGCTGCCGTCGTACCACTGCGACGGATTGACCGGTTCAATGGT
query       TTTGACCGCGTGGACGTCGCTGCCGTCGTACCACTGCGACGGATTGACCGGTTCAATGGT

template    GATGGACGCCGCCACCGGGTTATCGCTGGCCAGGCAGCTGGCGTAAATCGAGACGTTGCT
query       GATGGACGCCGCCACCGGGTTATCGCTGGCCAGGCAGCTGGCGTAAATCGAGACGTTGCT

template    GTTGGCAAATTCACTGCCGTTCGTCAGCGCGATCAGTTTCCAGGTGGTGGCGCTCTGGTA
query       GTTGGCAAATTCACTGCCGTTCGTCAGCGCGATCAGTTTCCAGGTGGTGGCGCTCTGGTA

template    GGTGGACGACCAGTAGTTAAGATAGGTCGGCCAGCCCTGAATGCTGTGAATCTTGCCGCC
query       GGTGGACGACCAGTAGTTAAGATAGGTCGGCCAGCCCTGAATGCTGTGAATCTTGCCGCC

template    GCTGTTGGCGTTATACAGCGCCTCAAGCTGGTCGATGCGCGGCAGGCGGTTCGCCGCGCA
query       GCTGTTGGCGTTATACAGCGCCTCAAGCTGGTCGATGCGCGGCAGGCGGTTCGCCGCGCA

template    GCCGCCCGCATCCGGGTTGCCCACCGCGTCGCTATGGGCCACGCGCGCCCAGGTCTCATT
query       GCCGCCCGCATCCGGGTTGCCCACCGCGTCGCTATGGGCCACGCGCGCCCAGGTCTCATT

template    GCTCTCGTCCTGGGTACGCAGCGGGTTGCTCACTTCACCCGCCAGTTTGGGCCGCTCAAA
query       GCTCTCGTCCTGGGTACGCAGCGGGTTGCTCACTTCACCCGCCAGTTTGGGCCGCTCAAA

template    GGTCCAGTCGCCCACGGTAATCGTGTCGGCCATATGGCCCCACATTTTGGCCGCTGGCGT
query       GGTCCAGTCGCCCACGGTAATCGTGTCGGCCATATGGCCCCACATTTTGGCCGCTGGCGT

template    ATCCGGGCTGGTGGCGACGGTAAACTTCACGCTGCGGGAAACCGGCGTGCTGATTAATGA
query       ATCCGGGCTGGTGGCGACGGTAAACTTCACGCTGCGGGAAACCGGCGTGCTGATTAATGA

template    ATCGACGGGCGCAATATTTAACGGCGTCAGCAGCCCCACGCCCTTTTTCTGGGTGAGGAT
query       ATCGACGGGCGCAATATTTAACGGCGTCAGCAGCCCCACGCCCTTTTTCTGGGTGAGGAT

template    AATGGTCGCATCGCCGTTAGCGTCGGTGATCCCGTGATAAACCTGCCCGGCAACGTAGCC
query       AATGGTCGCATCGCCGTTAGCGTCGGTGATCCCGTGATAAACCTGCCCGGCAACGTAGCC

template    CACGTCGTCAAACTGCATCTCGCCGTTGCTGGTATCGGTAAAGCCGGTCGTCAGCCCGTC
query       CACGTCGTCAAACTGCATCTCGCCGTTGCTGGTATCGGTAAAGCCGGTCGTCAGCCCGTC

template    ACGCCGCCTGCCGTTCGCCATGGTGATGGTGAAGTCGGTATTGCCGATCGCCATGCCGTT
query       ACGCCGCCTGCCGTTCGCCATGGTGATGGTGAAGTCGGTATTGCCGATCGCCATGCCGTT

template    AAGGGCGTTGCGCGAATGGATGTTCATCTTAACCTGTTCACCCACTTTGGCGTCGGCGTT
query       AAGGGCGTTGCGCGAATGGATGTTCATCTTAACCTGTTCACCCACTTTGGCGTCGGCGTT

template    TTTGATCACCGGGTCGTCGTTGAAGTAGACGTCCAGCACCGCCACCATTTCATTGGCCGA
query       TTTGATCACCGGGTCGTCGTTGAAGTAGACGTCCAGCACCGCCACCATTTCATTGGCCGA

template    GCAGGTCAGGTAGTTGGGCGTATCGGTAAATTTGCCGCTATCGCCGTTTTCGAGGTTGAC
query       GCAGGTCAGGTAGTTGGGCGTATCGGTAAATTTGCCGCTATCGCCGTTTTCGAGGTTGAC
```

```
template    GTGTACCGTCCCGGAGGCGTCGCTGTCGGCGGCGATGAAATAATCTTCAGTCGGCCAGCC
query       GTGTACCGTCCCGGAGGCGTCGCTGTCGGCGGCGATGAAATAATCTTCAGTCGGCCAGCC

template    GTGCTCGCTTTCCATCTGTCCGCCCGCATGTTCGCTGTACAAATGCGCCAGCGACGACTG
query       GTGCTCGCTTTCCATCTGTCCGCCCGCATGTTCGCTGTACAAATGCGCCAGCGACGACTG

template    ATCCGGCACCTGGCCGACACCGCACTGCGCGGTGGCCGACGCCACGGAGTTAAACGTCGC
query       ATCCGGCACCTGGCCGACACCGCACTGCGCGGTGGCCGACGCCACGGAGTTAAACGTCGC

template    CCACTCTTCGTTATTTTCGCTGAATTGGCCGTCTTTATGGCTGGCCTCCACCGCCAGCAG
query       CCACTCTTCGTTATTTTCGCTGAATTGGCCGTCTTTATGGCTGGCCTCCACCGCCAGCAG

template    CGGACGTTTGTACAGGTTGCCCGCGTCCACCACGCCGCGCATATGGCCCCACATGTTGGC
query       CGGACGTTTGTACAGGTTGCCCGCGTCCACCACGCCGCGCATATGGCCCCACATGTTGGC

template    GCCGTCCACGTCCGGGCTGGTGACCACGGTGAAAATTACGCTAAAGGACAGCTCGACGTT
query       GCCGTCCACGTCCGGGCTGGTGACCACGGTGAAAATTACGCTAAAGGACAGCTCGACGTT

template    GCCGCCTTCGTCGTCCGGCATCACGATGCGGATGGGCGTTTCGACGCCCAGGCCGCTGTT
query       GCCGCCTTCGTCGTCCGGCATCACGATGCGGATGGGCGTTTCGACGCCCAGGCCGCTGTT

template    ATGTTTTAACGTCAGGTGCGCCTGGCCGTTGACGTCAGTCACGCCGGTATAGTGCTCCGG
query       ATGTTTTAACGTCAGGTGCGCCTGGCCGTTGACGTCAGTCACGCCGGTATAGTGCTCCGG

template    CGACGAGGCGCTCATACCTTCGGAATCAACGGTGACCCGCAGGTTATCCCATGCGTCCTG
query       CGACGAGGCGCTCATACCTTCGGAATCAACGGTGACCCGCAGGTTATCCCATGCGTCCTG

template    ATGTTCCCCTTTGCGGTTTTTCGCCGGGTCGATAAACAGCTCCATGTAGCGGTAAGGCAG
query       ATGTTCCCCTTTGCGGTTTTTCGCCGGGTCGATAAACAGCTCCATGTAGCGGTAAGGCAG

template    CGGTTTTTGCGTGTCCCGGTCAACTACGGTCATCGTCAGATCAATCGACTCCCCCACCTG
query       CGGTTTTTGCGTGTCCCGGTCAACTACGGTCATCGTCAGATCAATCGACTCCCCCACCTG

template    CGCTTTATACGGTCCTTGTGGGGAGAGAGTAATCTGCGGGTGCGCCGCAGGCTGGGCTTT
query       CGCTTTATACGGTCCTTGTGGGGAGAGAGTAATCTGCGGGTGCGCCGCAGGCTGGGCTTT

template    ATCCACACAACTGACCAGCAGCGTGTCGCTGTCGGCGGCCCTGACGACCGATGCGCTGTT
query       ATCCACACAACTGACCAGCAGCGTGTCGCTGTCGGCGGCCCTGACGACCGATGCGCTGTT

template    CATATGTACGGCAGCGTGGTAGCCGCTCAGTTGGTTTTTGGTCGATGACCAATATTCAGC
query       CATATGTACGGCAGCGTGGTAGCCGCTCAGTTGGTTTTTGGTCGATGACCAATATTCAGC

template    ATCGCCCGCCACCGGCCAGCCGGTCCAGGTTTCCAGCGTGCTGTGCTCATGTCGCATCTG
query       ATCGCCCGCCACCGGCCAGCCGGTCCAGGTTTCCAGCGTGCTGTGCTCATGTCGCATCTG

template    CACCAGCTGTTCGGCGTCCGGCAGAATGTCACAGTGGTTATCCGCGCCGCTCCAGGTAAA
query       CACCAGCTGTTCGGCGTCCGGCAGAATGTCACAGTGGTTATCCGCGCCGCTCCAGGTAAA

template    CAGCGCCCAGGTTTCGTTGGTATCGACGACGCTTTTGTCAGCGCCGCTCGCTTCCGCCGC
query       CAGCGCCCAGGTTTCGTTGGTATCGACGACGCTTTTGTCAGCGCCGCTCGCTTCCGCCGC

template    CAGCTTCGGGCGGGTGAAGGTGTAGGTTATACCGTTCAGCGTCGCGGTAGCGCTCTCTAT
query       CAGCTTCGGGCGGGTGAAGGTGTAGGTTATACCGTTCAGCGTCGCGGTAGCGCTCTCTAT

template    CATGTGGCCGTACATACTGGCCTTATCGCTGTCCGGGCTGGTGATGGTGGTGAAAATGAC
query       CATGTGGCCGTACATACTGGCCTTATCGCTGTCCGGGCTGGTGATGGTGGTGAAAATGAC

template    GGCGGTTTCAGCCGTAAGCGCCGGAAAGTTGACCGAGCTGACAACCAGCGGCGTTTTGAC
query       GGCGGTTTCAGCCGTAAGCGCCGGAAAGTTGACCGAGCTGACAACCAGCGGCGTTTTGAC

template    GCCGGGGCCTTCCTTCTGGGTGACCACCACCGTGGCGTTGCCGTTGGCGTCAGTGACGCC
query       GCCGGGGCCTTCCTTCTGGGTGACCACCACCGTGGCGTTGCCGTTGGCGTCAGTGACGCC

template    GCGATATTCCGTTTGGGTGGTCGTGAGCTCGGTATCGCCAACGCGAACCGGGTTGGCGTT
query       GCGATATTCCGTTTGGGTGGTCGTGAGCTCGGTATCGCCAACGCGAACCGGGTTGGCGTT

template    ATTGACCACACCCTGGCGGTTTTCCGCATCTTTACGGCGGATGACAAACGGCGCGTTGCC
query       ATTGACCACACCCTGGCGGTTTTCCGCATCTTTACGGCGGATGACAAACGGCGCGTTGCC

template    TTCGGGCGTGCCGTCGCACGCTTTGGTGGAGATGGTGAGGGTGATGCTTTCCCCCACCTT
```

```
query     TTCGGGCGTGCCGTCGCACGCTTTGGTGGAGATGGTGAGGGTGATGCTTTCCCCCACCTT

template  CACTTTTGATGCCTCAAAGGCCGAATCAACGTGATCGCCGCTGACTGTAACCGGCGTGTC
query     CACTTTTGATGCCTCAAAGGCCGAATCAACGTGATCGCCGCTGACTGTAACCGGCGTGTC

template  GGTGGCCGGTTTAATGTTGAGCGCCACTTTCGGGCTTAACAGCGGAATGGTCGCATAATT
query     GGTGGCCGGTTTAATGTTGAGCGCCACTTTCGGGCTTAACAGCGGAATGGTCGCATAATT

template  CGGCACCCCGGTACGCGTAAAGAGCCACAGCGCGGGAACGTTGGTTTCATCTTCCACCTG
query     CGGCACCCCGGTACGCGTAAAGAGCCACAGCGCGGGAACGTTGGTTTCATCTTCCACCTG

template  CGCCCAGGCCACCAGCTGTCGCCCGGCGAGGTTTTGGGCGCAAAAGGTCAGGTTCGGAAC
query     CGCCCAGGCCACCAGCTGTCGCCCGGCGAGGTTTTGGGCGCAAAAGGTCAGGTTCGGAAC

template  CGGCTGCGGCGAGAGCGGCGTGTCGGGCGTGGCGGCATCGGCCCATACCAGGCCCATGGC
query     CGGCTGCGGCGAGAGCGGCGTGTCGGGCGTGGCGGCATCGGCCCATACCAGGCCCATGGC

template  GGGCGGCTGGCGGTTTTCCCAGGCGATGGTGGGCGGATCGCTAAAGAGATCGCCTTCGGT
query     GGGCGGCTGGCGGTTTTCCCAGGCGATGGTGGGCGGATCGCTAAAGAGATCGCCTTCGGT

template  ATCGGTGGAATTGACCGCCCGCATCGAGGTTGAGGTAGCATCCGCGCTAAAATCGCGCGG
query     ATCGGTGGAATTGACCGCCCGCATCGAGGTTGAGGTAGCATCCGCGCTAAAATCGCGCGG

template  CATGGCGCTGAATTCAATATCGTGGGTTTTGCTCGGGTCAAGCAGCGTACTTCCCTGGTA
query     CATGGCGCTGAATTCAATATCGTGGGTTTTGCTCGGGTCAAGCAGCGTACTTCCCTGGTA

template  AAGGGGGATAATAGCGCCGTCGGCCAGCGGCGCGGTGCCGTTAACCGCACCGGTGACTGA
query     AAGGGGGATAATAGCGCCGTCGGCCAGCGGCGCGGTGCCGTTAACCGCACCGGTGACTGA

template  GTTCAGCTCTTGCCAGGTTCCGCCCTTCAATAGCGCCTGCGCAGGTGCGATCGACACTGC
query     GTTCAGCTCTTGCCAGGTTCCGCCCTTCAATAGCGCCTGCGCAGGTGCGATCGACACTGC

template  AAACATGATCAGTAATGTCAGTAAGAAATGTACAACTTTATTCACTTGCCGATTCCTGTC
query     AAACATGATCAGTAATGTCAGTAAGAAATGTACAACTTTATTCACTTGCCGATTCCTGTC

template  CATGAGCGATAGCCACCCGTTTTCATTCTCTGTCGTAATTCAACCCACTTTGCTTAGCGC
query     CATGAGCGATAGCCACCCGTTTTCATTCTCTGTCGTAATTCAACCCACTTTGCTTAGCGC

template  GCGGCTAAGCAAAGTGCGCGGAAAATACCACTCCCCGCGGGGAGTGGTATGTTGAGGACA
query     GCGGCTAAGCAAAGTGCGCGGAAAATACCACTCCCCGCGGGGAGTGGTATGTTGAGGACA

template  ACTTATTTGCGTTTGTAGTCGATGGACAGACCAAAGCCCTGGACACCGTCGCTACCCACG
query     ACTTATTTGCGTTTGTAGTCGATGGACAGACCAAAGCCCTGGACACCGTCGCTACCCACG

template  GTAACGCCGCCGTCTGCTCCCTCGAACGCCGTTTTCGCTTCGGCGTTGGTGACAGGGATG
query     GTAACGCCGCCGTCTGCTCCCTCGAACGCCGTTTTCGCTTCGGCGTTGGTGACAGGGATG

template  ACAAGGTCTTTATCGTTCCAGCTTTCGTTGACGATACCGCCGGTACCGGTACCTGCAATT
query     ACAAGGTCTTTATCGTTCCAGCTTTCGTTGACGATACCGCCGGTACCGGTACCTGCAATT

template  TTACTGGTGCCGACGAATTTCCAGCGGTAGTCATACTGGTCGGTAACGTTTTCACCGGCA
query     TTACTGGTGCCGACGAATTTCCAGCGGTAGTCATACTGGTCGGTAACGTTTTCACCGGCA

template  TCATAGGTACCGTTGCCGTTCTTATCGCTCCACAGCAATACCTGATAGGTGGTATTCGTT
query     TCATAGGTACCGTTGCCGTTCTTATCGCTCCACAGCAATACCTGATAGGTGGTATTCGTT

template  TTGAGTGTCGTCCCTGAATTCTTCAACAGGTTGGTATTCGAATCTTTCTCATGAATAACC
query     TTGAGTGTCGTCCCTGAATTCTTCAACAGGTTGGTATTCGAATCTTTCTCATGAATAACC

template  ACATGGACGTTTTCATCCACCACCGGACCTTCCGGAATTTCGTCATCATCCGAACCACCG
query     ACATGGACGTTTTCATCCACCACCGGACCTTCCGGAATTTCGTCATCATCCGAACCACCG

template  CCCGCATCGGTCGACAGGTCTTTCAACAGCAGTTCGGTTGCCACGGCAGGGTCACCGGTG
query     CCCGCATCGGTCGACAGGTCTTTCAACAGCAGTTCGGTTGCCACGGCAGGGTCACCGGTG

template  GTAGTGGTTGGCGTGATTTTGATACCGATATAACGATCCGCATCAGCGGCCTGGATTTCA
query     GTAGTGGTTGGCGTGATTTTGATACCGATATAACGATCCGCATCAGCGGCCTGGATTTCA

template  TAACTGTCACCAGTACCAAGATCTTTGGGGTCGCTGCCATTCTGATCGCTAAAGCTCACC
query     TAACTGTCACCAGTACCAAGATCTTTGGGGTCGCTGCCATTCTGATCGCTAAAGCTCACC
```

```
template  CATTTCACGGTGGCTTTAGTTGCCGTGTTGTCGGTGTCCAGGTCGCCTTCCGTATCGCCA
query     CATTTCACGGTGGCTTTAGTTGCCGTGTTGTCGGTGTCCAGGTCGCCTTCCGTATCGCCA

template  ATCGCCCAGTTCACCGTGACCTTATCGCCAACGTGGAACTGCTTATCGCCTTCGGTCACA
query     ATCGCCCAGTTCACCGTGACCTTATCGCCAACGTGGAACTGCTTATCGCCTTCGGTCACA

template  ATTTTACGATCGCCGCGATCGATAGTTACCGTTACGTGATCTTTGTCGGTATCCGCAGTT
query     ATTTTACGATCGCCGCGATCGATAGTTACCGTTACGTGATCTTTGTCGGTATCCGCAGTT

template  TTATCCGCCGCACGGGTAATCCACGGCACCGTACCCTGAATGGTGCCCGTCTGTTCAGAG
query     TTATCCGCCGCACGGGTAATCCACGGCACCGTACCCTGAATGGTGCCCGTCTGTTCAGAG

template  ACAATCTGCCACTGCCCCGATTTCAGATTACCGTTTGCCGCCACCGCTGGCGCGGCGCAA
query     ACAATCTGCCACTGCCCCGATTTCAGATTACCGTTTGCCGCCACCGCTGGCGCGGCGCAA

template  TACCCGGCAACTACTAATGCTAAAGCGACCTTCGTCAGCCGCCGTTTTACTGTTGCTTGC
query     TACCCGGCAACTACTAATGCTAAAGCGACCTTCGTCAGCCGCCGTTTTACTGTTGCTTGC

template  ATTCAGACTCTCCTTTTTCTCAAAATTCAGCGGCTGGCTGGTCAGCCACAACGCTGACGC
query     ATTCAGACTCTCCTTTTTCTCAAAATTCAGCGGCTGGCTGGTCAGCCACAACGCTGACGC

template  TATTTCTGACTATACGTAACGCGTATGCCGTAACCCTGAACGCCATCTTCCACATTGGCG
query     TATTTCTGACTATACGTAACGCGTATGCCGTAACCCTGAACGCCATCTTCCACATTGGCG

template  GCAAACTTCTGCGCCGCCTCTTTATTGGTTACTGGCAGGACAAGATCTTCATTCATGGTG
query     GCAAACTTCTGCGCCGCCTCTTTATTGGTTACTGGCAGGACAAGATCTTCATTCATGGTG

template  TTGGCCAGCGCGCCGGTATGTCCGTTTGTACTCTGCCCGGTAAACTCCCACTGGTAGTCG
query     TTGGCCAGCGCGCCGGTATGTCCGTTTGTACTCTGCCCGGTAAACTCCCACTGGTAGTCG

template  TACAGCGCCATCTCTTCTTCCGTGAGCTGCTCGGACATCTGGAATACGCCGTCTTTATTT
query     TACAGCGCCATCTCTTCTTCCGTGAGCTGCTCGGACATCTGGAATACGCCGTCTTTATTT

template  TTGTCGCGCCACAGCAGGAAACGGTAAGCATTATTGACCGTAGGGTGCTTATCTTCTTTG
query     TTGTCGCGCCACAGCAGGAAACGGTAAGCATTATTGACCGTAGGGTGCTTATCTTCTTTG

template  CCGATAAGGTTGACAGGGTTGGCGGCTTTGACCTGATAAATCACCGCATTCAGCGCGCTC
query     CCGATAAGGTTGACAGGGTTGGCGGCTTTGACCTGATAAATCACCGCATTCAGCGCGCTC

template  AGGTTGTCGCCGATAAAAGTGACATCAACGTAGTTACTGTCGCCATAGGCGTCTGCTTTC
query     AGGTTGTCGCCGATAAAAGTGACATCAACGTAGTTACTGTCGCCATAGGCGTCTGCTTTC

template  GCCTTCCAGCGGAAAGTCCCCGGCAGGGTGCTGGAAAGCGTCACCGTCCCCGCTTCTTTG
query     GCCTTCCAGCGGAAAGTCCCCGGCAGGGTGCTGGAAAGCGTCACCGTCCCCGCTTCTTTG

template  TCACGACTGGCAATAAACACGCAGGGGGTTTCCGTGGTGCAGGGCGTCTGGGCCAGGGTG
query     TCACGACTGGCAATAAACACGCAGGGGGTTTCCGTGGTGCAGGGCGTCTGGGCCAGGGTG

template  ACTTTTTTATCATTGCTGTCGTACAACACGGGCGTCATGGCATCGTCCACCAGTTCGTTA
query     ACTTTTTTATCATTGCTGTCGTACAACACGGGCGTCATGGCATCGTCCACCAGTTCGTTA

template  CCATTGCGATCCGTGGAGTCGCCAAAGGAATCGGTGATCAGCAGATCCATCACCACGCCG
query     CCATTGCGATCCGTGGAGTCGCCAAAGGAATCGGTGATCAGCAGATCCATCACCACGCCG

template  TCTTCGCCATCGGCCAGCGCGCTGGCTTCATGGCTGTGATCGCTGCGATCGGTATCGGCA
query     TCTTCGCCATCGGCCAGCGCGCTGGCTTCATGGCTGTGATCGCTGCGATCGGTATCGGCA

template  ATGTTGTCGACGATCAGCTCAATTTTGCGATCCTGCTGGACGGTGATTTCCACCACGCCG
query     ATGTTGTCGACGATCAGCTCAATTTTGCGATCCTGCTGGACGGTGATTTCCACCACGCCG

template  GAGTTCTGCTTATTGCCCTTTTCATCTTCCAGCGTCATGCGTACTTTCCAGGTGTTCAGG
query     GAGTTCTGCTTATTGCCCTTTTCATCTTCCAGCGTCATGCGTACTTTCCAGGTGTTCAGG

template  GCGGTGCGCTGCTCTTCGGTATCGGCATTAACCCAGGCAGGAAGCACCAGATTCCAGCTG
query     GCGGTGCGCTGCTCTTCGGTATCGGCATTAACCCAGGCAGGAAGCACCAGATTCCAGCTG

template  TTATTGTTGCCTTCGGTTAACGCATTAGCTTTTACCACCGGCATCACCAGCGTACGGGCG
query     TTATTGTTGCCTTCGGTTAACGCATTAGCTTTTACCACCGGCATCACCAGCGTACGGGCG
```

```
template    GCGTTGTTTTTCGCCTGCCAGGAGGCAGAAATAATTTTGTAATGGTGGTTAATCAGCGCG
query       GCGTTGTTTTTCGCCTGCCAGGAGGCAGAAATAATTTTGTAATGGTGGTTAATCAGCGCG

template    TTGACAGTCCATTTGCACTTTTCCAGACCAACAGCGGCCTCAGGCGTATCTTCAATCACG
query       TTGACAGTCCATTTGCACTTTTCCAGACCAACAGCGGCCTCAGGCGTATCTTCAATCACG

template    CATTCCGGGTGTTCATTGGTGGCGTCAGCCTTCAGCCACAACGTGACGTCCAGCGGATCT
query       CATTCCGGGTGTTCATTGGTGGCGTCAGCCTTCAGCCACAACGTGACGTCCAGCGGATCT

template    TTCTCACGATATTCGAGAATGATGAAATTGTTACGATCGACAAAATCGGTGCGGCTGCCC
query       TTCTCACGATATTCGAGAATGATGAAATTGTTACGATCGACAAAATCGGTGCGGCTGCCC

template    ATTAAGGAGTGCTGAATTTTAACGTTTTCCGGATCGAGCTGCGCCGCCAGCGGCGTGCCT
query       ATTAAGGAGTGCTGAATTTTAACGTTTTCCGGATCGAGCTGCGCCGCCAGCGGCGTGCCT

template    ATCTGGTAGTTCACGGTCGCGTTAACGCTAAAGTCGTTACTATCGCCAGTACCGGCTTTG
query       ATCTGGTAGTTCACGGTCGCGTTAACGCTAAAGTCGTTACTATCGCCAGTACCGGCTTTG

template    TAATCCGTACCAACCGTGACCAGCGGTACCGGCGTATATTCCAGCCCCAAAGTGACGGCA
query       TAATCCGTACCAACCGTGACCAGCGGTACCGGCGTATATTCCAGCCCCAAAGTGACGGCA

template    TGAGGATCTTTTTGCAGATTATCGGTGCCAAACAGCGCTACTTCATCGCCGTAGTACTGT
query       TGAGGATCTTTTTGCAGATTATCGGTGCCAAACAGCGCTACTTCATCGCCGTAGTACTGT

template    TCGTAAACTAATTTAGCGCCAAGCTGCGGATAAAACGGCAGCCAGCTTTCCATGCGGATA
query       TCGTAAACTAATTTAGCGCCAAGCTGCGGATAAAACGGCAGCCAGCTTTCCATGCGGATA

template    TCCCAGCCGCGCGCGGGTCGTTCTTCATAAAAGTCGAAATCTTCGGAGTCTTTCCAGTCA
query       TCCCAGCCGCGCGCGGGTCGTTCTTCATAAAAGTCGAAATCTTCGGAGTCTTTCCAGTCA

template    GAAAGAGGGTGGTAATAGTTGCCGGAGAATTTCAGGTAATCCGTCCAGGCTTCAGTTCCC
query       GAAAGAGGATGGTAATAGTTGCCGGAGAATTTCAGGTAATCCGTCCAGGCTTCAGTTCCC

template    AGACCTAATCGACGGTGACCGCGCGTAAAATCGTAGTCATAGAATGCGTTGCCGCCAAGC
query       AGACCTAATCGACGGTGACCGCGCGTAAAATCGTAGTCATAGAATGCGTTGCCGCCAAGC

template    AGCCAGTTGCCGACGTTCTGGCGAACGCCAAGCCCAAAGTTACCAATGGTGCGGTCTTCC
query       AGCCAGTTGCCGACGTTCTGGCGAACGCCAAGCCCAAAGTTACCAATGGTGCGGTCTTCC

template    TTACGCTGAGCCGAAATCTGGCTAAAGAAAAGCGTGCTCTGGTTGTCATACCAGGGAATA
query       TTACGCTGAGCCGAAATCTGGCTAAAGAAAAGCGTGCTCTGGTTGTCATACCAGGGAATA

template    AAATAATCCAGCGAACTGCCGTCCAGATCGCCGCCTTCGCCCACGGACAGGCTGGTACGG
query       AAATAATCCAGCGAACTGCCGTCCAGATCGCCGCCTTCGCCCACGGACAGGCTGGTACGG

template    ATCTTGCCATAAGGTGACATTACACCTTCAAGATATGACTGTGCTGAGGAAGTGATTTGG
query       ATCTTGCCATAAGGTGACATTACACCTTCAAGATATGACTGTGCTGAGGAAGTGATTTGG

template    TTTTGCAAATAGCTACGCGCCTGGGATTCCAGCGCTTCCGGGGTGAGATTATCAAAGCCC
query       TTTTGCAAATAGCTACGCGCCTGGGATTCCAGCGCTTCCGGGGTGAGATTATCAAAGCCC

template    TGGGTGGCGGAATTAATAAAGTAGTCAGCGCCCTGTTCTTTAAAGGACTTTCCTTTATTT
query       TGGGTGGCGGAATTAATAAAGTAGTCAGCGCCCTGTTCTTTAAAGGACTTTCCTTTATTT

template    TCCTGTTCTTCTTTTTTCGCGGATTCGCTGGCTAAATCAGGCAGGGAGGAGTGGCTTTCA
query       TCCTGTTCTTCTTTTTTCGCGGATTCGCTGGCTAAATCAGGCAGGGAGGAGTGGCTTTCA

template    TCATTTGCAGATGCAGAGTTATCTGCGCCAGAAGCCGCAACTGAGACTCCAGACACTAAT
query       TCATTTGCAGATGCAGAGTTATCTGCGCCAGAAGCCGCAACTGAGACTCCAGACACTAAT

template    AGCAAAACCAGAATGCGAATCCTTCGCACCATATGATATCCCTGTCGTTAAATAAAAACG
query       AGCAAAACCAGAATGCGAATCCTTCGCACCATATGATATCCCTGTCGTTAAATAAAAACG

template    CCTCTATGAAAAGATATTATATGGATGATTCAGATGGAATCATAACTAACAGGCCTAAAT
query       CCTCTATGAAAAGATATTATATGGATGATTCAGATGGAATCATAACTAACAGGCCTAAAT

template    ATATTCCTAAAAAGAAAAGTTTAATATGAAACAGATTTATAATATTGATATAATAAATAA
query       ATATTCCTAAAAAGAAAAGTTTAATATGAAACAGATTTATAATATTGATATAATAAATAA

template    AAAGAAAGCTGTTATTTATTTCTAATA-TAATTTTCGGGAATAATATTCCCTATAAATAG
```

```
query     AAAGAAAGCTGTTATTTATTTCTAATATTAATTTTCGGGAATAATATTCCCTATAAATAG

template  TTGAATCAATAAAAAGAATAAAATAACCGCCGGGCAGGATACTATGCTGATATTACGGGA
query     TTGAATCAATAAAAAGAATAAAATAACCGCCGGGCAGGATACTATGCTGATATTACGGGA

template  GTATCGAATAACGCTTTTGCCCTTAGCCGGATAAGACGTCGGCGCCTCTGCTCTGCGGCG
query     GTATCGAATAACGCTTTTGCCCTTAGCCGGATAAGACGTCGGCGCCTCTGCTCTGCGGCG

template  CAACGTCGACAGCGGTTTTATTGATCGGAAATGACGAAATTAACCCGTTTTTTTGAAATT
query     CAACGTCGACAGCGGTTTTATTGATCGGAAATGACGAAATTAACCCGTTTTTTTGAAATT

template  AAACCATGACCGTTCTGGCAAAAATAGTCCACTGCGCCGCAGGCTTTCAAAACCTGTAAC
query     AAACCATGACCGTTCTGGCAAAAATAGTCCACTGCGCCGCAGGCTTTCAAAACCTGTAAC

template  GGCTGGCGGCAGTCAGGACAAAGTGCCTGTAACGAGAAATCTTTAGCGCATGTCTCGCAA
query     GGCTGGCGGCAGTCAGGACAAAGTGCCTGTAACGAGAAATCTTTAGCGCATGTCTCGCAA

template  TGCGCTGTATCGCCATTGCGCTCCAGCGCGTGATGGCAAACGGGGCAGGTTATTTCCATA
query     TGCGCTGTATCGCCATTGCGCTCCAGCGCGTGATGGCAAACGGGGCAGGTTATTTCCATA

template  ACATTCTCCTCAGGCGCGTGACTTACTTTTACTTGAAAGGCCTGATAGCGCAGCGCTTGT
query     ACATTCTCCTCAGGCGCGTGACTTACTTTTACTTGAAAGGCCTGATAGCGCAGCGCTTGT

template  CAGGCCTGGAATATTACTGAAAAAGCATTACTTGCTTTTCTTGATATGTTTCATCAGACG
query     CAGGCCTGGAATATTACTGAAAAAGCATTACTTGCTTTTCTTGATATGTTTCATCAGACG

template  CTTACGTTTGCGCATTTGCGTTGGCGTCAGTGTGTTACGCTTGTTCGCATACGGGTTCTC
query     CTTACGTTTGCGCATTTGCGTTGGCGTCAGTGTGTTACGCTTGTTCGCATACGGGTTCTC

template  CCCTTCTTTAAACTGAATGCGAATCGGCGTCCCCATCACTTCCAACGACTTGCGGAAGTA
query     CCCTTCTTTAAACTGAATGCGAATCGGCGTCCCCATCACTTCCAACGACTTGCGGAAGTA

template  GTTCATCAGATAGCGCTTATAGGAGTCTGGCAGATCTTTTACCTGGTTGCCGTGAATCAC
query     GTTCATCAGATAGCGCTTATAGGAGTCTGGCAGATCTTTTACCTGGTTGCCGTGAATCAC

template  CACAATCGGCGGGTTATAGCCGCCAGCGTGGGCATATTTCAGTTTTACACGGCGACCACG
query     CACAATCGGCGGGTTATAGCCGCCAGCGTGGGCATATTTCAGTTTTACACGGCGACCACG

template  CACCAGCGGCGGCTGATGATCTTCCACCGCCATCGTCATGATACGGGTCAGCATCGCAGT
query     CACCAGCGGCGGCTGATGATCTTCCACCGCCATCGTCATGATACGGGTCAGCATCGCAGT

template  GCTCACCCGGCGAGTGGAACTGTCGTAAGCCTCGCGTACGGACTCAAACAGGTTGCCGAC
query     GCTCACCCGGCGAGTGGAACTGTCGTAAGCCTCGCGTACGGACTCAAACAGGTTGCCGAC

template  GCCGCTGCCGTGTAGCGCAGAGATAAAATGGACGCGGGCAAAATCAATAAAGCCCAGACG
query     GCCGCTGCCGTGTAGCGCAGAGATAAAATGGACGCGGGCAAAATCAATAAAGCCCAGACG

template  GAAATCCAGCGTCTCTTTCACCTGCTCTTTGACTTCCTGGCTCAGGCCATCCCATTTGTT
query     GAAATCCAGCGTCTCTTTCACCTGCTCTTTGACTTCCTGGCTCAGGCCATCCCATTTGTT

template  GACTACGATAACGAGTGAGCGCCCACTATTGAGGATAAAGCCCAGCAGAGACAAATCCTG
query     GACTACGATAACGAGTGAGCGCCCACTATTGAGGATAAAGCCCAGCAGAGACAAATCCTG

template  GTCGGAGATCCCCTCGCGCGCATCGATAACCAACAGTACGACGTTAGCGTCTTCAATCGC
query     GTCGGAGATCCCCTCGCGCGCATCGATAACCAACAGTACGACGTTAGCGTCTTCAATCGC

template  CTGCAACGTTTTAATCACCGAGAACTTTTCTACCGCATCGGTGATTTTTCCGCGCTTACG
query     CTGCAACGTTTTAATCACCGAGAACTTTTCTACCGCATCGGTGATTTTTCCGCGCTTACG

template  CACCCCCGCCGTGTCAATCAGCACATATTCCCGTTCGTCGCGTTCCATCGGGATATAAAT
query     CACCCCCGCCGTGTCAATCAGCACATATTCCCGTTCGTCGCGTTCCATCGGGATATAAAT

template  GCTGTCGCGAGTGGTGCCCGGCATGTCGTATACCACCAAGCGCTTCTTAACCGAG
query     GCTGTCGCGAGTGGTGCCCGGCATGTCGTATACCACCACGCGCT-CTTCACCGAG
```

Not_named_JQ071613

```
template  ATGCGGCGCTCTTTTGTTATCAGTGGTGTACTGGATGAATCTCATCAACGCATTTTGCAA
```

```
query     ATGCGGCGCTCTTTTGTTATCAGTGGTGTACTGGATGAATCTCATCAACGCATTTTGCAA

template  GAAACGTTAGCAGCATTAAAGAAAAAGGATCCCGCTCTTTCTTTAATTTATCAGGATATT
query     GAAACGTTAGCAGCATTAAAGAAAAAGGATCCCGCTCTTTCTTTAATTTATCAGGATATT

template  GCGCCTTCCCATGATGAAAGCAAGTATCTGCCTGCGCCAGTGGCTGGCTTTGTACAGAGT
query     GCGCCTTCCCATGATGAAAGCAAGTATCTGCCTGCGCCAGTGGCTGGCTTTGTACAGAGT

template  CGCCATGGTAATTACTTATTACTGACGAATAAAGAGCGTTTACGTGTAGGGGCATTGTTA
query     CGCCATGGTAATTACTTATTACTGACGAATAAAGAGCGTTTACGTGTAGGGGCATTGTTA

template  CCCAATGGGGGAGAAATTGTCCATCTGAGTGCCGATGTGGTAACGATTAAACATTATGAT
query     CCCAATGGGGGAGAAATTGTCCATCTGAGTGCCGATGTGGTAACGATTAAACATTATGAT

template  ACTTTGATTAACTATCCATTAGATTTTAAG
query     ACTTTGATTAACTATCCATTAGATTTTAAG
```

SPI-1_AF148689

```
template  ACCCCCATTGCTGGCGATTTGTGATAAGGTATGGTGTATATATCAGCAAATAATGCAGAA
query     ACCCCCATTGCTGGCGATTTGTGATAAGGTATGGTGTATATATCAGCAAATAATGCAGAA

template  AATGCACCATGCCAGTGTGTCTATCTTATTGATGTATATGGATTATTAAGATGTCTGCCA
query     AATGCACCATGCCAGTGTGTCTATCTTATTGATGTATATGGATTATTAAGATGTCTGCCA

template  GCCGTTTTATTCTGAAATGATTATATTAATATTGTAAGCTTTCATTACAAAATTGTGCAT
query     GCCGTTTTATTCTGAAATGATTATATTAATATTGTAAGCTTTCATTACAAAATTGTGCAT

template  AAAGTTTGTAAGGGTATCTTTTTGTGACGAAATGAACGCGCGTTGGAATGTAATTATTGG
query     AAAGTTTGTAAGGGTATCTTTTTGTGACGAAATGAACGCGCGTTGGAATGTAATTATTGG

template  CTATAATAATAAAAAAATCGGATTTAAATCATCTCTGTGAGTTTTAATTATTTGTTTATA
query     CTATAATAATAAAAAAATCGGATTTAAATCATCTCTGTGAGTTTTAATTATTTGTTTATA

template  AGAATATAACGATTTTGAGTTCCTTATAGCACACAGGATAAAATATGGTATTGCCTTCAA
query     AGAATATAACGATTTTGAGTTCCTTATAGCACACAGGATAAAATATGGTATTGCCTTCAA

template  TGAATAAATCAGTTGAGGCCATTAGCAATAATCACCTTCAACAGCCGAACAAATTTCCAT
query     TGAATAAATCAGTTGAGGCCATTAGCAATAATCACCTTCAACAGCCGAACAAATTTCCAT

template  TAATAAATGGATTAGCTGACGTAAGAGACTATTATGTCGCAAACTGCTTATTGTTTAAAC
query     TAATAAATGGATTAGCTGACGTAAGAGACTATTATGTCGCAAACTGCTTATTGTTTAAAC

template  TTAATAAAGGCAGTTTGCGAATTGAAAACGAATTTGGGGAGTTCATCGAACGATCTGCGC
query     TTAATAAAGGCAGTTTGCGAATTGAAAACGAATTTGGGGAGTTCATCGAACGATCTGCGC

template  CGTGTTTATTTTTATTGGAAAAAGATCAAACAATCACGCTTAGTATGAGCGAAATAGAAG
query     CGTGTTTATTTTTATTGGAAAAAGATCAAACAATCACGCTTAGTATGAGCGAAATAGAAG

template  GGCATATTGATTTTTCTTCACTGGAAGTTTCCTATGACTTAATGCAAAAATTCTACAAAG
query     GGCATATTGATTTTTCTTCACTGGAAGTTTCCTATGACTTAATGCAAAAATTCTACAAAG

template  TTTTTTACAGTACAAGAAACTATAATGATCGAGAGCTATCATTAAAAACGAAACCAAAGT
query     TTTTTTACAGTACAAGAAACTATAATGATCGAGAGCTATCATTAAAAACGAAACCAAAGT

template  ATTTTTTTCATGCGGACTTGTTGCCAGGGATGAGTGATACTTTTGACTCTATTTTGCATG
query     ATTTTTTTCATGCGGACTTGTTGCCAGGGATGAGTGATACTTTTGACTCTATTTTGCATG

template  GTGTGGCATGTCCACGGGTTTGTAGTAATGTGAGTATTGATGATCATGATTATTCATATT
query     GTGTGGCATGTCCACGGGTTTGTAGTAATGTGAGTATTGATGATCATGATTATTCATATT

template  TCTCATTGATGTATCTTATATCGGCATTTGTACGTAAGCCCGGTGGGTTTGATTTCCTTG
query     TCTCATTGATGTATCTTATATCGGCATTTGTACGTAAGCCCGGTGGGTTTGATTTCCTTG

template  AGCGAGCAATAAAAATTACGACAAAAGAGAAAGTTTATAACATTATTATCAGCGATCTCA
query     AGCGAGCAATAAAAATTACGACAAAAGAGAAAGTTTATAACATTATTATCAGCGATCTCA

template  CCCGCAAATGGTCACAGGCTGAGGTGGCAGGAAAGCTGTTTATGAGCGTATCAAGTCTGA
```

```
query       CCCGCAAATGGTCACAGGCTGAGGTGGCAGGAAAGCTGTTTATGAGCGTATCAAGTCTGA

template    AGCGAAAACTGGCCGCTGAAGAGGTGAGTTTTAGTAAAATATACCTGGATGCTCGTATGA
query       AGCGAAAACTGGCCGCTGAAGAGGMGAGTTTTAGTAAAATATACCTGGATGCTCGTATGA

template    ATCAGGCTATAAAATTATTACGTATGGGGGCTGGAAATATTTCACAAGTCGCGACGATGT
query       ATCAGGCTATAAAATTATTACGTATGGGGGCTGGAAATATTTCACAAGTCGCGACGATGT

template    GTGGCTATGATACGCCTTCTTATTTTATCGCTATTTTTAAACGGCATTTTAAGATTACAC
query       GTGGCTATGATACGCCTTCTTATTTTATCGCTATTTTTAAACGGCATTTTAAGATTACAC

template    CGCTTAGCTTTATGCGTACAATGAACCATTGATTTGTGAAATTATTTTAAACGTTACTAT
query       CGCTTAGCTTTATGCGTACAATGAACCATTGATTTGTGAAATTATTTTAAACGTTACTAT

template    CTGTTTGCGTTACAGATACCAAATATTTTTTTAATCGTAGTGTATATTTCAGTATCGCTA
query       CTGTTTGCGTTACAGATACCAAATATTTTTTTAATCGTAGTGTATATTTCAGTATCGCTA

template    CACCAGTGATATGTAGTTAATGAAAGGGAATCGAAAGCGTTCATTGTCAGACATACATGG
query       CACCAGTGATATGTAGTTAATGAAAGGGAATCGAAAGCGTTCATTGTCAGACATACATGG

template    CCACCAGGCGCCGGGCAATCGTTGTACTCTAAGAGATTACAGTAAAGTAAAACAGCAAAT
query       CCACCAGGCGCCGGGCAATCGTTGTACTCTAAGAGATTACAGTAAAGTAAAACAGCAAAT

template    TTAATCATTATCAGATTTGTAAAAGGAATTCATTTCGGTAACGACATTGCGCATGTAATA
query       TTAATCATTATCAGATTTGTAAAAGGAATTCATTTCGGTAACGACATTGCGCATGTAATA

template    ACGTATCTGGTCTTATTACCTTTGTAGTAAGAAGGCGTTATCTACTTGCGCAGTAGTCGC
query       ACGTATCTGGTCTTATTACCTTTGTAGTAAGAAGGCGTTATCTACTTGCGCAGTAGTCGC

template    CCAGGAAGAAAAATCATTACCGAAGCTGTGGTCAGTTTTCAAAAAAGGATAAATAAATGA
query       CCAGGAAGAAAAATCATTACCGAAGCTGTGGTCAGTTTTCAAAAAAGGATAAATAAATGA

template    GAAATGTAATTATATACGGTATTAACTGGACTAATTGTTATGCCCTACAGTCTATTTTTA
query       GAAATGTAATTATATACGGTATTAACTGGACTAATTGTTATGCCCTACAGTCTATTTTTA

template    AGCAAAAATATCCGGAAAAGTGCGTTAAAACGTGTAACTCATTAACTGCACTTTTGCATT
query       AGCAAAAATATCCGGAAAAGTGCGTTAAAACGTGTAACTCATTAACTGCACTTTTGCATT

template    CCCTATCCGATATGCCGGACGCCGGGCTGATTTTAGCTTTGAACCCGCATGAGCATGTTT
query       CCCTATCCGATATGCCGGACGCCGGGCTGATTTTAGCTTTGAACCCGCATGAGCATGTTT

template    ATCTTTTTCATGCGTTGCTGACGCGGTTGCAGAACCGTAAAGTTTTGGTAGTGGCAGATC
query       ATCTTTTTCATGCGTTGCTGACGCGGTTGCAGAACCGTAAAGTTTTGGTAGTGGCAGATC

template    GCTTGTATTATATCGATCGCTGTGTGCTGCAATATTTTGGCGTTATGGATTATGTTTTAA
query       GCTTGTATTATATCGATCGCTGTGTGCTGCAATATTTTGGCGTTATGGATTATGTTTTAA

template    AAGACGAACTATCCTGCGCTATCCGCTCGGAGCGAGAGAAGCTCCGTCTTCCGGAAGCCT
query       AAGACGAACTATCCTGCGCTATCCGCTCGGAGCGAGAGAAGCTCCGTCTTCCGGAAGCCT

template    GGCTGCGTTTCTGCCACAGGCCACAAAAGAAAACCGTGGCTGCTACGTATGCTTTTAATG
query       GGCTGCGTTTCTGCCACAGGCCACAAAAGAAAACCGTGGCTGCTACGTATGCTTTTAATG

template    CCGGCGAGACGCCGGAAGAGGTATTGTTTAATATCAATCAATACGCCTGGTGGAATCTAC
query       CCGGCGAGACGCCGGAAGAGGTATTGTTTAATATCAATCAATACGCCTGGTGGAATCTAC

template    CTCCCGGTGTCACGCAGGCGAAATATGCGCTGCTGATATTATTATCCTCCGGACATCCGG
query       CTCCCGGTGTCACGCAGGCGAAATATGCGCTGCTGATATTATTATCCTCCGGACATCCGG

template    CAATTGAACTGGCGAAAAAATTTGGTCTGGGAACAAAAACCGTCAGTATTTATCGTAAGA
query       CAATTGAACTGGCGAAAAAATTTGGTCTGGGAACAAAAACCGTCAGTATTTATCGTAAGA

template    AGGTTATGTATCGTTTGGGGATGGACTCTTCACCGCTATCGCTGTTCCGGGGACTGAAAC
query       AGGTTATGTATCGTTTGGGGATGGACTCTTCACCGCTATCGCTGTTCCGGGGACTGAAAC

template    TAGACGCGCATTTACAGCGCACTGCTTTTGCACATAACCCTGCTGTACCTGACGATAATT
query       TAGACGCGCATTTACAGCGCACTGCTTTTGCACATAACCCTGCTGTACCTGACGATAATT

template    GCGCGTTACCGATTGCGGTAGGAATGAATTAAAATATTTTTTATCATTTATGACCATGTG
query       GCGCGTTACCGATTGCGGTAGGAATGAATTAAAATATTTTTTATCATTTATGACCATGTG
```

```
template   TTGAGCTTTTATTATAAAAAAGATTTTTTGAGTAGTAATTCTTATATATAATCATCCGGA
query      TTGAGCTTTTATTATAAAAAAGATTTTTTGAGTAGTAATTCTTATATATAATCATCCGGA

template   GGTGGTTGGTAGCCTGGCTCAATCATTGAGGCATATTTTTGCAGGCAATATATTGAATCT
query      GGTGGTTGGTAGCCTGGCTCAATCATTGAGGCATATTTTTGCAGGCAATATATTGAATCT

template   GAAAAGTTAAAGATGATATTTTCGGTGCAGGAGCTATCATGTGGAGGGAAAAGTATGCTA
query      GAAAAGTTAAAGATGATATTTTCGGTGCAGGAGCTATCATGTGGAGGGAAAAGTATGCTA

template   AGTCCTACGACTCGTAATATGGGGGCGAGTTTATCGCCTCAGCCTGACGTCAGCGGGGAG
query      AGTCCTACGACTCGTAATATGGGGGCGAGTTTATCGCCTCAGCCTGACGTCAGCGGGGAG

template   CTAAACACCGAAGCATTGACCTGTATTGTTGAGCGTCTGGAAAGTGAAATTATAGATGGC
query      CTAAACACCGAAGCATTGACCTGTATTGTTGAGCGTCTGGAAAGTGAAATTATAGATGGC

template   AGCTG
query      AGCTG
```

SPI-1_AY956822

```
template   GGCTCAAGGGGCGCTCATTTCAGTACGGGGCGGAAGGTTATATCAAAATGAGCCCAGGCC
query      GGCTCAAGGGGCGCTCATTTCAGTACGGGGCGGAAGGTTATATCAAAATGAGCCCAGGCC

template   ATTGGTATTTCCCAAGCCCACTTTAATTTAACGTAAATAAGGAAGTCATTATGGCAACAC
query      ATTGGTATTTCCCAAGCCCACTTTAATTTAACGTAAATAAGGAAGTCATTATGGCAACAC

template   CTTGGTCAGGCTATCTGGATGACGTCTCAGCAAAATTTGATACGGGCGTTGATAATCTAC
query      CTTGGTCAGGCTATCTGGATGACGTCTCAGCAAAATTTGATACGGGCGTTGATAATCTAC

template   AAACGCAGGTAACAGAGGCGCTGGATAAATTAGCAGCAAAACCCTCCGATCCGGCGCTAC
query      AAACGCAGGTAACAGAGGCGCTGGATAAATTAGCAGCAAAACCCTCCGATCCGGCGCTAC

template   TGGCGGCGTATCAGAGTAAGCTCTCGGAATATAACTTGTACCGTAACGCGCAATCGAACA
query      TGGCGGCGTATCAGAGTAAGCTCTCGGAATATAACTTGTACCGTAACGCGCAATCGAACA

template   CGGTAAAAGTCTTTAAGGATATTGATGCTGCCATTATTCAGAACTTCCGTTAATCAGTTA
query      CGGTAAAAGTCTTTAAGNATATTGATGCTGCCATTATTCAGAACTTCCGTTAATCAGTTA

template   TAAGGTGGATTATGTCGATTGCAACTATTGTCCCTGAGAATGCCGTTATAGGGCAGGCGG
query      TAAGGTGGATTATGTCGATTGCAACTATTGTCCCTGAGAATGCCGTTATAGGGCAGGCGG

template   TCAATATCAGGTCTATGGAA
query      TCAATATCAGGTCTATGGAA
```

SPI-1_AY956823

```
template   CGCTTGAAGAGGTCAATGGCCACATGGATTTCGATATACTCGAGATACCGACGCAACGAC
query      CGCTTGAAGAGGTCAATGGCCACATGGATTTCGATATACTCGAGATACCGACGCAACGAC

template   TTGGCGCTCTCTATGCACTTATCCCAAACGAGCAGCAAACCAAAATGGCGGTACCCACAG
query      TTGGCGCTCTCTATGCACTTATCCCAAACGAGCAGCAAACCAAAATGGCGGTACCCACAG

template   AGAAAGCGCAGAAGATCTTCTATACGCCTGACTTTCCTGCCAGAAGAGAGGTATTTGAAC
query      AGAAAGCGCAGAAGATCTTCTATACGCCTGACTTTCCTGCCAGAAGAGAGGTATTTGAAC

template   ATCTGAAAACGGCGTTCTCCTGTACGAAGGATACAAGCAAAGGTTGCAGTAACTGTAACA
query      ATCTGAAAACGGCGTTCTCCTGTACGAAGGATACAAGCAAAGGTTGCAGTAACTGTAACA

template   ACAAAAGTTGTATTGAAAATGAAGAGTTAATTCCTTATTTTCTGCTGTTCCTGCTTACTG
query      ACAAAAGTTGTATTGAAAATGAAGAGTTAATTCCTTATTTTCTGCTGTTCCTGCTTACTG

template   CTTTTCTCCGACTCCCGGAGAGTTATGAGATCATCCTTAGCTCGGCTCAGATAACGTTAA
query      CTTTTCTCCGACTCCCGGAGAGTTATGAGATCATCCTTAGCTCGGCTCAGATAACGTTAA

template   AGGAGCGCGTTTACAACATTATATCTTCGTCACCCAGTAGACAGTGGAAGCTTACGGATG
```

```
query       AGGAGCGCGTTTACAACATTATATCTTCGTCACCCAGTAGACAGTGGAAGCTTACGGATG

template    TTGCCGATCA
query       TTGCCGATCA
```

SPI-1_AY956824

```
template    TTTCCAAAATGGGAGGCTGGTTATTACCAGGTAAGGACGGTAATACCGTTAAGCTAGATG
query       TTTCCAAAATGGGAGGCTGGTTATTACCAGGTAAGGACGGTAATACCGTTAAGCTAGATG

template    TTACCTCACTCAAAAATGATTTAAACAGTTTAGTCAATAAATATAATCAAATAAACAGTA
query       TTACCTCACTCAAAAATGATTTAAACAGTTTAGTCAATAAATATAATCAAATAAACAGTA

template    ATACCGTTTTATTTCCAGCGCAGTCAGGCAGCGGCGTTAAAGTAGCCACTGAAGCGGAAG
query       ATACCGTTTTATTTCCAGCGCAGTCAGGCAGCGGCGTTAAAGTAGCCACTGAAGCGGAAG

template    CGAGACAGTGGCTCAGTGAATTGAATTTACCGAATAGCTGCCTGAAATCTTATGGATCCG
query       CGAGACAGTGGCTCAGTGAATTGAATTTACCGAATAGCTGCCTGAAATCTTATGGATCCG

template    GTTATGTCGTCACCGTTGATCTGACGCCATTACAAAAAATGGTTCAGGATATTGATGGTT
query       GTTATGTCGTCACCGTTGATCTGACGCCATTACAAAAAATGGTTCAGGATATTGATGGTT

template    TAGGCGCGCCGGGAAAAGACTCAAAACTCGAAATGGATAACGCCAAATATCAAGCCTGGC
query       TAGGCGCGCCGGGAAAAGACTCAAAACTCGAAATGGATAACGCCAAATATCAAGCCTGGC

template    AGTCGGGTTTTAAAGCGCAGGAAGAAAATATGAAAACCACATTACAGACGCTGACGCAAA
query       AGTCGGGTTTTAAAGCGCAGGAAGAAAATATGAAAACCACATTACAGACGCTGACGCAAA

template    AATATAGCAATGCCAATTCATTGTACGACAACCTGGTAAAAGTGCTGAGC
query       AATATAGCAATGCCAATTCATTGTACGACAACCTGGTAAAAGTGCTGAGC
```

SPI-1_AY956825

```
template    GTCTGGCGGTGACGCTGGCGCGCAACGTCAATGAATATTTCGGTATTCAGGAAACAAAAC
query       GTCTGGCGGTGACGCTGGCGCGCAACGTCAATGAATATTTCGGTATTCAGGAAACAAAAC

template    ATATGCTGGACCAACTGGAAGCGAAATTTCCTGATTTACTTAAAGAAGTGCTCAGACATG
query       ATATGCTGGACCAACTGGAAGCGAAATTTCCTGATTTACTTAAAGAAGTGCTCAGACATG

template    CCACGGTACAACGTATATCTGAAGTTTTGCAGCGTTTATTAAGCGAACGTGTTTCCGTGC
query       CCACGGTACAACGTATATCTGAAGTTTTGCAGCGTTTGTTAAGCGAACGTGTTTCCGTGC

template    GTAATATGAAATTAATTATGGAAGCGCTCGCATTGTGGGCGCCAAGAGAAAAAGATGTCA
query       GTAATATGAAGTTAATTATGGAAGCGCTCGCATTGTGGGCGCCAAGAGAAAAAGATGTCA

template    TTAACCTTGTAGAGCATATTCGTGGAGCAATGGCGCGTTATATTTGTCATAAATTCGCCA
query       TTAACCTTGTGGAGCATATTCGTGGAGCAATGGCGCGTTATATTTGTCATAAATTCGCCA

template    ATGGCGGCGAATTACGAGCAGTAATGGTATCTGCTGAAGTTGAGGATGTTATTCGCAAAG
query       ATGGCGGCGAATTACGAGCAGTAATGGTATCTGCTGAAGTTGAGGATGTTATTCGCAAAG

template    GGATCCGTCAGACCTCTGGCAGTACCTTCCTCAGCCTTGACCCGGAAGCCTCCGC
query       GGATCCGTCAGACCTCTGGCAGTACCTTCCTCAGCCTTGACCCGGAAGCCTCCGC
```

SPI-1_JN982040

```
template    TTCGTTA-TGGCG-TAGCCTGGCGGTGGGCTTTGTTGTCTTCTCTATTGTCACCGTGGTC
query       TTCGTTATTGGCGATAGCCTGGCGGTGGGTTTTGTTGTCTTCTCTATTGTCACCGTGGTC

template    CAGTTTATCGTTATTACCAAAGGTTCAGAACGTGTCGCGGAAGTCGCGGCCCGATTTTCT
query       CAGTTTATCGTTATTACCAAAGGTTCAGAACGTGTCGCGGAAGTCGCGGCCCGATTTTCT

template    CTGGATGGTATGCCCGGTAAACAGATGAGTATTGATGCCGATTTGAAGGCCGGTATTATT
query       CTGGATGGTATGCCCGGTAAACAGATGAGTATTGATGCCGATTTGAAGGCCGGTATTATT
```

```
template    GATGCGGATGCCGCGCGCGAACGGCGAAGCGTACTGGAAAGGGAAAGCCAGCTTTACGGT
query       GATGCGGATGCCGCGCGCGAACGGCGAAGCGTACTGGAAAGGGAAAGCCAGCTTTACGGT

template    TCCTTTGACGGTGCGATGA
query       TCCTTTGACGGTGCGATGA
```

SPI-1_U16303

```
template    GTTCGGAAGCATCCGTTTCCCTCTTAATTTTGTTGAAATTTCTCTTTGTTGCGAGCATGG
query       GTTCGGAAGCATCCGTTTCCCTCTTAATTTTGTTGAAATTTCTCTTTGTTGCGAGCATGG

template    TCTGAACTAATTGCCTGGCAGCTTGCCTTGTTTCGTCAATATTTGGAAAATCGAACACAT
query       TCTGAACTAATTGCCTGGCAGCTTGCCTTGTTTCGTCAATATTTGGAAAATCGAACACAT

template    TTCCAACTTTTACTTTCAAGACAGAACTTGGCTTCCTACCCTCACTGACATTACGATCTG
query       TTCCAACTTTTACTTTCAAGACAGAACTTGGCTTCCTACCCTCACTGACATTACGATCTG

template    ACGAGGCTACTCTTCGCTGGATTACGTAGGTTTTTTTCGTCAAACTGGCTTTTACCCCGA
query       ACGAGGCTACTCTTCGCTGGATTACGTAGGTTTTTTTCGTCAAACTGGCTTTTACCCCGA

template    AACCAGCAGGAGCTTCTCTGTGGTCATCGAAAACAGTGTAGAGTTTTTGAGCTGGGTTAG
query       AACCAGCAGGAGCTTCTCTGTGGTCATCGAAAACAGTGTAGAGTTTTTGAGCTGGGTTAG

template    CTTCGAAAACGAATTTTTCCAGAGCGACTGAGTGCAGGCTTAACATTGATACTTAACTTG
query       CTTCGAAAACG-ATTTTTCCAGAGCGACTGAGTGCAGGCTTAACATTGATACTTAACTTG

template    TTGAT-ATGAAAGGCTGGCTGAGAGTTGTTTTCAAACTATCACCC-TGTTGTAGT-CAAG
query       TTGATAATGAAAGGCTGGCTGAGAGTTGTTTTCAAAGTATCACCCCTGTTGTAGTCCAGG

template    -ATGATGAG-ATGTAA-CTGTCACA-TTTAGGCTACAGGTGAGCTACAAATTTATGGGTT
query       AATGATGAGAATGTAACCTGTCACATTTTAGGCTACAGGTGAGCTACAAATTTATGGGTT

template    TATGTGAGTTTATAAGGGTTTTACCTGGGGCCACATGAGCACTTGAAAGAATAAAAAAAC
query       TATGTGAGTTTATAAGGGTTTTACCTGGGGCCACATGAGCACTTGAAAGAATAAAAAAAC

template    GAGTAAAATCAATATGAATGAGTCATTTGATAAGGACTTCTCCAACCACACCCCGATGAT
query       GAGTAAAATCAATATGAATGAGTCATTTGATAAGGACTTCTCCAACCACACCCCGATGAT

template    GCAGCAGTATCTCAAGCTGAAAGCCCAGCATCCGGAGATCCTGCTCTTTTATCGTATGGG
query       GCAGCAGTATCTCAAGCTGAAAGCCCAGCATCCGGAGATCCTGCTCTTTTATCGTATGGG

template    AGACTTTTACGAGCTGTTTTATGACGACGCAAAGCGTGCGTCGCAGTTGCTCGATATTTC
query       AGACTTTTACGAGCTGTTTTATGACGACGCAAAGCGTGCGTCGCAGTTGCTCGATATTTC

template    GCTGACCAAACGCGGCGCATCGGCTGGCGAACCTATCCCTATGGCGGGTATCCCGCACCA
query       GCTGACCAAACGCGGCGCATCGGCTGGCGAACCTATCCCTATGGCGGGTATCCCGCACCA

template    CGCCGTAGAAAACTACCTCGCGAAACTGGTCAATCAGGGCGAATCCGTCGCTATTTGCGA
query       CGCCGTAGAAAACTACCTCGCGAAACTGGTCAATCAGGGCGAATCCGTCGCTATTTGCGA

template    ACAGATTGGCGATCCGGCCACCAGCAAAGGTCCCGTTGAACGTAAGGTCGTGCGTATCGT
query       ACAGATTGGCGATCCGGCCACCAGCAAAGGTCCCGTTGAACGTAAGGTCGTGCGTATCGT

template    TACGCCTGGCACTATCAGCGATGAAGCCCTGTTACAGGAGCGTCAGGATAACCTGCTGGC
query       TACGCCTGGCACTATCAGCGATGAAGCCCTGTTACAGGAGCGTCAGGATAACCTGCTGGC

template    GGCTATCTGGCAGGATGGTAAAGGTTACGGTTACGCCACGCTGGATATCAGCTCCGGGCG
query       GGCTATCTGGCAGGATGGTAAAGGTTACGGTTACGCCACGCTGGATATCAGCTCCGGGCG

template    CTTTCGTCTGAGCGAACCCGCCGACCGTGAAACGATGGCCGCAGAGCTGCAGCGCACCAA
query       CTTTCGTCTGAGCGAACCCGCCGACCGTGAAACGATGGCCGCAGAGCTGCAGCGCACCAA

template    TCCCGCCGAACTGTTGTATGCCGAAGATTTTGCTGAAATGGCGTTAATAGAGGGACGCCG
query       TCCCGCCGAACTGTTGTATGCCGAAGATTTTGCTGAAATGGCGTTAATAGAGGGACGCCG

template    GGGTCTGCGCCGTCGCCCGTTGTGGGAGTTTGAGATTGATACCGCCCGCCAGCAGTTAAA
query       GGGTCTGCGCCGTCGCCCGTTGTGGGAGTTTGAGATTGATACCGCCCGCCAGCAGTTAAA
```

```
template   TCTGCAGTTCGGTACGCGGGATCTGGTCGGTTTCGGCGTCGAAAATGCCTCGCGTGGATT
query      TCTGCAGTTCGGTACGCGGGATCTGGTCGGTTTCGGCGTCGAAAATGCCTCGCGTGGATT

template   ATGTGCGGCAGGCTGCCTGTTACAGTACGTAAAAGATACCCAGCGCACCTCCCTGCCGCA
query      ATGTGCGGCAGGCTGCCTGTTACAGTACGTAAAAGATACCCAGCGCACCTCCCTGCCGCA

template   TATTCGTTCCATTACGATGGAACGCCAGCAGGACAGCATCATTATGGATGCCGCGACCCG
query      TATTCGTTCCATTACGATGGAACGCCAGCAGGACAGCATCATTATGGATGCCGCGACCCG

template   CCGCAATCTGGAAATTACCCAGAACCTGGCCGGTGGTGTCGAAAATACCCTCGCCGCGGT
query      CCGCAATCTGGAAATTACCCAGAACCTGGCCGGTGGTGTCGAAAATACCCTCGCCGCGGT

template   GCTTGACTGTACCGTGACGCCAATGGGTAGCCGAATGCTTAAACGCTGGCTGCATATGCC
query      GCTTGACTGTACCGTGACGCCAATGGGTAGCCGAATGCTTAAACGCTGGCTGCATATGCC

template   GGTACGAAATACCGACATATTACGTGAACGCCAGCAGACCATCGGCGCCTTGCAGGACAC
query      GGTACGAAATACCGACATATTACGTGAACGCCAGCAGACCATCGGCGCCTTGCAGGACAC

template   CGTCAGCGAACTGCAACCGGTGCTGCGTCAGGTCGGCGATTTGGAGCGTACTCCTTGCGC
query      CGTCAGCGAACTGCAACCGGTGCTGCGTCAGGTCGGCGATTTGGAGCGTA-TCCTTGCGC

template   GTCTGGCGCTGCGCA--GC-CGTCCGCGCGATCTCGCCCGAATGCGTCACGCCTTCCAGC
query      GTCTGGCGCTGCGCACCGCGCGTCCGCGCGATCTCGCCCGAATGCGTCACGCCTTCCAGC

template   AGCTGCC-GAATTGCACGCTCAGCTTGAAACCGTTGACAGCGCGCCGGTACA-GCGTTGC
query      AGCTGCCGGAATTGCACGCTCAGCTTGAAACCGTTGACAGCGCGCCGGTACAGGCGTTGC

template   GTAAAAAAATGGGCGATTTCGCCGAGCTGCGCGACCTCCTGGAACGCGCCATTATTGACG
query      GTAAAAAAATGGGCGATTTCGCCGAGCTGCGCGACCTCCTGGAACGCGCCATTATTGACG

template   CGCCGCCGGTACTGGTC-GCGACGGC-GCGTTATTGCGCCCGGCTACCATGAAGAGCTGG
query      CGCCGCCGGTACTGGTCCGCGACGGCGGCGTTATTGCGCCCGGCTACCATGAAGAGCTGG

template   ACGAATGGCGCGCGCTGGCGGACGGCGCCACCGATTATCTCGATCGTCTGGAAATTCGCG
query      ACGAATGGCGCGCGCTGGCGGACGGCGCCACCGATTATCTCGATCGTCTGGAAATTCGCG

template   AGCGCGAGCGTACCGGGCTGGATACGCTGAAAGTCGGCTATAACGCGGTACATGGTTATT
query      AGCGCGAGCGTACCGGGCTGGATACGCTGAAAGTCGGCTATAACGCGGTACATGGTTATT

template   ACATTCAGATTAGCCGTGGTCAAAGCCATCTGGCGCCCATCAATTATGTGCGTCGCCAGA
query      ACATTCAGATTAGCCGTGGTCAAAGCCATCTGGCGCCCATCAATTATGTGCGTCGCCAGA

template   CGCTGAAAAATGCCGAACGCTACATTATTCCTGAGCTTAAAGAGTACGAAGATAAAGTTC
query      CGCTGAAAAATGCCGAACGCTACATTATTCCTGAGCTTAAAGAGTACGAAGATAAAGTTC

template   TGACCTCGAAAGGCAAAGCGCTGGCGCTGGAAAAACAGCTATATGACGAATTGTTTGATC
query      TGACCTCGAAAGGCAAAGCGCTGGCGCTGGAAAAACAGCTATATGACGAATTGTTTGATC

template   TGCTGTTGCCGCATCTGGCCGATTTACAGCAGAGCGCCAACGCGCTGGCGGAGCTGGATG
query      TGCTGTTGCCGCATCTGGCCGATTTACAGCAGAGCGCCAACGCGCTGGCGGAGCTGGATG

template   TGCTGGTTAACCTGGCCGAACGCGCCTGGACGCTGAATTACACCTGCCCGACATTTACCG
query      TGCTGGTTAACCTGGCCGAACGCGCCTGGACGCTGAATTACACCTGCCCGACATTTACCG

template   ATAAACCCGGTATCCGTATTACCGAAGGTCGCCACCCGGTGGTTGAACAGGTACTGAATG
query      ATAAACCCGGTATCCGTATTACCGAAGGTCGCCACCCGGTGGTTGAACAGGTACTGAATG

template   AGCCGTTTATCGCTAACCCGCTCAATTTATCGCCGCAGCGCCGGATGTTGATCATTACCG
query      AGCCGTTTATCGCTAACCCGCTCAATTTATCGCCGCAGCGCCGGATGTTGATCATTACCG

template   GCCCCAATATGGGCGGTAAAAGTACCTATATGCGACAAACGGCATTGATTGCCCTGCTGG
query      GCCCCAATATGGGCGGTAAAAGTACCTATATGCGACAAACGGCATTGATTGCCCTGCTGG

template   CCTATATCGGCAGTTACGTTCCGGCGCAAAACGTGGAAATCGGCCCCATCGACCGTATTT
query      CCTATATCGGCAGTTACGTTCCGGCGCAAAACGTGGAAATCGGCCCCATCGACCGTATTT

template   TTACCCGCGTCGGCGCAGCGGACGACCTGGCCTCCGGGCGTTCGACCTTTATGGTGGAGA
query      TTACCCGCGTCGGCGCAGCGGACGACCTGGCCTCCGGGCGTTCGACCTTTATGGTGGAGA
```

```
template  TGACCGAAACCGCGAACATTCTGCATAATGCCACGGAAAACAGTCTGGTATTGATGGATG
query     TGACCGAAACCGCGAACATTCTGCATAATGCCACGGAAAACAGTCTGGTATTGATGGATG

template  AAATCGGGCGCGGTACCTCCACGTATGACGGGCTGTCGTTGGCCTGGGCCTGCGCGGAGA
query     AAATCGGGCGCGGTACCTCCACGTATGACGGGCTGTCGTTGGCCTGGGCCTGCGCGGAGA

template  ATCTCGCGAATAAGATTAAAGCGTTAACGCTGTTCGCTACCCACTACTTCGAGCTGACCC
query     ATCTCGCGAATAAGATTAAAGCGTTAACGCTGTTCGCTACCCACTACTTCGAGCTGACCC

template  AATTACCGGAGAAAATGGAAGGCGTGGCGAACGTCCACCTGGATGCGCTGGAACACGGCG
query     AATTACCGGAGAAAATGGAAGGCGTGGCGAACGTCCACCTGGATGCGCTGGAACACGGCG

template  ATACTATCGCGTTTATGCACAGCGTGCAGGACGGCGCGGCAAGTAAGAGCTATGGCCTGG
query     ATACTATCGCGTTTATGCACAGCGTGCAGGACGGCGCGGCAAGTAAGAGCTATGGCCTGG

template  CGGTCGCGGCGCTTGCGGGCGTCCCTAAAGAAGTCATCAAACGCGCGCGCCAGAAACTCC
query     CGGTCGCGGCGCTTGCGGGCGTCCCTAAAGAAGTCATCAAACGCGCGCGCCAGAAACTCC

template  GCGAGCTGGAAAGCATCTCGCCCAATGCGGCGGCAACGCAGGTGGACGGCACGCAAATGT
query     GCGAGCTGGAAAGCATCTCGCCCAATGCGGCGGCAACGCAGGTGGACGGCACGCAAATGT

template  CGTTGCTTGCCGCCCCGGAGGAGACATCGCCTGCCGTTGAAGCGCTGGAGAATCTCGATC
query     CGTTGCTTGCCGCCCCGGAGGAGACATCGCCTGCCGTTGAAGCGCTGGAGAATCTCGATC

template  CGGACTCCCTGACGCCACGCCAGGCGCTGGAGTGGATCTATCGGCTGAAAAGTCTGGTGT
query     CGGACTCCCTGACGCCACGCCAGGCGCTGGAGTGGATCTATCGGCTGAAAAGTCTGGTGT

template  AAACGTGTCAGACCCGATAGCGCCGCGCTATCGGG
query     AAACGTGTCAGACCCGATAGCGCCGCGCTATCGGG
```

SPI-12_NC_006905

```
template  CGGCACGTAGCGCAGCCTGGTAGCGCACCGTCATGGGGTGTCGGGGGTCGGAGGTTCAAA
query     SAGACCACCAGCACGATAGATAACCGACTCATTACRGAACACATCACGAACAGCAGGATC

template  TCCTCTCGTGCCGACCAAAATTCCCCTTAAAAGCCAGCCTGCCAGGGCTGGTTTTTTTAT
query     ATCCGCCAGCGGCTGTGATACCGCCGGGACCGCGCCGCWGGCGAAAGATGAAWATTGYTC

template  ATATCCAATTTGCAAATGGTGAAATGATGGTGAAATAGCTATCGAAGGTCACTGCAAATT
query     CGGCTCTGGTTCAAGCAGAACACGCCCCTGCATAAACAGGGGCATCAGTTCCGMTCCCGG

template  GACTTCAAGCTCCCTCCAACGCGAAAAGTTGAACAGATGACTATAAATACATTGATTAGT
query     CCTGAACAATACAACGCCCATACGAGGAGCAATTTCAGGGGTCAGTAACGCTCTCACGAT

template  CATCCTTAACCAGTAGAATCCGCCACGACTGGTAACTATTCAATACACGCACTATCGAAC
query     CACCTCAATGAACGGTATCGAGCAGCTTCAGCAGCTCAGGGAATTTGGAYTCGAAGAAAT

template  GATCGCCAGTTTGCCGCAGCCCGTTCTTGCATACAATGTGGCTGCGGCACACCATCACAT
query     GCGGYTGCGTCTCGCGAGGGTTTGCCGGGCTGGTGATGTTTTTGCCGAACATGCAGCCTT

template  CAGAAATTTGAAAATCAGGGCTAAACCTGTCACTTAACCTGAAGCGGGTGGTGTATCGGG
query     TYGCCGTCAGCGACCAGAATTTTTTAATGCCGTTAATCGCGGAGCGACTGTAACGCTCAC

template  ATGACAGTATGTTTCTCTTCATAGCCCACTGTTGCTGGATACCCTCCCCGGCAAAGTACA
query     GATGTTCAACAACACCCAGCTTTGCTAACTGCTGATACGCCTGATTAGCCGTCATCCgga

template  GCTTTCCCCTGCCCTTCTTTGCATTCAGGTGATCCAGTACTTCCATCGGCTTCTCGCTAC
query     tatttcacCTGTACATCATCCGTACTGTCTGATGTTGTTCGTCCGGGAATATCTTTGTCG

template  CGGCGCGCGGCGCGTTATCATCGAAAAAGTTGCGCCGCGCCCTGACTGAAGAAGTCTCCC
query     CACCGCACCTCATTCACCTGGTGCATCAGTTACGACGCCACTGAACGTTCAGATAGCTCC

template  CTATTTCACCTGTACATCATCCGTACTGTCTGATGTTGTTCGTCCGGGAATATCTTTGTC
query     CCAGCATCTCCTCCACAAGAGGTCGCAGGCCATCCAAAAATGTCTTTTTCGCGCTCTCCA

template  GCACCGCACCTCATTCACCTGGTGCATCAGTTACGACGCCACTGAACGTTCAGATAGCTC
query     TCGCTCTTGCTCCGATAGTGCGCTCTGCATCGGTATTACCGACCAGCCCAGACGGTCTCA
```

```
template   CCCAGCATCTCCTCCACAAGAGGTCGCAGGCCATCCAAAAATGTCTTTTTCGCGCTCTCC
query      GCTCTGTGTCAGACAGCATCCGGTACGTTTCCTCATAATCCGATATTTGCTTTTCACGAA

template   ATCGCTCTTGCTCCGATAGTGCGCTCTGCATCGGTATTACCGACCAGCCCAGACGGTCTC
query      GCGCGTTAACGCGTTCCGGCGCTTTGCGCTCCAGCACTCTGTGTAACGGCCCCCACTGCA

template   AGCTCTGTGTCAGACAGCATCCGGTACGTTTCCTCATAATCCGATATTTGCTTTTCACGA
query      GTATCCACTCCCTGAACTCGCTTTTTTCAGCGGCTTTCACCTGAAGCTCCGCGTCCTGAA

template   AGCGCGTTAACGCGTTCCGGCGCTTTGCGCTCCAGCACTCTGTGTAACGGCCCCCACTGC
query      GGTCTGTAACCGTCACGCCGGATACGTCAAAGAAACGCATTTCTGACGTCACGCTGGTCA

template   AGTATCCACTCCCTGAACTCGCTTTTTTCAGCGGCTTTCACCTGAAGCTCCGCGTCCTGA
query      GCCCGAGTGATTTCTTCAGCTTATTCTGATACGCCAGCCAGACCTCAATTTCATCAACGA

template   AGGTCTGTAACCGTCACGCCGGATACGTCAAAGAAACGCATTTCTGACGTCACGCTGGTC
query      GAGCCAGCGTTCTGACCTTTTCCCGGGCAATCTGTTCCAGTTTTCCCAGACGGAACATCT

template   AGCCCGAGTGATTTCTTCAGCTTATTCTGATACGCCAGCCAGACCTCAATTTCATCAACG
query      CACGCCCCGTGGCAACCAGCGCCGCGAGATCGTTATCGTATTGCCCTTTTTCTGCATTAT

template   AGAGCCAGCGTTCTGACCTTTTCCCGGCAATCTGTTCCAGTTTTCCCAGACGGAACATCT
query      GTACCAGCTGTACGTTCTTCATCTGGTGCAAAAAAAATGTGACCCGGTCCTCGCAGCTTG

template   CACGCCCCGTGGCAACCAGCGCCGCGAGATCGTTATCGTATTGCCCTTTTTCTGCATTAT
query      AGGTTGCCTCTGTTGCCATAGCAAAGGTGTTTGCTCTTAACGCCTCATCTTCAGCCAGTT

template   GTACCAGCTGTACGTTCTTCATCTGGTGCAAAAAAAATGTGACCCGGTCCTCGCAGCTTG
query      GTGCCAGCCAGGACGATATCTGTGCCTTAAACCCCGCGTCCTTTATGAAGTTTTCCGTCT

template   AGGTTGCCTCTGTTGCCATAGCAAAGGTGTTTGCTCTCAACGCCTCATCTTCAGCCAGTT
query      CACTCAGTCTGTCCAGGAAGAGGCTGAATGCGTCGGCGTTATCTTCCTGTCCGAACATAT

template   GTGCCAGCCAGGACGATATCTGTGCCTTAAACCCCGCGTCCTTTATGAAGTTTTCCGTCT
query      GCCATCTGTCTGCAGGAGCCGGTTCCCCCTCCCGGGCAGGCACCAGCCAGTCAGCGGCCG

template   CACTCAGTCTGTCCAGGAAGAGGCTGAAGGCGTCGGCGTTATCTTCCTGTCCGAACATAT
query      CCAGGTGCAGTGCCCGAGTTTCCCGGGGGGCGGAGGCTCCCGCCATATCGAATCGTATTA

template   GCCATCTGTCTGCAGGAGCCGGTTCCCCCTCCCGGGCAGGCACCAGCCAGTCAGCGGCCG
query      TGGGGCCTGAATAGCCAGGCGCGCTGGTGATCTCCCGCAGCGCCTGCAAAGTACGTTCAG

template   CCAGGCGCAGTGCCCGAGTTTCCCGGGGGGCGGAGGCTCCCGCCATATCGAATCGTATTA
query      ACAGTGGGTTCCCTTCCAGATTTACGGTTGTCTCTGAAGACAGATGAATGAGACTTTCCG

template   TGGGGCCTGAATAGCCAGGCGCGCTGGTGATCTCCCGCAGCGCCTGCAAAGTACGTTCAG
query      GCAGGCGGGTCAGCTGGTTACGATAGACCGACAGCGACAGTAGTCCTGACGGCAGCATCG

template   ACAGTGGGTTCCCTTCCAGATTTACGGTTGTCTCTGAAGACAGATGAATGAGACTTTCCG
query      GCAGGCTGGTCAGCCGGTTACCAGATACCATCAGCTCCTTCAGTTCTGACGGCAGCACCG

template   GCAGGCGGGTCAGCTGGTTACGATAGACCGACAGCGACAGTAGTCCTGACGGCAGCATCG
query      GCAGACTGGTCAGCCGGTTACCAGATACAATCAGCTCCTTCAGTCCTGACGGCAACGCCG

template   GCAGGCTGGTCAGCCGGTTACCAGATACCATCAGCTCCTTCAGTTCTGACGGCAGCACCG
query      GCAGGCTGGTCAGCCGATTATTATAGGCCCACAGCTTATATAATTCTGACGGCAGCGTCG

template   GCAGACTGGTCAGCCGGTTACCAGATACAATCAGCTCCTTCAGTCCTGACGGCAACGCCG
query      GCAGGCTGGCCAGTTGGTTATCAGATACCGACAGCTCCTGTAGCCCTGACGGCAACATCG

template   GCAGGCTGGTCAGCCGATTATTATAGGCCCACAGCTTATATAATTCTGACGGCAGCGTCG
query      GCAGGCTGGTCAGCTGGTTATTATAGGCCCACAGCTTACATAATTCTGACGGCAGCGCCG

template   GCAGGCTGGCCAGTTGGTTATCAGATACCGACAGCTCCTGTAGCCCTGACGGCAACGTCG
query      GCAGGCTGGCCAGTTGGTTATCAGATACCGACAGCTCCTGTAGCCCTGGCGGCAACACCG

template   GCAGGCTGGTCAGCTGGTTATTATAGGCCCACAGCTTACATAATTCTGACGGCAGCGCCG
query      GCAGGCTGGTCAGTTGATTACCAAAGATCCACAGCTTACATAGTCCTGACGGCAGCGCCG

template   GCAGGCTGGCCAGTTGGTTA-T-CAGATACCGACAGCTCCTGTAGCCCTGGCGGCAACAC
```

```
query       GCAGGTGGGTCAGCGGGTTACTAAAGAT--CGACAGTTCCAGTAGTCCTGGCGGCAGCAC

template    CGGCAGGCTGGTCAGCTGGTTATTATAGGCCCACAGCTTACATAGTCCTGACGGCAGCGC
query       CGGCAGGCTAGTCAGCTGGTTACCAGAGACCTCCAGCGTCCGCAGTTCTGGCGGCAGCGC

template    CGGCAGGCTGGTCAGATTATTATCAGGAATAACCAGTGTGGTAATATGCGCGGGTAAACA
query       CGGCAGGCTGGTCAGATTATTATCAGGAATAACCAGTGTGGTAATATGCGCGGGTAAACA

template    GTCTGGCAAGGTGGTAAGACCTGATTCTCCCACGTTAAGCACTGCATTGCCATTATTCAG
query       GTCTGGCAAGGTGGTAAGACCTGATTCTCCCACGTTAAGCACTGCATTGCCATTATTCAG

template    GCAGGCACGCATTTTCTGTACCGCTGCTGCACGGCCGCGTGACTCTTCTGCGGGTGCAGC
query       GCAGGCACGCATTTTCTGTACCACTGCTGCACGGCCGCGTGACTCTTCTGCGGGTGCAGC

template    CCTTCTCCACGCTGACCAGACAACATCATACTCTGCTGGCGTCTGTGGAGCCGTTGTGGC
query       CCTTCTCCACGCTGACCAGACAGCATCATACTCTGCTGGCGTCTGTGGAGCCGTTGTGGC

template    AGGGAGGAAGGATGTCCTGAATGTCCCGGATGCCCCTTCCGCGTGTCCTGTGCCCTCCTC
query       AGGGAGGAAGGATGTCCTGAATGTCCCGGATGCCCCTTCCGCGTGTCCTGTGCCCTCCTC

template    ACCCTGTGCTGTGTCCAGGGTGAGGCGATGGGTTTCACTGTACCCCTGGCAATTTACGGT
query       ACCCTGTGCTGTGTCCAGGGTGAGGCGATGTGTTTCACTGTACCCCTGGCAATTTACGGT

template    ATAGTTCCCGACATCATCAAGGGTGACTGACAACATCTCCTGACTGTCTTCATCCAGAAT
query       ATAGTTCCCGGCATCATCAAGGGTGACTGACAATATCTCCTGACTGTCTTCATCCAGAAT

template    ACAGAAGTAGTTTTCCCCGTGCAGGCCGGAATGAATGTTTTCCTCCCATCCGTCATACGC
query       ACAGAAGTAATTTTCCCCGTGCTGGCCGGAATGAATGCTTTCCTCCCATCCGGCATACGC

template    GAGCGTCCTGAGCAGTTCAAATCTGCTGACCACATCCTCCCGCGTCGTTCCGGCCGGCGG
query       GAGCGTCCTGAGCAGTTCAAATCTGTTGATCACATCCTCCCGCGTCGTTCCGGCCGGCGG

template    GTGACAAATCGCCCAGATGCACTCCAGCGCTTCAGTCTGGTGCGTTGAGCAAAAAAATTC
query       GTGACAAATCGTCCAGATGCACTCCAGCGCTTCAGTCTGGTGCGTTGAGCAAAAAAATTC

template    CTTCATTTTTTCCCAGGAACTCATTTCAGGGGGGGGTATCAGACCAGGCAATACGATAAA
query       CTTCATTTTTTCCCAGGAACTCATTTCA-GGGGGGGTATCAGACCAGGCAATACGATAAA

template    TGCGACGGTTACTGATGGTGGCGGGAAGACATCCGCTTCCAATATGAAAGGGCATAACAA
query       TGCGGCGATTACTGATGGTGGCGGGAAGACATCCGCTTCCAATATGAAAGGGCATAACAA

template    AAAACCTTTATAAATTTACATATAGTATCTGTCCGGCAGACATCATCTCTTCCTGGTCTT
query       AAAACCTTTATAAATTTACATATAGTATCTGTCCGGCAGACATCATCTCTTCCTGGTCTT

template    AATTTCACAATAAGGTTATCGGCGGATTCATGGTCGTCCTGCCATGGCGGGCTTCAGAAG
query       AATTTCACAATAAGGTTATCGGCGGATTCATGGTCGTCCTGACATGGCGGGCTTCAGAAG

template    GTGCAGAAGAAAAATCCGTTATGATGACCGGATGGCGGGACTGTCATTTTACAGCTAAAG
query       GTGCAGAAGAAAAATCCGTTATGATGACCGGATGGCGGGACTGTCATTTTACAGCTAAAG

template    TGTCGA-TTTTTCAGGGTCGCTTTCCACGATGACCAGATCATCCGGTATCAACGCCCGGG
query       TGTCGATTTTTTCAGGGTCGCTTTCCACGATGACCAGATCATCCGGTATCAACGCCCGGG

template    CGTTCAATTTTCGATTCTGCCTGCCTGCGCAGCTCTTCCGGCGAATAAACCCTTTGAACG
query       CGTTCAATTTTCGATTCTGCCTGCCTGCGCAGCTCTTCCGGCGAATAAACCCTTTGAACG

template    ACTTTACTGTCTTTAAACTGCCAGCAGCATGAAATATCAGCGCGACGGTTTTCATCCGTA
query       ACTTTACCGTCTTTAAACTGCCAGCAGCATG-AATATCAGCGCGACGGTTTTCATCCGTA

template    TCAGGCAACTCTGCAACGCTCTGGCCAGCAGGCCACAGCCCGGAAACATCATGCGTAATA
query       TCAGGCAACTCTGCAACGCTCTGGCCAGCAGGCCACAGCCCGGAAACATCACGCGTAATA

template    CACGTAATAACATCGTTATCGTCGTAGGTAATTTTCAGCGTGTCTGCTGAAAATAATTGC
query       CACGTAATAACATCGTTATCGTCGTAGGTAATTTTCAGCGTGTCTGCTGAAAATAATTGC

template    TGGCACCCATACCAGTCCTGACCATCCTCTGATTTAAGATACGCCGCATCGGCGAATAAT
query       TGGCACCCATACCAGTCCTGACCATCCTCTGATTTAAGATACGCCGCATCGGCGAATAAT

template    GTGTCATCTGGTTCCGGTGCATACGGAATGAAATTTTTAATATTTAAAAACTGTTTGTTC
query       GTGTCATCTGGTTCCGGTGCATACGGAATGAAATTTTTAATATTTAAAAACTGTTTGTTC
```

```
template    TTTTTA-TTTTTTTCCGCTGTCATAATTATTCCTTCTTATACACTTGGGGCTGTCACCCA
query       TTTTTATTTTTTTTCCGCTGTCATAATTATTCCTTCTTATACACTTGGGGCTGTCACCCA

template    CGTATCACCAACCAGATACTGAATGGGACGTTGTCACCCGAATAGTCATCGCGCCACGCA
query       CGTATCACCAACCAGATACTGAATGGGACGTTGTCACCCGAATAGTCATCGCGCCACGCA

template    CTCCCATTACCTCAATCACTGCGCCAGCGAGACAGATATTACCGCAGCCAGTATCCGTGA
query       CTCCCATTACCTCAATCACTGCGCCAGCGAGACAGATATTACCGCAGCCAGTATCCGTGA

template    TGATTTTGTTATAAGCGTAAGACCAGGAGCCACGACACATCCAGTAAGGGTGATTGAATG
query       TGATTTTGTTATAAGCGTAAGACCAAGAGCCACGACACATCCAGTAAGGGTGATTGAATG

template    CGCCCTGAGACTCCAGCCAGACGATAAACTCCGCCGTCGTCCACGGATTATCATCACAGC
query       CGCCCTGAGACTCCAGCCAGACGATAAACTCCGCCGTCGTCCACGGATTATCATCACAGC

template    CGATAGTAACTGCGCCATCAAACGCTCGCGCGGCACCGATACGACGCACAAACAAATCCT
query       CGATAATAACTGCGCCATCAAACGCTCGCGCGGCACCGATACGACGCACAAACAAATCCT

template    TGTCCGGAATATCGCCGCCATTCTGTGATTTTTGTAATGCATCGGCGGCACGACTTATGG
query       TGTCCGGAATATCGCCGCCATTCTGTGATTTTTGTAATGCATCGGCGGCACGACTTATGG

template    TTTCTCGCAAACCAACGTATTCGATAAGACCGTCAACGCTTTTTCCTGACAGCGCCGTCA
query       TTTCTCGCAAACCAACGTATTCGATAAGACCGTCAACACTTTTTCCTGACAGCGCCGTCA

template    GGGTTTCGTCCAGCGGCTGCTTGCCCGCCAGTTTATTCAGTACCGTGGTGGCAAAGTTCG
query       GGGTTTCGTCCAGCGGCTGCTTGCCCGCCAGTTTATTCAGTACCGTGGTGGCAAAGTTCG

template    GATCATTTCCCAACACGTCAGCCAGTTCCTGCAGGGTGTCCAGTGGCTCCAGTACGGAAC
query       GATCATTTCCCAACGCGTCAGCCAGTTCCTGCAGGGTGTCCAGTGGCTCCAGTACGGAAC

template    CAACCAGCGCGGCAATCAGTTTGCGGACAAACTCCGCGTTTGTTGTCTGAAGCCCTTTAG
query       CAACCAGCGCGGCAATCAGTTTGCGGACAAACTCCGCGTTTGTTGTCTGAAGCCCTTTAG

template    CATCGCCTAGCGGCGTCGGTGTGGTCGGCGTTCCAGTGAATGCCGGGCTGTCCAGCGGCG
query       CATCGCCTGGCGGCGTCGGTGTGGTCGGCGTTCCAGTGAATGCCGGGCTGTCCAGCGGCG

template    CTTTGGTCCGTACCTCACCCATAACGGTTTTTACCGCCTTTGGTGTGGCCGCCAGCGCTT
query       CTTTGGTCCGTACCTCACCCATAACGGTTTTTACCGCCTTTGGTGTGGCCGCCAGCGCTT

template    CGCTGTCACTGTCCGTGGCACTGCTTAATTTAACGATACCTTTTTTCGTCAGGCTTGCAT
query       CGCTGTCACTGTCCGTGGCACTGCTTAATTTAACGATACCTTTTTTCGTCAGGCTTGCAT

template    CTTCCAGGGAAATCACGTCTGCGATATCCTCGGCCCGTTTCGCGGCCTCTTCGGCTCTGG
query       CTTCCAGGGAAATCACGTCTGCGATATCCTCGGCCCGTTTCGCGGCCTCTTCGGCTCAGG

template    TTGCCGATTCTCCGGCTGCAGCTCTGCTTTGCGCTGCCAGTGATGCGATGGTATCAGATG
query       TTGCCAATTCTCCGGCTGCAGCTCTGCTTTGCGCTGCCAGTGATGCGCTGGTATCAGATG

template    CGGCGGCGTGGGTGGATGCCTACGATGCTGATGACGAGGCGGCTGCCGCCGTTCTGGCTG
query       CGGCGGCGTGGGTGGATGCCTCCGATGCTGATGACTAGGCGGCTGCCGCCGTTCTGGCTG

template    TGTCAGCCGACGCCGCGCTGGCTGATGCCTCCCCGGCTTTTGTGGTCGCCGTACCTGCGC
query       TGTCAGCCGACGCCGCGCTGGCTGATGCCTCCCCGGCTTTTGTGGTCGCCGTACCTGCGC

template    TGCTCTCCGCAGATGCTGCGGATGAAGCTGCCGACCAAAATTCCCCTTGAAAACCAGCCT
query       TGCTCTCCGCAGATGCTGCGGATGAAGCTGCCGACCAAAATTCCCCTTGAAAACCAGCCT

template    GTCAGGGCTGGTTTTTTTATGCCTACAATTAAAAAGTTAGCTGATGCCAGAGACGTTATT
query       GTCAGGGCTGGTTTTTTTATGCCTACAATTAAAAAGTTAGCTGATGCCAGAGACGTTATT

template    TTGCATGAACTGATTTTGCATGAACTGATTGGACATCTTTCCTGTGCAGTGGTCACAGTA
query       TTGCATGAACTGATTTTGCATGAACTGATTGGACATCTTTCCTGTGCAGTGGTCACAGTA

template    AAGTCAATGCAGGCAACTAACGTAAACCATGTTTGTACTAAAACAGAAAAAGCGCATCTA
query       AAGTCAATGCAGGCAACTAACGTAAACCATGTTTGTACTAAAACAGAAAAAGCGCATCTA

template    CACCCTATACTGGAAAAAATGTTTGCAGTGGCAGGTAAATGAGTAAGCAATCTGTCGCAG
query       CACCCTATACTGGAAAAAATGTTTGCAGTGGCAGGTAAATGAGTAAGCAATCTGTCGCAG
```

```
template    CAATAATAAAACGTTCTGGTAAGGTAAACGGCCTACCACGCCTTTGTCGCTAACAATTAG
query       CAATAATAAAACGTTCTGGTAAGGTAAACGGCCTACCACGCCTTTGTCGCTAACAATTAG

template    CCTCCCTGTCGCCATAGGGTCGATAACGATCGGCTATTCACAACACGTTTTGTAGACAAC
query       CCTCCCTGTCGCCATAGGGTCGATAACGATCGGCTATTCACAACACGTTTTGTAGACAAC

template    GTACGGTGATTAATTACTCCTTCAGCCAGCAACTCGCTAATTGTTATTCAACTTCAGAAA
query       GTACGGTGATTAATTACTCCTTCAGCCAGCAACTCGCTAATTGTTATTCAACTTCAGAAA

template    TTATAACTAATTGAAGCACCTACAAAATTATGCCCTGAATTTTTATCCGTAAGTGATCCA
query       TTATAACTAATTGAAGCACCTACAAAATTATGCCCTGAATTTTTATCCGTAAGTGATCCA

template    TTCGAGAAATGCCGGATATAAGCTTCTGTAGAAAAAGCCCCAAAGTTCCAGCCAAGAAAC
query       TTCGAGAAATGCCGGATATAAGCTTCTGTAGAAAAAGCCCCAAAGTTCCAGCCAAGAAAC

template    GCTTTTTCTCCAAAGGTAAATGCAGAATTGACACGCATATCATCTCGCGACTTAGATTTT
query       GCTTTTTCTCCAAAGGTAAATGCAGAATTGACACGCATATCATCTCGCGACTTAGATTTT

template    ATATAAGCCCCCAGACCAATACCGGTATAGATACCAGCGAAATGAACCAGCGCCACATCC
query       ATATAAGCCCCCAGACCAATACCGGTATAGATACCAGCGAAATGAACCAGCGCCACATCC

template    TTCGAGATACCCAATACGCCCTGGTTATATTTATTGCAGGGAACGCTGCCAGAATGTTTA
query       TTCGAGATACCCAATACGCCCTGGTTATATTTATTACAGGGAACGCTGCCAGAATGTTTA

template    CTGCAATCATCGCTACCCTTAGCCTTAAATCCTCCTAACTCCAGATTATTTCTTGCCTGT
query       CTGCAATCATCGCTACCCTTAGCCTTAAATCCTCCTAACTCCAGATTATTTCTTGCCTGT

template    AAAAAGAAAAAATCGCTGGGTTCACTGTATGTCAGAAAAGCCGTATATAAGTGCTCAACC
query       AAAAAGAAAAAATCGCTGGGTTCACTGTATGTCAGAAAAGCCGTATATAAGTGCTCAACC

template    CCTCCTCTTCTTATACTTTCTCCCGCAGCAAAAGAGATCTGATGCTTGTAGCCCTGGCCA
query       CCTCCTCTTCTTATACTTTCTCCCGCAGCAAAAGAGATCTGATGCTTGTTGCCCTTGCCA

template    AAAAAAACATTATCATTAGCAGAACATGCGAATGCACAAGGTAATAATAGATATATAAAT
query       AAAAAAACATTATCATTAGCAGAACATGCGAATGCACAAGGTAATAATAGATATATAAAT

template    ATTCTCTTCATATACACTCCACCACCATTTCAATGTCAATAGTTATTTTTAACCGGCTAA
query       ATTCTCTTCATATACACTCCACCACCATTTCAATGTCAATAGTTATTTTTAACCGGCTAA

template    CATATTTAAAATTTTCTACGCCACGCTTACGCTTACAGAAACGACTGATATAGCATCCAC
query       CATATTTAAAATTTTCTACGCCACGCTTACGCTCACAGAAACGGCTGATATAGCATCCAC

template    ACTACGTTCTCCCCTTGAGTCGCCCCAACGGGTGCTCAAAACGCCGCGAGTGTTGTTCAG
query       ACTACGTTCTCCCCTTGAGTCGCCCCAACGGGTGCTCAAAACGCCGCGAGTGTTGTTCAG

template    GCACATCCAGGATAAGTTATAAGTGAATGACACTCATTTCTGACCACATTTTCATTTATG
query       GCACATCCAGGATAAGTTATAAGTGAATGACACTCATTTCTGACCACATTTTCATTTATG

template    ATAAGAATATCGTCAGAATCTCTTTATCGCATAAAAATACCTCTGCTATTGACTTAATAT
query       ATAAGAATATCGTCAGAATCTCTTTATCGCATAAAAATACCTCTGCTATTGACTTAATAT

template    TTAATTTAACAGATGTCGCGGTTCATTCGTTCTGCTCAGATGTATTTCATTGACATTAAT
query       TTAATTTAACAGATGTCGCGGTTTATTCGTTCTGCTCAGATGTATTTCATTGACATTAAT

template    AAAACCATAAATAAATTCAGTTAAAATAATTTGTTAATAATGACAAGCTTTAGACGCTAT
query       AAAACCATAAATAAATTCAGTTAAAATAATTTGTTAATAATGACAAGCTTTAGACGCTAT

template    GACTTTTAATAAGGAATTTCCTGCCAGTAAAGATAGTCTGGACTTACGGGTGCAAGGGCG
query       GACTTTTAATAAGAAATTTCCTGCCAGTAAAGATAGTCTGGACTTACGGGTGCAAGGGCG

template    CAGCAACATATGCCACACTTCTGTTATGTCACATTGTCCGAAAGAAGCGTCCAGACCAGT
query       CAGCAACATATGCCACACTTCTGTTATGTCACATTGTCCGAAAAAAGCGTCCAGACCAGT

template    ATTCAACTTCAGGGAGTATTCTGTTTTCTCTCACAACGGGTTGTTTTATCCTTTCTATCG
query       ATTCAACTTCAGGGAGTATTCTGTTTTCTCTCACAACGGGTTGTTTTATCCTTTCTATCG

template    TTCAGCTAATTACTCGCCTGTCACAGGTTTTACTACTTACCCATGCACTAATTAGGGTAA
query       TTCAGCTAATTACTCGCCTGTCACAGGTTTTACTACTTACCCATGCACTAATTAGGGTAA

template    AATTTACGCGCTTGACAATGTGGCAAATCTGGACGATTTCCGCGCTGAAAACAATAATCA
```

```
query       AATTTACGCGCTTGACAATGTGGCAAATCTGGACGATTTCCGCGCTGAAAACAATAATCA

template    TTCTAATAAAACTCAGGAGATACAGCATGCCTGAAGTAACACCTTTTCAGGTGCTTATTG
query       TTCTAATAAAACTCAGGAGATACAGCATGCCTGAAGTAACACCTTTTCAGGTGCTTATTG

template    TGGACGACCATCCACTTATGCGGCGAGGTATTCGTCAATTACTGGAACTGGACCCGGCTT
query       TGGACGACCATCCACTTATGCGGCGAGGTATTCGTCAATTACTGGAACTGGACCCGGCTT

template    TTCATGTCGTTGCCGAAGCGGGTGACGGGGCGAGTGCGATCGATCTGGCGAATCGCATTG
query       TTCATGTCGTTGCCGAAGCGGGTGACGGGGCGAGTGCGATCGATCTGGCGAATCGCATTG

template    AGCCCGATCTGATCCTGCTGGATCTGAATATGAAAGGTCTGAGCGGGTTAGATACCCTGA
query       AGCCCGATCTGATCCTGCTGGATCTGAATATGAAAGGTCTGAGCGGGTTAGATACCCTGA

template    ACGCGCTACGCCGGGATGGTGTGACGGCGCAAATTATTATTCTTACCGTCTCGGATTCCG
query       ACGCGCTACGCCGGGATGGTGTGACGGCGCAAATTATTATTCTTACCGTCTCGGATTCCG

template    CCAGCGACATCTACGCGCTGATCGACGCCGGCGCCGACGGTTATCTGCTCAAAGATAGCG
query       CCAGCGACATCTACGCGCTGATCGACGCCGGCGCCGACGGTTATCTGCTCAAAGATAGCG

template    ATCCGGAAGTGCTGCTGGAGGCCATTCGCAAAGGCGCTAACGGCGGTAAAGTCTTTAGCG
query       ATCCGGAAGTGCTGCTGGAGGCCATTCGCAAAGGCGCTAACGGCGGTAAAGTCTTTAGCG

template    ATCGGGTCAATGAATATCTGCGTGAGCGCGAACGGTTTGGCGCGCAGGAAGATCCCTTTA
query       ATCGGGTCAATGAATATCTGCGTGAGCGCGAACGGTTTGGCGCGCAGGAAGATCCCTTTA

template    GTATCCTGACCGAACGAGAGTTAGATGTCTTGCATGAGCTGGCGCAGGGGCTTTCTAACA
query       GTATCCTGACCGAACGAGAGTTAGATGTCTTGCATGAGCTGGCGCAGGGGCTTTCTAACA

template    AACAGATTGCGTCAGTACTGAATATTTCTGAGCAGACGGTTAAGGTGCACATTCGTAATC
query       AACAAATTGCGTCAGTACTGAATATTTCTGAGCAGACGGTTAAGGTGCACATTCGTAATC

template    TACTTCGCAAGCTAAACGTGCGCTCACGCGTCGCGGCGACCATTCTATTTTTACAAACAC
query       TACTTCGCAAGCTAAACGTGCGCTCGCGCGTCGCGGCGACCATTCTATTTTTACAAACAC

template    GCGGAATGCAGTAACAAGCCAGGCCCGATGGCGCCACGCCACTGGGCCGTCAGGGCATTA
query       GCGGAATGCAGTAACAAGCCAGGCCCGGTGGCGCCGCGCCACCGGGCCGTCAGGGTCTTA

template    TTTCTCCTGCGCCAGCCGGATACTGCGCTCTATCACCGCCCGCCGGGCGTCACCCGCCGG
query       TTTCTCCTGCGCCAGCCGGATACTGCGCTCTATCACCGCCCGCCGGGCGTCACCCGCCGG

template    CAGCAGCTTCAGCATCATCTCCCAGGCCGCCACCGCCTCGCCAAAGCGCTGCTGCTCAAA
query       CAGTAACTTCAGCATCATCTCCCAGGCCGCCACCGCCTCGCCAAAGCGCTGCTGCTCAAA

template    GGCGTTAAACGCATACAGGCTTAACACCCGGATATCCGTGTGGTCGCGGCTCACCAGCTG
query       GGCGCTGAACGCATACAGGCTTAACACCCGGATATCCGTGTGGTCGCGGCTCACCAGCTG

template    ACGCAGCAGCTCCCCGCCGCAGCGGTTATCCTCCGGGTCGGACGAGCGCGTCAGCGCCTC
query       ACGCAGCAGCTCCCCGCCGCGGCGGTTATCCTCCGGGTCGGACGAGCGCGTCAGCGCCTC

template    CGCGTAGCCCAGCGCCGCATCGCGGTTTTTCGGGTCCAGGTGGTAGGCGTTCGCATACGC
query       CGCGTAGCCCAGCGCCGCATCGCGGTTTTTCGGGTCCAGACGGTAAGCGTTTGCATAGGC

template    CCCGGTGGCGGTACCGGCATTACCCAGTACCATACCGGTGCGCCCCAGCATGAGCCAGCC
query       CCCGGTGGCGGTACCGGCATTACCCAGTACCATACCGGTGCGCCCCAGCATGAGCCAGCC

template    CTCAACATTGCCGGCATCATTTTGCAGGCGGGTGCGCAGCCCCAGCGCCAGCCGCGCCAT
query       CTCAACATTGCCGGCATCATTCTGCAGGCGGGTGCGCAGCCCCAGCGCCAGCCGCGCCAT

template    CTCCTCCTCATTCAGCGGCTGCGCCTGCGGGTCCAGCGCCCGCGCCAGCAGTCCCGGCGT
query       CTCCTCCTCATTCAGCGGCTGCGCCTGCGGGTCCAGCGCCCGCGCCAGCAGTCCCGGCGT

template    CTGCGCCGTTGCCTGCTGCCAGGCCCTCACCTGCGGATAGCTGCCGGTCAGGGCGTAGCT
query       CTGCGCCGTTGCCTGCTGCCAGGCCCTCACCTGCGGATAGCTGCCGGTCTGTGAGTAACT

template    GCCGGCGCCGACCGCCAGCGCAATGACGGCCCCCGGCACGTAAACGCCCCACCCGGCGCG
query       GATGGCGGCCACGACCAGCGCCATAACCACGCCCGGCACGTAAGCACCCCACCCGGCGCG

template    CGCGCC-GCAAACCGGGGGTCCGCCGGC-AGCGGC-TCCCGGCGCAGGCGCACCCGCCG
query       CGGTCCCGCCG-CAGGAATGGC-GTCGGCCAG-GACGTCCTGTCTGATACGCACCCGCCG
```

```
template    GCGCGTGCGGGCGACGATTATCCACCCGCCCGCCACGATGGCGGCCAGCGGCAGCACCCA
query       GCGCGTGCGGGCGACGATTATCCACCCGCCCGCCACGATGGTGGCCAGCGGCAGCACCCA

template    CAGCAGCACCGTCAGCGGGGTCAGCGGCGGGTCGTAGGTGACGAAGTTGCCGTAGCGCGC
query       CAGCAGCACCGTCAGCGGGGTCAGCGGCGGGTCGTAGGTGACAAAGTTGCCGTAGCGCGC

template    CACCATATAATCGATGATTTCCTGGCGGCTCTTCCCCTCCTGCATCAGGTCATACACCCT
query       CACCATGTAATCGATGATTTCCTGGCGGCTCTTCCCCTCCTGCATCAGGTCATACACCCT

template    GCGACGCATGTCGGTGGCTATCATCGCGTTCGAGTCCGCAATGCTGTTGTTCTGGCATTT
query       GCGGCGCATGTCGGTGGCTATCATCGCGTTCGAGTCCGCAATGCTGTTGTTCTGGCATTT

template    CGGGCAGCGCAGCTGCTCCGTGAGCTGGCGGAACTGCTGCTCCTGCGCTTCATCTTTAAA
query       CGGGCAGCGCAGCTGCTCCGTGAGCTGGCGGAACTGCTGCTCCTGCGCTTCATCTTTAAA

template    CGGCATCACGTCGGTGGTTGCCCGCGCTGACCCGGCGATAACCAGCGCCAGCATCAGCAT
query       CGGCATCACGTCGGTGGTCGCCCGCGCTGACCCGGCGATAACCAGCACCAGCATCAGCAT

template    CACCATGCCCGGTAACAGTCTCATTGCGCCGCCTCCCGGCTGTATCTGTCCCACAGCGGT
query       CACCATGCCCGGTAACAGTCTCATTGCGCCGCCTCCCGGCTGTATCTGTCCCACAGCGGT

template    TTCAGTTCACTTTCCCATACCCGGGCATTCAAATCGCCCGCATGGCGGTAGCGGATAATC
query       TTCAGTTCACTTTCCCATACCCGGGCATTCAAATCGCCCGCATGGCGGTAGCGGATAATC

template    CCGTTGCCGTCGATGAGGAAGGTTTCCGGCGCGCCGTACACGCCCAGGTCCAGCCCCAGC
query       CCCCTGCCGTCGATGAGGAAGGTTTCCGGCGCGCCGTACACGCCCAGGTCCAGCCCCAGC

template    ATCCCGTCGCTGTCCGATAAACTCAGCGCATACGGGTTGCCCAGTTCTTTTAACCAGGCC
query       ATCCCGTCGCTGTCCGATAAACTCAGCGCATACGGGTTGCCCAGTTCCTTTAACCAGGCC

template    ACCGCCTTCGCCCGGTCGTCCTTATAGTTCAGCCCGACCACCCGGATACCCTGCGCAGAA
query       ACCGCCTTCGCCCGGTCGTCCTTATAGTTCAGCCCCACCACCCGGATACCCTGCGCAGAA

template    AGCTGGTTCAGGTACTGATGCTCGGCGCGGCAGGTCGGGCACCAGGTGGCCCAGACGTTA
query       AGCTGGTTCAGGTACTGATGCTCGGCGCGGCAGGTCGGGCACCAGGTGGCCCAGACGTTA

template    AGCAGCACCGGTTTCCCCTGCGTCAGCACCTCCGCCTGATAGTACTGACCCGGCGTCTCC
query       AGCAGCACCGGTTTCCCCTGCGTCAGCACCTCCGCCTGATAGTACTGACCCGGCGTCTCC

template    AGCGATTCCAGGCGGAACACCGGCACCGGCTTTCCGGTCAGCGCCGATTCGAGATTCGTC
query       AGCGATTCCAGGCGGAACGCCGGCACCGGCTTTCCGGTCAGTGCCGATTCGAGATTCGTC

template    GGGTCATCCCCCTGCGCGTTGCGCGCCAGCTGCCACAGCAGCGCCGCGGCAATCAGCAGA
query       GGGTCATCCCCCTGCGCGTTGCGCGCCAGCTGCCACAGCAGCGCCGCGGCAATCAGCAGA

template    AAAATCAGCAGCGGTAATAACAGTACGTTGCGTTTCATCCGGCCTCCGGCAATGGTTTAC
query       AAAATCAGCAGCGGTAATAACAGTACGTTGCGTTTCATCCGGCCTCCGGCAATGGTTTAC

template    GGCGGCGGTAGCGCGGGTCCGCCAGGCACAGCAGCCCGCCCAGCGCCATCAGCAGTCCCC
query       GGCGGCGGTAGCGCGGGTCCGCCAGGCACAGCAGCCCGCCCAGCGCCATCAGCAGTCCCC

template    CGGCCCATATCCAGCGGACAAACGGTTTGTAGTACAGGCGCACGGCCCACGCGCCGTTGT
query       CGGCCCATATCCAGCGGACAAACGGTTTGTAGTACAGGCGCACGGCCCACGCGCCGTTGT

template    CCAGCTCCTCCCCGAGCGCGGCGTACAGGTCGCGGGTCAGCCCGCCGTCAATCGCCGCCT
query       CCAGCTCCTCCCCGAGCGCGGCGTACAGGTCGCGGGTCAGCCCGCCGTCAATCGCCGCCT

template    CGGTCATCACCATCCGGCTGGTGTTGTAGAGCCGTTTCTCCGCGTGCAGCACCGCCTCCG
query       CGGTCATCACCATCCGGCTGGTGTTGTAGAGCCGTTTCTCCGCGTGCAGCACCGCCTCCG

template    GCTCGCCGTGGCGCGTCACCCCGATGAGGGCCACCCCGCCGCGGTAGTTGGGGCCGGTGA
query       GCTCGCCGTGGCGCGTCACCCCGATGAGGGCCACCCCGCCGCGGTAGTTGGGCCCGGTGA

template    TGTCCCGCACCTCCCGGAAGGTGAAGCGGTAGTCGTGAATGGTCACGCTGTCGCCCGCCC
query       TGTCCCGCACCTCCCGGAAGGTGAAGCGGTAGTCGTGAATGGTCACGCTGTCGCCCGCCC

template    GCATCCGCACGTCACGCTCCACGCTGTAATTCTGGCTGAAGGCGATGCCGGTAATCGTCA
query       GCATCCGCACGTCACGCTCCACGCTGTAATTCTGGCTGAAGGCGATGCCGGTAATCGTCA
```

```
template   CCGCCAGCCCGAGGTGCGCCGCCACCATTCCCCAGTAGCTGAGAGAGGTTTTCGTGCCGC
query      CCGCCAGCCCGAGGTGCGCCGCCACCATTCCCCAGTAGCTGAGAGAGGTTTTCGTGCCGC

template   GGGACACGCGCTGTACGGCTTCGGCCACCGCCAGCACCGCAATCCAGCAGGCCATCGCCA
query      GGGACACGCGCTGTACGGCTTCGGCCACCGCCAGCACCGCAATCCAGCAGGCCATCGCCA

template   TCCCCACCGCCGTCATGGCGATGATTTTATCTTCCAGCAGCCACGGCAAAAGTACCGACA
query      TCCCCACCGCCGTCATGGCGATGATTTTATCTTCCAGCAGCCATGGCAAAAGTACCGACA

template   GCACCAGGGTGGTGACAACGGCAGCCCACAGCAGTTTCCTGATGTTACGCGGCCGGTCCC
query      GCACCAGGGTGGAGACCAGGGCGGTGAGCAGCAGCGTCCTGATGTTACGCGGCCGGTCCC

template   GGCCCCAGCGCACCAGCGGCCCCACCCCCAGCAGCAGGGCAAAGGGGACCATCAGCCAGG
query      GGCCCCAGCGCACCAGCGGCCCCACCCCCAGCAGCAGGGCAAAGGGGACCATCAGCCAGG

template   TGAACATGGTGTTAAAGAACGGCTCCCCCACCGAAATGCTGCCCAGCCCCAGCTGTTTGT
query      TGAACATGGTGTTAAAGAACGGCTCCCCCACCGAAATGCTGCCCAGCCCCAGCTGTTTGT

template   GCACCAGCGGCAGCAGGGTGCCCAGCAGCACCACCAGCATGGCGGCCATCAGCAGGACGT
query      GCACCAGCGGCAGCAGGGTGCCCAGCAGCACCACCAGCATGGCGGCCATCAGCAGGACGT

template   TGTTGCCGAGCAGCAGCGACTCGCGCGACCACAGCGCGTTGTTCACCCGCGAACGCACCC
query      TGTTGCCGAGCAGCAGCGACTCGCGCGACCACAGCGCGTTGTTCACCCGCGAACGCACCC

template   TGTGCCCGCGCACGGCGAACAGCAGCAGCGAGCCGCCGGTGACCAGCACCATAAAGGCGA
query      TGTGCCCGCGCACGGCGAACAGCAGCAGCGAGCCGCCGGTGACCAGCACCATAAAGGCGA

template   GGATAAACATTCCGCGCGCCGGGTCGGAGGCGAAGGCGTGCACCGACACCAGCACCCCGG
query      GGATAAACATCCCGCGCGCCGGGTCGGAGGCGAAGGCGTGCACCGACACCAGCACCCCGG

template   AGCGCACCAGGAAGGTGCCCAGCAGGCACAGCGAGAAGGCGCAGATGGACAGCAGCAGCG
query      AGCGCACCAGGAAGGTGCCCAGCAGGCACAGCGAGAAGGCGCAGATGGACAGCAGCAGCG

template   TCCACGCCTTAAAGCCGGCGCGCTGTTCCGTGACCGCCATCGAGTGCAGCAGGGCGGTGC
query      TCCACGCCTTAAAGCCGGCGCGCTGTTCGGTGACCGCCAGCGAGTGCAGCAGGGCGGTGC

template   CCGCCAGCCACGGCATAAAGGAGGCGTTCTCCACCGGGTCCCAGAACCACCAGCCGCCCC
query      CCGCCAGCCACGGCATAAAGGAGGCGTTCTCCACCGGGTCCCAGAACCACCAGCCGCCCC

template   AGCCCAGCTCGTAGTAGGCCCACGCCGAGCCGAGCACGATGCCCAGCGTCAGGAACACCC
query      AGCCCAGCTCGTAGTAGGCCCACGCCGAGCCGAGCACGATGCCCAGCGTCAGGAACACCC

template   ACGCCGCCAGCGTCCACGGGCGGGCAAAACGGGTGAACGCGCTGTCCAGACGCCCGCTCA
query      ACGCCGCCAGCGTCCACGGGCGGGCAAAACGGGTGAACGCGCTGTCCAGACGCCCGCTCA

template   GCAGCGCGGCGATGGCGAAGGCGAAGGCCACCGAGAAGCCGACATAGCCCATGTACAGCA
query      GCAGCGCGGCGATGGCGAAGGCGAAGGCCACCGAGAAGCCGACATAGCCCATGTACAGCA

template   GCGGCGGGTGGAAAATCAGCCCCGGGTCCTGCAGCAGCGGGTTCAGGTCGCGCCCCTCCA
query      GCGGCGGGTGGAAAATCAGCCCCGGGTCCTGCAGCAGCGGGTTCAGGTCGCGCCCCTCCA

template   CCGGAAAGGCCGGCAGCGTGCGGGCGAACGGGCCGGAGGTGAACAGGATGAACGCCAGAA
query      CCGGAAAGGCCGGCAGCGTGCGGGCGAACGGGCCGGAGGTGAACAGGATGAACGCCAGAA

template   AACCGGCGCAGACCATCCCCATCACCGCCAGCACCCGGGCGACGATATCCGCCGGCACCT
query      AACCGGCGCAGACCATCCCCATCACCGCCAGCACCCGGGCGACGATATCCGCCGGCACCT

template   GCCGGCTGAACACCGCCACCGCCAGGGTCCAGCCGCTCATCAGCAGCACCCACAGCAGCA
query      GCCGGCTGAACACCGCCACCGCCAGGGTCCAGCCGCTCATCAGCAGCACCCACAGCAGCA

template   GCGAGCCCTCGTGCGCCCCCCAGGTGGCGGCCACCCGATACCACACCGGCAGCTGCGTGT
query      GCGAGCCCTCGTGCGCCCCCCAGGTGGCGGCCACCCGGTACCACACCGGCAGCTGCGTGT

template   TCGAGTTGCCGGCGACATAGGCCACGGTGAAGTCGTTAACCACAAAGGCGTGCACCAGCA
query      TCGAGTTGCCGGCGACATAGGCCACGGTGAAGTCGTTAACCACAAAGGCGTGCACCAGCA

template   CGAAAAACGCGCCCGCCACGCAGATAAACAGCAGCCAGGCGAACACCCCGGCCGACGCCA
query      CGAAAAACGCGCCCGCCACGCAGATAAACAGCAGCCAGGCGAACACCCCGGCCGACGCCA

template   TCATCCGCGCGTCGCCGCGCGCCACGCCCCACAGCGGGTAAACGGACAGCAGCAGCGCCA
```

```
query       TCATCCGCGCGTCACCGCGCGCCACGCCCCACAGCGGGTAAACGGACAGCAGCAGCGCCA

template    CGCCGAGCGCCAGGCACAGCAGCGCGTGGCCGTATTCAGGCATCATGATGAGGTGTCCTT
query       CGCCGAGCGCCAGGCACAGCAGCGCGTGGCCGTATTCAGGCATCATGATGAGGTGTCCTT

template    ATCAACGCGTTGCGGGCGGCGGTGGTTTTCCTGCATCGCCTTTTCCACTTCCGGCGGGGT
query       ATCAGCGCGTTGCGGGCGGCGGTGGTTTTCCTGCATCGCCTTTTCCACTTCCGGCGGGGT

template    GTAGTTCTCGTCATGCTTGGCCAGCACCTCGTGCGCCAGGACGTGGTTGCCCTTCTCCAG
query       GTAGTTCTCGTCATGTTTGGCCAGCACCTCGTGCGCCAGGACGTGGTTGCCCTTCTCCAG

template    GGTGCCCTGCACCACCACCCCCTGCCCCTCGCGGAACAGGTCCGGCAGTATCCCCTCATA
query       GGTGCCCTGCACCACCACCCCCTGCCCCTCGCGGAACAGGTCCGGCAGTATCCCCTCATA

template    GCTCACCGTCACCGACCCTTCGGCGTCGTAGAGGCTGAAGTTCACCTTCAGCGAGTCCGG
query       GCTCACCGTCACCGACCCTTCGGCGTCGTAGAGACTGAAGTTCACCTTCAGCGAGTCCGG

template    GTCGCGCCTGACGCTGCCGGGCATCACCATCCCGCCGACGCGCAGGCGCTGGCCCACCGC
query       GTCGCGCCTGACACTGCCGGGCATCACCATCCCGCCGACGCGCAGGCGCTGGCCCGCCGC

template    CGGCAGCTGCTGCGTCTCGCGCTTGCCGTAGAGAATTTCGCCGGGGGTATAGAACAGGTC
query       CGGCAGCTGCTGCGTCTCGCGCTTGCCGTAGAGGATTTCGCCGGGGGTATAGAACAGGTC

template    GATATTCGCGCGCAGCGCGTACAGGACCAGGGCGGTGGTCAGCGCCAGGCCCGCCAGCAC
query       GATATTCGCGCGCAGCGCGTACAGGACCAGGGCGGTGGTCAGCCCCAGGCCCGCCAGCAC

template    CGCGCAGACCACCCATAGCCGGTTTTTACGTCGCAGGTTCAC
query       CGCGCAGACCACCCATAGCCGGTTTTTACGTCGCAGGTTCAC
```

SPI-13_AY956832

```
template    AAATATCGGGCATATTGCCCCTCCTGTTAACCTCACTATGCCCTCGCTGGTTTCATTCTT
query       AAATATCGGGCATATTGCACCTCCTGTTAACCTCACTATGCGCTCGCTGGTTTCATTCTT

template    CCAATATATGATGGTGCCATTCTTAATTGCTTTTGGAGATGAGGATGGAACTGCGCCATA
query       CCAATATATGATGGTGCCATTCTTAATTGCTTTTGGAGATGAGGATGGAACTGCGCCATA

template    TTCGCTATTTTCTCGCCGTCGCTGAAGAGCGGCATTTCACGCGGGCTGCGACAAAATTAG
query       TTCGCTATTTTCTCGCCGTCGCTGAAGAGCGGCATTTCACGCGGGCTGCGACAAAATTAG

template    GCATAGGTCAGCCGCCGCTAAGCCAACAGATTAAGGATCTGGAAAGAGAGCTGGGAGCGC
query       GCATAGGTCAGCCGCCGCTAAGCCAACAGATTAAGGATCTGGAAAGAGAGCTGGGAGCGC

template    AGCTATTCCGGAGAGTGCCTCATGGCGCGGAATTAACCGAGGCTGGAAAAGCATTTTATG
query       AGCTATTCCGGAGAGTGCCTCATGGCGCGGAATTAACCGAGGCTGGAAAAGCATTTTATG

template    ATGTTGTTAAAGGGATGCCTGCTACCGCGACAAGAGCC
query       ATGTTGTTAAAGGGATGCCTGCTACCGCGACAAGAGCC
```

SPI-13_AY956833

```
template    CCTGCAAATCGTGGATAGTCTTATCATGATGGCTGGTTCTGATACTCGCCTTATTGGCAT
query       CCTGCAAATCGTGGATAGTCTTATCATGATGGCTGGTTCTGATACTCGCCTTATTGGCAT

template    TATTGAGTCAGTTGTTGGGCTTAATGCCGTAGAGTCTATTGCGGACGCCACCCCCAGACT
query       TATTGAGTCAGTTGTTGGGCTTAATGCCGTAGAGTCTATTGCGGACGCCACCCCCAGACT

template    CTGCGGGTTGATGTTTGGCGCCGCAGATATGGCGGCTGACATTGGCGCCACACCCGCCTG
query       CTGCGGGTTGATGTTTGGCGCCGCAGATATGGCGGCTGACATTGGCGCCACACCCGCCTG

template    GGAGCCTCTTGCGCTGGCGCGGGCCAGAATAGTGGCTGCCTGCGCTATGAAAGGGCTTCT
query       GGAGCCTCTTGCGCTGGCGCGGGCCAGAATAGTGGCTGCCTGCGCTATGAAAGGGCTTCT

template    TGCAATAGATGCTCCCTTCTTTGATATAGGCGATTTTTCAGGTCTGAAAGAGGAAACGCT
query       TGCAATAGATGCTCCCTTCTTTGATATAGGCGATTTTTCAGGTCTGAAAGAGGAAACGCT
```

```
template    GCAGGCGCTCAGTTTTGGTTTTTCTGCAAAATCGGCTATTCACCCCGCTCAGATTAGTGT
query       GCAGGCGCTCAGTTTTGGTTTTTCTGCAAAATCGGCTATTCACCCCGCTCAGATTAGTGT

template    AATCAACGCCGCATTTACTCCTACAACGGCAGAAATTAATCATG
query       AATCAACGCCGCATTTACTCCTACAACGGCAGAAATTAATCATG
```

SPI-13_AY956834

```
template    AGATGGGGGTCGGCGCATTACCGGAGCTCATCTGTAATGCGCTCAAGGAACATAATGATC
query       AGATGGGGGTCGGCGCATTACCGGAGCTCATCTGTAATGCGCTCAAGGAACATAATGATC

template    TGGGGGTCCATACAGAGGCGCTGAATCCGGGGTTGGTAAGTTTGATTCAACAAGGCGTCG
query       TGGGGGTCCATACAGAGGCGCTGAATCCGGGGTTGGTAAGTTTGATTCAACAAGGCGTCG

template    TTACCAATCAGCGTAAAAATATCGACCGGGGAATGTCTGTATTTACTTTTGCTATGGGAC
query       TTACCAATCAGCGTAAAAATATCGACCGGGGAATGTCTGTATTTACTTTTGCTATGGGAC

template    AAAAGGATATGTATGACTATCTCAACGACAACCCCTCTTTTTTTAGTCGGCCTGTCGATT
query       AAAAGGATATGTATGACTATCTCAACGACAACCCCTCTTTTTTTAGTCGGCCTGTCGATT

template    ATGTTAACGATCCGGGGATCATTGCGCAAAATGAGAATGTTGTCTCCATTAACGCTACCT
query       ATGTTAACGATCCGGGGATCATTGCGCAAAATGAGAATGTTGTCTCCATTAACGCTACCT

template    TACAAATCGATCTCACTGGTGCCTGTAACTCTGAATATTTG
query       TACAAATCGATCTCACTGGTGCCTGTAACTCTGAATATTTG
```

SPI-14_AY956835

```
template    GCTTTAGTTTGTGTAAAACAAGTTGTCGATCATAACGTTAGAATTAGAATAAAGCGAGAT
query       GCTTTAGTTTGTGTAAAACAAGTTGTCGATCATAACGTTAGAATTAGAATAAAGCGAGAT

template    CATTCTGATGTTGATATTTCTGATTCAAAACTCAGTATCAATCCATTTGATGAAATTGCA
query       CATTCTGATGTTGATATTTCTGATTCAAAACTCAGTATCAATCCATTTGATGAAATTGCA

template    GTTGAAGAAGCAGTACGTTTAAAAGAACGGGGATTAATCAGTGAGGTTGTTGTAGTGTCT
query       GTTGAAGAAGCAGTACGTTTAAAAGAACGGGGATTAATCAGTGAGGTTGTTGTAGTGTCT

template    ATAGGAAACACAGGTGTAGGGGATGTATTGCGTACAGCACTGGCAGCAGGGGCAGACAGG
query       ATAGGAAACACAGGTGTAGGGGATGTATTGCGTACAGCACTGGCAGCAGGGGCAGACAGG

template    GCAATACACATATTAACAAAAAACTCTCTTACACCACTAATTGTGGCCAAAACTATCACC
query       GCAATACACATATTAACAAAAAACTCTCTTACACCACTAATTGTGGCCAAAACTATCACC

template    GCTATTACCCGGAATGAAAAACCCGATATTATATTATTAGGCAAGCAAGCTATTGACGAT
query       GCTATTACCCGGAATGAAAAACCCGATATTATATTATTAGGCAAGCAAGCTATTGACGAT

template    GATTGTAATCAGGTAGGCCAAATGCTGGCAGCATTACTCGATCTTCCCCAAGCCACTAAT
query       GATTGTAATCAGGTAGGCCAAATGCTGGCAGCATTACTCGATCTTCCCCAAGCCACTAAT

template    GTTAGTGAAATTACTATTTCTGATAATTCATTAACTGCTGTTCGTGAGGTTGATGGAGGA
query       GTTAGTGAAATTACTATTTCTGATAATTCATTAACTGCTGTTCGTGAGGTTGATGGAGGA

template    TTAGAAACGTTAAATTTATCC
query       TTAGAAACGTTAAATTTATCC
```

SPI-14_AY956836

```
template    CGTTATCCTCTGCTGGTGAAATATTTAAACACCACGCCAGCCTTATTGTGAATCAGATGG
query       CGTTATCCTCTGCTGGTGAAACATTTAAACACCACGCCAGCCTTATTGTGAATCAGATGG

template    AAATGTTACGTAGCGATATGCAGGAGCATGTTAAAGGCATCAAAGGACATGTCAGAATAT
query       AAATGTTACGTAGCGATATGCAGGAGCATGTTAAAGGCATCAAAGGACATGTCAGAATAT

template    TTTCCAGTATAACTGCTATTGCAGAGTATCTCCCCCCATTGTTGAGCCATTTCCTTACAC
```

```
query       TTTCCAGTATAACTGCTATTGCAGAGTATCTCCCCCCATTGTTAAGCCATTTCCTTACAC

template    TATATAACGATGTGAATATTGATCTCAGGGAGTATCCAAGTCCGGAAATTGTACGTTCTG
query       TATATAACGATGTGAATATTGATCTCAGGGAGTATCCAAGTCCGGAAATTGTACGTTCTG

template    TTATTGAGGGGAAAACTGATCTCGGAATTGTTGCTGGTAATATTACAACAGATAATCTTG
query       TTATTGAGGGGAAAACTGATCTCGGAATTGTTGCTGGTAATATTACAACAGATAATCTTG

template    AAATACTGCCGTTCCGTACTGATAGGCTAGTGCTTATCACCGCGACAGACCATCCCTTTG
query       AAATACTGCCGTTCCGTACTGATAGGCTAGTGCTTATCACCGCGACAGACCATCCCTTTG

template    CTGACAGAAAAAGCGTGACCTTTCATGAATTAATTCAATATGAACAAATCAGCCTGCCTG
query       CTGACAGAAAAAGCGTGACCTTTCATGAATTAATTCAATATGAACAAATCAGCCTGCCTG

template    AAGGGACAGGAATGTTTGAAT
query       AAGGGACAGGAATGTTTGAAT
```

SPI-2_AY956826

```
template    GAAAAGCAATTACGCGGCAAGCTTACCGTACTTGATCAGCAGCAACAGGCGATTTTTACG
query       GAAAAGCAATTACGCGGCAAGCTTACCGTACTTGATCAGCAGCAACAGGCGATTATTACG

template    GAACAGCAGATTTGCCAGACGCGCGCTTTAGCAGTGTCTACCAGACTGAAAGAATTAATT
query       GAACAGCAGATTTGCCAGACGCGCGCTTTAGCAGTGTCTACCAGACTGAAAGAATTAATG

template    GGCTGGCAAGGTACGTTATCTTGTCATTTATTGTTGGATAAGAAACAACAAATGGCCGGG
query       GGCTGGCAAGGTACGTTATCTTGTCATTTATTGTTGGATAAGAAACAACAAATGGCCGGG

template    TTATTCACTCAGGCGCAGAGCTTTTTGACGCAACGGCAGCAGTTAGAGAATCAGTATCAG
query       TTATTCACTCAGGCGCAGAGCTTTTTGACGCAACGGCAGCAGTTAGAGAATCAGTATCAG

template    CAGCTTGTCTCCCGGCGAAGCGAATTACAGAAGAATTTTAATGCGCTTATGAAAAAGAAA
query       CAGCTTGTCTCCCGGCGAAGCGAATTACAGAAGAATTTTAATGCGCTTATGAAAAAGAAA

template    GAAAAAATTACTATGGTATTAAGCGATGCGTATTACCAAAGTTGAGGGAAGTCTTGGGTT
query       GAAAAAATTACTATGGTATTAAGCGATGCGTATTACCAAAGTTGAGGGAAGTCTTGGGTT

template    GCCATGCCAGTCTTATCAGGATGATAACGAGGCGGAGGCGGAACGTATGGACTTTGAACA
query       GCCATGCCAGTCTTATCAGGATGATAACGAGGCGGAGGCGGAACGTATGGACTTTGAACA

template    ACTCATGCACCAGGCATTACCCATTGGTGAGAATAATCCTCCTGCAGCATTGAATAAGAA
query       ACTCATGCACCAGGCATTACCCATTGGTGAGAATAATCCTCCTGCAGCATTGAATAAGAA

template    CGTGGTTTTTACGCAACGTTATCGTGTTAGTGGCGGTTATCTTGACGGTGTAGAGTGTGA
query       CGTGGTTTTCACGCAACGTTATCGTGTTAGTGGCGGTTATCTTKACGGKGTAGAGTGTGA

template    AGTCTGTGAATCAGGGGGGCTAATTCAGTTAAGAATCAATGTCCCTCATCATGAAATTTA
query       AGTATGTGAATCAGGGGGGCTAATCCAGTTAAGAATCAATGTCCCTCATCATGAAATTTA

template    CCGTTCGATGAAAGCGCTAAAGCAGTGGCTGGAGTCT
query       CCGTTCGATGAAAGCGCTAAAGCAGTGGCTGGAGTCT
```

SPI-2_AY956827

```
template    AGGATATGCTTAGTGGAATCATTGCTAAAATCTGAAGTTATTAGCGACGATGTTCGACGG
query       AGGATATGCTTAGTGGAATCATTGCTAAAATCTGAAGTTATTAGCGACGATGTTCGACGG

template    TTGCTGCTGGAAATCATGTTTGCGGGCGTTAACCATAGCCTGATTTCCCAGGTACATGCG
query       TTGCTGCTGGAAATCATGTTTGCGGGCGTTAACCATAGCCTGATTTCCCAGGTACATGCG

template    ATGTTACCAGCGCTAACGGTTATTGTTCCGGATAAAAAATTACAGTTGGTATGTCTGGCA
query       ATGTTACCAGCGCTAACGGTTATTGTTCCGGATAAAAAATTACAGTTGGTATGTCTGGCA

template    TTATTGTTGGCGGGTTTAAATGAGCCGCTAAAAGCCGCGAAAATTTTATCGGATATAGAT
query       TTATTGTTGGCGGGTTTAAATGAGCCGCTAAAAGCCGCGAAAATTTTATCGGATATAGAT
```

```
template   TTGCCAGAGGCTATGGCGCTGCGTCTGTTATTTCCTGCACCAAATGAGGGGTTTGAAAAT
query      TTGCCAGAGGCTATGGCGCTGCGTCTGTTATTTCCTGCACCAAATGAGGGGTTTGAAAAT

template   TGAATATTTCTGATATGAGCGTAGTGCCTGTAAGCACTCAATCTTATGTAAAGTCCTCTG
query      TGAATATTTCTGATATGAGCGTAGTGCCTGTAAGCACTCAATCTTATGTAAAGTCCTCTG

template   CAGAACCGAGCCAGGAGCAAATTAATTTTTTTGAACAATTGCTGAAAGATGAAGCATCCA
query      CAGAACCGAGCCAGGAGCAAATTAATTTTTTTGAACAATTGCTGAAAGATGAAGCATCCA

template   CCAGTAACGCCAGTGCTTTATTACCGCAGGTTATGTTGACCAGACAAATGGATTATATGC
query      CCAGTAACGCCAGTGCTTTATTACCGCAGGTTATGTTGACCAGACAAATGGATTATATGC

template   AGTTAACGGTAGGCGTCGATTATCTTGCCAGAATATCAGGCGCAGCATCGCAAGCGCTTA
query      AGTTAACGGTAGGCGTCGATTATCTTGCCAGAATATCAGGCGCAGCATCGCAAGCGCTTA

template   ATAAGCTGGATAACATGGCATGAAGGTTCATCGTATAGTATTTCTTACTGTCCTTACGTT
query      ATAAGCTGGATAACATGGCATGAAGGTTCATCGTATAGTATTTCTTACTGTCCTTACGTT

template   CTTTCTTACGGCATGTGATGTGGATCTTTATCGCTCATTGCC
query      CTTTCTTACGGCATGTGATGTGGATCTTTATCGCTCATTGCC
```

SPI-2_AY956828

```
template   GAGTGAGGCTGTTCACACTTATAGCGTACTGTCAATCGGAGATGGTTTATGTGGGCAAAT
query      GAGTGAGGCTGTTCACACTTATAGCGTACTGTCAATCGGAGATGGTTTATGTGGGCAAAT

template   TCCATCGCTGCTGATTTCCCTTAGCGCGGGAATTATTGTCACCCGTGTCCCGGGTGAGAA
query      TCCATCGCTGCTGATTTCCCTTAGCGCGGGAATTATTGTCACCCGTGTCCCGGGTGAGAA

template   ACGCCAGAACCTGGCGACAGAGTTGAGTTCTCAAATTGCCAGACAACCTCAGTCGCTCAT
query      ACGCCAGAACCTGGCGACAGAGTTGAGTTCTCAAATTGCCAGACAACCTCAGTCGCTCAT

template   ATTAACCGCTGTGGTTTTAATGCTCCTCGCTTTAATTCCTGGCTTTCCTTTTATCACTCT
query      ATTAACCGCTGTGGTTTTAATGCTCCTCGCTTTAATTCCTGGCTTTCCTTTTATCACTCT

template   CGCTTTCTTTTCAGCGTTGTTAGCATTGCCAATTATCCTCATTCGCCGCAAAAAGTCTGT
query      CGCTTTCTTTTCAGCGTTGTTAGCATTGCCAATTATCCTCATTCGCCGCAAAAAGTCTGT

template   GGTTTCCGCAAATGGCGTCGAAGCACCGGAAAAAGATAGTATGGTTCCCGGCGCATGTCC
query      GGTTTCCGCAAATGGCGTCGAAGCACCGGAAAAAGATAGTATGGTTCCCGGCGCATGTCC

template   TCTAATCTTACGTCTTAGCCCGACGTTACATTCTGG
query      TCTAATCTTACGTCTTAGCCCGACGTTACATTCTGC
```

SPI-2_AY956829

```
template   AGTTGGCGTGCCCATATTGCATTGGCTGGCACCTGGATGATGCTTAAAGAATACACGACG
query      AGTTGGCGTGCCCATATTGCATTGGCTGGCACCTGGATGATGCTTAAAGAATACACGACG

template   GCCATTAATTTCTATGGACATGCCTTGATGCTGGATGCCAGCCATCCAGAACCGGTTTAC
query      GCCATTAATTTCTATGGACATGCCTTGATGCTGGATGCCAGCCATCCAGAACCGGTTTAC

template   CAAACGGGCGTCTGTCTCAAAATGATGGGGGAACCCGGGTTGGCGAGAGAGGCTTTTCAA
query      CAAACGGGCGTCTGTCTCAAAATGATGGGGGAACCCGGGTTGGCGAGAGAGGCTTTTCAA

template   ACCGCAATCAAGATGAGTTATGCGGATGCCTCATGGAGTGAGATTCGCCAGAATGCGCAA
query      ACCGCAATCAAGATGAGTTATGCGGATGCCTCATGGAGTGAGATTCGCCAGAATGCGCAA

template   ATAATGGTTGATACTCTTATTGCTTAAATAACAGAACGAAATATGAAAATTCATATTCCG
query      ATAATGGTTGATACTCTTATTGCTTAAATAACAGAACGAAATATGAAAATTCATATTCCG

template   TCAGCGGCAAGTAATATAGTCGATGGTAATAGTCCTCCTTCCGATATACAAGCGAAGGAG
query      TCAGCGGCAAGTAATATAGTCGATGGTAATAGTCCTCCTTCCGATATACAAGCGAAGGAG

template   GTATCGTTTCCTCCCCCTGAAATTCCAGCGCCTGGTACCCCCGCAGCCCCTGTGCTGCTT
```

```
query      GTATCGTTTCCTCCCCCTGAAATTCCAGCGCCTGGCACCCCCGCAGCCCCTGTGCTGCTT

template   ACGCC
query      ACGCC
```

SPI-2_AY956830

```
template   ACATGTCCCTCTCTATTTTCATACCGACAGTATCCGTTTACGGCAAATTTTGGTTAATTT
query      ACATGTCCCTCTCTATTTTCATACCGACAGTATCCGTTTACGGCAAATTTTGGTTAATTT

template   ACTCGGGAATGCGGTAAAATTTACCGAAACCGGAGGGATACGTCTGACGGTCAAGCGTCA
query      ACTCGGGAACGCGGTAAAATTTACCGAAACCGGAGGGATACGTCTGACGGTCAAGCGTCA

template   TGAGGAACAATTAATATTTCTGGTTAGCGATAGCGGTAAAGGGATTGAAATACAGCAGCA
query      TGAGGAACAATTAATATTTCTGGTTAGCGATAGCGGTAAAGGGATTGAAATACAGCAGCA

template   GTCTCAAATCTTTACTGCTTTTTATCAAGCAGACACAAATTCGCAAGGTACAGGAATTGG
query      GTCTCAAATCTTTACTGCTTTTTATCAAGCAGACACAAATTCGCAAGGTACAGGAATTGG

template   ACTGACTATTGCGTCAAGCCTGGCTAAAATGATGGGCGGTAATCTGACACTAAAAAGTGT
query      ACTGACTATTGCGTCAAGCCTGGCTAAAATGATGGGCGGTAATCTGACACTAAAAAGTGT

template   CCCCGGGGTTGGAACCTGTGTCTCGCTAGTATTACCCTTACAAGAATACCAGCCGCCTCA
query      CCCCGGGGTTGGAACCTGTGTCTCGCTAGTATTACCCTTACAAGAATACCAGCCGCCTCA

template   ACCAATTAAAGGGACGCTGTCAGCGCCGTTCTGCCTGCATCGGCAACTGGCTTGCTGGGG
query      ACCAATTAAAGGGACGCTGTCAGCGCCGTTCTGCCTGCATCGGCAACTGGCTTGCTGGGG

template   AATACGCGGTGAACCACCCCACCAGCAAAATGCGCTTCTCAACGCAGAGCTTTTGTATTT
query      AATACGCGGTGAACCACCCCACCAGCAAAATGCGCTTCTCAACGCAGAGCTTTTGTATTT

template   CTCCGGAAAACTCTACGACCTGGCGCAACAGTTAATATTGTGTACACCAAATATGCCAGT
query      CTCCGGAAAACTCTACGACCTGGCGCAACAGTTAATATTGTGTACACCAAATATGCCAGT

template   AATAAAT
query      AATAAAT
```

SPI-2_JN673272

```
template   ATGCTGGCAGTTTTAAAAGGCATTCCATTAATTCAGGATATCAGGGCCGAAGGTAATAGC
query      ATGCTGGCAGTTTTAAAAGGCATTCCATTAATTCAGGATATCAGGGCCGAAGGTAATAGC

template   CGATCCTGGATAATGACTATTGATGGGCATCCTGCCAGAGGAGAAATTTTCTCAGAAGCA
query      CGATCCTGGATAATGACTATTGATGGGCATCCTGCCAGAGGAGAAATTTTCTCAGAAGCA

template   TTTTCTATTTCTTTGTTCTTAAATGACCTGGAAAGCTTACCTAAGCCTTGTCTTGCCTAT
query      TTTTCTATTTCTTTGTTCTTAAATGACCTGGAAAGCTTACCTAAGCCTTGTCTTGCCTAT

template   GTGACACTACTGCTTGCAGCACACCCGGACGTCCATGATTATGCTATACAGCTCACAGCG
query      GTGACACTACTGCTTGCAGCACACCCGGACGTCCATGATTATGCTATACAGCTCACAGCG

template   GATGGGGGATGGTTAAACGGTTATTATACCACAAGTAGTAGCTCTGAGCTTATTGCTATT
query      GATGGGGGATGGTTAAACGGTTATTATACCACAAGTAGTAGCTCTGAGCTTATTGCTATT

template   GAGATAGAAAAACACCTGGCTTTAACTTGCATTTTAAAAAATGTAATACGCAATCACCAT
query      GAGATAGAAAAACACCTGGCTTTAACTTGCATTTTAAAAAATGTAATACGCAATCACCAT

template   AAACTTTATTCGGGTGGGGTATAA
query      AAACTTTATTCGGGTGGGGTATAA
```

SPI-2_Z95891

```
template   TTACAAAACCAGTGACATTGGCTACCTTAGCTCGCTACATCAGTATTGCCGCAGAATACC
query      TTACAAAACCAGTGACATTGGCTACCTTAGCTCGCTACATCAGTATTGCCGCAGAATACC
```

```
template   AACTTTTACGAAATATAGAGCTACAGGAGCAGGATCCGAGTCGCTGCTCAGCGCTACTGG
query      AACTTTTACGAAATATAGAGCTACAGGAGCAGGATCCGAGTCGCTGCTCAGCGCTACTGG

template   CGACAGATGATATGGTCATTAATAGCAAGATTTTCCAATCACTGGACCTCTTGCTGGCTG
query      CGACAGATGATATGGTCATTAATAGCAAGATTTTCCAATCACTGGACCTCTTGCTGGCTG

template   ATATTGAAAATGCCGTATCGGCTGGAGAAAAAATCGATCAGTTAATTCACACATTAAAAG
query      ATATTGAAAATGCCGTATCGGCTGGAGAAAAAATCGATCAGTTAATTCACACATTAAAAG

template   GCTGTTTAGGTCAAATAGGGCAGACTGAATTGGTATGCTATGTCATAGACATTGAGAATC
query      GCTGTTTAGGTCAAATAGGGCAGACTGAATTGGTATGCTATGTCATAGACATTGAGAATC

template   GCGTAAAAATGGGGAAAATCATCGCGCTGGAGGAACTAACCGACTTACGCCAGAAAATAC
query      GCGTAAAAATGGGGAAAATCATCGCGCTGGAGGAACTAACCGACTTACGCCAGAAAATAC

template   GTATGATCTTCAAAAACTACACCATTACTTAATATTATCTTAATTTTCGCGAGGGCAGCA
query      GTATGATCTTCAAAAACTACACCATTACTTAATATTATCTTAATTTTCGCGAGGGCAGCA

template   AAATGAAAGAATATAAGATCTTATTAGTAGACGATCATGAAATCATCATTAACGGCATTA
query      AAATGAAAGAATATAAGATCTTATTAGTAGACGATCATGAAATCATCATTAACGGCATTA

template   TGAATGCCTTATTACCCTGGCCTCATTTTAAAATTGTAGAGCATGTTAAAAATGGTCTTG
query      TGAATGCCTTATTACCCTGGCCTCATTTTAAAATTGTAGAGCATGTTAAAAATGGTCTTG

template   AGGTTTATAATGCCTGTTGTGCATACGAGCCTGACATACTTATCCTTGATCTTAGTCTAC
query      AGGTTTATAATGCCTGTTGTGCATACGAGCCTGACATACTTATCCTTGATCTTAGTCTAC

template   CTGGCATCAATGGCCTGGATATCATTCCTCAATTACATCAGCGTTGGCCAGCAATGAATA
query      CTGGCATCAATGGCCTGGATATCATTCCTCAATTACATCAGCGTTGGCCAGCAATGAATA

template   TTCTGGTTTACACAGCATACCAACAAGAGTATATGACCATTAAAACTTTAGCCGCAGGTG
query      TTCTGGTTTACACAGCATACCAACAAGAGTATATGACCATTAAAACTTTAGCCGCAGGTG

template   CTAATGGCTATGTTTTAAAAAGCAGTAGTCAGCAAGTTCTGTTAGCGGCATTGCAAACAG
query      CTAATGGCTATGTTTTAAAAAGCAGTAGTCAGCAAGTTCTGTTAGCGGCATTGCAAACAG

template   TAGCAGTAAACAAGCGTTACATTGACCCAACGTTGAATCGGGAAGCTATCCTGGCTGAAT
query      TAGCAGTAAACAAGCGTTACATTGACCCAACGTTGAATCGGGAAGCTATCCTGGCTGAAT

template   TAAACGCTGACACGACCAATCATCAACTGCTTACTTTGCGCGAGCGTCAGGTTCTTAAAC
query      TAAACGCTGACACGACCAATCATCAACTGCTTACTTTGCGCGAGCGTCAGGTTCTTAAAC

template   TTATTGACGAGGGGTATACCAATCATGGGATCAGCGAAAAGCTACATATCAGTATAAAAA
query      TTATTGACGAGGGGTATACCAATCATGGGATCAGCGAAAAGCTACATATCAGTATAAAAA

template   CCGTCGAAACACACCGGATGAATATGATGAGAAAGCTACAGGTTCATAAAGTGACAGAGT
query      CCGTCGAAACACACCGGATGAATATGATGAGAAAGCTACAGGTTCATAAAGTGACAGAGT

template   TACTTAACTGTGCCCGAAGAATGAGGTTAATAGAGTATTAACCAGGGGCGTCCGATGGTA
query      TACTTAACTGTGCCCGAAGAATGAGGTTAATAGAGTATTAACCAGGGGCGTCCGATGGTA

template   TTAAGCATTGGTCATATTTTGATGAGCCTTACGCCACGCAGTATTGCTCATCATCGACAA
query      TTAAGCATTGGTCATATTTTGATGAGCCTTACGCCACGCAGTATTGCTCATCATCGACAA

template   AATCCATACGGATGCCCTGGTATGCCGCACCATTTATCACTACCTTAGTCTTCATTTGAT
query      AATCCATACGGATGCCCTGGTATGCCGCACCATTTATCACTACCTTAGTCTTCATTTGAT

template   CATGATATAGTAGAATCCCCTTATTTAACGGGCTTTACCATGTCGTATTCTA
query      CATGATATAGTAGAATCCCCTTATTTAACGGGCTTTACCATGTCGTATTCTA
```

SPI-3_AJ000509

```
template   ATGGGTTCCACCAGAAAAGGGATGCTAAACGTCCTGATTGCCGCCGTATTGTGGGGGAGT
query      ATGGGTTCCACCAGAAAAGGGATGCTAAACGTCCTGATTGCCGCCGTATTGTGGGGGAGT

template   TCAGGCGTTTGCGCGCAGTACATCATGGAGCAAAGCCGTATGTCGTCACAGTTCCTGACG
query      TCAGGCGTTTGCGCGCAGTACATCATGGAGCAAAGCCGTATGTCGTCACAGTTCCTGACG
```

```
template   ATGATACGTTTGTTATTCGCCGGGCTGATACTGGTGACCTTCTCCTTTATGCACGGCGAT
query      ATGATACGTTTGTTATTCGCCGGGCTGATACTGGTGACCTTCTCCTTTATGCACGGCGAT

template   AAGATATTTTCAATTCTTAAAAACCGCAAAGATGCCCTGAGTCTGCTGATTTTCTCCGTG
query      AAGATATTTTCAATTCTTAAAAACCGCAAAGATGCCCTGAGTCTGCTGATTTTCTCCGTG

template   GTGGGCGCGCTCACCGTTCAGCTAACCTTCCTGCTCACGATTGAAAAATCCAATGCCGCC
query      GTGGGCGCGCTCACCGTTCAGCTAACCTTCCTGCTCACGATTGAAAAATCCAATGCCGCC

template   ACCGCGACAGTGCTGCAATTTTTATCGCCGACCATTATTGTAGCGTGGTTTGCATTAGCG
query      ACCGCGACAGTGCTGCAATTTTTATCGCCGACCATTATTGTAGCGTGGTTTGCATTAGCG

template   CGAAGAACACGACCAGGCATTCTGGTCTTAACCGCCATTCTTACATCGCTTATCGGCACC
query      CGAAGAACACGACCAGGCATTCTGGTCTTAACCGCCATTCTTACATCGCTTATCGGCACC

template   TTTTTACTGGTGACTCACGGCAATCCAACATCGCTGTCGATCTCTTCAGCCGCGCTGTTC
query      TTTTTACTGGTGACTCACGGCAATCCAACATCGCTGTCGATCTCTTCAGCCGCGCTGTTC

template   TGGGGTATCGCCTCCGCATTTGCCGCCGCCTTTTATACGACCTGGCCTTCCAGGCTAATC
query      TGGGGTATCGCCTCCGCATTTGCCGCCGCCTTTTATACGACCTGGCCTTCCAGGCTAATC

template   GCCCAATACGGCACACTGCCAGTGGTCGGCTGGAGTATGTCCTTTGGCGGCCTTATTCTG
query      GCCCAATACGGCACACTGCCAGTGGTCGGCTGGAGTATGTCCTTTGGCGGCCTTATTCTG

template   CTGCCCTTCTACGCTAAAGAAGGAACGCACTTTGCGGTGAGCGGCAGCCTGATTCTGGCC
query      CTGCCCTTCTACGCTAAAGAAGGAACGCACTTTGCGGTGAGCGGCAGCCTGATTCTGGCC

template   TTTTTCTACCTGGTGGTGATCGGTACATCGCTGACGTTCAGCCTGTATTTGAAAGGCGCG
query      TTTTTCTACCTGGTGGTGATCGGTACATCGCTGACGTTCAGCCTGTATTTGAAAGGCGCG

template   CAACTGATTGGTGGCCCC
query      CAACTGATTGGTGGCCCC
```

SPI-3_Y13864

```
template   AGCGGGAATGCCGAAAATATCGGTATAGAAGAACATCATATATAACATGACGTTATCAAA
query      AGCGGGAATGCCGAAAATATCGGTATAGAAGAACATCATATATAACATGACGTTATCAAA

template   GATGATGTGGCTGGCGGCATCGCCCATACCATAGCCAATCTTCTCCTTAACGGAGAGAAT
query      GATGATGTGGCTGGCGGCATCGCCCATACCATAGCCAATCTTCTCCTTAACGGAGAGAAT

template   TTCACTTTTCATTACGCATCCTTAACGCTCACAACGCTCACTAATACCGGTTACAGTTTT
query      TTCACTTTTCATTACGCATCCTTAACGCTCACAACGCTCACTAATACCGGTTACAGTTTT

template   TGTAAACCTTCCCTGCGAGCGTGAATATTCCGTTATTTGGTTATCAAATTACGATTCTTG
query      TGTAAACCTTCCCTGCGAGCGTGAATATTCCGTTATTTGGTTATCAAATTACGATTCTTG

template   TTTTCTGTGATCAGGGTAGCGTCAAAAAGTGTGATGAAATGTAACTAATGGATTTTATGG
query      TTTTCTGTGATCAGGGTAGCGTCAAAAAGTGTGATGAAATGTAACTAATGGATTTTATGG

template   GGGTTTATATGAAACCGATCATGGCAGAAGGAAAGGGAAGTTAAAAAAGCACGGGAGGGG
query      GGGTTTATATGAAACCGATCATGGCAGAAGGAAAGGGAAGTTAAAAAAGCACGGGAGGGG

template   ATGTGGAAACGCCAGGAAGTAGCTATAATGCGCGCGCCTCCATGTAGCAATCGAGGCGCG
query      ATGTGGAAACGCCAGGAAGTAGCTATAATGCGCGCGCCTCCATGTAGCAATCGAGGCGCG

template   GAAGATCGTCGTCTCCGGTGAGGCGGCTGGACTTCAAATCCAGTTGGGGCCGCCAGCGGT
query      GAAGATCGTCGTCTCCGGTGAGGCGGCTGGACTTCAAATCCAGTTGGGGCCGCCAGCGGT

template   CCCGGGCAGGTTCGACTCCTGTGATCTTCCGCCAACATCCTTCTCTCATGCCCACCATTC
query      CCCGGGCAGGTTCGACTCCTGTGATCTTCCGCCAACATCCTTCTCTCATGCCCACCATTC

template   GTCCTCTATTTCGGGACAAAGTATTTGCTTATTATTAAGACTAGTTCTTGCATATAATAC
query      GTCCTCTATTTCGGGACAAAGTATTTGCTTATTATTAAGACTAGTTCTTGCATATAATAC

template   GAAAGGAAAGGTGGATAGAGAAAGTGGAATATTATTTTATTAATCACCATTTACCGATTG
query      GAAAGGAAAGGTGGATAGAGAAAGTGGAATATTATTTTATTAATCACCATTTACCGATTG
```

```
template    CTAAAACCGAACGTCCATTTATTGTGGGATATCGTCTGCAAAGAAGGGATAAAAGTACGC
query       CTAAAACCGAACGTCCATTTATTGTGGGATATCGTCTGCAAAGAAGGGATAAAAGTACGC

template    CAGGACTAACGCGGGCGGGACTTAACTAATCACCAAACGAACTTGTGGGTCATACTTACA
query       CAGGACTAACGCGGGCGGGACTTAACTAATCACCAAACGAACTTGTGGGTCATACTTACA

template    GGCTCATCCTTTCTCAAAAGAGATACGCTTACCAGGCCAAAATTATGAGATCAACGTATA
query       GGCTCATCCTTTCTCAAAAGAGATACGCTTACCAGGCCAAAATTATGAGATCAACGTATA

template    GAGCCATCCGGATAAAGATACATGCCTCCCTCCAGATACACGGGTTCTCCTTCTTCTACC
query       GAGCCATCCGGATAAAGATACATGCCTCCCTCCAGATACACGGGTTCTCCTTCTTCTACC

template    GCTAAATTATGATATAGCGATTGCATTTCATCATCTGCACTTTCTTCCGGTTGGTTTTCA
query       GCTAAATTATGATATAGCGATTGCATTTCATCATCTGCACTTTCTTCCGGTTGGTTTTCA

template    ACTCGATAATAGGTATCTACATCATAGTTCTGTTTACGGAGATCGTCGTCGCTGTATACG
query       ACTCGATAATAGGTATCTACATCATAGTTCTGTTTACGGAGATCGTCGTCGCTGTATACG

template    CCAAGCCGTTTTTTTAAGCCTGTTATATTAAATATCCATTTTAACCCATCTTTAATATAA
query       CCAAGCCGTTTTTTTAAGCCTGTTATATTAAATATCCATTTTAACCCATCTTTAATATAA

template    TAGGCATCACCATAAGCATTTTCATCGGGGCATTCTTTATAGCCCATCCGCAATGCAACA
query       TAGGCATCACCATAAGCATTTTCATCGGGGCATTCTTTATAGCCCATCCGCAATGCAACA

template    TCACAGCCATGCTTACCAACATATTTATAACTCATATCCCCCCCAATATGTTGCTTCAAT
query       TCACAGCCATGCTTACCAACATATTTATAACTCATATCCCCCCCAATATGTTGCTTCAAT

template    ATCCCAGATAAAATTCATAACGAAAACCTATCCTGATAACCTATGCCACATGAAGGATAC
query       ATCCCAGATAAAATTCATAACGAAAACCTATCCTGATAACCTATGCCACATGAAGGATAC

template    AATTTAAACTAAAGTACGCGTTATATTATCCCCTAAGTCCGTATCAAGTATGCGTATTCT
query       AATTTAAACTAAAGTACGCGTTATATTATCCCCTAAGTCCGTATCAAGTATGCGTATTCT

template    GTAGTAGGTAGTAATATGTTCTAATTTATTAATGTGAGTTATATATGAAGAGATTATTTA
query       GTAGTAGGTAGTAATATGTTCTAATTTATTAATGTGAGTTATATATGAAGAGATTATTTA

template    CAGCGATGATTATCGCTTTTATTACCGTCACGGCATCATCCAGTGCGCTTGCCTGCCCAA
query       CAGCGATGATTATCGCTTTTATTACCGTCACGGCATCATCCAGTGCGCTTGCCTGCCCAA

template    AGGGTCAACATCCACATGGGGGTACAGGCTCACATCATAAGGGTGGATGGTGTTCATACG
query       AGGGTCAACATCCACATGGGGGTACAGGCTCACATCATAAGGGTGGATGGTGTTCATACG

template    AGCAGTAACGTCGC
query       AGCAGTAACGTCGC
```

SPI-4_AJ576316

```
template    TATTGTCTTTGTATCCCTTTCAAAAAAGAGAAGCGCATATCTTACGGAAAAATGTCGCTT
query       TATTGTCTTTGTATCCCTTTCAAAAAAGAGAAGCGCATATCTTACGGAAAAATGTCGCTT

template    ATCGCCTCTGGCCGACTCGCCTCGGCACATCATTATCCAGCCGAAGTTCATAAATGTACT
query       ATCGCCTCTGGCCGACTCGCCTCGGCACATCATTATCCAGCCGAAGTTCATAAATGTACT

template    GCAATAACCCGGATTGTCTTAAATATGAAAGAGAAATCTCATCTGCAAAATATATAATTT
query       GCAATAACCCGGATTGTCTTAAATATGAAAGAGAAATCTCATCTGCAAAATATATAATTT

template    ATAGCCATTTTTTGACAACAAAAAGATATTATAAAATAACGGTAGAGAATGGTCGGTATT
query       ATAGCCATTTTTTGACAACAAAAAGATATTATAAAATAACGGTAGAGAATGGTCGGTATT

template    ATCAATGGTTAATTAAATGTTTGCTTTAGCTTGTGATGAGCTCAAATATGATATGTGTAT
query       ATCAATGGTTAATTAAATGTTTGCTTTAGCTTGTGATGAGCTCAAATATGATATGTGTAT

template    CTTGCTTTATTTTTAATTGCTGAAGATAAAATTGTTACTTATAGCTGGCTTTATATAAAA
query       CTTGCTTTATTTTTAATTGCTGAAGATAAAATTGTTACTTATAGCTGGCTTTATATAAAA

template    AATGGTTTTATTTGTGTATTTTTTTACACAATTCTGATTTTTTACTCCCCACTTATTATA
query       AATGGTTTTATTTGTGTATTTTTTTACACAATTCTGATTTTTTACTCCCCACTTATTATA
```

```
template  TTTTCAATGATTTAAGTTATATTGAAGTCCATATGACCCTATGTTTTAATTGTGAAAATT
query     TTTTCAATGATTTAAGTTATATTGAAGTCCATATGACCCTATGTTTTAATTGTGAAAATT

template  AATTTTATCCTCTGGAGGCAAATTTATTAAATACGTATGGTTATAACGCGTATTAAAAAG
query     AATTTTATCCTCTGGAGGCAAATTTATTAAATACGTATGGTTATAACGCGTATTAAAAAG

template  AAATATGTCCTTAAAATGATTTAGAGTTTCAATGATTAGCTAACAAATCTATTTATCGGC
query     AAATATGTCCTTAAAATGATTTAGAGTTTCAATGATTAGCTAACAAATCTATTTATCGGC

template  GGGTGGTTTTAATTTGCTGATGACTATTTTTTATTTATGAGTTGAGAGCTTATGCGATAC
query     GGGTGGTTTTAATTTGCTGATGACTATTTTTTATTTATGAGTTGAGAGCTTATGCGATAC

template  CTATTATTGGGGAGGATATAGTTAGTGCAATAATTGATGTCTACCTAATTAAAAAAATGA
query     CTATTATTGGGGAGGATATAGTTAGTGCAATAATTGATGTCTACCTAATTAAAAAAATGA

template  AGAGTGTTTTTAATTAAAGCACTCATCTTTGTTGTGGGCGCATAAAAATGGCGTCGTTGT
query     AGAGTGTTTTTAATTAAAGCACTCATCTTTGTTGTGGGCGCATAAAAATGGCGTCGTTGT

template  TTTTATTTTTCTGATTACGAGATGTAAGAAAACTGAAATCTATAAAGCGTATTGGTAGCA
query     TTTTATTTTTCTGATTACGAGATGTAAGAAAACTGAAATCTATAAAGCGTATTGGTAGCA

template  GGAAGCCAAGGGCGGTAGCGTTCACTTTCTGAATAAGTTGAGCATCCTTTATCCTTTCTA
query     GGAAGCCAAGGGCGGTAGCGTTCACTTTCTGAATAAGTTGAGCATCCTTTATCCTTTCTA

template  ATCCACAAAAACATTTTATTCACAATGTAATATCAGGAGACAACATGGAAGACGAAAGTA
query     ATCCACAAAAACATTTTATTCACAATGTAATATCAGGAGACAACATGGAAGACGAAAGTA

template  ATCCGTGGCCTAGTTTTGTTGATACATTCTCTACGGTATTGTGCATTTTTATATTTCTTA
query     ATCCGTGGCCTAGTTTTGTTGATACATTCTCTACGGTATTGTGCATTTTTATATTTCTTA

template  TGTTGGTGTTTGCACTTAATAATATGATTATTATGTATGACAACTCAATTAAAGTTTATA
query     TGTTGGTGTTTGCACTTAATAATATGATTATTATGTATGACAACTCAATTAAAGTTTATA

template  AAGCAAATATAGAGAATAAGACTAAATCTACTGCTCAAAATAGCGGAGCCAATGACGATT
query     AAGCAAATATAGAGAATAAGACTAAATCTACTGCTCAAAATAGCGGAGCCAATGACGATT

template  CAAATCCTAATGAAATAGTTAATAAGGAAGTGAATACTCAAGATGTGTCGGACGGAATGA
query     CAAATCCTAATGAAATAGTTAATAAGGAAGTGAATACTCAAGATGTGTCGGACGGAATGA

template  CTACAATGTCAGGTAAGGAGGTTGGAGTATACGATATCGCCGATGGTCAGAAAACTGATA
query     CTACAATGTCAGGTAAGGAGGTTGGAGTATACGATATCGCCGATGGTCAGAAAACTGATA

template  TAACGTCTACAAAAAATGAGCTTGTTATTACGTATCATGGCAGGCTGAGAAGCTTTTCAG
query     TAACGTCTACAAAAAATGAGCTTGTTATTACGTATCATGGCAGGCTGAGAAGCTTTTCAG

template  AAGAGGACACTTATAAAATAAAGGCTTGGTTAGAAGACAAAATCAATAGTAATTTATTGA
query     AAGAGGACACTTATAAAATAAAGGCTTGGTTAGAAGACAAAATCAATAGTAATTTATTGA

template  TAGAAATGGTTATTCCTCAGGCAGACATCTCTTTCTCTGACTCTCTACGACTGGGATATG
query     TAGAAATGGTTATTCCTCAGGCAGACATCTCTTTCTCTGACTCTCTACGACTGGGATATG

template  AACGGGGAATTATTTTGATGAAAGAGATTAAGAAAATATATCCTGATGTAGTTATTGACA
query     AACGGGGAATTATTTTGATGAAAGAGATTAAGAAAATATATCCTGATGTAGTTATTGACA

template  TGAGTGTTAACTCCGCAGCATCAAGTACAACGAGTAAAGCTATTATCACGACTATTAATA
query     TGAGTGTTAACTCCGCAGCATCAAGTACAACGAGTAAAGCTATTATCACGACTATTAATA

template  AAAAGGTGTCAGAGTGAAATATATAAATCATTACCGCTATTTGTTTGTCTGTTTTTTCCT
query     AAAAGGTGTCAGAGTGAAATATATAAATCATTACCGCTATTTGTTTGTCTGTTTTTTCCT

template  TGCCATACTCCCTTTTTTTGCTTTATCTTTTCCCGGTATAAGAGAGTATGTTTTTGATAA
query     TGCCATACTCCCTTTTTTTGCTTTATCTTTTCCCGGTATAAGAGAGTATGTTTTTGATAA

template  CTTCATGGTTTCTGCAATTTACAATGGAGTCATTATTGCCATTTATATTACAGGTTCTTT
query     CTTCATGGTTTCTGCAATTTACAATGGAGTCATTATTGCCATTTATATTACAGGTTCTTT

template  GTGTGCATTATTCACTATTCTTAAAAATATTTCAGCAAAAGACATATTAATAGCTCAGGA
query     GTGTGCATTATTCACTATTCTTAAAAATATTTCAGCAAAAGACATATTAATAGCTCAGGA
```

```
template    CGCCAGTAGAAAAAATAGCATTCTTTCTAACCTCAATCAGGTTTTGTTTGCCGGGGAGTC
query       CGCCAGTAGAAAAAATAGCATTCTTTCTAACCTCAATCAGGTTTTGTTTGCCGGGGAGTC

template    CAAACAGTGTGATTTCAATTTACTGATGGAATTAGATGACAATGTGTCTACCGCCCGTAA
query       CAAACAGTGTGATTTCAATTTACTGATGGAATTAGATGACAATGTGTCTACCGCCCGTAA

template    TCAACGATTGTCATTTATTATGAGCTGTAGCAATGTGTCGACGCTTGTTGGCCTGTTGGG
query       TCAACGATTGTCATTTATTATGAGCTGTAGCAATGTGTCGACGCTTGTTGGCCTGTTGGG

template    GACGTTTGCGGGTCTGTCTATTACGATTGGTTCAATCGGGAACTTATTGAGCTCGCCATC
query       GACGTTTGCGGGTCTGTCTATTACGATTGGTTCAATCGGGAACTTATTGAGCTCGCCATC

template    AGATGTTGGCGGTGATAATGCAAGTAATACACTCAATATGATCGTGACAATGGTAGCGTC
query       AGATGTTGGCGGTGATAATGCAAGTAATACACTCAATATGATCGTGACAATGGTAGCGTC

template    GCTTTCTGAACCATTGAAAGGGATGAATACCGCATTTGTATCTTCTATCTATGGTGTTGT
query       GCTTTCTGAACCATTGAAAGGGATGAATACCGCATTTGTATCTTCTATCTATGGTGTTGT

template    TTGCGCCATACTCCTGACCTCACAAAGTGTTTTTGTTCGCAGCTCCTATTCCCTTGTTTC
query       TTGCGCCATACTCCTGACCTCACAAAGTGTTTTTGTTCGCAGCTCCTATTCCCTTGTTTC

template    TACGGAAATCAAAAAATTAAAAATCATAAGCAACAGAGCCAATAATAAACAGCGAAGCCT
query       TACGGAAATCAAAAAATTAAAAATCATAAGCAACAGAGCCAATAATAAACAGCGAAGCCT

template    GCGGGTTGAATCAGAGACGCTTGTAGAATTTAAAGAGTTGTTTAAAGCGTTTTTTGATAA
query       GCGGGTTGAATCAGAGACGCTTGTAGAATTTAAAGAGTTGTTTAAAGCGTTTTTTGATAA

template    CTACCTGACAGTTGAAAACTTACGGACGCAGGATGAAGAAAAGAAACGAGAGATGCTATC
query       CTACCTGACAGTTGAAAACTTACGGACGCAGGATGAAGAAAAGAAACGAGAGATGCTATC

template    AGATAGCTTTGTCACTTTGCAAAACCGACTATTGGATAACTCAGCAAAACTGGAACAAAT
query       AGATAGCTTTGTCACTTTGCAAAACCGACTATTGGATAACTCAGCAAAACTGGAACAAAT

template    TTCTACGCTGATTGATGGTTATCTGGTAAGCAGTAACGAAAATCTCAAAAAATTATCTGA
query       TTCTACGCTGATTGATGGTTATCTGGTAAGCAGTAACGAAAATCTCAAAAAATTATCTGA

template    CGGTGTAATAACAATTACCTCTCGTTTATCTGAAGGTAATATCCTGCTTGCTGATAATAA
query       CGGTGTAATAACAATTACCTCTCGTTTATCTGAAGGTAATATCCTGCTTGCTGATAATAA

template    TGCACGACTGGAGGCAATGAGTACAATACAAAATATTATAGATAAAAAGAATGATTCAAT
query       TGCACGACTGGAGGCAATGAGTACAATACAAAATATTATAGATAAAAAGAATGATTCAAT

template    AATGACATCAGTTGATAAATGTTATCAGGAATCTCTTTCACATGGTAAGACCATAAACGA
query       AATGACATCAGTTGATAAATGTTATCAGGAATCTCTTTCACATGGTAAGACCATAAACGA

template    TATTGCCGCTGGCAGTGCCGATATTTCTCATACTCTCGATGGGCTGAGAAAAGAAATGGA
query       TATTGCCGCTGGCAGTGCCGATATTTCTCATACTCTCGATGGGCTGAGAAAAGAAATGGA

template    TGAGGATATGAATAATGTTCATTTAGCGCTATCCGATCTATCGGCTACTGATAAAAAGAT
query       TGAGGATATGAATAATGTTCATTTAGCGCTATCCGATCTATCGGCTACTGATAAAAAGAT

template    TATTGCCAATACTAAAGAGATTAGTGCTGAAATGGTTAGCTACCGTGATACCTATATGCC
query       TATTGCCAATACTAAAGAGATTAGTGCTGAAATGGTTAGCTACCGTGATACCTATATGCC

template    ATTAATGGAAAAAATAACATCTATGCATCAGGAAATAGTAAAGCAACGTTTGTTAAACAA
query       ATTAATGGAAAAAATAACATCTATGCATCAGGAAATAGTAAAGCAACGTTTGTTAAACAA

template    GGAGGAAAAAAATGAAGATTAAGATGTTTTTTCTGACGACTGCTTTTATTACACAAAGCA
query       GGAGGAAAAAAATGAAGATTAAGATGTTTTTTCTGACGACTGCTTTTATTACACAAAGCA

template    CATACGCCAGCGAGCTTCCGGTCATTCCTCTAAGAGATTTAGTTAATGCCGCATTAACGC
query       CATACGCCAGCGAGCTTCCGGTCATTCCTCTAAGAGATTTAGTTAATGCCGCATTAACGC

template    ATCAGCCCTCTGTTGCTGTTTCATATTATGAGACTGAAAAGAAAAACAGTGACTTAGATC
query       ATCAGCCCTCTGTTGCTGTTTCATATTATGAGACTGAAAAGAAAAACAGTGACTTAGATC

template    TTTCACGGGCAGCACTTTATCCTACCCTGGATCTAACATCTGGCCTTAATAATAACAGGA
query       TTTCACGGGCAGCACTTTATCCTACCCTGGATCTAACATCTGGCCTTAATAATAACAGGA

template    AAGAGTCATCAGGTACCGAGAGGAACGTTGAAAATAAGGTGTCTTTATCATACCGAATAA
```

```
query      AAGAGTCATCAGGTACCGAGAGGAACGTTGAAAATAAGGTGTCTTTATCATACCGAATAA

template   CGGATTTTGGAGTGAGGGGGGCTAATATCAGGAAATCTGAATATGAAAGAGATAATAGTA
query      CGGATTTTGGAGTGAGGGGGGCTAATATCAGGAAATCTGAATATGAAAGAGATAATAGTA

template   AAACTGACTATGAAAAAACAAAGAATATAGTGTCTCAGGAGGTTGTGACAACCTATTACA
query      AAACTGACTATGAAAAAACAAAGAATATAGTGTCTCAGGAGGTTGTGACAACCTATTACA

template   ACATCAGTAAATATCGTGAAATGATTGATGGCGTAAATCTGGAGAAAGAGTTTTATAAAA
query      ACATCAGTAAATATCGTGAAATGATTGATGGCGTAAATCTGGAGAAAGAGTTTTATAAAA

template   AGATGCTGGAAACTTTTTCGTTGCTGGTATCTTCCGGTGTAGCTATGCAATCTGATATGC
query      AGATGCTGGAAACTTTTTCGTTGCTGGTATCTTCCGGTGTAGCTATGCAATCTGATATGC

template   GTAAAGTACAAGTATCTATCGATGCATTAAATACCAGAAGCATTATGTATCAGTCGATGT
query      GTAAAGTACAAGTATCTATCGATGCATTAAATACCAGAAGCATTATGTATCAGTCGATGT

template   TGGATGATGAAATGTATAAAATGCAGAATATGACTGGTCTGAATTTATCGCCAGTTCAGA
query      TGGATGATGAAATGTATAAAATGCAGAATATGACTGGTCTGAATTTATCGCCAGTTCAGA

template   TTCAAAGCGATGAAAAATTCAACCTCTTCAAAAAATATATCTTTGTCGAAAGCCCTGAAA
query      TTCAAAGCGATGAAAAATTCAACCTCTTCAAAAAATATATCTTTGTCGAAAGCCCTGAAA

template   AACTTATGGACATGGTGATGAAATACAACGATGACTATAAGATGCTTGTCAATACCCGAA
query      AACTTATGGACATGGTGATGAAATACAACGATGACTATAAGATGCTTGTCAATACCCGAA

template   AAGCCGCGACCGAAGATATTAATGCCGCAAAATCATCCTATTTTCCGACTGTAGATCTTG
query      AAGCCGCGACCGAAGATATTAATGCCGCAAAATCATCCTATTTTCCGACTGTAGATCTTG

template   TTTCCAGCTATGTACAGAATAACCCGAGCGGTAGTGCCAAAAAAAGTGATTATGAAGATG
query      TTTCCAGCTATGTACAGAATAACCCGAGCGGTAGTGCCAAAAAAAGTGATTATGAAGATG

template   AATTTAAAACGGGTATCAATGTCAGTTTCAACATTTTTAATGGGTTCAGAAATTCAGCCC
query      AATTTAAAACGGGTATCAATGTCAGTTTCAACATTTTTAATGGGTTCAGAAATTCAGCCC

template   AGGAAAGAAAAATGGTGGCAAGTTACTCGCAGGCTAAGCTGCAAATTGACGATTTTTTGA
query      AGGAAAGAAAAATGGTGGCAAGTTACTCGCAGGCTAAGCTGCAAATTGACGATTTTTTGA

template   TTAAAACGCGTTATAACATTGATTCACAACTTTCAAGATACGCCGCTGCAAAAGAGACTT
query      TTAAAACGCGTTATAACATTGATTCACAACTTTCAAGATACGCCGCTGCAAAAGAGACTT

template   ACTCAGTGGCGGAACGTTCACATACAAACGCGTTACAGCTTACTGAATTATATGAGCAGG
query      ACTCAGTGGCGGAACGTTCACATACAAACGCGTTACAGCTTACTGAATTATATGAGCAGG

template   AGTTTCAGTTAGGGCAAAAAAGTTTGCTTGATTTAATTTCAAGCCGTAATGAAGCATTTC
query      AGTTTCAGTTAGGGCAAAAAAGTTTGCTTGATTTAATTTCAAGCCGTAATGAAGCATTTC

template   AGGCATATGTAAGCATGATCGACAGCAAATATAGCCTGTATATTTTAAAGCTACAGCAAC
query      AGGCATATGTAAGCATGATCGACAGCAAATATAGCCTGTATATTTTAAAGCTACAGCAAC

template   TCTCATTGATTTTTCATTTAATGGATTATTTAAAAGGAAATACTGAAAGTGAGTTAAATG
query      TCTCATTGATTTTTCATTTAATGGATTATTTAAAAGGAAATACTGAAAGTGAGTTAAATG

template   TAATGAAATGAATAGAAGACAAAGCGATCATCTCATGATGATAATTATTTCTTTGACGAT
query      TAATGAAATGAATAGAAGACAAAGCGATCATCTCATGATGATAATTATTTCTTTGACGAT

template   CTTAATTATTATCCTTACCTACTTTATAGAAATTAATTCAGTGGTTCATGGTCAGGGCGT
query      CTTAATTATTATCCTTACCTACTTTATAGAAATTAATTCAGTGGTTCATGGTCAGGGCGT

template   TATCACTACTAAAGATAATGCTCAGTTAATATCTCTTTCTAAAGGAGGGACGATACAAGA
query      TATCACTACTAAAGATAATGCTCAGTTAATATCTCTTTCTAAAGGAGGGACGATACAAGA

template   TATTTATGTAGCCGAGGGTGATACTGTAAAAAAAGGAGAACTCCTTGCAAAGGTCGTTAA
query      TATTTATGTAGCCGAGGGTGATACTGTAAAAAAAGGAGAACTCCTTGCAAAGGTCGTTAA

template   CCTTGATCTGCAAAAAGAATATCAAAGGTATAGAACTCAAAAAGGGTATCTGGATAAAGA
query      CCTTGATCTGCAAAAAGAATATCAAAGGTATAGAACTCAAAAAGGGTATCTGGATAAAGA

template   TGTTAACGAAATATCTTTCATTCTTGATAAAGAAAATGAGAGTGGGTTGATTACCCTGGA
query      TGTTAACGAAATATCTTTCATTCTTGATAAAGAAAATGAGAGTGGGTTGATTACCCTGGA
```

template TGGCACCCGTTCTTTAAGCAATAAAGAGGTAAAAGCGAATATTGAATTAGTGCATAGTCA
query TGGCACCCGTTCTTTAAGCAATAAAGAGGTAAAAGCGAATATTGAATTAGTGCATAGTCA

template GATAAGAGCTAAAGAGTTAAAAAAAACCTCTCTTGATTCTGAGATTAGCGGATTACAAGA
query GATAAGAGCTAAAGAGTTAAAAAAAACCTCTCTTGATTCTGAGATTAGCGGATTACAAGA

template GAAGCTGAGTTCGAAAGAAAAAGAACTCGCATTGCTTGCCGAAGAAATAAATATTCTTTC
query GAAGCTGAGTTCGAAAGAAAAAGAACTCGCATTGCTTGCCGAAGAAATAAATATTCTTTC

template CCCACTGGTAAAAAAAGGAATTAGCCCATATACCAATTTTCTTAACAAGAAACAGGCGTA
query CCCACTGGTAAAAAAAGGAATTAGCCCATATACCAATTTTCTTAACAAGAAACAGGCGTA

template TATAAAAGTTAAGTCTGAAATTAATGATATTGAAAGTAGCATCACTTTAAAAAAAGATGA
query TATAAAAGTTAAGTCTGAAATTAATGATATTGAAAGTAGCATCACTTTAAAAAAAGATGA

template TATTGAGTTGGTTGTTAATGATATTGAGGCGCTTAATAATGAATTGCGACTATCTTTATC
query TATTGAGTTGGTTGTTAATGATATTGAGGCGCTTAATAATGAATTGCGACTATCTTTATC

template TAAAATAATATCTAAAAATCTTCAGGAACTTGAGGTTGTTAACTCTACATTAAAAGTAAT
query TAAAATAATATCTAAAAATCTTCAGGAACTTGAGGTTGTTAACTCTACATTAAAAGTAAT

template AGAGAAGCAGATAAATGAGGAGGATATCTATTCACCGGTTGACGGTGTAATTTATAAAAT
query AGAGAAGCAGATAAATGAGGAGGATATCTATTCACCGGTTGACGGTGTAATTTATAAAAT

template CAATAAAAGTGCCACTACTCATGGTGGTGTGATTCAGGCGGCGGACTTACTTTTTGAAAT
query CAATAAAAGTGCCACTACTCATGGTGGTGTGATTCAGGCGGCGGACTTACTTTTTGAAAT

template AAAACCAAAAGTAAGGACTATGCTGGCCGATGTGAAAATATTACCCAAATACCGTGACCA
query AAAACCAAAAGTAAGGACTATGCTGGCCGATGTGAAAATATTACCCAAATACCGTGACCA

template AATATATGTAGATGAAGCCGTTAAACTGGATGTGCAGTCAATTATCCAACCAAAGATAAA
query AATATATGTAGATGAAGCCGTTAAACTGGATGTGCAGTCAATTATCCAACCAAAGATAAA

template ATCGTATAATGCGACTATCGATAATATTAGCCCTGATTCCTATGAGGAAAATACCGGAGG
query ATCGTATAATGCGACTATCGATAATATTAGCCCTGATTCCTATGAGGAAAATACCGGAGG

template AACAATTCAGCGTTATTATAAAGTAATTATTGCATTCGATGTTAATGAAGATGATTTACG
query AACAATTCAGCGTTATTATAAAGTAATTATTGCATTCGATGTTAATGAAGATGATTTACG

template GTGGTTAAAACCAGGTATGACTGTTGACGCCAGTGTAATTACCGGAAAACACAGCATTAT
query GTGGTTAAAACCAGGTATGACTGTTGACGCCAGTGTAATTACCGGAAAACACAGCATTAT

template GGAATACCTGTTATCTCCCTTGATGAAAGGCGTGGACAAAGCCTTTTCAGAACCAGTTAA
query GGAATACCTGTTATCTCCCTTGATGAAAGGCGTGGACAAAGCCTTTTCAGAACCAGTTAA

template TACTAAACGATTAGATACACCTTGAGAGTGAATATAATATTATGGGAAATAAAAGCATAC
query TACTAAACGATTAGATACACCTTGAGAGTGAATATAATATTATGGGAAATAAAAGCATAC

template AAAAGTTTTTTGCCGATCAAAATTCTGTAATTGATTTATCTTCTTTGGGTAATGCCAAAG
query AAAAGTTTTTTGCCGATCAAAATTCTGTAATTGATTTATCTTCTTTGGGTAATGCCAAAG

template GCGCAAAAGTTTCTCTTTCCGGGCCAGACATGAACATTACCACGCCGCGTGGTTCAGTGA
query GCGCAAAAGTTTCTCTTTCCGGGCCAGACATGAACATTACCACGCCGCGTGGTTCAGTGA

template TCATTGTCAATGGCGCTCTTTATTCAAGTATCAAAGGCAATAACCTCGCTGTTAAATTTA
query TCATTGTCAATGGCGCTCTTTATTCAAGTATCAAAGGCAATAACCTCGCTGTTAAATTTA

template AAGATAAGACTATTACCGGCGCTAAAATTCTGGGCAGCGTAGATTTAAAAGATATTCAAC
query AAGATAAGACTATTACCGGCGCTAAAATTCTGGGCAGCGTAGATTTAAAAGATATTCAAC

template TGGAGAGAATTGACAGCTCATTGGTTGATTCTGCTCAGGTAGAAAAGAAAGGTAATGGCA
query TGGAGAGAATTGACAGCTCATTGGTTGATTCTGCTCAGGTAGAAAAGAAAGGTAATGGCA

template AACGACGAAATAAGAAGGAAGAAGAGGAATTAAAAAAGCAGCTTGACGATGCTGAAAACG
query AACGACGAAATAAGAAGGAAGAAGAGGAATTAAAAAAGCAGCTTGACGATGCTGAAAACG

template CAAAGAAAGAAGCTGATAAGGCGAAGGAAGAAGCAGAGAAAGCTAAGGAGGCTGCAGAAA
query CAAAGAAAGAAGCTGATAAGGCGAAGGAAGAAGCAGAGAAAGCTAAGGAGGCTGCAGAAA

```
template   AAGCGCTCAATGAAGCGTTTGAAGTACAGAACTCGTCAAAGCAAATTGAAGAAATGCTGC
query      AAGCGCTCAATGAAGCGTTTGAAGTACAGAACTCGTCAAAGCAAATTGAAGAAATGCTGC

template   AGAACTTTTTGGCTGACAATGTAGCAAAAGACAATCTGGCTCAGCAAAGCGATGCTTCCC
query      AGAACTTTTTGGCTGACAATGTAGCAAAAGACAATCTGGCTCAGCAAAGCGATGCTTCCC

template   AGCAAAATACACAGGCTAAAGCAACGCAGGCTTCTAAACAGAACGATGCTGAAAAAGTTC
query      AGCAAAATACACAGGCTAAAGCAACGCAGGCTTCTAAACAGAACGATGCTGAAAAAGTTC

template   TTCCTCAACCTATTAATAAAAATACCAGTACTGGCAAAAGTAATAGCAGTAAAAATGAGG
query      TTCCTCAACCTATTAATAAAAATACCAGTACTGGCAAAAGTAATAGCAGTAAAAATGAGG

template   AAAATAAGCTCGATGCCGAGTCTGTTAAAGAGCCGCTTAAAGTCACATTAGCGCTTGCGG
query      AAAATAAGCTCGATGCCGAGTCTGTTAAAGAGCCGCTTAAAGTCACATTAGCGCTTGCGG

template   CCGAGAGTAACAGCGGTAGCAAAGATGATAGTATAACTAATTTTACCAAACCTCAGTTTG
query      CCGAGAGTAACAGCGGTAGCAAAGATGATAGTATAACTAATTTTACCAAACCTCAGTTTG

template   TAGGTAGCACTGCTCCCAATGCCACGGTTATTATTAAAATTAATGGTATTGCTGTCGGTC
query      TAGGTAGCACTGCTCCCAATGCCACGGTTATTATTAAAATTAATGGTATTGCTGTCGGTC

template   AGGCTGTAGCGGATAGTTTGGGTAACTTCACCTTTACAGCGCCTGAAACATTGACTGATG
query      AGGCTGTAGCGGATAGTTTGGGTAACTTCACCTTTACAGCGCCTGAAACATTGACTGATG

template   GAACATATAATCTGGAGGCAGAGGCCAAGACTGCTGATGGGAGCGGTAGCGCCAAACTTG
query      GAACATATAATCTGGAGGCAGAGGCCAAGACTGCTGATGGGAGCGGTAGCGCCAAACTTG

template   TCATTACTATCGATTCCGTTACCGATAAACCAACATTTGAACTTTCGCCTGAAAGTAGTG
query      TCATTACTATCGATTCCGTTACCGATAAACCAACATTTGAACTTTCGCCTGAAAGTAGTG

template   TGTCCGGTCATAAGGGCTTAACGCCGACCTTGACGCCTTCAATTGTTGGTACGGCGGAAG
query      TGTCCGGTCATAAGGGCTTAACGCCGACCTTGACGCCTTCAATTGTTGGTACGGCGGAAG

template   AGAATGCTAAGGTTGACATTTATGTAGATAATAAACTGGTTGCCAGCGTTGATGTCGATA
query      AGAATGCTAAGGTTGACATTTATGTAGATAATAAACTGGTTGCCAGCGTTGATGTCGATA

template   AAGATGGAAACTGGAGTTATGAATTTAAGGATAATGAATTATCTGAGGGCGAAAATAGTA
query      AAGATGGAAACTGGAGTTATGAATTTAAGGATAATGAATTATCTGAGGGCGAAAATAGTA

template   TAAAAGTCGTTGCTGTAGATAAAGCAGGTAATAAAAACGAAACGACGGATAGTATCATAA
query      TAAAAGTCGTTGCTGTAGATAAAGCAGGTAATAAAAACGAAACGACGGATAGTATCATAA

template   CCGACACCATTGCTCCAGAAAAGCCGACGATTGAGCTGGATGATAGTAGTGATTCCGGCA
query      CCGACACCATTGCTCCAGAAAAGCCGACGATTGAGCTGGATGATAGTAGTGATTCCGGCA

template   TTAAAAATGACAACATTACAAATAGCACCCTGCCAACATTTATTGGTGTGGCGGAACCCG
query      TTAAAAATGACAACATTACAAATAGCACCCTGCCAACATTTATTGGTGTGGCGGAACCCG

template   GTTCTACAGTCTCTATTTATCTTGGACTTAAACATCTTGGTGAGGTCATTGTTGCTAAAG
query      GTTCTACAGTCTCTATTTATCTTGGACTTAAACATCTTGGTGAGGTCATTGTTGCTAAAG

template   ATGGGACATGGAGCTATACGCTTACTACGCCGCTCAAGGATGGCGAATACAATATAACAG
query      ATGGGACATGGAGCTATACGCTTACTACGCCGCTCAAGGATGGCGAATACAATATAACAG

template   CAACAGCTACTGATATTGCCGGGCATACCTCAGCGACGGCAAATCTGCCTTTTACTATTG
query      CAACAGCTACTGATATTGCCGGGCATACCTCAGCGACGGCAAATCTGCCTTTTACTATTG

template   ATACACGTATCAGCTATTTCAGCGCTGAGATTGAAACGACGAATGATAGCGGTATTGTCG
query      ATACACGTATCAGCTATTTCAGCGCTGAGATTGAAACGACGAATGATAGCGGTATTGTCG

template   GAGATAACGTTACTAACAATACTCGCCCAACCTTTACAGGTAAAACTGAGCCAAATGCTA
query      GAGATAACGTTACTAACAATACTCGCCCAACCTTTACAGGTAAAACTGAGCCAAATGCTA

template   TTATCAGTGTCATAAATAGTGAGACTGGCGAAGAGGTTATTTTTAAAGCGAATGACAAGG
query      TTATCAGTGTCATAAATAGTGAGACTGGCGAAGAGGTTATTTTTAAAGCGAATGACAAGG

template   GCGAATGGACGTTCAATTTCACTTCCGACTCAGTGGAAGGGATTAACAATCTTACGTTCA
query      GCGAATGGACGTTCAATTTCACTTCCGACTCAGTGGAAGGGATTAACAATCTTACGTTCA

template   CTGTTGAAGATGTCGCTGGCAACAAAAAGGATTTTTCCTTTAGTTACGTTATTGATACTA
```

```
query       CTGTTGAAGATGTCGCTGGCAACAAAAAGGATTTTTCCTTTAGTTACGTTATTGATACTA

template    TTGCCCCTGTACCTCCGACGGTTTCTTTGGAGGATTATGTTGTTTTGCCGAATGGTATAA
query       TTGCCCCTGTACCTCCGACGGTTTCTTTGGAGGATTATGTTGTTTTGCCGAATGGTATAA

template    TTTTATCAGGGAATGATTTACCGGCTTTAGTCGGTACGGCAGAGCCAAAGTCTACCATCT
query       TTTTATCAGGGAATGATTTACCGGCTTTAGTCGGTACGGCAGAGCCAAAGTCTACCATCT

template    TATTGATGCGAGATGGTAAATTATATGACAGCATTGAGGTTGACTCAAACGGGACCTGGA
query       TATTGATGCGAGATGGTAAATTATATGACAGCATTGAGGTTGACTCAAACGGGACCTGGA

template    ATTATCAGTTTAGTAATAAATTTCTTCAGGGCGCCTATGATATTGAAATCATTTCTCAGG
query       ATTATCAGTTTAGTAATAAATTTCTTCAGGGCGCCTATGATATTGAAATCATTTCTCAGG

template    ATGCCGCCGGCAATAAATCCTCTACTGTTAAATATTCTTTTACTATTCAAACTGAAGTTG
query       ATGCCGCCGGCAATAAATCCTCTACTGTTAAATATTCTTTTACTATTCAAACTGAAGTTG

template    TACCTCCAAAAGCGGAACTCGATGCCAGTGATGATTCCGGTGCAAAAGGCGACTGGATTA
query       TACCTCCAAAAGCGGAACTCGATGCCAGTGATGATTCCGGTGCAAAAGGCGACTGGATTA

template    CCAATAAACATAATGCTCTGACATTACTGGGAACAGCGGATAGGTTTGCTACCGTAAATA
query       CCAATAAACATAATGCTCTGACATTACTGGGAACAGCGGATAGGTTTGCTACCGTAAATA

template    TCCTTATTGACGGTAAAACGATAGGCGTGACGACTGCGGATGCAGACGGTAACTGGAATT
query       TCCTTATTGACGGTAAAACGATAGGCGTGACGACTGCGGATGCAGACGGTAACTGGAATT

template    TTGATATTTCCAGAAATCTGTCTGACAATGTTTATAAGATTACGGTTGAATCCATCGATC
query       TTGATATTTCCAGAAATCTGTCTGACAATGTTTATAAGATTACGGTTGAATCCATCGATC

template    CTTTAGGAAGAACGTCATCTGTAGATTATCAGCTTACCATTGATAGCTTTACGCCGATCC
query       CTTTAGGAAGAACGTCATCTGTAGATTATCAGCTTACCATTGATAGCTTTACGCCGATCC

template    CTACTGTTATGTTGCATGATAGCGCTGACTCTGGCGTTAAAGGCGATATGATTACTAAAA
query       CTACTGTTATGTTGCATGATAGCGCTGACTCTGGCGTTAAAGGCGATATGATTACTAAAA

template    TTAATACACCGTTGTTTACCGGGATGGCTGAAGCTAATGCTAAGGTTTCCATCTATGTTG
query       TTAATACACCGTTGTTTACCGGGATGGCTGAAGCTAATGCTAAGGTTTCCATCTATGTTG

template    ACGGTGTGTTAAGTGGTGAGGCTATTGCTGGCGATGATGGTGTATGGAATTTTCAATTTA
query       ACGGTGTGTTAAGTGGTGAGGCTATTGCTGGCGATGATGGTGTATGGAATTTTCAATTTA

template    CCACAGCGTTATCCGATGGCTCGCATGACGTAACGGTAAAGGTAGAAGATATTGCCGGTA
query       CCACAGCGTTATCCGATGGCTCGCATGACGTAACGGTAAAGGTAGAAGATATTGCCGGTA

template    ATACTGCCTCCTCATCAGCGTATAATTTCCAAATCGTAACGCAAACGCAAAAACCAACAA
query       ATACTGCCTCCTCATCAGCGTATAATTTCCAAATCGTAACGCAAACGCAAAAACCAACAA

template    TAGAGTTGGTCAACGATACGGGGGTTGATAATACAGACCATATTATTAATGAAAAGAATC
query       TAGAGTTGGTCAACGATACGGGGGTTGATAATACAGACCATATTATTAATGAAAAGAATC

template    CTGCACTGACAGGAACCGCTGCACCCTATTCAACGGTTAAACTCTATATTGATGGTGCAC
query       CTGCACTGACAGGAACCGCTGCACCCTATTCAACGGTTAAACTCTATATTGATGGTGCAC

template    TGATCGCTGAGGTCAGAACAAATAAAGATGGCAGATGGGAGTATACCCTGAAAGCCGATC
query       TGATCGCTGAGGTCAGAACAAATAAAGATGGCAGATGGGAGTATACCCTGAAAGCCGATC

template    AAGGTTTGGTTGATGGCGATCATAGAATAACCGCTTCAGTTGAAGATATCGCTGGCAACA
query       AAGGTTTGGTTGATGGCGATCATAGAATAACCGCTTCAGTTGAAGATATCGCTGGCAACA

template    TTGCTCATTCGGATCCTTTCTTAATTAGCGTCGATACTGCTATTTCAATACCGATAGTTT
query       TTGCTCATTCGGATCCTTTCTTAATTAGCGTCGATACTGCTATTTCAATACCGATAGTTT

template    CATTGAGCCCGGATTCAGATTCGGGAATTTCAGATGATAATTTAACGAATATCGTTAAAC
query       CATTGAGCCCGGATTCAGATTCGGGAATTTCAGATGATAATTTAACGAATATCGTTAAAC

template    CTACCTTGCACCTAAAAGATATTGATCCGGACATTATCAGTGTTCAGGTATGGGATGCCA
query       CTACCTTGCACCTAAAAGATATTGATCCGGACATTATCAGTGTTCAGGTATGGGATGCCA

template    TGTCTGATACGCAGATCGGTGTTGCCACGCAACAACCTGATGGTTCATGGGCCTATACCT
query       TGTCTGATACGCAGATCGGTGTTGCCACGCAACAACCTGATGGTTCATGGGCCTATACCT
```

```
template  TTACTTCAGATTTAACGGAAGGCTTGCATCAGGTTTATGTCAAGGTTGAGGACATTGCGG
query     TTACTTCAGATTTAACGGAAGGCTTGCATCAGGTTTATGTCAAGGTTGAGGACATTGCGG

template  GTAATAAAGCGAACAGCGCGATATTCGATTTTACTATCGATACCACAGTATCAACGCCGG
query     GTAATAAAGCGAACAGCGCGATATTCGATTTTACTATCGATACCACAGTATCAACGCCGG

template  TGATTTCCCTGCTTTCTAAGGATGATACGGGGGTTACAGGCGATAACCTGACCAATATCA
query     TGATTTCCCTGCTTTCTAAGGATGATACGGGGGTTACAGGCGATAACCTGACCAATATCA

template  ATAAGCCAGGTTTTGCTATTTCCGGTGTTGATGCCGATGCGCATCGGGTCGTCGTACAGG
query     ATAAGCCAGGTTTTGCTATTTCCGGTGTTGATGCCGATGCGCATCGGGTCGTCGTACAGG

template  TGATGCACAATGGCGTGAGCGAAGAGATCGAACTTTCCCACCTCAATGGTAGTTGGTTAT
query     TGATGCACAATGGCGTGAGCGAAGAGATCGAACTTTCCCACCTCAATGGTAGTTGGTTAT

template  TTATACCAGGGAATACGTGGGCGGATGGCAGCTACACGTTAACGGTGAAAGTAGAAGATA
query     TTATACCAGGGAATACGTGGGCGGATGGCAGCTACACGTTAACGGTGAAAGTAGAAGATA

template  AGGCAGGAAATACCAACTACTCAGCGCCGCTGACGGTCGTTATCGATACCCAAATCGCCA
query     AGGCAGGAAATACCAACTACTCAGCGCCGCTGACGGTCGTTATCGATACCCAAATCGCCA

template  TTGATGGGGTGGAACTGGTCAACGATAGCGGCGTGAAAGGCGATAATATGACCAACGACG
query     TTGATGGGGTGGAACTGGTCAACGATAGCGGCGTGAAAGGCGATAATATGACCAACGACG

template  ACCGTCCCCACTTTCGTGTGACGGTACCTACGGATGTCAATGAAGTCCGTCTGAGCATTG
query     ACCGTCCCCACTTTCGTGTGACGGTACCTACGGATGTCAATGAAGTCCGTCTGAGCATTG

template  ACGGTGGTAATTCGTGGGTTCAGGCAACTCCGGGCGTGGCAGGAAGCTGGGAGTATATCT
query     ACGGTGGTAATTCGTGGGTTCAGGCAACTCCGGGCGTGGCAGGAAGCTGGGAGTATATCT

template  GGCCGACAGACCTGGCAGATGGTCAATACACGCTAACGGTGGAAGCGACTGATAAAGCAG
query     GGCCGACAGACCTGGCAGATGGTCAATACACGCTAACGGTGGAAGCGACTGATAAAGCAG

template  GCAATACAGTGACGAAGACCATCGATTTCGCGGTGGATACCACGCTGTCAGTGCCGGTCA
query     GCAATACAGTGACGAAGACCATCGATTTCGCGGTGGATACCACGCTGTCAGTGCCGGTCA

template  TCGTACTGGATAGCGCGGACGACACCGGCATCCAGGGCGATAACATGACGAATAGCACCC
query     TCGTACTGGATAGCGCGGACGACACCGGCATCCAGGGCGATAACATGACGAATAGCACCC

template  AGCCGACATTTGCCCTGCAGCATATTGATGATGATGCCGTTCGCGTTACGGTCAGCGTGG
query     AGCCGACATTTGCCCTGCAGCATATTGATGATGATGCCGTTCGCGTTACGGTCAGCGTGG

template  AGCATGGCGGCGTCACCACCACATTTGACGCCACGAAAGGCACAGGCGGATGGACCTTTA
query     AGCATGGCGGCGTCACCACCACATTTGACGCCACGAAAGGCACAGGCGGATGGACCTTTA

template  CGCCGCCGACATCATGGGCGGATGGTGATTATACCCTGAGTGTGTCAGTCGAAGATAAAG
query     CGCCGCCGACATCATGGGCGGATGGTGATTATACCCTGAGTGTGTCAGTCGAAGATAAAG

template  CGGGGAACACCAGCCATTCTGCATCGCTGACGGTGACGGTGGACACGCAAATCGCCATTA
query     CGGGGAACACCAGCCATTCTGCATCGCTGACGGTGACGGTGGACACGCAAATCGCCATTA

template  ATAACATTGAACTGGTCAATGACAGCGGTATTCCCGACGATAATCTGACTAATAATGTGC
query     ATAACATTGAACTGGTCAATGACAGCGGTATTCCCGACGATAATCTGACTAATAATGTGC

template  GTCCGCACTTCCAGGTGACGGTACCGACGGATGTCAACGTGGTGCGCCTGAGCATTGACG
query     GTCCGCACTTCCAGGTGACGGTACCGACGGATGTCAACGTGGTGCGCCTGAGCATTGACG

template  GCGGCAAGACGTGGTTCAACGCTACCCAGAGCGCGACGCCAGGCGTCTGGGATTATATCT
query     GCGGCAAGACGTGGTTCAACGCTACCCAGAGCGCGACGCCAGGCGTCTGGGATTATATCT

template  GGCCGGATGATGTGGCCGACGGAGGCTATACCCTGACGGTAGAAGCGACCGATGAGGCGG
query     GGCCGGATGATGTGGCCGACGGAGGCTATACCCTGACGGTAGAAGCGACCGATGAGGCGG

template  GAAATAAGGCAACACAGACGCTCGATTTCACCATCGATACCACTCTGTCTGTGCCGACCC
query     GAAATAAGGCAACACAGACGCTCGATTTCACCATCGATACCACTCTGTCTGTGCCGACCC

template  TCTCGCTGGACAGCGCAGATGACAGCGGCATCGCGGGCGATAATATCACCAATGTTAAAA
query     TCTCGCTGGACAGCGCAGATGACAGCGGCATCGCGGGCGATAATATCACCAATGTTAAAA
```

```
template    CGCCGGGCTTTACCCTCAACAATATTGATACCGATGTCAGCCGGGTGATAGTGGAGGTAA
query       CGCCGGGCTTTACCCTCAACAATATTGATACCGATGTCAGCCGGGTGATAGTGGAGGTAA

template    TGCACAATGGCATTAAACAGGAGGTGCCACTGGTTCAGACCGGCGGACAGTGGCGCTTTG
query       TGCACAATGGCATTAAACAGGAGGTGCCACTGGTTCAGACCGGCGGACAGTGGCGCTTTG

template    CGCCGACCAGCGACTGGGCGGACGGCGACTATATCCTGACGGTGAAGGTAGAAGATAGGG
query       CGCCGACCAGCGACTGGGCGGACGGCGACTATATCCTGACGGTGAAGGTAGAAGATAGGG

template    CCGGAAATGTGAAGCAGTCCGCGCCGTTGACGGTGACAGTAGACACGCATATCGCCATTG
query       CCGGAAATGTGAAGCAGTCCGCGCCGTTGACGGTGACAGTAGACACGCATATCGCCATTG

template    ACCGTATTGAACTGGTTAACGACAGCGGTATCCCCGGCGATAATCTGACCAATGAAGCGC
query       ACCGTATTGAACTGGTTAACGACAGCGGTATCCCCGGCGATAATCTGACCAATGAAGCGC

template    GCCCGCACTTTCAGGTGACAGTACCGGCGGATGTTAACGGTGTAAGACTGAGTATTGATG
query       GCCCGCACTTTCAGGTGACAGTACCGGCGGATGTTAACGGTGTAAGACTGAGTATTGATG

template    GCGGCAAAACGTGGTTTGACGCCACGCAGAGCGCGACGTCGGGCGTCTGGGATTACACCT
query       GCGGCAAAACGTGGTTTGACGCCACGCAGAGCGCGACGTCGGGCGTCTGGGATTACACCT

template    GGCTGACGAATGTGGCTAACGGCCCTCACACCCTGATGGTGGAAGCGTCCGACAAGGCGG
query       GGCTGACGAATGTGGCTAACGGCCCTCACACCCTGATGGTGGAAGCGTCCGACAAGGCGG

template    GAAACAAAACGACGCAGAAACTGGACTTCACCATCGATACCATTCTGTCAGAACCGACGA
query       GAAACAAAACGACGCAGAAACTGGACTTCACCATCGATACCATTCTGTCAGAACCGACGA

template    TTACCCTGGACAGCGCGGATGATAGCGCCGCTGGCGATAACATCACCAACGTTAAGATGC
query       TTACCCTGGACAGCGCGGATGATAGCGCCGCTGGCGATAACATCACCAACGTTAAGATGC

template    CAGGCTTTACCCTCGGTAATATCGACGCCGACGTGACCAAAGTGGTGGTGACGGTGGCGC
query       CAGGCTTTACCCTCGGTAATATCGACGCCGACGTGACCAAAGTGGTGGTGACGGTGGCGC

template    ATGATGGTAAGAACCAACAGATAGAGTTGATTAAGAACGGCGGTGTGTGGCGCTTTACGC
query       ATGATGGTAAGAACCAACAGATAGAGTTGATTAAGAACGGCGGTGTGTGGCGCTTTACGC

template    CGGGCGCAGCCTGGACCGATGGCGACTATACGTTGACGGTAAAGGTAGAAGATAAGGCGG
query       CGGGCGCAGCCTGGACCGATGGCGACTATACGTTGACGGTAAAGGTAGAAGATAAGGCGG

template    GTAATACAAATTATTCTGCGCCGCTGACGGTGACTATCGATACGCAAACGTCTATTGATC
query       GTAATACAAATTATTCTGCGCCGCTGACGGTGACTATCGATACGCAAACGTCTATTGATC

template    GCATTGAGCTTCTTAATGACACGGGTATTGTCGGGGATAACCTGACCAATGAAGCACGTC
query       GCATTGAGCTTCTTAATGACACGGGTATTGTCGGGGATAACCTGACCAATGAAGCACGTC

template    CACAGTTTCATATTACGGTACCGACGGACGTGAACTCTGTGCAACTGAGTCTTGATGGCG
query       CACAGTTTCATATTACGGTACCGACGGACGTGAACTCTGTGCAACTGAGTCTTGATGGCG

template    GCATCAACTGGGTTAACGCAACGCTGACGTCTGACGGCGTTTGGGAGTATATATGGCCGA
query       GCATCAACTGGGTTAACGCAACGCTGACGTCTGACGGCGTTTGGGAGTATATATGGCCGA

template    CAGATCTGGTCGAAAATACGTATACCCTGACAGTGAAAGCAACCGATGTTGCAGGCAACA
query       CAGATCTGGTCGAAAATACGTATACCCTGACAGTGAAAGCAACCGATGTTGCAGGCAACA

template    CGGCGACGGAAACGCTCAATTTTATCATTGATACCACATTGTCGACACCGACCATCACGC
query       CGGCGACGGAAACGCTCAATTTTATCATTGATACCACATTGTCGACACCGACCATCACGC

template    TGGATAGCGCAGATGATAGCGGCACCGCCAACGATAATAAGACTAACGTTAAAACGCCGG
query       TGGATAGCGCAGATGATAGCGGCACCGCCAACGATAATAAGACTAACGTTAAAACGCCGG

template    GTTTTATTATCGGCGGTATTGATTCTGACGTGACTCAGGTCGTCGTGCAGGTGATGCGCG
query       GTTTTATTATCGGCGGTATTGATTCTGACGTGACTCAGGTCGTCGTGCAGGTGATGCGCG

template    ATGGTCACAGCGAGGAGGTGGAGCTGACGCAGACTAACGGGCAGTGGCGTTTTGTACCCG
query       ATGGTCACAGCGAGGAGGTGGAGCTGACGCAGACTAACGGGCAGTGGCGTTTTGTACCCG

template    GCAGCGCGTGGACTGATGGCGACTATACGCTGACGGTAACGGTGAAAGATGAGGCGGGTA
query       GCAGCGCGTGGACTGATGGCGACTATACGCTGACGGTAACGGTGAAAGATGAGGCGGGTA

template    ATATTCGCCACTCAGCGCCGTTGACGGTCACCATCGATACGCAAATCACCATTGACCATA
```

```
query       ATATTCGCCACTCAGCGCCGTTGACGGTCACCATCGATACGCAAATCACCATTGACCATA

template    TTGAACTGGTCAATGACAGCGGTATTCCCGACGATAATCTAACTAATAATGTGCGTCCGC
query       TTGAACTGGTCAATGACAGCGGTATTCCCGACGATAATCTAACTAATAATGTGCGTCCGC

template    ACTTCCAGGTGACGGTACCGACGGATGTCAACGTGGTGCGCCTGAGCATTGACGGCGGTA
query       ACTTCCAGGTGACGGTACCGACGGATGTCAACGTGGTGCGCCTGAGCATTGACGGCGGTA

template    AGACGTGGTTCAACGCTACCCAGAGCGCGACGCCGGGCGTCTGGGATTATACCTGGCTGG
query       AGACGTGGTTCAACGCTACCCAGAGCGCGACGCCGGGCGTCTGGGATTATACCTGGCTGG

template    CTGATGTGGGAGAGGGTAAGCATACCCTGACAGTGGAGGCGACCGACAAGGCGGGAAACA
query       CTGATGTGGGAGAGGGTAAGCATACCCTGACAGTGGAGGCGACCGACAAGGCGGGAAACA

template    AAACGACGCAGCAACTGGACTTCATCATCGATACCCTACTGTCAGAACCGACTATCGTGC
query       AAACGACGCAGCAACTGGACTTCATCATCGATACCCTACTGTCAGAACCGACTATCGTGC

template    TGGACAGCACGGACGACAGCGGAACAAAAGGCGATCACCTGACCAACGTAAATAAGCCGA
query       TGGACAGCACGGACGACAGCGGAACAAAAGGCGATCACCTGACCAACGTAAATAAGCCGA

template    CGTTTTTACTGGGCAATATTGACGCAGACGCGCGGTATGTCACGGTTGAGGTACAGCATG
query       CGTTTTTACTGGGCAATATTGACGCAGACGCGCGGTATGTCACGGTTGAGGTACAGCATG

template    GCGGCACGAAAGAGGTGCTGACGGCCACCAAAGACGCGACCGGCAACTGGAGCGTGACAC
query       GCGGCACGAAAGAGGTGCTGACGGCCACCAAAGACGCGACCGGCAACTGGAGCGTGACAC

template    CGACCGGCACATGGGCAGATGGCGACTATACGCTGACAGTGAGGGTGGAAGATGAGGCGG
query       CGACCGGCACATGGGCAGATGGCGACTATACGCTGACAGTGAGGGTGGAAGATGAGGCGG

template    GGAACGAAAAACACTCAGCGTCGCTGACGGTCACTGTTGATACCCAAATCACCATTGATG
query       GGAACGAAAAACACTCAGCGTCGCTGACGGTCACTGTTGATACCCAAATCACCATTGATG

template    TTATTGAACTGGTTAATGATAACGGTATTCCCGGCGACAATATGACTAACGACGCCCATC
query       TTATTGAACTGGTTAATGATAACGGTATTCCCGGCGACAATATGACTAACGACGCCCATC

template    CGCAGTTCCGCGTGACGGTACCGGGGGACGTTAACGAAGTCAGTCTGAGCATTGACGGTG
query       CGCAGTTCCGCGTGACGGTACCGGGGGACGTTAACGAAGTCAGTCTGAGCATTGACGGTG

template    GCGTGACCTGGGTTAAGGCGACACAGAGCGCGACGCCGGGCGTCTGGAATTATACCTGGC
query       GCGTGACCTGGGTTAAGGCGACACAGAGCGCGACGCCGGGCGTCTGGAATTATACCTGGC

template    CGGGCACCGTGCCGGATGGCGACTATACGCTGAATGTGAAAGCGACTGACAATGCGGGTA
query       CGGGCACCGTGCCGGATGGCGACTATACGCTGAATGTGAAAGCGACTGACAATGCGGGTA

template    ATACGGTGACGGAGACACTCCACTTCACTATTGATACTACGTTGTCGACGCCGGTGATCG
query       ATACGGTGACGGAGACACTCCACTTCACTATTGATACTACGTTGTCGACGCCGGTGATCG

template    TACTGGATAGCGCGGACGACAGTGGTGTCCATGGCGATAACATGACCAATCACACCCAGC
query       TACTGGATAGCGCGGACGACAGTGGTGTCCATGGCGATAACATGACCAATCACACCCAGC

template    CGACATTTGCCCTGCAGCATATTGATGATGATGCCGTTCGCGTTACGGTCAGCGTAGAGC
query       CGACATTTGCCCTGCAGCATATTGATGATGATGCCGTTCGCGTTACGGTCAGCGTAGAGC

template    ATGGCGGCGTCACCACCACATTTGACGCCACGAAAGACGCAGGCGGATGGACCTTTACGC
query       ATGGCGGCGTCACCACCACATTTGACGCCACGAAAGACGCAGGCGGATGGACCTTTACGC

template    CGACAGGGGCGTGGGCGGATGGTGATTATACCCTGAGTGTGTCAGTCGAAGATAAAGCGG
query       CGACAGGGGCGTGGGCGGATGGTGATTATACCCTGAGTGTGTCAGTCGAAGATAAAGCGG

template    GGAACACCAGCCATTCTGCATCGCTGACGGTGACGGTGGACACGCAAATCGCCATTAATA
query       GGAACACCAGCCATTCTGCATCGCTGACGGTGACGGTGGACACGCAAATCGCCATTAATA

template    ACATTGAACTGGTCAATGACAGCGGTATTCCCGACGATAATCTGACTAATAACGTGCGTC
query       ACATTGAACTGGTCAATGACAGCGGTATTCCCGACGATAATCTGACTAATAACGTGCGTC

template    CGCACTTCCAGGTGACGGTACCGACTGATGTCAACGTGGTGCGCCTGAGCATTGACGGCG
query       CGCACTTCCAGGTGACGGTACCGACTGATGTCAACGTGGTGCGCCTGAGCATTGACGGCG

template    GCAAGACGTGGTTCAACGCGACCCAGAGCGCGACGCCGGGTGTCTGGGATTATACCTGGC
query       GCAAGACGTGGTTCAACGCGACCCAGAGCGCGACGCCGGGTGTCTGGGATTATACCTGGC
```

```
template  TGGCTGATGTGGGAGAGGGGAAGCATACCCTGACAGTGGAGGCGACCGACAAGGCGGGAA
query     TGGCTGATGTGGGAGAGGGGAAGCATACCCTGACAGTGGAGGCGACCGACAAGGCGGGAA

template  ACAAAACGACGCAGCAACTGGACTTCATCATCGATACCCTACTGTCAGAACCGACTATCG
query     ACAAAACGACGCAGCAACTGGACTTCATCATCGATACCCTACTGTCAGAACCGACTATCG

template  TGCTGGACAACACGGACGACAGCGGAACAAAAGGCGATAACCTGACCAACGTAAATAAGC
query     TGCTGGACAACACGGACGACAGCGGAACAAAAGGCGATAACCTGACCAACGTAAATAAGC

template  CGACGTTTTTACTGGGCAATATTGACGCAGACGCGCGGTATGTCACGGTTGAGGTGCAAC
query     CGACGTTTTTACTGGGCAATATTGACGCAGACGCGCGGTATGTCACGGTTGAGGTGCAAC

template  ATGGCGGCACGAAAGAAGTGCTGACGGCCACCAAAGGCGCGACCGGCATCTGGAGCGTGA
query     ATGGCGGCACGAAAGAAGTGCTGACGGCCACCAAAGGCGCGACCGGCATCTGGAGCGTGA

template  CACCGACCGGCACATGGGCAGATGGCGACTATACGCTGACGGTGAGGGTGGAGGATGATG
query     CACCGACCGGCACATGGGCAGATGGCGACTATACGCTGACGGTGAGGGTGGAGGATGATG

template  CGGGGAACGTAAAATACTCAGCGCCGCTGACGGTCACGGTTGACACCCAAATCACCATCG
query     CGGGGAACGTAAAATACTCAGCGCCGCTGACGGTCACGGTTGACACCCAAATCACCATCG

template  ATGTTATTGAACTGGTTAATGATAACGGTATTCCCGGCGACAACCTGACCAATGACGTTC
query     ATGTTATTGAACTGGTTAATGATAACGGTATTCCCGGCGACAACCTGACCAATGACGTTC

template  GTCCACACTTCCGCGTCACGGTGCCAGGGGATGTCAACGAGGTACGTCTGAGTATCGACG
query     GTCCACACTTCCGCGTCACGGTGCCAGGGGATGTCAACGAGGTACGTCTGAGTATCGACG

template  GCGGTAATACGTGGGTTCGTGCAACACAGGGCACGGCAGGGATCTGGGATTACACCTGGC
query     GCGGTAATACGTGGGTTCGTGCAACACAGGGCACGGCAGGGATCTGGGATTACACCTGGC

template  CGAAAGATGTGACCGACGGGCTACATACCCTGACGGTAGAAGCGACCGATAAGGCGGGAA
query     CGAAAGATGTGACCGACGGGCTACATACCCTGACGGTAGAAGCGACCGATAAGGCGGGAA

template  ATAAGACGACGCAGACGCTCGATTTTACCATTGATACCCGGCTGTCAACGCCTACCATCG
query     ATAAGACGACGCAGACGCTCGATTTTACCATTGATACCCGGCTGTCAACGCCTACCATCG

template  CTATGGATAGCAGGGACGATACAGGTGCCATTGGCGATCATATTACGAGCGTCAAAAGAC
query     CTATGGATAGCAGGGACGATACAGGTGCCATTGGCGATCATATTACGAGCGTCAAAAGAC

template  CGGGCTTTACTATTGGCAATATTGACGCCGATGCGCACTCGGTCATTTTGCGGATCACAC
query     CGGGCTTTACTATTGGCAATATTGACGCCGATGCGCACTCGGTCATTTTGCGGATCACAC

template  AGGGCGGCAATAGCCAGGAAGTGACACTAACCCAGGTTGGAGGACAGTGGCGCTTTACGC
query     AGGGCGGCAATAGCCAGGAAGTGACACTAACCCAGGTTGGAGGACAGTGGCGCTTTACGC

template  CAGATGCTGACTGGGCGGACGGTAGCTATACGCTGACGGTAGAGGTAACGGATAACGCAG
query     CAGATGCTGACTGGGCGGACGGTAGCTATACGCTGACGGTAGAGGTAACGGATAACGCAG

template  GAAACGTTCGTCAGTCCACGCCGCTGGTGGTGACGGTGGACACGCAAACCAGCATTACTG
query     GAAACGTTCGTCAGTCCACGCCGCTGGTGGTGACGGTGGACACGCAAACCAGCATTACTG

template  ATATTACATTGGTCAATGATCATGGCGTGCCTGATGACAATCTAACTAATAGCACCCGTC
query     ATATTACATTGGTCAATGATCATGGCGTGCCTGATGACAATCTAACTAATAGCACCCGTC

template  CGCAGTTTGAGATCACGGTGCCGGCGGATGTGAATTCTGTGCAACTGAGCATTGATGGGG
query     CGCAGTTTGAGATCACGGTGCCGGCGGATGTGAATTCTGTGCAACTGAGCATTGATGGGG

template  GCGCAAACTGGGTGAGCGCGACGCAGGGTATCGAAGGCGTCTGGGGCTATACCTGGCCAA
query     GCGCAAACTGGGTGAGCGCGACGCAGGGTATCGAAGGCGTCTGGGGCTATACCTGGCCAA

template  CGGATATGGGCGATGGAAAACACACCCTAACCGTCATGGTCACCGACAGAGCGGGCAATA
query     CGGATATGGGCGATGGAAAACACACCCTAACCGTCATGGTCACCGACAGAGCGGGCAATA

template  CGGCGACGCAAACGCTTGAATTTTTCATCGACACCCGGTTGTCGACGCCGACCATTGCGC
query     CGGCGACGCAAACGCTTGAATTTTTCATCGACACCCGGTTGTCGACGCCGACCATTGCGC

template  TGGATAGCACGGATGATACCGGTACGCCTGGCGATGATATGACCAATCGCACCCGACCGA
query     TGGATAGCACGGATGATACCGGTACGCCTGGCGATGATATGACCAATCGCACCCGACCGA
```

```
template  CCTTTATTCTGCAGAATATCGATTCGGATGTTATCAACGTTACAGTCAGCGTCACGCATA
query     CCTTTATTCTGCAGAATATCGATTCGGATGTTATCAACGTTACAGTCAGCGTCACGCATA

template  ATGGAACGACAACCTCGTTTACTGCGACACAGGGGGCTGGAGGATGGAGCTTTACACCGC
query     ATGGAACGACAACCTCGTTTACTGCGACACAGGGGGCTGGAGGATGGAGCTTTACACCGC

template  CAGCGCCGTGGGGCGACGGTGATTATACGCTGACGGTGACAGTGGAGGATCGGGCGGGAA
query     CAGCGCCGTGGGGCGACGGTGATTATACGCTGACGGTGACAGTGGAGGATCGGGCGGGAA

template  ATACGCGTCCGTCTACGCCGCTGACGGTGACAGTGGATACGCAAATAGCCATTGATCGTA
query     ATACGCGTCCGTCTACGCCGCTGACGGTGACAGTGGATACGCAAATAGCCATTGATCGTA

template  TTGAATTAGTCAACGATAGCGGCGTCCCTGGCGATAATGTGACAAAACATGTGCGTCCGC
query     TTGAATTAGTCAACGATAGCGGCGTCCCTGGCGATAATGTGACAAAACATGTGCGTCCGC

template  AGTTCCAGATCTCGGTACCGGATGATGTGGAAAAGGTTCTTCTGAGTATTGACGGCGGCA
query     AGTTCCAGATCTCGGTACCGGATGATGTGGAAAAGGTTCTTCTGAGTATTGACGGCGGCA

template  CGACCTGGGTTACTGCAATCAAGAGTTCGACGGCTGGCATTTGGGATTACACCTGGCCGA
query     CGACCTGGGTTACTGCAATCAAGAGTTCGACGGCTGGCATTTGGGATTACACCTGGCCGA

template  CGGATATGCCAGAGGGACAGCATACCCTGACCGTGGAAGTGACTGACGGTGCGGGTAATA
query     CGGATATGCCAGAGGGACAGCATACCCTGACCGTGGAAGTGACTGACGGTGCGGGTAATA

template  AGATGACGGAGACGCTCAATTTCACTATCGATATCACGTTGTTAACGCCAACCATTGAGC
query     AGATGACGGAGACGCTCAATTTCACTATCGATATCACGTTGTTAACGCCAACCATTGAGC

template  TAGCGCCCGATCAGGATACCGGACAGAATAAGAACGATAATCTGACCAGCGTCACTCAGC
query     TAGCGCCCGATCAGGATACCGGACAGAATAAGAACGATAATCTGACCAGCGTCACTCAGC

template  CGGTATTTGTGTTGGGGAGTATCGATAAAGATGTTCGACACGTGGAATTGAGTATTGAGC
query     CGGTATTTGTGTTGGGGAGTATCGATAAAGATGTTCGACACGTGGAATTGAGTATTGAGC

template  ATAACGGCACGTTTAAAACGGTGGTACTCACCGAATCAGCCGACGGCTGGCGCTATCGAC
query     ATAACGGCACGTTTAAAACGGTGGTACTCACCGAATCAGCCGACGGCTGGCGCTATCGAC

template  CGGATTCTGCTTTGGCGGACGGTAGCTACACATTCACCGTGACGGTAACAGATGTGGCAG
query     CGGATTCTGCTTTGGCGGACGGTAGCTACACATTCACCGTGACGGTAACAGATGTGGCAG

template  GCAACCAGCAAACATCCGCGCCTTTAAAGGTGACGATAGACGGTACGTTGACTACGCCGG
query     GCAACCAGCAAACATCCGCGCCTTTAAAGGTGACGATAGACGGTACGTTGACTACGCCGG

template  TGATTGAACTGGCAGCTGGCGAAGATAGCGGTACTGTTGGCGATCGCCTCACCAATCACG
query     TGATTGAACTGGCAGCTGGCGAAGATAGCGGTACTGTTGGCGATCGCCTCACCAATCACG

template  ATCGGCCTGTGTTCGACATACATCAGGTTGATTCTGACGTTACGCGCGTGATGGTCAAAG
query     ATCGGCCTGTGTTCGACATACATCAGGTTGATTCTGACGTTACGCGCGTGATGGTCAAAG

template  TAACTTACAACGGTAAAACGCACGAGGAAGCGGCGGTATTCACCAATGGTCAATGGCGCT
query     TAACTTACAACGGTAAAACGCACGAGGAAGCGGCGGTATTCACCAATGGTCAATGGCGCT

template  TTACGCCTTCTGCGAGCTGGGCTGATGGCTCATATCAGTTAGCCGTTGTGGTGGAAGATC
query     TTACGCCTTCTGCGAGCTGGGCTGATGGCTCATATCAGTTAGCCGTTGTGGTGGAAGATC

template  TGGCGGGGAATGTAAAAGAGTCTGCGCCGTTTGAGGTGCGTATTGATACCACGACAACCA
query     TGGCGGGGAATGTAAAAGAGTCTGCGCCGTTTGAGGTGCGTATTGATACCACGACAACCA

template  TTAACAATATCGTATTGCTTAATGATACCGGCGTGCAGAATGATCAATTAACGAATGTTG
query     TTAACAATATCGTATTGCTTAATGATACCGGCGTGCAGAATGATCAATTAACGAATGTTG

template  CCAAACCGTCATTCAGAATTGACGTTCCCGGTGATGTCGTCCAGGTACGCGTAACCCTGG
query     CCAAACCGTCATTCAGAATTGACGTTCCCGGTGATGTCGTCCAGGTACGCGTAACCCTGG

template  ATGGTGGCGCTAACTGGAATGTGATACGCAAAAATGCCGACGGACAGTGGATTTTTGACA
query     ATGGTGGCGCTAACTGGAATGTGATACGCAAAAATGCCGACGGACAGTGGATTTTTGACA

template  GCCCGAATACTCTGGTTGACGGCACATATACCCTTCGCGTAGAGGCCACGGATGAGGCAG
query     GCCCGAATACTCTGGTTGACGGCACATATACCCTTCGCGTAGAGGCCACGGATGAGGCAG

template  GTAATATTGCGAATAAAGATTTAGTATTTAATATCGATACTAATATACAGGTTCCTACTA
```

```
query      GTAATATTGCGAATAAAGATTTAGTATTTAATATCGATACTAATATACAGGTTCCTACTA

template   TTGCTTTAGACGCAGGACAAGATACCGGAGCGAATACCGCCGATAATATTACTAATATTT
query      TTGCTTTAGACGCAGGACAAGATACCGGAGCGAATACCGCCGATAATATTACTAATATTT

template   CACGACCCACCTTTACGATTGGTAATGTTGACCCCGATGTTATCAAAGTCGTGGTGACGA
query      CACGACCCACCTTTACGATTGGTAATGTTGACCCCGATGTTATCAAAGTCGTGGTGACGA

template   TTGATGGTCATGATTATAACGCGACTAAGGTTGGGGCTGGTTGGCAATTTACACCAGGCA
query      TTGATGGTCATGATTATAACGCGACTAAGGTTGGGGCTGGTTGGCAATTTACACCAGGCA

template   ATGCCATTCCGGATGGTTCTTATAATATTACCGTTACGGTTGAAGATAAGGCCGGAAATA
query      ATGCCATTCCGGATGGTTCTTATAATATTACCGTTACGGTTGAAGATAAGGCCGGAAATA

template   CCGCGACATCGAAACCATTACCTGTTGTGATAGATACGACGGCTGAAATTGAAAGCGTCA
query      CCGCGACATCGAAACCATTACCTGTTGTGATAGATACGACGGCTGAAATTGAAAGCGTCA

template   CGTTGGTTACAGATAGCGGTGATAGCGATGTAGATAACATTACCAAAGTCGACAAGCCGC
query      CGTTGGTTACAGATAGCGGTGATAGCGATGTAGATAACATTACCAAAGTCGACAAGCCGC

template   AGTTTAGTATTGTTACCGCTGATGATATTACCCATGTGCGCGTTAAAATCGATAACGCCG
query      AGTTTAGTATTGTTACCGCTGATGATATTACCCATGTGCGCGTTAAAATCGATAACGCCG

template   CTAATTGGATTGAACTCACAAAAGGAGGGGATGGCCGCTGGATATTTAATGTCGGTTCGG
query      CTAATTGGATTGAACTCACAAAAGGAGGGGATGGCCGCTGGATATTTAATGTCGGTTCGG

template   CATTACCTGATGGGCAACACACTCTCTTGGTTGATGTGACTGATATCGCCGGCAACGTTG
query      CATTACCTGATGGGCAACACACTCTCTTGGTTGATGTGACTGATATCGCCGGCAACGTTG

template   CGCAAGAAACGCTGCAGTTTACGATTGATACGACTCTGCGAGAGCCGACAATTGTACTCG
query      CGCAAGAAACGCTGCAGTTTACGATTGATACGACTCTGCGAGAGCCGACAATTGTACTCG

template   ATCCCACCCATGATACTGGTGATGATACTAATGATAATCTTACCAGGATTAACAAACCGG
query      ATCCCACCCATGATACTGGTGATGATACTAATGATAATCTTACCAGGATTAACAAACCGG

template   TGTTTATTATCGGTAATGTCGATAATGATGTATCACACATTGTGGTTCATATTGATGGTC
query      TGTTTATTATCGGTAATGTCGATAATGATGTATCACACATTGTGGTTCATATTGATGGTC

template   GGGATTACACCATTGAAAACACAGGGGGGAATTTAACCTTTACGCCGGATCAACCGCTGT
query      GGGATTACACCATTGAAAACTCAGGGGGGAATTTAACCTTTACGCCGGATCAACCGCTGT

template   CTGACGGTCAGCATACGATCTCTGTTACCGTAACGGATATTGCTGGTAATACCAAAACAT
query      CTGACGGTCAGCATACGATCTCTGTTACCGTAACGGATATTGCTGGTAATACCAAAACAT

template   CGGCCGAACTGCGGATTGAAATCGACACGCAGGTTCAGATTGACAGTGTTACGTTAACAA
query      CGGCCGAACTGCGGATTGAAATCGACACGCAGGTTCAGATTGACAGTGTTACGTTAACAA

template   CAGATAGCGGCGTCAACGATCACGATAATGTCACCAATGCTACCCGTCCCTCTTTTGAAA
query      CAGATAGCGGCGTCAACGATCACGATAATGTCACCAATGCTACCCGTCCCTCTTTTGAAA

template   TTGCAACGCCTGATGATGTGACATCGGTGCTGGTTTCTTTCGATGGCGTAAACTGGACGC
query      TTGCAACGCCTGATGATGTGACATCGGTGCTGGTTTCTTTCGATGGCGTAAACTGGACGC

template   CCATCAGTAAAAATGCGGCCGGGCAGTGGGAATTTACTGCAGGTAGCGCATTGCCTGATG
query      CCATCAGTAAAAATGCGGCCGGGCAGTGGGAATTTACTGCAGGTAGCGCATTGCCTGATG

template   GTCATTATACTCTCCATGTCCAGGCGACGGATCGGGCAGGGAATACGGCAAATTCCACGC
query      GTCATTATACTCTCCATGTCCAGGCGACGGATCGGGCAGGGAATACGGCAAATTCCACGC

template   TGGGCTTCACCGTGGATACGCAGATTGACGGCCTGAGCGTCGTGATGCTGGACGACGCCG
query      TGGGCTTCACCGTGGATACGCAGATTGACGGCCTGAGCGTCGTGATGCTGGACGACGCCG

template   GAAAGGATTCTACGGATGGTATTACGAATATTACCTCTCCACGTTTTGAAATTTCAGCCA
query      GAAAGGATTCTACGGATGGTATTACGAATATTACCTCTCCACGTTTTGAAATTTCAGCCA

template   GAGAACCGCTGCAGAGCGTGACGGTAATTTTAAACGGGAAATCCAGCACACTGACTCAGG
query      GAGAACCGCTGCAGAGCGTGACGGTAATTTTAAACGGGAAATCCAGCACACTGACTCAGG

template   GGGCAGGTAATAAATGGCTGTTTACCCCTGATACACCGTTAGTGGATGGAACTTACAAAA
query      GGGCAGGTAATAAATGGCTGTTTACCCCTGATACACCGTTAGTGGATGGAACTTACAAAA
```

```
template    TAGAAATAGTGGCTGAAGATATCGCAGGTAATAAAATTAGCAAAGAGGTATCATTCACAA
query       TAGAAATAGTGGCTGAAGATATCGCAGGTAATAAAATTAGCAAAGAGGTATCATTCACAA

template    TAGACACTATTGTTTCTGATCCCAGTATTGATTTGCTGGATGCGGATGATACTGGCGAAA
query       TAGACACTATTGTTTCTGATCCCAGTATTGATTTGCTGGATGCGGATGATACTGGCGAAA

template    GCGCTGTTGATAATATTACGAGTGTCACTACACCACGTTTCGTTATTGGCAATGTACCCG
query       GCGCTGTTGATAATATTACGAGTGTCACTACACCACGTTTCGTTATTGGCAATGTACCCG

template    CCGATATTGATACTGTTGTAATCAGAATTAACGGCGTTTCTTATCCGGTTACGGCAAATG
query       CCGATATTGATACTGTTGTAATCAGAATTAACGGCGTTTCTTATCCGGTTACGGCAAATG

template    GCAATAACCTCTGGGAATTTCAGGTTCCCGTTGCGTTAAACGATGGCGTATATGAAGCCG
query       GCAATAACCTCTGGGAATTTCAGGTTCCCGTTGCGTTAAACGATGGCGTATATGAAGCCG

template    TTGTTGTCTTCAGAGATATTGCCGGAAATACTTCTGAAACTAAGCTGCCCTTTACCATTG
query       TTGTTGTCTTCAGAGATATTGCCGGAAATACTTCTGAAACTAAGCTGCCCTTTACCATTG

template    ATACCACGACAAGCGTCAGTGTCAGAATGGAGCCAGCGTCTGATACCGGAAATTCCAATA
query       ATACCACGACAAGCGTCAGTGTCAGAATGGAGCCAGCGTCTGATACCGGAAATTCCAATA

template    GCGATAACCTTACGAATAAGCAAAATCCCAAATTCGAAGGTACTGCAGAGCCCAATGCGA
query       GCGATAACCTTACGAATAAGCAAAATCCCAAATTCGAAGGTACTGCAGAGCCCAATGCGA

template    AACTGGTGATTACCATTGTTGACGATAAGTCAGGTCGGGAGGTTTTAAAACAAACGATTA
query       AACTGGTGATTACCATTGTTGACGATAAGTCAGGTCGGGAGGTTTTAAAACAAACGATTA

template    CGGTTGGCGCTGATGGCAACTGGAGTGTGACGCCGAATATACTGCCGGATGGCATGTATA
query       CGGTTGGCGCTGATGGCAACTGGAGTGTGACGCCGAATATACTGCCGGATGGCATGTATA

template    CCATCAACGTCGTCGCAACAGATGTCGCGGGAAATACTGCGCAAACGCAGGAAAGATTCA
query       CCATCAACGTCGTCGCAACAGATGTCGCGGGAAATACTGCGCAAACGCAGGAAAGATTCA

template    CTATCGATACGGTTACGATCGATCCCACCATTCGCCTTTCGGATCCATCTATTGATGATC
query       CTATCGATACGGTTACGATCGATCCCACCATTCGCCTTTCGGATCCATCTATTGATGATC

template    AGCATGAAGCAACCAGCCTGCGTCCTGAGTTCAAAGGGTTTGCCGAAGCGTTCTCGACGA
query       AGCATGAAGCAACCAGCCTGCGTCCTGAGTTCAAAGGGTTTGCCGAAGCGTTCTCGACGA

template    TTATGATTCAGTGGGATGGGAAAGTGGTCGGCTCGGCAAACGCCAATGCGAATGGCGAAT
query       TTATGATTCAGTGGGATGGGAAAGTGGTCGGCTCGGCAAACGCCAATGCGAATGGCGAAT

template    GGAGTTGGACGCCGCCATCAGTATTAGCGCCAGGCTCCTATGTTGTGAGCATTGTTGCCA
query       GGAGTTGGACGCCGCCATCAGTATTAGCGCCAGGCTCCTATGTTGTGAGCATTGTTGCCA

template    AAGATAAAGCGGGTAATGAATCGTCGCAGGTCGACTTTCCTGTCGTAATACCTGTTATTG
query       AAGATAAAGCGGGTAATGAATCGTCGCAGGTCGACTTTCCTGTCGTAATACCTGTTATTG

template    ATGTCACGCCTCCAACCATAAAGCTCAGCGAGGAGAGCGATAGTGGCGCCTTAGGAGACT
query       ATGTCACGCCTCCAACCATAAAGCTCAGCGAGGAGAGCGATAGTGGCGCCTTAGGAGACT

template    TTACCACGAATAATAAAACGCCGACCCTGATTGGGAGCACGTTACCTAATACGATTGTGA
query       TTACCACGAATAATAAAACGCCGACCCTGATTGGGAGCACGTTACCTAATACGATTGTGA

template    GTATTTATGTGGATGGCGTGAAGGTCGGCGAGGCGACAGCGGATACAGCGGGTCGATATA
query       GTATTTATGTGGATGGCGTGAAGGTCGGCGAGGCGACAGCGGATACAGCGGGTCGATATA

template    CTTTCCAGTTATCGGAAATGAAAGATGGCCATTATGTCGTCCAGGTGGGTATCGTCAACC
query       CTTTCCAGTTATCGGAAATGAAAGATGGCCATTATGTCGTCCAGGTGGGTATCGTCAACC

template    CTCGCGATAATAGCGAACTGCGTTCTACCGCCGTTGATGTCACTATCGATACCGAGGTTG
query       CTCGCGATAATAGCGAACTGCGTTCTACCGCCGTTGATGTCACTATCGATACCGAGGTTG

template    CTGAACTGGTATGGAATATATCTGGAATGCATGAGGGCGGATATATCAATACGGTGACGC
query       CTGAACTGGTATGGAATATATCTGGAATGCATGAGGGCGGATATATCAATACGGTGACGC

template    CGGAGATTGGCGGCACCAGTGAGCCAAACAGCAAAATCACTATCTTTGTGAATGGCGTTG
query       CGGAGATTGGCGGCACCAGTGAGCCAAACAGCAAAATCACTATCTTTGTGAATGGCGTTG
```

```
template    AAAAAGCGATTGCTTATACGACAGGCGCAGGACACTGGGGCGTAGTATTACCCGCTTTGG
query       AAAAAGCGATTGCTTATACGACAGGCGCAGGACACTGGGGCGTAGTATTACCCGCTTTGG

template    GTAATGACGGTAATTATGAATTAACGTTTAAAGTTGAAGACGTTGCCGGTAATATCAGAG
query       GTAATGACGGTAATTATGAATTAACGTTTAAAGTTGAAGACGTTGCCGGTAATATCAGAG

template    AGTTTGGTCCGCAGAATGTAATACTGGATACAGTAATTTCGCCGTTAACCGTGGTATTAC
query       AGTTTGGTCCGCAGAATGTAATACTGGATACAGTAATTTCGCCGTTAACCGTGGTATTAC

template    GCGAAGCTGATGACAGTGGCAAAGTTGGCGACTGGATCACCAATAAATCTCATGTCACCA
query       GCGAAGCTGATGACAGTGGCAAAGTTGGCGACTGGATCACCAATAAATCTCATGTCACCA

template    TCGATGGTACTGCCGAAGCCGGAAGTACTTTAACCATCAGGAATCCGCAGGGAGTGGTTA
query       TCGATGGTACTGCCGAAGCCGGAAGTACTTTAACCATCAGGAATCCGCAGGGAGTGGTTA

template    TTGCTACCCTGGTGGTAGGCAATGATGGTCGATGGAGCGCAGAATTAGATCTGCGTGAAG
query       TTGCTACCCTGGTGGTAGGCAATGATGGTCGATGGAGCGCAGAATTAGATCTGCGTGAAG

template    GTAGTAATGCCTTTGTCGTGGTATCGGAAGATAAAGCGGGCAACAGTCAACAAAAAGAGA
query       GTAGTAATGCCTTTGTCGTGGTATCGGAAGATAAAGCGGGCAACAGTCAACAAAAAGAGA

template    TTCTGATAGAACATGATACGCAGATTGAAATCAGCGATATTTCATTAAGTCGGGATACTA
query       TTCTGATAGAACATGATACGCAGATTGAAATCAGCGATATTTCATTAAGTCGGGATACTA

template    ATAGCGGTGATAAATATGATCTGATTACCAATAATAAGTCTCCGGTACTGGTTGCCATGA
query       ATAGCGGTGATAAATATGATCTGATTACCAATAATAAGTCTCCGGTACTGGTTGCCATGA

template    CCGATCCCGGCGCGACGGTACAGGTTTATATTAATGGTGTGTTACAAGGCACAGTAGAGG
query       CCGATCCCGGCGCGACGGTACAGGTTTATATTAATGGTGTGTTACAAGGCACAGTAGAGG

template    CGAGTTCGTCAGGTAATATTAGCTATACCATGCCGGCAAATAGCGCCGACGGCGAGTATC
query       CGAGTTCGTCAGGTAATATTAGCTATACCATGCCGGCAAATAGCGCCGACGGCGAGTATC

template    AGGTGCAATTTGTTGCTACGGATACTGCTGGTAACCGGGTTGAGTCTGCGATTACAACCG
query       AGGTGCAATTTGTTGCTACGGATACTGCTGGTAACCGGGTTGAGTCTGCGATTACAACCG

template    TGACAATCGATTCTCAAATTGCTGTCTTTGATATTGATGAAGATTCATTACCGGCCCTCT
query       TGACAATCGATTCTCAAATTGCTGTCTTTGATATTGATGAAGATTCATTACCGGCCCTCT

template    CTAATAACCGAGCGTTGTCAGTCTCAGGTGTCGGGGAGGCTGGTTCTCAGGTCAGCATCT
query       CTAATAACCGAGCGTTGTCAGTCTCAGGTGTCGGGGAGGCTGGTTCTCAGGTCAGCATCT

template    TTGTCGATGGTAAATTAGTCAACGTTGTTATGGTTGAGGCTGATGGCACATGGCGCGCGC
query       TTGTCGATGGTAAATTAGTCAACGTTGTTATGGTTGAGGCTGATGGCACATGGCGCGCGC

template    CGATACTGCTGCAAGATGATGGTACGTTTAATATTCATTTCAGCATTACTGACGTTGCTG
query       CGATACTGCTGCAAGATGATGGTACGTTTAATATTCATTTCAGCATTACTGACGTTGCTG

template    GCAACACTGAAGTGAGCAAGGATTATAGCGTGGATGTCGATTCATCAACCGACTTCCCAA
query       GCAACACTGAAGTGAGCAAGGATTATAGCGTGGATGTCGATTCATCAACCGACTTCCCAA

template    CGCTCAACCTTGAAGATGCAAGCAACTCTGGTTCACTTGACGATCTGATTACTAATCACA
query       CGCTCAACCTTGAAGATGCAAGCAACTCTGGTTCACTTGACGATCTGATTACTAATCACA

template    ACAAGCCTGTATTAGTTGGCACCGCAGAAGCGGGAGCCACAATCCATATTTATGTGGATG
query       ACAAGCCTGTATTAGTTGGCACCGCAGAAGCGGGAGCCACAATCCATATTTATGTGGATG

template    AAAAGATCGTGGCAAATGTTCTTGTGCTTGAAGATGGAACCTGGTCCTATCAGTTTGATA
query       AAAAGATCGTGGCAAATGTTCTTGTGCTTGAAGATGGAACCTGGTCCTATCAGTTTGATA

template    ATGCGTTAAAAGATGGTGAATATTCTATCCGTGTGGTTGCCGAAGACCCGGCAGGTAATA
query       ATGCGTTAAAAGATGGTGAATATTCTATCCGTGTGGTTGCCGAAGACCCGGCAGGTAATA

template    CGGCAGAATCGCCTCGCTTACTCGTCACGATAGATACCAGTACGTTTATCGATAATCCTG
query       CGGCAGAATCGCCTCGCTTACTCGTCACGATAGATACCAGTACGTTTATCGATAATCCTG

template    CTATGGTGGCAGGTTCTGATAATGGTATTTTCAGTAATGATAGTATAACGAGTCAGACCC
query       CTATGGTGGCAGGTTCTGATAATGGTATTTTCAGTAATGATAGTATAACGAGTCAGACCC

template    GGCCTACGTTTAGTATTTTTGGAGAAATGAACCAGAGTGTTCAGATTTTCATTGATGGAG
```

```
query     GGCCTACGTTTAGTATTTTTGGAGAAATGAACCAGAGTGTTCAGATTTTCATTGATGGAG

template  TGCTAGTCGATACGATCACGGTGACCGACAGAAATCAAGTTTATCGACCTGAGTCACCGT
query     TGCTAGTCGATACGATCACGGTGACCGACAGAAATCAAGTTTATCGACCTGAGTCACCGT

template  TGGGCGATGGTTCCCATAGCATTTATTATGTTATCACCGATAAAGCAGGCAACACGGCTA
query     TGGGCGATGGTTCCCATAGCATTTATTATGTTATCACCGATAAAGCAGGCAACACGGCTA

template  CCTCGAAAACGCTAAACTTTACTATCGATACCTTTAATACGACGCCTGTCGCCATTGATT
query     CCTCGAAAACGCTAAACTTTACTATCGATACCTTTAATACGACGCCTGTCGCCATTGATT

template  CTATCGGTGGACAAACGTTAGCAGAGATGACCGGTAGTGATGGCAAAATATATATAACGG
query     CTATCGGTGGACAAACGTTAGCAGAGATGACCGGTAGTGATGGCAAAATATATATAACGG

template  ACACGACGCGTAACTTATTGTTTAGTGGCAGTGCCGAGCCCAATAGCAAAATAGAAATCA
query     ACACGACGCGTAACTTATTGTTTAGTGGCAGTGCCGAGCCCAATAGCAAAATAGAAATCA

template  TCATTAATGGCTTAAATGTGGGGGAAGTTTGGGTTAATGAAAAAGGCCACTGGCAGATGC
query     TCATTAATGGCTTAAATGTGGGGGAAGTTTGGGTTAATGAAAAAGGCCACTGGCAGATGC

template  CGGTGAACCCGCTTTATTTCACAGAAGGCCAACTGGATATCACTGTTAAATCTACGGACC
query     CGGTGAACCCGCTTTATTTCACAGAAGGCCAACTGGATATCACTGTTAAATCTACGGACC

template  GTGCTGGTAACGTAAATCAGGAAAAGTATTCCATTTGGGTTGATACGCATATCAAGGTAT
query     GTGCTGGTAACGTAAATCAGGAAAAGTATTCCATTTGGGTTGATACGCATATCAAGGTAT

template  TTACCAGCGAGCTTGATGACAATAAATCATCATCGAAAACGGAATGGTGGAGTAATAGCG
query     TTACCAGCGAGCTTGATGACAATAAATCATCATCGAAAACGGAATGGTGGAGTAATAGCG

template  ATCTCATTACCATGCGAGGCACGGGTGAAATTGGCGCTACGGTATCATTAATCGTGGCTG
query     ATCTCATTACCATGCGAGGCACGGGTGAAATTGGCGCTACGGTATCATTAATCGTGGCTG

template  GCGTCACGCTGGCAACTGCTGTTGTGGCGGCAACAGGACGATGGGAATTATCAACAGACA
query     GCGTCACGCTGGCAACTGCTGTTGTGGCGGCAACAGGACGATGGGAATTATCAACAGACA

template  AGCTTCCAGAAGGGACTTACGATATTAGTTTGGTCATTGAAGATAGCGCCGGAAATCGTT
query     AGCTTCCAGAAGGGACTTACGATATTAGTTTGGTCATTGAAGATAGCGCCGGAAATCGTT

template  GGGAAGATGTGCGTGAAATATTTATTGACCGAACGCCGCCAAATGCTCCGGTCGTAACGT
query     GGGAAGATGTGCGTGAAATATTTATTGACCGAACGCCGCCAAATGCTCCGGTCGTAACGT

template  ATTCAGATATTGTCAACGATCTAATTATTATGCAGGGGACGGCGGAAGCCAAATCTCAGC
query     ATTCAGATATTGTCAACGATCTAATTATTATGCAGGGGACGGCGGAAGCCAAATCTCAGC

template  TAATAATAACCGATAGTGAGGGGAATACTTATACGTTAACCGTTCCTGATAATGGTAAAT
query     TAATAATAACCGATAGTGAGGGGAATACTTATACGTTAACCGTTCCTGATAATGGTAAAT

template  GGAGTATGGCTATCCCGTATCCATCAGAAGGGAAGTTTACCATTACGAGTGTGGATGCTA
query     GGAGTATGGCTATCCCGTATCCATCAGAAGGGAAGTTTACCATTACGAGTGTGGATGCTA

template  TTGGTAACCGGAGTGATGATGTCCCTCTCGATATCATGAAAGAGGTTCCCGTTATTTCAT
query     TTGGTAACCGGAGTGATGATGTCCCTCTCGATATCATGAAAGAGGTTCCCGTTATTTCAT

template  TATCTCCAGACTCAGACAGTGGTACGGTGGGCGATAATATTACGCGAGATAAGCAACCTA
query     TATCTCCAGACTCAGACAGTGGTACGGTGGGCGATAATATTACGCGAGATAAGCAACCTA

template  CCTTTATTATCGGGAATCTGGAAAGCGATGTTGTGGTCGTTCAGGTCGATATCAATGGGA
query     CCTTTATTATCGGGAATCTGGAAAGCGATGTTGTGGTCGTTCAGGTCGATATCAATGGGA

template  CCGTATATAATGCTGAAAAAAATGCCGATGGCGTTTGGTTCTTTACGCCAGGTACACCGT
query     CCGTATATAATGCTGAAAAAAATGCCGATGGCGTTTGGTTCTTTACGCCAGGTACACCGT

template  TAGCTGATGGTTCCTATACGATATCGGTAATCGCAAGCGATGCCGCGGGTAATCAGAAAA
query     TAGCTGATGGTTCCTATACGATATCGGTAATCGCAAGCGATGCCGCGGGTAATCAGAAAA

template  ACTCGTTACCCATTACTGTCACGATCGACAGCACGCTGACGGTGCCGGAGATTGCGTTGG
query     ACTCGTTACCCATTACTGTCACGATCGACAGCACGCTGACGGTGCCGGAGATTGCGTTGG

template  CAGCAGGTGAAGACAATGGCGCTTCAGACAGCGATAACGTGACGAATCACACCCAGCCTA
query     CAGCAGGTGAAGACAATGGCGCTTCAGACAGCGATAACGTGACGAATCACACCCAGCCTA
```

```
template   AGTTCACGCTGCAGCATATTGATGCTGATGTGACCGGGGTGACCGTAAACGTGACGCATA
query      AGTTCACGCTGCAGCATATTGATGCTGATGTGACCGGGGTGACCGTAAACGTGACGCATA

template   ATGGCGTGACAGACATCTATCAGGCGACGCAAGGCGCGGATGGCTGGACCTTCACGCCGC
query      ATGGCGTGACAGACATCTATCAGGCGACGCAAGGCGCGGATGGCTGGACCTTCACGCCGC

template   CAGCCGCCTGGAATGACGGTAACTACACGCTGAGCGTGACGGTGGTGGATCGCGCGGGGA
query      CAGCCGCCTGGAATGACGGTAACTACACGCTGAGCGTGACGGTGGTGGATCGCGCGGGGA

template   ATTCACAGCAATCTGCTTCGCTAGCGGTGACGGTTGACTCAACGGTGACGGTAACAGCGG
query      ATTCACAGCAATCTGCTTCGCTAGCGGTGACGGTTGACTCAACGGTGACGGTAACAGCGG

template   ATAGCCAGCATGACGATGCGAGCGATGACGCCACGGCAACAGCGGTTACTCCACCGGAGT
query      ATAGCCAGCATGACGATGCGAGCGATGACGCCACGGCAACAGCGGTTACTCCACCGGAGT

template   CTGAAACAGTGAATGCCGAAAGCGCTACGCATCTTCGTACAGAGCCGTCTGCGGCGGAAG
query      CTGAAACAGTGAATGCCGAAAGCGCTACGCATCTTCGTACAGAGCCGTCTGCGGCGGAAG

template   AAAGCGTGGTGAAGGTGACAGCCTATAGTATTACATTGTTAAACGCTGACTCTGGGGATG
query      AAAGCGTGGTGAAGGTGACAGCCTATAGTATTACATTGTTAAACGCTGACTCTGGGGATG

template   AAATAGATCGTTCAATTAGTCAGACACCTTCTTTTGAAATATCAGTACCTGAGAATATTG
query      AAATAGATCGTTCAATTAGTCAGACACCTTCTTTTGAAATATCAGTACCTGAGAATATTG

template   TTAATGTCAGTATTATGTTTGAAGGAGAAGAGTTTACTCTGCCGATAACTAACCAGAAAG
query      TTAATGTCAGTATTATGTTTGAAGGAGAAGAGTTTACTCTGCCGATAACTAACCAGAAAG

template   CAATATTCGAAGTTCCGCTATCTTTGGAAGATGGTGAATATACTATGGACGTGAAATTCA
query      CAATATTCGAAGTTCCGCTATCTTTGGAAGATGGTGAATATACTATGGACGTGAAATTCA

template   TTGATAAAGACAATGATTTCCTGATTAAGGAGAAAACATTCTCAGTCGATCACTCCTCGG
query      TTGATAAAGACAATGATTTCCTGATTAAGGAGAAAACATTCTCAGTCGATCACTCCTCGG

template   CGGATATTGTGAACGCAATGAATGTAAGAGGAAAGACCGAGGATGATATTAATGATTCCC
query      CGGATATTGTGAACGCAATGAATGTAAGAGGAAAGACCGAGGATGATATTAATGATTCCC

template   CTTCCACGAGTTCTGTAGGGCACAACAATAACGGCGCTATTGATGTTTTCGCCGTTAATG
query      CTTCCACGAGTTCTGTAGGGCACAACAATAACGGCGCTATTGATGTTTTCGCCGTTAATG

template   AAGTTACGCTACCTGTAGATAATCAAGAAGAACACGCATAATAACGGAGGCCCCTCACCT
query      AAGTTACGCTACCTGTAGATAATCAAGAAGAACACGCATAATAACGGAGGCCCCTCACCT

template   TTGGGTGAGGGGGTTTACTTATGGATAAAAAACTAGAACCTTATTATTTAAGTGCGGAAA
query      TTGGGTGAGGGGGTTTACTTATGGATAAAAAACTAGAACCTTATTATTTAAGTGCGGAAA

template   CGGCATTATCTATAGTGTCTAAAAAATTCAACATAAAAATTGACATCAAAGAGGATGATA
query      CGGCATTATCTATAGTGTCTAAAAAATTCAACATAAAAATTGACATCAAAGAGGATGATA

template   TAAATTTAAGATTTAAAAAGTACGACAGAAATAACACTGACGACTCTATACAAATGAAGA
query      TAAATTTAAGATTTAAAAAGTACGACAGAAATAACACTGACGACTCTATACAAATGAAGA

template   ATTTCTTTTTGTCGTTAGGGCTTTCTCTACAGGATATATTATTTAATAATGGTGAGGATT
query      ATTTCTTTTTGTCGTTAGGGCTTTCTCTACAGGATATATTATTTAATAATGGTGAGGATT

template   TACTAAATGAGCCTATGCCGATTTTACTATTAACACCAGAAATGAAGTGGATGGTGTGTG
query      TACTAAATGAGCCTATGCCGATTTTACTATTAACACCAGAAATGAAGTGGATGGTGTGTG

template   TGAGTGGCGGGCAAAAAATAAAGTTGGTAAACGCGCGCGGTGAACTCTGTTATGTTGAAA
query      TGAGTGGCGGGCAAAAAATAAAGTTGGTAAACGCGCGCGGTGAACTCTGTTATGTTGAAA

template   TTGAAGATGAATATTTAAAAGAGTTATCTGCATTTAGTATACTACCTTTAAATAAAGTTG
query      TTGAAGATGAATATTTAAAAGAGTTATCTGCATTTAGTATACTACCTTTAAATAAAGTTG

template   TTGATAGTATAAGAGTAAAAAATATCATAAAAAACTCTTTATCGATGAACAAGATTTTTT
query      TTGATAGTATAAGAGTAAAAAATATCATAAAAAACTCTTTATCGATGAACAAGATTTTTT

template   ATACTAAATACTTTTTTTCATCTCTTTTTATGGCAATTTTTGCGTTAACTATCCCAGTAT
query      ATACTAAATACTTTTTTTCATCTCTTTTTATGGCAATTTTTGCGTTAACTATCCCAGTAT
```

```
template   TTAGTAATCTGTTCTATGATAAGCTTGTTCCAAGCGCTTCGGTTTCATCTTTATTTGGCG
query      TTAGTAATCTGTTCTATGATAAGCTTGTTCCAAGCGCTTCGGTTTCATCTTTATTTGGCG

template   TGGCTATAATTGTTGCTGTATTTATTGTTTTTGAGTTTATCCTTCGTACTTCGAAAGATA
query      TGGCTATAATTGTTGCTGTATTTATTGTTTTTGAGTTTATCCTTCGTACTTCGAAAGATA

template   TTTATCAGTCTATCACAGCAAGGCAGGATGACGTCGATATTGATATCGCATTTCTTGAAG
query      TTTATCAGTCTATCACAGCAAGGCAGGATGACGTCGATATTGATATCGCATTTCTTGAAG

template   CGGTACTTTATAGTAAAAAGAAAAATGGCAGATCCATGTCATCAGCATTTGTGCTATGGA
query      CGGTACTTTATAGTAAAAAGAAAAATGGCAGATCCATGTCATCAGCATTTGTGCTATGGA

template   ATGAGTTTCAGAAAATTAAACCCGTTTTATTAAACTCGATCTTTCAACGTATAGCCGATA
query      ATGAGTTTCAGAAAATTAAACCCGTTTTATTAAACTCGATCTTTCAACGTATAGCCGATA

template   TTCCAATATTTATTATATTTCTCATTGTTATATATGTAAATTTAGGTCTGGTTGTTATTG
query      TTCCAATATTTATTATATTTCTCATTGTTATATATGTAAATTTAGGTCTGGTTGTTATTG

template   TACCTATTACCATGTTTATCGTCTCTATTATTATTTCCCTCGTTAACCACCATTATACTA
query      TACCTATTACCATGTTTATCGTCTCTATTATTATTTCCCTCGTTAACCACCATTATACTA

template   ATGAGTTAATGAACAAACAAAAAGAAGGACAGAAGAACAGGAATATTTTTATCTCAGAAG
query      ATGAGTTAATGAACAAACAAAAAGAAGGACAGAAGAACAGGAATATTTTTATCTCAGAAG

template   TTTTCTTATCTATTAAAATGATCCATACCTTAAATAATCAAGGTTTACTTTTTGATTGGG
query      TTTTCTTATCTATTAAAATGATCCATACCTTAAATAATCAAGGTTTACTTTTTGATTGGG

template   TTAATACATCAAATGAACAGTCGTATCTTAACCTGAAGATAAGGAAATTAAATCTTATCT
query      TTAATACATCAAATGAACAGTCGTATCTTAACCTGAAGATAAGGAAATTAAATCTTATCT

template   ATCAATCTATATTGGGGAGTATGTCATCTATTACCCAAATAACTATTATGGTAATAGCCT
query      ATCAATCTATATTGGGGAGTATGTCATCTATTACCCAAATAACTATTATGGTAATAGCCT

template   TTTTTATGGTAATCAAGGGTGATGTTACTACTGGCGCAATTGTTTCATCTGTCATTGTCT
query      TTTTTATGGTAATCAAGGGTGATGTTACTACTGGCGCAATTGTTTCATCTGTCATTGTCT

template   CTGGCCGTATTTCCGGGATCATTTCGAATTTTTCTTCTACATTAATCTCTATTTTATCAG
query      CTGGCCGTATTTCCGGGATCATTTCGAATTTTTCTTCTACATTAATCTCTATTTTATCAG

template   CAGAAAAAACCGGTAAGGATCTGCTTTCTTTTTTTGATGAAGATCAGGCAGAAAAAACAC
query      CAGAAAAAACCGGTAAGGATCTGCTTTCTTTTTTTGATGAAGATCAGGCAGAAAAAACAC

template   CGGCATTACAGTCAATATCAAAGTGCAATGGCGATATCTCTATCCGGGGCGTGAGTTATC
query      CGGCATTACAGTCAATATCAAAGTGCAATGGCGATATCTCTATCCGGGGCGTGAGTTATC

template   AGTATGATGCTCAATCTCCGATGATTATTAACCGACTGTCTATAGACATACCTGCGGGGC
query      AGTATGATGCTCAATCTCCGATGATTATTAACCGACTGTCTATAGACATACCTGCGGGGC

template   AACGTGTCGCGGTGGTAGGCGAATGCGGAGCAGGAAAAAGCTCATTACTGGGAATGCTAT
query      AACGTGTCGCGGTGGTAGGCGAATGCGGAGCAGGAAAAAGCTCATTACTGGGAATGCTAT

template   CTGGCTACCTTTCGCCAACAGACGGTGCCATTTTATATGATGGATATAACTTAGGACATT
query      CTGGCTACCTTTCGCCAACAGACGGTGCCATTTTATATGATGGATATAACTTAGGACATT

template   TATCGCAGAACTTTTTTTCTCAGCATTTAAGCGTGGTGACGACACATGATGTTTTATTCA
query      TATCGCAGAACTTTTTTTCTCAGCATTTAAGCGTGGTGACGACACATGATGTTTTATTCA

template   CCGGAACCATTGAAAGTAATTTCGCGTTAAAACCGCAAAACGACAGGGGCCGGGTACTCA
query      CCGGAACCATTGAAAGTAATTTCGCGTTAAAACCGCAAAACGACAGGGGCCGGGTACTCA

template   AGGCGCTTCAGCTGGCGAACTGTGGTTTTATCTTGCAACATCCTATGGGGCTGAAGTTTC
query      AGGCGCTTCAGCTGGCGAACTGTGGTTTTATCTTGCAACATCCTATGGGGCTGAAGTTTC

template   CGGTGAATTTTATGGCTAAAAACCTGTCATCCGGACAGCAGCAGCAGTTATTATTAGCAC
query      CGGTGAATTTTATGGCTAAAAACCTGTCATCCGGACAGCAGCAGCAGTTATTATTAGCAC

template   GTAGTCTGAGTAGTGACGCCAGCGTCTTTTTATGGGATGAACCAACATCAAATCTGGATG
query      GTAGTCTGAGTAGTGACGCCAGCGTCTTTTTATGGGATGAACCAACATCAAATCTGGATG

template   AGAATACCGAGAAGCAAATTTTTGATAACTTAGATGAGTTTATTCATGGGAAAACGTTGA
```

```
query      AGAATACCGAGAAGCAAATTTTTGATAACTTAGATGAGTTTATTCATGGGAAAACGTTGA

template   TCATGGTGACGCATCGTCGATATCTGATAAAGTATTTTGACCGGGTCCTGGTAATGAAAG
query      TCATGGTGACGCATCGTCGATATCTGATAAAGTATTTTGACCGGGTCCTGGTAATGAAAG

template   GTGGAAAAATAATCCGTGATTGTTCTCCGGATAAATTATTAATGTAAAATAAGCAGCGCT
query      GTGGAAAAATAATCCGTGATTGTTCTCCGGATAAATTATTAATGTAAAATAAGCAGCGCT

template   TGTCGCTGTTATCAGGTGGTACTGCTTAATAAAAAAGACCCGTTGCACAAACGGGTCTTT
query      TGTCGCTGTTATCAGGTGGTACTGCTTAATAAAAAAGACCCGTTGCACAAACGGGTCTTT

template   TTTGTCATTTAACGAGTCGGCAACGTCTTCAATAAGTTTAGCTCGATTCTGTTAGGGCTA
query      TTTGTCATTTAACGAGTCGGCAACGTCTTCAATAAGTTTAGCTCGATTCTGTTAGGGCTA

template   TTCCACTTGCCATTTTTGGATAACCACACCTGGCGGCCCTCATCAACGGCAATGCGAGGG
query      TTCCACTTGCCATTTTTGGATAACCACACCTGGCGGCCCTCATCAACGGCAATGCGAGGG

template   ACGTGATGGTGCGCAAGGCTACCCCTGGCGCGCGATTCCGCGTTGAGATAACCGGTGGGC
query      ACGTGATGGTGCGCAAGGCTACCCCTGGCGCGCGATTCCGCGTTGAGATAACCGGTGGGC

template   GGCTTCAGCGGCAGCGATAGCGTATCAGGTCAGCACCATCGGCCATTCTGGCGGGGTCTG
query      GGCTTCAGCGGCAGCGATAGCGTATCAGGTCAGCACCATCGGCCATTCTGGCGGGGTCTG
```

SPI-5_NC_003197

```
template   CGGAAGTGTGGCCGAGCGGTTGAAGGCACCGGTCTTGAAAACCGGCGACCCGAAAGGGTT
query      CGGAAGTGTGGCCGAGCGGTTGAAGGCACCGGTCTTGAAAACCGGCGACCCGAAAGGGTT

template   CCAGAGTTCGAATCTCTGCGCTTCCGCCAGATTAAACAAGGGGTTACCGAAAGGTAGCCC
query      CCAGAGTTCGAATCTCTGCGCTTCCGCCAGATTAAACAAGGGGTTACCGAAAGGTAGCCC

template   CTTTGTTTTTTTAGGCGCTATAGAATATAGTTAGAAAATACTGTTAGAATATTTCCCGAA
query      CTTTGTTTTTTTAGGCGCTATAGAATATAGTTAGAAAATACTGTTAGAATATTTCCCGAA

template   AAAACGGACTACGCGAGTCTTTAGTTTCTTTTCGTTTCCCGATGTGTCTATTTATTGAAG
query      AAAACGGACTACGCGAGTCTTTAGTTTCTTTTCGTTTCCCGATGTGTCTATTTATTGAAG

template   ATGTAGACCATTCTGGGAGGTGAAGGATGCCCCATCTCTTTCAGAATAATGTTGGTATAT
query      ATGTAGACCATTCTGGGAGGTGAAGGATGCCCCATCTCTTTCAGAATAATGTTGGTATAT

template   TCGACAACAGGGCCTCTTGGATGATTTTCTTCTTTATCCTGAAGATGGGTCAGGGCATGT
query      TCGACAACAGGGCCTCTTGGATGATTTTCTTCTTTATCCTGAAGATGGGTCAGGGCATGT

template   ACAACTTCATGAATAAATGAACGTTTTGTGTCAAAAAGTTGTCTTCCTTCGTTACTTTCA
query      ACAACTTCATGAATAAATGAACGTTTTGTGTCAAAAAGTTGTCTTCCTTCGTTACTTTCA

template   TAATGTTCGGTATATGAATCATCAGAATCGTCCAGATTGAGACAAATAACTTTCCTGCCT
query      TAATGTTCGGTATATGAATCATCAGAATCGTCCAGATTGAGACAAATAACTTTCCTGCCT

template   TCTGAAAGTTTGAAGTGTTCCTGAGCCACGGTAGTTTCAAAGGCTTCGCCTGCCCCCAGT
query      TCTGAAAGTTTGAAGTGTTCCTGAGCCACGGTAGTTTCAAAGGCTTCGCCTGCCCCCAGT

template   AGCCAGCGCTGCTCCACATCATGTAGTTCTTTTTCATATGCGTAATTCATCAGTCTGCGG
query      AGCCAGCGCTGCTCCACATCATGTAGTTCTTTTTCATATGCGTAATTCATCAGTCTGCGG

template   AATGTTTCGCTTTGGGTATACGCATTTTGAAGTACGGAGGATAGTTCATCGTAGCATTCG
query      AATGTTTCGCTTTGGGTATACGCATTTTGAAGTACGGAGGATAGTTCATCGTAGCATTCG

template   TCATAAGTGTCGTCATCAATTTCTGTATCAGGGTCTATTCCACCCGCGCCTGAGATAAGG
query      TCATAAGTGTCGTCATCAATTTCTGTATCAGGGTCTATTCCACCCGCGCCTGAGATAAGG

template   TACTCCACCACACACTCTGGCTCCAGACGGAATTCACTGTTTATGGCAAGGCTGTCATGA
query      TACTCCACCACACACTCTGGCTCCAGACGGAATTCACTGTTTATGGCAAGGCTGTCATGA

template   GCAAGGCGTAGCCGTGAGGGGTTTGGTGCATGTTCGGGAATATCGGGGAAAACAGGTGTA
query      GCAAGGCGTAGCCGTGAGGGGTTTGGTGCATGTTCGGGAATATCGGGGAAAACAGGTGTA

template   TCTGCGGTATTTAATCTATATGTGGATACTCCGCTTTGAGGTATTAATCTGTAGGTGACC
```

```
query       TCTGCGGTATTTAATCTATATGTGGATACTCCGCTTTGAGGTATTAATCTGTAGGTGACC

template    GGAAGCATAACTTCTTATTTCCTGACTACAATTATGAATTATTTAAATTAAACAATAATA
query       GGAAGCATAACTTCTTATTTCCTGACTACAATTATGAATTATTTAAATTAAACAATAATA

template    GTGTTTTTAGTTAATGTGCCACATACAGGTAACGCTATGATTCAGACAAAGCCAACAAGT
query       GTGTTTTTAGTTAATGTGCCACATACAGGTAACGCTATGATTCAGACAAAGCCAACAAGT

template    AATACGCTGAAAATGTTATTTATAATGAAACTTAGGGGCGGGGTTTATTTAATAAAACAA
query       AATACGCTGAAAATGTTATTTATAATGAAACTTAGGGGCGGGGTTTATTTAATAAAACAA

template    GGGGGCCTCTGTTTGAATACTTCTTGTTTATAAAATCCCTTTATCTCGACTAAAATATCG
query       GGGGGCCTCTGTTTGAATACTTCTTGTTTATAAAATCCCTTTATCTCGACTAAAATATCG

template    GATGGGGGAAAAGAGTTCTATCATTGTAATCCGGGAGTGGAGTAGGGGTATGTTGACTAC
query       GATGGGGGAAAAGAGTTCTATCATTGTAATCCGGGAGTGGAGTAGGGGTATGTTGACTAC

template    CTGTCAGATCGGCTCCTGTTAATGCTTTCGCTAAAGTTAGAGCTATCTTATCTAATTTCG
query       CTGTCAGATCGGCTCCTGTTAATGCTTTCGCTAAAGTTAGAGCTATCTTATCTAATTTCG

template    CACCATTTAGTTTGGTGTCAGTTAAGTCTGAGCCGAATAGAATTGCAGCGGTTAAGTTTA
query       CACCATTTAGTTTGGTGTCAGTTAAGTCTGAGCCGAATAGAATTGCAGCGGTTAAGTTTA

template    CACCGGACATGTTAGCGCAAGTCAGGTCTGCGTGAGTCAGGTTTGCTTTAGTCATGTTTG
query       CACCGGACATGTTAGCGCAAGTCAGGTCTGCGTGAGTCAGGTTTGCTTTAGTCATGTTTG

template    AACCCATTAGATTTACAGCGTGTAGATTTGCACAACACAAATTTGCACCGACTAAATTTA
query       AACCCATTAGATTTACAGCGTGTAGATTTGCACAACACAAATTTGCACCGACTAAATTTA

template    CATTAGATAGAGTTGTTTCACGGAAATCTGCATTAGAAGCATCTATTTTTGACAAATCTT
query       CATTAGATAGAGTTGTTTCACGGAAATCTGCATTAGAAGCATCTATTTTTGACAAATCTT

template    CCCCCTGGAAATCTTTATGAGCCAGGTTTACTCCGCGCAGGTTCAGAAAACCATCCTCGG
query       CCCCCTGGAAATCTTTATGAGCCAGGTTTACTCCGCGCAGGTTCAGAAAACCATCCTCGG

template    TTATAAGTGAATCAGGCTGTTGTATATTATGTTTACTCATCAGTTTTAACATTCGACATA
query       TTATAAGTGAATCAGGCTGTTGTATATTATGTTTACTCATCAGTTTTAACATTCGACATA

template    CATTCCAAAATCTCTCACTATCAACTTTATCCGTTACAGTTTTTCCATTTTTGTTCACTT
query       CATTCCAAAATCTCTCACTATCAACTTTATCCGTTACAGTTTTTCCATTTTTGTTCACTT

template    CAATAATGACCATGGGATCCGTATTTTCGGATGCTGCTCCACATGACAGACATATAGTAC
query       CAATAATGACCATGGGATCCGTATTTTCGGATGCTGCTCCACATGACAGACATATAGTAC

template    ACCCGTTGACATCCTCCAGAAATATTTTATTACCATCGAAGAAAGCATTTTTATTTACAT
query       ACCCGTTGACATCCTCCAGAAATATTTTATTACCATCGAAGAAAGCATTTTTATTTACAT

template    ATAATAATGATGTGGTCAGTTTTCCAATTACCTCCCGAAACCATCTTTCATTGCTTCTTC
query       ATAATAATGATGTGGTCAGTTTTCCAATTACCTCCCGAAACCATCTTTCATTGCTTCTTC

template    TTACTCCACCACAGGTAAAAAAATTGACAAACCATTCCAGTATACCGCGTGGTGAAGTTG
query       TTACTCCACCACAGGTAAAAAAATTGACAAACCATTCCAGTATACCGCGTGGTGAAGTTG

template    CACTTTTCATTGCTTCTTTCGTACCGGTACCGGCCGCATGCAAATATCTTAATATATTTT
query       CACTTTTCATTGCTTCTTTCGTACCGGTACCGGCCGCATGCAAATATCTTAATATATTTT

template    CTGGGGACGCGTTAGTTATTGGCATTTTGATTCCTTCTTATGGAAGTGAGCCGACTCCTT
query       CTGGGGACGCGTTAGTTATTGGCATTTTGATTCCTTCTTATGGAAGTGAGCCGACTCCTT

template    ATAGGTGTGAGTCAAGTCACATTTATACAAGGAATAATAAAAAAAGATATAAGGTGATTT
query       ATAGGTGTGAGTCAAGTCACATTTATACAAGGAATAATAAAAAAAGATATAAGGTGATTT

template    TTGTATTTAGAGCTTTGGTTGAGTATGAGTAGGTGTCTGGCATCTGACAAGAGGCGATGA
query       TTGTATTTAGAGCTTTGGTTGAGTATGAGTAGGTGTCTGGCATCTGACAAGAGGCGATGA

template    TATCTGCCATAACGGTGAAGCAGAAGTAATATCACCCGTGAATAATAAGATTATCCTTGC
query       TATCTGCCATAACGGTGAAGCAGAAGTAATATCACCCGTGAATAATAAGATTATCCTTGC

template    TATTGACTTCCTTCATTTCCAGCATAGCTTACGCTTCATCATCATTGAATAAACAATTAA
query       TATTGACTTCCTTCATTTCCAGCATAGCTTACGCTTCATCATCATTGAATAAACAATTAA
```

```
template    GTTTGTTGAGCGAAAATTTACTTAAAGAAAAAATAATAAGCACCAATATTTTTTGCAGTA
query       GTTTGTTGAGCGAAAATTTACTTAAAGAAAAAATAATAAGCACCAATATTTTTTGCAGTA

template    TTTCAACTTATACCAGAATAAATGCGCGTAGATGGCGTAAAAACCTGATGAGCAGGAATA
query       TTTCAACTTATACCAGAATAAATGCGCGTAGATGGCGTAAAAACCTGATGAGCAGGAATA

template    CTTACACACTAAAAATGCTCCCGCAAACAGAGCAAAATGAGAGAGATGAACGTTTGCCCC
query       CTTACACACTAAAAATGCTCCCGCAAACAGAGCAAAATGAGAGAGATGAACGTTTGCCCC

template    TTACATTTCCACCAAAGATTCTGGTCTTGTCTCTTGTAAAGGGCATACGTATCGCGTTTT
query       TTACATTTCCACCAAAGATTCTGGTCTTGTCTCTTGTAAAGGGCATACGTATCGCGTTTT

template    ATCTCATTAAGAAAGTATGTTGACGTATTAAATTATGCATAATGCTCTTTCAATTGCTTC
query       ATCTCATTAAGAAAGTATGTTGACGTATTAAATTATGCATAATGCTCTTTCAATTGCTTC

template    ACGTTTGAAATGAATAATTCAAAACCAGTGAGCGCCTCTTCTTCGGTACTGGTTTGCGGC
query       ACGTTTGAAATGAATAATTCAAAACCAGTGAGCGCCTCTTCTTCGGTACTGGTTTGCGGC

template    AAGCGATAAAGCGCCACTAAAGCAGTATTGTCTGCGTCAGCGCCGATAGTGACGGCGCTG
query       AAGCGATAAAGCGCCACTAAAGCAGTATTGTCTGCGTCAGCGCCGATAGTGACGGCGCTG

template    GTGTAGTTCAGACGTAAAAAATGCTGCAAAGTCAGGATGTCGTCAGGCAATGGCATAAAG
query       GTGTAGTTCAGACGTAAAAAATGCTGCAAAGTCAGGATGTCGTCAGGCAATGGCATAAAG

template    GGACAGCACATTTCCAGTGTATGATCGGATTCATTAAAATAAACCTGTATCCCATCATCA
query       GGACAGCACATTTCCAGTGTATGATCGGATTCATTAAAATAAACCTGTATCCCATCATCA

template    ATGATAAGCAGTGGCTCATCTTCTGGCGCATCCAGGCCTAACGCGTCATATAAACGATTT
query       ATGATAAGCAGTGGCTCATCTTCTGGCGCATCCAGGCCTAACGCGTCATATAAACGATTT

template    AATAGACTTTCCATATAGTTACCTCAAGACTCAAGATGTGATTAATGAAGAAATGCCTTT
query       AATAGACTTTCCATATAGTTACCTCAAGACTCAAGATGTGATTAATGAAGAAATGCCTTT

template    TACTGACTGCCAAATATTTTCATCCCCAACTCGTTTTTGATAGGAAAGATTGAGCACCTC
query       TACTGACTGCCAAATATTTTCATCCCCAACTCGTTTTTGATAGGAAAGATTGAGCACCTC

template    TGGCGATAAATTTTTCATTACTTTGTTTCCCGCCCCGCCCGTATTTTGTTTCTGAATCTC
query       TGGCGATAAATTTTTCATTACTTTGTTTCCCGCCCCGCCCGTATTTTGTTTCTGAATCTC

template    CAGGTTACCGCTATTCAGTAATACTTTTTGGAAAATTTTCTGTCCACCGCTATCCGGAAG
query       CAGGTTACCGCTATTCAGTAATACTTTTTGGAAAATTTTCTGTCCACCGCTATCCGGAAG

template    ACTACCAGGCGCACTTAACATATGGGTCTGATGTAAGGAAATGATCTCTCGCTTGATTTC
query       ACTACCAGGCGCACTTAACATATGGGTCTGATGTAAGGAAATGATCTCTCGCTTGATTTC

template    TGAATCCATCATCCCTGTACGATCTTTGCCGCTTTTACAATTCCAGGCGGGTACCGCGTC
query       TGAATCCATCATCCCTGTACGATCTTTGCCGCTTTTACAATTCCAGGCGGGTACCGCGTC

template    AATTTCATGGGCTAACATGGCAAGGCGTTGTGCGAGTTTATAGGGTTCGCCGCCATCTTT
query       AATTTCATGGGCTAACATGGCAAGGCGTTGTGCGAGTTTATAGGGTTCGCCGCCATCTTT

template    ATGATGTTGGTTATTTTTCCATATATCCTTAATCTGGCGCGCTAATGTATTGACGACCTC
query       ATGATGTTGGTTATTTTTCCATATATCCTTAATCTGGCGCGCTAATGTATTGACGACCTC

template    ATAATTATCCGGGTATTGCGCCAGCCATTCGCCAACCCAGCCACCTGGTCTGGCTTCAGG
query       ATAATTATCCGGGTATTGCGCCAGCCATTCGCCAACCCAGCCACCTGGTCTGGCTTCAGG

template    GCGTAAATCATTGCCTAATAACTGATGTAGCGCCTCGGCATTATAGCTATCCGATGCCTT
query       GCGTAAATCATTGCCTAATAACTGATGTAGCGCCTCGGCATTATAGCTATCCGATGCCTT

template    AAGGCCAAAGCCGAGCTTGAGCGCCAGCTCATTAACACCCACATTAAATGCGGCGACGTC
query       AAGGCCAAAGCCGAGCTTGAGCGCCAGCTCATTAACACCCACATTAAATGCGGCGACGTC

template    CGGTTTTATTTTTACCGTCTGTAGATCGCCATCTTTATTGCGGATTTTTAAATGAATCAT
query       CGGTTTTATTTTTACCGTCTGTAGATCGCCATCTTTATTGCGGATTTTTAAATGAATCAT

template    TTTTCCCGGCTGGGTCAACGATTGCCATGCGCGCATTTGGTCCTCGACCATCGTTCCCTC
query       TTTTCCCGGCTGGGTCAACGATTGCCATGCGCGCATTTGGTCCTCGACCATCGTTCCCTC
```

```
template  TTTGCCGAAAATATTCGACGCGGTGAGTAACCCGACGGATACCAGTTTCAGGCTTACCGC
query     TTTGCCGAAAATATTCGACGCGGTGAGTAACCCGACGGATACCAGTTTCAGGCTTACCGC

template  CTCGCCCGCTAAGGCTTTGTTAAGCAACTCAGGTTTACTAAAAAGTGCCGCAGTTAATAC
query     CTCGCCCGCTAAGGCTTTGTTAAGCAACTCAGGTTTACTAAAAAGTGCCGCAGTTAATAC

template  TTCTTTGGCTTTGTTTTCAGCGCCGACGTGACGCAGAAGCGGATCTTTTTCATGATAGGG
query     TTCTTTGGCTTTGTTTTCAGCGCCGACGTGACGCAGAAGCGGATCTTTTTCATGATAGGG

template  GGAAAGCACGCCATGACGTATCCCGCAAAAAAGCGTTTTATCTTTACCGTCCTCATGCAC
query     GGAAAGCACGCCATGACGTATCCCGCAAAAAAGCGTTTTATCTTTACCGTCCTCATGCAC

template  ACTCACCGTGGACATCCACAAATTATTGGCGTGATGAATATTCTTGGTATCCCAACTGCA
query     ACTCACCGTGGACATCCACAAATTATTGGCGTGATGAATATTCTTGGTATCCCAACTGCA

template  TACGCCCTTTCCCTCATAAGCACTGGGAAAGATATCTTTTGCGCCGATTTTCATCTCTGC
query     TACGCCCTTTCCCTCATAAGCACTGGGAAAGATATCTTTTGCGCCGATTTTCATCTCTGC

template  TGCAGGGAGCTGCGTGTTGGTATAGTGATGCCCGTTATGCGTGAGTGTATTTTTTATGGT
query     TGCAGGGAGCTGCGTGTTGGTATAGTGATGCCCGTTATGCGTGAGTGTATTTTTTATGGT

template  TTGCCAGGGCTGGTTATTAAGCTGCTTGACCTGAGCATCTCTAAACGCTACTGCCGCAAA
query     TTGCCAGGGCTGGTTATTAAGCTGCTTGACCTGAGCATCTCTAAACGCTACTGCCGCAAA

template  GCGATGAGCTTCTTTTGCAGGTAAGCCATCCTGCTGCTGAGTGCGTGCTGCAATAAGTTC
query     GCGATGAGCTTCTTTTGCAGGTAAGCCATCCTGCTGCTGAGTGCGTGCTGCAATAAGTTC

template  GATAAGATTTTTCTTCAATGCTTTTGCCGCGGCTTCTTCTTGTTTTGTTGCTGGCCGGTC
query     GATAAGATTTTTCTTCAATGCTTTTGCCGCGGCTTCTTCTTGTTTTGTTGCTGGCCGGTC

template  CGCTTTAACTTTGGCTAACTCCATTTGGTTTGCCATTGACGTTAGAACCGGGTCTTGTTT
query     CGCTTTAACTTTGGCTAACTCCATTTGGTTTGCCATTGACGTTAGAACCGGGTCTTGTTT

template  ACCCAGAACGGTTGCCGCGACGGTAAGAAGATCGCGCTGTAAGTTATAGAGGTTATGCAG
query     ACCCAGAACGGTTGCCGCGACGGTAAGAAGATCGCGCTGTAAGTTATAGAGGTTATGCAG

template  CGAGTGGTTAGACGCCTTCTGATGCTGTAGATAATTCCCCCATGTCGCGCCGGGTTCTCG
query     CGAGTGGTTAGACGCCTTCTGATGCTGTAGATAATTCCCCCATGTCGCGCCGGGTTCTCG

template  CAGGACAATAATTTCCGGGCGAGCGTCGGGCGCTTTAGCCGGCGCTTTGCCCTGGCCTGA
query     CAGGACAATAATTTCCGGGCGAGCGTCGGGCGCTTTAGCCGGCGCTTTGCCCTGGCCTGA

template  GAGAATCTGCATTCCGTTGTATAAGGTTTTTTGTAGGCTTTTAAAAGCCTCCTGGGTTTT
query     GAGAATCTGCATTCCGTTGTATAAGGTTTTTTGTAGGCTTTTAAAAGCCTCCTGGGTTTT

template  TAGTGAAGCTGAGTGATAGAAGCTCTGTATTTGCATAGCGTTTTTAATATTCCTGAATAG
query     TAGTGAAGCTGAGTGATAGAAGCTCTGTATTTGCATAGCGTTTTTAATATTCCTGAATAG

template  GGGAGTGGGAACATTCAACAGGGTTAACAACCCTTTAAAAGGATGTGACTTTTATGCACC
query     GGGAGTGGGAACATTCAACAGGGTTAACAACCCTTTAAAAGGATGTGACTTTTATGCACC

template  AGGAACTTTAAAAAAATGTCCTTTTTAATGAGGAAACGCTTCTGATCAGGCGGGGAGGCA
query     AGGAACTTTAAAAAAATGTCCTTTTTAATGAGGAAACGCTTCTGATCAGGCGGGGAGGCA

template  TCCTGACGTCCATACGTAGTGGCGATCATGGGGGAGATCGGTACATCAAATGTTCATAAA
query     TCCTGACGTCCATACGTAGTGGCGATCATGGGGGAGATCGGTACATCAAATGTTCATAAA

template  ATCTACAAACTTCACAATTTATGGCACAACTTTTATTGAAAACCCACCTTCTTGACTCAG
query     ATCTACAAACTTCACAATTTATGGCACAACTTTTATTGAAAACCCACCTTCTTGACTCAG

template  ATCAAATTCACCGATTTACTCCTGCGTAAAGTAACGGCGTTACATCAAATGTTATACAGG
query     ATCAAATTCACCGATTTACTCCTGCGTAAAGTAACGGCGTTACATCAAATGTTATACAGG

template  TGTGTGTATGCCAGCAAAGCCCCGGACAAGTAAGACCGTGACGAAGAATATTCGTTTTTC
query     TGTGTGTATGCCAGCAAAGCCCCGGACAAGTAAGACCGTGACGAAGAATATTCGTTTTTC

template  CTATTCCATGCTTGAACAGATAGAATTCGCGTTGAAATCTGAAAAGACGCGGAATTTTTC
query     CTATTCCATGCTTGAACAGATAGAATTCGCGTTGAAATCTGAAAAGACGCGGAATTTTTC

template  AGCATGGGTAAAAGAGGCCTGTCGGGAAAAGTTATGTAACACGGGACATAAGCTGTAGTT
```

```
query     AGCATGGGTAAAAGAGGCCTGTCGGGAAAAGTTATGTAACACGGGACATAAGCTGTAGTT

template  ATCGTCTTATGTAAGAGGGGGCGGCCATTTTGAAAAGAACAACGTGCTTATATACCTCCT
query     ATCGTCTTATGTAAGAGGGGGCGGCCATTTTGAAAAGAACAACGTGCTTATATACCTCCT

template  GGGTTTTTGCCGCTTTTGTCTCTCTGCTGATATTTGCCTGGAGTGTCATAAACTATCCTC
query     GGGTTTTTGCCGCTTTTGTCTCTCTGCTGATATTTGCCTGGAGTGTCATAAACTATCCTC

template  TCTATGAATCCATAATAATTATTGTCTTTTATATCTGGCTAATTCTGGTACCGCTTTATC
query     TCTATGAATCCATAATAATTATTGTCTTTTATATCTGGCTAATTCTGGTACCGCTTTATC

template  TTATTGTGTATGAGTGGCTAATAGATTGTCATTAAATTTATGGCTGCGTACTTGAAAAGT
query     TTATTGTGTATGAGTGGCTAATAGATTGTCATTAAATTTATGGCTGCGTACTTGAAAAGT

template  GATATTTCATATCTGTACGGTAAGTCATTGTCGTAATAATATTATTGGTCAGGCGACGGG
query     GATATTTCATATCTGTACGGTAAGTCATTGTCGTAATAATATTATTGGTCAGGCGACGGG

template  CGAGCGTCTGCGCATTTTGCATCGTTTTATCTTCAAAATTTTGCAGTAAGCGTTGCGCTT
query     CGAGCGTCTGCGCATTTTGCATCGTTTTATCTTCAAAATTTTGCAGTAAGCGTTGCGCTT

template  CTTTCGGATGCGACGCATATAATCTCAGATAGCTCTGCTCCATCTTATACTGCTGCTTAG
query     CTTTCGGATGCGACGCATATAATCTCAGATAGCTCTGCTCCATCTTATACTGCTGCTTAG

template  CTGTTTGCTGTTCAAATGTTTTCCAGGCGTGTTGCACATCTGGCGCAAACGCATTGTAGT
query     CTGTTTGCTGTTCAAATGTTTTCCAGGCGTGTTGCACATCTGGCGCAAACGCATTGTAGT

template  CCTGCATAACCAGCGTTTGCAGCGTGCGGAATGTCCAGTAGGTAGAGTCGTTGCTCGCCC
query     CCTGCATAACCAGCGTTTGCAGCGTGCGGAATGTCCAGTAGGTAGAGTCGTTGCTCGCCC

template  GATCGGTTCCTTTATCATCTCCGGGTTGATAATGACGCATCCCCTGATAATAGGGGAGAT
query     GATCGGTTCCTTTATCATCTCCGGGTTGATAATGACGCATCCCCTGATAATAGGGGAGAT

template  AGACGCTAAGAGATGGCATTCCATAGGCGATGTATTCTACGTTGCCGATAGCCTGCGGTA
query     AGACGCTAAGAGATGGCATTCCATAGGCGATGTATTCTACGTTGCCGATAGCCTGCGGTA

template  ATTTCGGTCTGACCTGTAAAATATGTGACTCCTGGGTACGAAAAACGGATATAGGTCGCC
query     ATTTCGGTCTGACCTGTAAAATATGTGACTCCTGGGTACGAAAAACGGATATAGGTCGCC

template  ATGGTTCTTGTGGATTATGACTGGCATAAGGGTCGTGCGACGTTCCCTGATAGTGATTGC
query     ATGGTTCTTGTGGATTATGACTGGCATAAGGGTCGTGCGACGTTCCCTGATAGTGATTGC

template  GTAGCGCGTTTTTTACTGCCGCCACGCTGATCTTCGTTATTGGCGTTAAAAAAACAGGAA
query     GTAGCGCGTTTTTTACTGCCGCCACGCTGATCTTCGTTATTGGCGTTAAAAAAACAGGAA

template  ATGTTTCCCCTTCGCTAACGACCGTATCCAGATGCGGATTAAACTGGTGTTGTAGCGTCC
query     ATGTTTCCCCTTCGCTAACGACCGTATCCAGATGCGGATTAAACTGGTGTTGTAGCGTCC

template  AGACGCGCGGATAATTATAGGTGGTATCGTTTTTGTTATCCTGCGAATAGGCTTGATGAA
query     AGACGCGCGGATAATTATAGGTGGTATCGTTTTTGTTATCCTGCGAATAGGCTTGATGAA

template  AGTCGAATTCGCCGCGGGCCGGATCATATAATCCCTGCTTTTTCGCAAAGCTTACTAACG
query     AGTCGAATTCGCCGCGGGCCGGATCATATAATCCCTGCTTTTTCGCAAAGCTTACTAACG

template  TTGGTGACGCCATATAATTCGCGTTATCATTCGGATCGTAATGGCGTAAACGTCCCTGAT
query     TTGGTGACGCCATATAATTCGCGTTATCATTCGGATCGTAATGGCGTAAACGTCCCTGAT

template  TGGCGGAAACGAAATAGCTATCTGCCGGAAGTCGTACTGCCAGCCATTGATGACCGCTTC
query     TGGCGGAAACGAAATAGCTATCTGCCGGAAGTCGTACTGCCAGCCATTGATGACCGCTTC

template  CCGTCTCCAGATACCATATCTCTTTGCTATCAATAAACGCGACGCCGAAACCTTCGCCCG
query     CCGTCTCCAGATACCATATCTCTTTGCTATCAATAAACGCGACGCCGAAACCTTCGCCCG

template  CGCCTTTTTGTTCAATAATATCTCCCAGTAATTTGGCGCCCTGACGCGCCGATTGCGCCA
query     CGCCTTTTTGTTCAATAATATCTCCCAGTAATTTGGCGCCCTGACGCGCCGATTGCGCCA

template  CTGGCAGGATCACGGACTCAATGGCGTCTTCCGTGATTCCCGTTTTTGTCACGTAAGGAT
query     CTGGCAGGATCACGGACTCAATGGCGTCTTCCGTGATTCCCGTTTTTGTCACGTAAGGAT

template  CGGCAGCCAGCGCCGCTCTGCCGTTATAAATGGTTTCCGTTGCGCTCATTCCGACGCCCG
query     CGGCAGCCAGCGCCGCTCTGCCGTTATAAATGGTTTCCGTTGCGCTCATTCCGACGCCCG
```

```
template   CCGAATTGAAACCGGCTTCACCCATGGCGTTATCGTTAGTATCAAAGTCATGAATCGCCG
query      CCGAATTGAAACCGGCTTCACCCATGGCGTTATCGTTAGTATCAAAGTCATGAATCGCCG

template   TATAGCGCATCGCTGTCTCCGGAAGCGGCCAGCTAAAATTGTTCCGATGTGCTTTATACT
query      TATAGCGCATCGCTGTCTCCGGAAGCGGCCAGCTAAAATTGTTCCGATGTGCTTTATACT

template   CGCCTTGTTGATGAAACGCGATGGGATGAATAACCTTATGCTTGGCGTTATTTGCCGAGC
query      CGCCTTGTTGATGAAACGCGATGGGATGAATAACCTTATGCTTGGCGTTATTTGCCGAGC

template   CATCTTCGTTGCGCGCGATAATGAAGGAGCCGTCAGCCGAAGCCTGATTGCCTACCAAAA
query      CATCTTCGTTGCGCGCGATAATGAAGGAGCCGTCAGCCGAAGCCTGATTGCCTACCAAAA

template   GGGTAGTACAGGCGATGACTTTACCCATACCCAGCAGCGTAACGGCGAATGCAAGATACT
query      GGGTAGTACAGGCGATGACTTTACCCATACCCAGCAGCGTAACGGCGAATGCAAGATACT

template   TTTTCATAAAGGTTCCCACTGAATAACGCATTATGGGATGAATTGACCCTGGATTGGAAA
query      TTTTCATAAAGGTTCCCACTGAATAACGCATTATGGGATGAATTGACCCTGGATTGGAAA

template   CCGAGAAAGTGATCGAGCCAGCAATATTCTTTGCCGGCATCCTTTATTTTCTCTTTATTG
query      CCGAGAAAGTGATCGAGCCAGCAATATTCTTTGCCGGCATCCTTTATTTTCTCTTTATTG

template   AGGTTGTATTGATAACCACAGCCCTGTGGCAGGGAAGGGGAACAGAACCTGTCCTGACCT
query      AGGTTGTATTGATAACCACAGCCCTGTGGCAGGGAAGGGGAACAGAACCTGTCCTGACCT

template   TAGCTATCACCACTATCAGGCAGACGAACCGAAAAGATATTATGTTCATCGGCATAGCGG
query      TAGCTATCACCACTATCAGGCAGACGAACCGAAAAGATATTATGTTCATCGGCATAGCGG

template   TAAGATGCCGAGCCACCGTGTAATAGAGCAATCGCGTTAACTAACGATAACCCCAGGCCG
query      TAAGATGCCGAGCCACCGTGTAATAGAGCAATCGCGTTAACTAACGATAACCCCAGGCCG

template   AAACCGGCAGTGTGGCGGGCATTATCTCCTCGCCAAAAACGCCGGAAAAGCTTATCCGCA
query      AAACCGGCAGTGTGGCGGGCATTATCTCCTCGCCAAAAACGCCGGAAAAGCTTATCCGCA

template   TCGGCGGGGTGGCTGCCTGGATTAGCGACCCGAATTTCTGCAACGTTATCATCATAAGCG
query      TCGGCGGGGTGGCTGCCTGGATTAGCGACCCGAATTTCTGCAACGTTATCATCATAAGCG

template   CTTTCAATACGTATCACGGCGTTTTCATCAGAATAACGGATGGCATTCGTCAGCAGGTTT
query      CTTTCAATACGTATCACGGCGTTTTCATCAGAATAACGGATGGCATTCGTCAGCAGGTTT

template   GAGAGCACTCTTTGTAATAATATTTCGTCAGCCCATACCGTTCCCTGACATTGATTTATA
query      GAGAGCACTCTTTGTAATAATATTTCGTCAGCCCATACCGTTCCCTGACATTGATTTATA

template   AAACAGATGCGCTTCTCCTCGGCAAGGGGGCTAAGATAATCCAGCATATTTTCGACCAGC
query      AAACAGATGCGCTTCTCCTCGGCAAGGGGGCTAAGATAATCCAGCATATTTTCGACCAGC

template   GCATTGAGCGAAACAGGCTGTTTTTTTACCGCTATATTCTGGTGCTCCGCGCGTGCCAGA
query      GCATTGAGCGAAACAGGCTGTTTTTTTACCGCTATATTCTGGTGCTCCGCGCGTGCCAGA

template   AAGAGAATATTTTCTGTCAGTCGCGACAGTCCCTCCAGCTCTTCAATATTATCGACAAGG
query      AAGAGAATATTTTCTGTCAGTCGCGACAGTCCCTCCAGCTCTTCAATATTATCGACAAGG

template   GCTTGTTGATACTCTTCGGCGCTGCGTTCCTGACTCAGCATCACCTGATTCTTCCCCAGT
query      GCTTGTTGATACTCTTCGGCGCTGCGTTCCTGACTCAGCATCACCTGATTCTTCCCCAGT

template   AAAATATTAACCGGCGTGCGCAGCTCATGCGCCAGATCGTCGGCAAATTGGTTCAGGCGT
query      AAAATATTAACCGGCGTGCGCAGCTCATGCGCCAGATCGTCGGCAAATTGGTTCAGGCGT

template   TCAAAATCGTCGGAAAGCTTCTGGCGCATGGTATTTAGCGCTTGTCCAAGCGGCCTGAGC
query      TCAAAATCGTCGGAAAGCTTCTGGCGCATGGTATTTAGCGCTTGTCCAAGCGGCCTGAGC

template   TCAACGGGTAACGCCTGTTCCGCCAACGGCTGGCGAAGTGTGCCGCTATCTGTCGCCGCG
query      TCAACGGGTAACGCCTGTTCCGCCAACGGCTGGCGAAGTGTGCCGCTATCTGTCGCCGCG

template   GTGAGTCGGCTGAGCGACGTAATGGCCCGCAGCCCGTTTCTGATGACTAATGGACTGAGC
query      GTGAGTCGGCTGAGCGACGTAATGGCCCGCAGCCCGTTTCTGATGACTAATGGACTGAGC

template   GCCGAACAGACGAGGATCGCGATAAGGCTCATCAGCAAACTGTTGCGGCGATATTGCGCC
query      GCCGAACAGACGAGGATCGCGATAAGGCTCATCAGCAAACTGTTGCGGCGATATTGCGCC
```

```
template   AGCATTTGCCGCCTTTCCGTCGCCAGCCTGGCAATAGTAAGGGTCAGCGGGTCATCGCCG
query      AGCATTTGCCGCCTTTCCGTCGCCAGCCTGGCAATAGTAAGGGTCAGCGGGTCATCGCCG

template   CTTCTGGCGCTTACGCGTACCGCGGTCAGTTCCGTGCCTTGTACCGCCTGGCGAAATAAG
query      CTTCTGGCGCTTACGCGTACCGCGGTCAGTTCCGTGCCTTGTACCGCCTGGCGAAATAAG

template   GTTTCGCGGGTGATGTTTTTAGTCAGCGGAATCTCGTTAAAGCGTTGGTCGGGGATGCCG
query      GTTTCGCGGGTGATGTTTTTAGTCAGCGGAATCTCGTTAAAGCGTTGGTCGGGGATGCCG

template   CTATGATTAATCGCAACATTGTGGCCTGTTGCTGAATGGATCAATAAGATGTCCTGCTTC
query      CTATGATTAATCGCAACATTGTGGCCTGTTGCTGAATGGATCAATAAGATGTCCTGCTTC

template   GTATCCACCATCCGATTGAAATAGAGCGGCAGATTTTCCGGCCTGGCGCCATCCAGTAAC
query      GTATCCACCATCCGATTGAAATAGAGCGGCAGATTTTCCGGCCTGGCGCCATCCAGTAAC

template   AGTTGCTGCATTTGCGCCGCCCGATTTATTAGCGTCATATCATCCCGATACGTTAATTCT
query      AGTTGCTGCATTTGCGCCGCCCGATTTATTAGCGTCATATCATCCCGATACGTTAATTCT

template   TTGCTCAGCGCGTTATAGAGCGTCCAGCTAATGCCGGTACAGGCGAGGATAAGGATGGCG
query      TTGCTCAGCGCGTTATAGAGCGTCCAGCTAATGCCGGTACAGGCGAGGATAAGGATGGCG

template   ATAAAAGAAATTGTCAGGCGCAGCGTCATTGATAACTTAACCATTCTGCGACGTTTCCGC
query      ATAAAAGAAATTGTCAGGCGCAGCGTCATTGATAACTTAACCATTCTGCGACGTTTCCGC

template   CTGTAATCGATAACCCATCCCCCGGACGGTCATAATGAGCTTCTTTTCAAATGGATCGTC
query      CTGTAATCGATAACCCATCCCCCGGACGGTCATAATGAGCTTCTTTTCAAATGGATCGTC

template   TACTTTGGCGCGCAGCCGACGAATCGCGACATCAACGGTGTTGGTTTCACTATCAAAGTT
query      TACTTTGGCGCGCAGCCGACGAATCGCGACATCAACGGTGTTGGTTTCACTATCAAAGTT

template   AATTCCCCAAACTTCGCTGGCGATCGCGGTTCGGGGCACGATTTCTCCTGCCCGGGACGC
query      AATTCCCCAAACTTCGCTGGCGATCGCGGTTCGGGGCACGATTTCTCCTGCCCGGGACGC

template   CAGTAACCAGAGGAGCAGGAATTCTTTGCGGGTCAGGGAAATCGGTTTGCCATTTCGTGA
query      CAGTAACCAGAGGAGCAGGAATTCTTTGCGGGTCAGGGAAATCGGTTTGCCATTTCGTGA

template   CACTGATTGCTTTGTGGCATCCATGTCCAGACCATTGATCGTCAGTCGAGTAAAGGCAGG
query      CACTGATTGCTTTGTGGCATCCATGTCCAGACCATTGATCGTCAGTCGAGTAAAGGCAGG

template   GACATGCTGTCTGAGTTGAGCTCTCACCCGGGCCAGCAGTTCGGCGAAGGAAAAAGGCTT
query      GACATGCTGTCTGAGTTGAGCTCTCACCCGGGCCAGCAGTTCGGCGAAGGAAAAAGGCTT

template   AACAAGGTAATCATTAGCGCCCGCCTCAAGACCTTTGACGCGATCCTCAACCGAGTCGCG
query      AACAAGGTAATCATTAGCGCCCGCCTCAAGACCTTTGACGCGATCCTCAACCGAGTCGCG

template   CGCCGTCAGGCAAATAACAGGGGACTGATGCGCAGTGCGTAACGCGCGTAAAACCTGCCA
query      CGCCGTCAGGCAAATAACAGGGGACTGATGCGCAGTGCGTAACGCGCGTAAAACCTGCCA

template   TCCATCAAGCCCCGGCAGCATAATATCAAGAATAATCAATGAATAATGTTCCTGAAGGGC
query      TCCATCAAGCCCCGGCAGCATAATATCAAGAATAATCAATGAATAATGTTCCTGAAGGGC

template   CAGGTGTAATCCGTCTCGTCCATCACAGGCATAATCAACCACATAGCCTGCTTCCGTGAG
query      CAGGTGTAATCCGTCTCGTCCATCACAGGCATAATCAACCACATAGCCTGCTTCCGTGAG

template   TCCCTGACGTACCCACTCAATGGTTTTCTGGTTATCTTCAATCAATAAAATCTTCATCAA
query      TCCCTGACGTACCCACTCAATGGTTTTCTGGTTATCTTCAATCAATAAAATCTTCATCAA

template   CGCAGTATGCCATAGGCTTGTTAGCGAATCCGTAAATCTCCAACAAGATGACATAATTGT
query      CGCAGTATGCCATAGGCTTGTTAGCGAATCCGTAAATCTCCAACAAGATGACATAATTGT

template   CATGTCCGT
query      CATGTCCGT
```

SPI-9_NC_003198

```
template   ATGCGTCTACTCGCCGTGGTTTCGAAATTGACTGGCGTCTCCACCACTGTGGAATCCTCA
query      GCGGACGGAAAAACGGTAAGCGGGCAAGCCGAAGCGGGCAGTACCATTACCATCAAAGAT
```

```
template  GCGGTCACTCTTAACGCGCCTTCAATTGTTAAATTATCAGTGGCCCGGGATGAAATTAGT
query     CCCTCAGGCAACGTCGTTGGCGAGGGCAAAGCGGATAGCGACGGTAAATTTAGTATTGAT

template  CAACTTACGCGCATTAATCAGGATCTGGTGGTGAGGCTCCATTCCGGCGAAACGATCACG
query     CTGACAGCGCCACAGATTAGCGGCGAACAACTTACCGTGACCGCGACTGACGATGCCGGC

template  ATTAAAAACTTTTACGTTACCAACGATCTGGGCGCAAGCCAGCTGGTACTGGCGGAAAAC
query     AATACCGGCCCATCGGCAACCATTGATGCGCCCAACATTCCTCTCCCCGATACACCGGTT

template  GATGGCACGTTATGGTGGGTGGAAAATCCGCAAGCCGGGCTACATTTTGAACAAATCGCT
query     ATCACCGCCGCTATCGATGATGCCGCTCCCCTCACCGGCACGCTGAGCAATAATCAGTTT

template  GATATTAATGAGCTGCTGGTCACTTCCGGCGCTTCCCATGAAGCAGGCGGCGCCGTTTGG
query     ACGAACGACAATACCCCCACTCTGGAGGGCACCGGCAGCGCAGGCACAGTCATCCATATT

template  CCGTGGGTACTGGCTGGCGCGGTGGCGGCTGGCGGCATTGCCGCTATCGCGTCTTCCGGC
query     TACGCCAATGGTCAGGAAATAGGCTCAACAACGGTTGATACCAGCGGAAACTGGCATTTT

template  GGCGGCGATTCCCACCATCATTCGGATGGCGATAATCCGCCCCCCGATAACACCAATCCT
query     GCCATTACCAGCGCGCTAGCGGATGGGGAAAATCATTTCACCGCCATTGCGACTAACGTG

template  GACGGTAATCCCCCTGATAACAGCAATCCCGGCGGCAGTAACCCTAACGGCAATACTCCA
query     AAAGGCGAAAGTAGCGAATCAGCCCGCTTTACGCTGACTATCGACACACTCATCCCCGAT

template  GGTAGCAGTAATCCTGTAGATACTACCCCGCCTCTCGCTCCCGGCGAATTATTGATTTCA
query     GCCCCACGTGTTGAACTGATTGCCGATAACACCAGTTTGCTCACCGGGCCGCTACAGAAT

template  GCGGACGGAAAAACGGTGAGCGGCGAGGCCGAAGCGGGCAGTCTCATTACCATCAAAGAT
query     AATGACCGAACTGACGAGGCAAAACCGCTATTTTCCGGGCAGGGAGACGCAGGCAATACC

template  CCCTCAGGCAACGTCGTTGGCGAGGGCAAAGCGGATAGCGACGGTAAATTTAGTATTGAT
query     ATCACGATTAAAGAAGGTTCAACCGTTATCGGCAGCGCTACCGTAGACGAAAATGGACGC

template  CTGACAGCGCCACAGATTAGCGGCGAACAACTTACCGTGACTGCGACTGACGATGCCGGC
query     TGGACCTTTACGCCGACTACGCCGTTAAGCGATGGCGAACATACCTTTACCGTCGAACAA

template  AATACCGGCCCATCCGCAACCATTGATGCGCCCAACATTCCTCTCCCCGATACACCGGTT
query     AGCGACAAAGCCGGAAACACGAGCCGCGTGACGACAACGCCTACTATCATTGTGGACACC

template  ATCACCGCCGCTATCGATGATGCCGCTCCCCTCACCGGCACGCTGAGCAATAATCAGTTT
query     ACGCCGCCTGACGCCGCTATCATTGATAATGTTGCGAAAGACGGCACAACCGTTAGCGGC

template  ACGAACGACAATACCCCCACTCTGGAGGGCACCGGCAGCGCAGGCACAGTCATCCATATT
query     ACCGCTGAAGCTGGCAGTACTGTGTCGATCTATGACCCGGCGGGAAATTACCTGGGCTCC

template  TACGCCAATGGTCAGGAAATAGGCTCAACAACAGTTGATACCAGCGGAAACTGGCATTTT
query     ACGATTACCGGAGAAAATAACCACTTCAGCATCACGCTGAATCCGGCTCAGACCCACGGC

template  GCCATTACCAGCGCGCTAGCGGATGGGGAAAATCATTTCACCGCCATTGCGACTAACGTT
query     GAGCGTCTGGAAGCGCGTATTCAGGACGCCGTCGGTAACATCGGCCCCGCCACGGAGTTT

template  AAAGGCGAAAGTAGCGAATCAGCCCGCTTTACGCTGACTATCGACACACTCAGCCCCGAT
query     ACCGCTTCTGACTCACAGTATCCTGCCCAGCCGACTATCCTTACCGTGACGGATGACGCT

template  GCTCCACGCGTTGAACTGATGGCCGATAACACCGGTTTGCTCACCGGACCGCTACAGAAT
query     GGCGCCGTTACCGGGCTGCTGAAAAATGGCGATGCCACAGATGATAACCGCCCAACCCTC

template  AATGACCGGACTGACGAGGCAAAACCGCTATTTTCCGGGCAGGGAGAGGCAGGCAATACC
query     AGCGGTACTGCTGAACCAGGCAGTACGATATCGATTAACGATAATGGCTTTCCTGTAGCG

template  ATCACGATTAAAGAGGGTTCAACCGTTATCGGCAGCGCTACCGTAGACGAAAATGGACGC
query     ACCTTTCCGCCCATTGTCGCTGACGCTGACGGCAAATGGAGCTTTACCCCCTCGCTGGCG

template  TGGACCTTTACGCCGACTACGCCGTTAAGCGATGGCGAACATACCTTTACCGTCGAACAG
query     CTTGCCGATGGCGACCATGTCTTTACCGCTACCGCGACCAACGATCGCGGCACCAGCGGG

template  AGCGACAAAGCCGGAAACGCAAGCCGCGTGACGACAACGCCTACTATCATTGTGGACACC
query     CAGTCCGTCTCCTTTACCATTGATATCGACACGCAGCCGCCGGTGCTGGAAGGCCTGGCG

template  ACGCCGCCGGACGCCGCTATCATTGATAATGTCGCGAAAGACGGCACAACCGTTAGCGGC
```

```
query     GTTAGCGACGTCGGCGACAGACTCACCGGCACTACGGAAGCTGGCAGTACTGTGGTTATC

template  ACCGCTGAAGCTGGCAGTACCGTGTCGATCTATGACCCGGCGGGAAATTACCTGGGCTCC
query     AAAGACAGCCTGGGAAATACGCTCGGGAGTGGAACGGCAGGCGACGACGGTACCTTCTCA

template  ACGATTACCGGAGAAAATAACCACTTCAGCATCACGCTGAATCCGGCCCAGACCCACGGC
query     ATAGGTATTAGCCCGGCGAAAATTAACGGCGAAACATTAAGCATTAGCGTTACCGATAAA

template  GAGCGTCTGGAAGCGCGTATTCAGGACGCCGTCGGTAACATCGGCCCCGCCACAGAGTTT
query     GCCGCGAATAGCGGTCCGGTAGAAACGCTGAACGCGCCGGATAAAACTGCGCCTGCGGCA

template  ACCGCTTCTGACTCACAGTATCCTGCCCAGCCGACTATCCTTACCGTGACGGATGACGCT
query     CCGGACGGTCTTACCGTGGCGACCGACGGTCTGTCCGTAAGCGGTCAGGCGGAAGCCGGG

template  GGCGCCGTTACCGGGCTGTTGAAAAATGGCGATGCCACAGATGATAACCGCCCAACCCTC
query     GCAACGGTCACTATCCGCGACAGTAGCAACACCGTACTTGGCAGCGCCGTCGCTAATGGC

template  AGCGGTACTGCTGAACCAGGCAGTACGATATCGATTAACGATAATGGCTTTCCTGTTCCG
query     AACGGACAATTTATCGTTCCGCTGAATACGGCGCAGACTAACGGCCAGGCACTTATCGCT

template  AGCTTTCCGCCCATTGTCGCTGACGCTGACGGCAAATGGAGCTTTACCCCCTCGCTGGCG
query     ACCGCCACCGATGTCGCGAAAAACGAAAGCGCCGCCGCGACGGTTATCGCGCCGGACAGT

template  CTTGCCGATGGCGACCATGTCTTTACCGCTACCGCGACCAACGATCGCGGCACTAGCGGG
query     ACCGCGCCGGAAATGCCGAAAAACGTGGTAATTAGTGAGGATGGCACCAGTATCAGCGGC

template  CAGTCCGTCGCCTTTACCATTGATATCGACACGCAGCCGCCGGTGCTGGAAGGCCTGGCA
query     ACCGCCGAACCGGGTAGCGCCATCACGATCGCCACGCCGGACGGCAAGCCGCTTGGCAGC

template  GTTAGCGACGTCGGCGACAGACTCACCGGCACTACGGAAGCTGGCAGCACTGTGGTTATC
query     GGCAAAGCAGATGGCGAAGGTCATTTTACCCTTCCCCTCGTCCCCGCACAGACCAACGGC

template  AAAGATAGCCTGGGAAATACGCTCGGGAGTGGAACGGCAGGCGACGACGGCACCTTCTCA
query     GAACAGGTTACCGTCACCGCCACCGACAGCGCCAACAACGTCAGCCCGCCAACCACAGCG

template  ATAGGTATTAGCCCGGCGAAAATTAACGGTGAAACATTAAGCATTAGCGTTACCGATAAA
query     CAAGCGCCCGATATCACCGCCCCGGATAAGCCCATTATCACTCAGGTACTGGACGATGTT

template  GCCGCGAATAGCGGTCCGGTAGAAACGCTGAACGCGCCGGATAAAACTGCGCCTGCGGCA
query     GAAAGCTTCACCGGGCCGCTGGTTAACGGACAAACCACCAACGACAACCGTCCCACCCTT

template  CCGAACGGTCTTATCGTGGCGACCGACGGTCTGTCCGTAAGCGGTCAGGCGGAAGCCGGG
query     AGCGGTACGGCGGAGGCCGGCGCGCGTGTCGAAGTCTTTGATAACGGCTTTTCACTGGGA

template  GCAACGGTCACTATCCGCGACAGTAGCAATACCGTACTTGGCAGCGCCGTCGCTAACGGC
query     CTCGCCACGCTACAGCCCAACGGTGCCTGGACGTTTACGCCGTCGCAAAATTTAGGTGAA

template  AACGGACAATTTATCGTTCCGCTGAATGCGGCGCAGACTAACGGCCAGGCGCTTATCGCT
query     GGCGCGCATCGACTGACCGTAATCGCAACCGACGCTAAAGGCAATGCCAGTCAGGCCGCG

template  ACCGCCACCGATATAGCGAACAACGAAAGCGCCGCCGCGACGGTCGACGCGCCGGACAGT
query     TCATTCGACCTGGTGGTCGATACGCAATCGCCGCAGCAACCGGTAATCACCTTCATTACA

template  ACCGCGCCAGAAATGCCGAAAAACGTGGTAATTAGTGAGGATGGCGCCAGTATCAGCGGC
query     GATGATGCGCCGGGTATTCTCGGTAGCGTCGCGCATCTGGGGCTCACTAACGACAGCACG

template  ACCGCCGAACCGGGTAGCTCCATTACGATCACCACGCCGGACGGCACGCCGCTCGGCAGC
query     CCAACGATTAATGGTACAGGTGAACCGGGTTCCACAGTACACCTGTATCAGAATGGCGCC

template  GGCAAAGCAGATGGCGAAGGTCATTTTACCCTTCCCCTCGCCCCCGCACAGACCAACGGC
query     CGGATAGCAGATATTATCGTCGGTAATTCCGGCGTCTGGAGCTACGCTTACACCACGGCC

template  GAACAGGTTACCGTCACCGCCACCGACAGCGCCAACAACGTCAGCCCGCCAACTACAGCG
query     TCGCCACTGGCGGACGACACCTACACCTTTACCGTGACGGCCAGCGACAGTAACGGCAAC

template  CAAGCGCCCGATATCACCGCCCCGGATAAGCCCATTATCACCCAGGTGCTGGACGATGTT
query     ACCACGCCTTTTTCGACCGATTTTACGATTACCATTGATACCCAGGCCCCTGCCGCCCCC

template  GAAAGCTTCACCGGGCCGCTGGTTAACGGACAAACCACCAATGACAACCGCCCCACCCTT
query     GGCGTTATCGGCGTAGCTGACGGCGACGGAAATACGATTGATACCAATCAGATTACCCAG
```

```
template   AGCGGTACGGCGGAGGCTGGCGCGCGTGTCGAAATTTTTGATAACGGCGTTTCGCTGGGG
query      GAATCCCAGCCCCGGTTGAGCGGTAGCGGCACCGCAGGCGACACAATCATCCTTTACGAT

template   CTCGCCACGCTACAGCCCAACGGCGGCTGGACGTTTACGCCGTCGCAAAATTTAGGTGAA
query      AATGGCAATGCCATAGGTCAGGCGCTGGTCGGCACGGACGGGCGCTGGCAGTTTACGCCG

template   GGCGCGCATCGACTGACCGTAATCGCAACCGACGCTAAAGGCAATGCCAGTCCGGCGGGG
query      CCTGCCGCGCTGGGCGACGGCGACCACCTTCTGACCGCTCGCGCCAACGATCCGGCGGGG

template   AACGAAAGTCCGGAATCCATCAGCTTTACCCTACGCATCGATACCCAGGCGCCGGATGCG
query      AACGAAAGTCCCGAATCCATCAGCTTTACCCTACGCATCGATACCCAGGCGCCGGATGCG

template   CCGCAGATCGTGTCAGCCGCCATCACAGGCGGAGAAGGCGAGGTGCTACTGGCAAACGGC
query      CCGCAGATCGTGTCAGCCGCCATCACCGGCGGAGAAGGCGAGGTGCTACTGGCAAACGGC

template   AGTATTACCAATCAGCGTATGCCGACCCTCAGCGGCACCGGCGAACCCGGCGCCATCATC
query      AGTATTACCAATCAGCGTATGCCGACCCTCAGCGGCACCGGCGAACCCGGCACCATCATC

template   ACCCTGTACAATAACGGCGTAGAACTGGCTACCGTCCAGGTCAATCCACAGGGTAGCTGG
query      ACCCTGTACAATAACGGCGTAGAACTGGCTACCGTCCAGGTCAATCCACAGGGTAGCTGG

template   ACCTATCCGCTAACCCGTAATCTGAGCGAAGGGTTAAACATCCTGACGGCCACCGCCACG
query      ACCTATCCGCTAACCCGTAATCTGAGCGAAGGGTTAAACATCCTGACGGCCACCGCCACG

template   GATGCCGCAGGCAATAGTAGCCCGACCTCCGGCGTTTTCTCCGTTACCCTTGATACCCAG
query      GATGCCGCAGGCAATAGTAGCCCGACCTCCGGCGTTTTCTCCGTTACCCTTGATACCCAG

template   CCTCCAGCGCAGCCTGACGCGCCGCTAATCAGCGATAACGTCGCGCCGGTTATCGGCAAC
query      CCTCCAGCGCAGCCTGACGCGCCGCTAATCAGCGATAACGTCGCGCCGGTTATTGGCAAC

template   ATCGGCAATAATGGCGCAACGAACGATACCACGCCGACCTTCAGCGGCACGGGAGAGATC
query      ATCGGCAATAATGGCGCAACGAACGATACCACGCCGACCTTCAGCGGCACGGGAGAGATC

template   GGCAGCACGATAATTCTCTACAATAATGGCAGTGAAATTGGTCGCACAACGGTAGGCGAT
query      GGCAGCACGATAATTCTCTACAATAATGGCAGTGAAATTGGTCGCACAACGGTAGGCGAT

template   AACGGTAGCTGGAACTTTACGCCTGCGGCACTGACGCCAGAAACCTATACCATTACCGTC
query      AACGGTAGCTGGAACTTTACGCCTGCGGCACTGACGCCAGAAACCTATACCATTACCGTC

template   ACGGAAACCGATATAGCGGGCAATATCAGTCCACCTTCCGCCTCAGTCACTTTTACGCTA
query      ACGGAAACCGATAGAGCGGGCAATATCAGTCCACCTTCCGCCTCAGTCACTTTTACGCTA

template   GACACCACTGCGCCCGCCAATCCGGTTATCACTTTTGCCGAAGATAACGTCGGCGAAGTC
query      GACACCACTGCGCCCGCCAATCCGGTTATCACTTTTGCCGAAGATAACGTCGGCGAAGTC

template   CAGGATACTATTGTCAGCGGCGCAACCACTGACGACAATACACCGGTCATTCACGGCACT
query      CAGGATACTATTGTCAGCGGCGCAACCACTGACGACAATACACCGGTCATTCACGGCACT

template   GGCGACATCGGCAGCGTGATCACGCTCTATAATGGCAGTAGTGTCGTGGGCGTAGTCACC
query      GGCGACATCGGCAGCATTATTACGCTCTATAATGGCAGCAGCGTTTTAGGCGTAGTCACC

template   GTCGATGAGACCGGTACCTGGACGCTGCCGGTGACCAGCGCGTTGCCGGATGGCGTCTAC
query      GTCGATGAGACCGGCACCTGGACGCTGCCGGTGACCAGCGCGTTGCCGGATGGCGTCTAC

template   ACCCTGACCGCCATTGCCGCCGATGCCGCCGGAAACAGCAGCGGCGTATCGAACAGCTTT
query      ACCCTGACTGCCATTGCCGCCGATGCCGCCGGAAACAGCAGCGGCGTATCGAACAGCTTT

template   ACCTTCACCGTCGACACCGTTCCGTTGCAGCCGCCCGTCGTCAATGAGATCCTCGACGAT
query      ACCTTCACCGTCGACACCGTTCCGTTGCAGCCGCCCGTCGTCAATGAAATCCTTGACGAT

template   GTTGCACCAGTGACCGGGCCATTAACCGATGGCGCCTTTACTAACGATCGGACGCTGACT
query      GTTGCACCAGTGACCGGGCCATTAACCGATGGCGCCTTTACTAACGATCGGACGCTGACT

template   ATTAACGGCAGCGGCGAAAACGGCAGCACCGTCACGATTTACGACAATGGCGTGGCAATC
query      ATTAACGGCAGCGGCGAAAACGGCAGCACCGTCACGATTTACGACAATGGCGTGGCAATA

template   GGGACGGCGCTCGTTACCGACGGGGTCTGGACATTCAATACGTCCGAATTGTCAGAAGCC
query      GGGACGGCGCTCGTCACCGACGGGGTCTGGACATTCAATACGCCCGAATTATCAGAAGCC
```

```
template    AGCCATGCGCTAACCTTCAGCGCGACTGACGATGCTGGAAATACCACGGCGCAAACCCAA
query       AGCCATGCGCTAACCTTCAGCGCGACTGACGATGCTGGAAATACCACGGCGCAAACCCAG

template    CCGATCACCATTACCGTGGATATCACCGCCCCGCCCGCGCCAACGATCCAGACGGTGGCC
query       CCGATCACTATTACCGTGGATATCACCGCCCCGCCCGCGCCAACAGTCCAGACGGTGGAT

template    GATGATGGCACGCGCGTCGCCGGACTTGCCGATCCTTACGCTACCGTTGAAATTCATCAT
query       GACGATGGCACGCGCGTCGCCGGAATTGCCGACCCTTACGCTACCGTTGAAATTCATCAT

template    GCCGATGGCACCCTGGTCGGCAGCGCTGTCGCTAATGGCACCGGTGAATTTGTCGTCACG
query       ATCGATGGCACCCTGGTCGGCAGCGCTGTCGCTAATGGCACCGGTGAATTCGTCGTTACG

template    CTCAGTCCGGCGCAAACCGATGGCGGTACGCTGACGGCAATTGCTATCGATCGCGCGGGG
query       CTCAGTCCGGCGCAAACCGATGGCGGTACGCTGACGGCAATTGCTATCGATCGCGCGGGG

template    AATAACGGCCCGGCTACGAATTTTCCTGCTTCCGACAGCGGTCTGCCCGCCGTCCCGGCC
query       AATAACGGCCCGGCTACGAATTTTCCCGCTTCCGACAGCGGTCTGCCCGCCGTCCCGGCC

template    ATCACGGCGATTGAAGATGATGTCGGGAGCATACAGGGGAATATTGCAGCGGGCGGCGCC
query       ATCACGGCGATTGAAGATGATGTCGGGAGCGTACAGGGGAATATTGCGGCGGGCGGCGCC

template    ACGGACGACACCATGCCGACGCTGCGCGGCACCACGGATATCGGCTCTACCGTTGAAGTT
query       ACGGACGACACCACGCCGACGCTGCGCGGCACCACGGATATCGGCTCTACCGTTGAAGTT

template    TTCATTGATGGCGATTCGGCAGGCTTTGCCACCGTTGACGCCAGCGGGAACTGGATCTTT
query       TTCATTGATGGCGATTCGGCAGGCTTTGCCACCGTTGACGCCAGCGGGAACTGGATCTTT

template    GAGATCGCGACGCCATTAAGCGAAAGCACACATTACTTCACCGTCCAGGCAACCAATGCG
query       GAGATCGCGACGCCATTAAGCGAAAGCACACATTACTTCACCGTCCAGGCAACCAATGCG

template    AATGGCCCGGGCGGCCTGTCCGCACCGGTCGGGATCACTGTCGATCTTAGCGCGCCGGCA
query       AATGGCCCGGGCGGCCTGTCCGCACCGGTCGGGATCACTGTCGATCTTAGCGCGCCGGCG

template    CAACCGGTTATTACCAGCGCAACGGATGATGTCCCCGGCATGACCGGTACGCTGGATAAC
query       CAACCGGTTATTACCAGCGCAACGGATGATGTCCCCGGCATGACCGGTACGCTGGATAAC

template    GGCGCGCTCACCAATGATTCACGCCCGACGCTCAACGGAACGGGAGAAGCAGGCGCCACA
query       GGCGCGCTCACCAATGATTCACGCCCGACGCTCAACGGAACGGGAGAAGCTGGCGCCACG

template    ATCCGCATTCTGGATAACGGCGTAGAAATCGGTTCCGCTACGGTAGATCAAAGCGGCAAC
query       ATCCGCATTCTGGATAACGGCGTAGAAATCGGTTCCGCCACGGTAGATCAAAGCGGCAAC

template    TGGCGCTTCACCCCGAACACGCCGCTGGAGAGCAACGCACACATCTTTACCGCCGTGGCG
query       TGGCGCTTCACCCCGAACGCGCCGCTGGAGAGCAACGCGCACATCTTTACCGCCGTAGCG

template    ACCGATCCCGCCGGCAATAGCGGTCAGCTTTCGGATGGTTTTACGCTGAACATTGACGCG
query       ACCGATCCCGCCGGCAATAGCGGCCAGCCTTCGGACGGCTTTACGCTGAACATTGACGCG

template    CAGGCACCAGATGTGCCGGTTATCACCTCCGTGATTGACGATAACAATCAACCGACCGTC
query       CAGGCGCCAGATGTGCCGGTTATCACCTCCGTGATTGACGATAACAATCAACCGACCGTT

template    CCGGTGTTACCGGGGCAATCCACCGACGATCGGCAGCCAATACTGAACGGAACTGGCGAA
query       CCGGTGTTACCGGGGCAATCCACCGACGATCGGCAGCCAATACTGAACGGAACTGGCGAA

template    CCTGGCGCGACAATCACCATTTTTGATAACGGTACGCCGCTTGGCACGGCTCAGGTAGGC
query       CCTGGCGCGACAATCACCATTTTTGATAACGGTACGCCGCTTGGCACGGCTCAGGTAGGC

template    GAAAATGGTAGCTGGACCTTCCCGGTGCCCCGCAATTTGTCAGAGGGAAGCCATAATCTG
query       GAAAACGGTAGCTGGACCTTCCCGGTGCCCCGCAATTTGTCAGAGGGAAGCCATAATCTG

template    ACGGTTAGCGCTACCGATCCGGCGGGCAATACCAGCGCGGTCTCCGCGCCGTGGACGATC
query       ACGGTTAGCGCTACCGATCCGGCGGGCAATACCAGCGCGGTCTCCGCGCCGTGGACGATC

template    GTGGTCGATATTACGCCTCCGGCGATCCCGGTTCTCACCTCCGTTGTGGATGACCAGCCC
query       GTGGTCGATATTACGCCTCCGGCGATCCCGGTTCTCACCTCCGTCGTGGATGACCAGCCC

template    GGTATTACCGGCAACCTGGTTAGCGGGCAGCTAACGAACGATGCGACGCCCACCCTGAAC
query       GGAATTACCGGCAACCTGGTTAGCGGGCAGCTAACGAACGATGCGACGCCCACCCTGAAC

template    GGGCGCGGAGAGGCAGGCGCGACGATTAATGTCTATCTTGACGGTAATCCCGCGTCCATC
```

```
query       GGGCGCGGAGAGGCAGGCGCGACGATTAATGTCTATCTTGACGGTAATCCCGCGTCCATC

template    GGTACCACGACGGTGAATAGCGACGGCACGTGGAGTTTCACGCCGCAGACGCCGCTTGCA
query       GGTACCACGACGGTGAATAGCGACGGCACGTGGAGTTTCACGCCGCAGACGCCGCTTGCA

template    AACGGTAGCCACACGTTCACCCTTAGCGCCACCGATCCGGCGGGTAATAGCAGCGCGGTG
query       AACGGTAGCCACACGTTCACCCTTAGCGCCACCGATCCGGCGGGTAATAGCAGCGCGGTG

template    TCCAGCGGATTTGTGCTGACGATTGACACCACACCGCCCGCCGCGCCGGTTATCGCCAGC
query       TCCAGCGGATTTGTGCTGACGATTGACGCCACACCGCCCGCCGCGCCGGTTATCGCCAGC

template    GTGGCAGATAATACGGCGCCGGTAACGGGCATCGTCCCCAACGGCGGCTCGACGAACGAA
query       GTGGCAGACAATACGGCGCCGGTAACGGGCATCGTCCCCAACGGCGGCTCGACGAACGAA

template    ACCCGACCAACACTCTCGGGTACCGGTGAGGCGGGTACAACCATCTCGATTTATAATGGC
query       ACCCGACCAACACTCTCGGGTACCGGTGAGGCGGGTACAACCATCTCGATTTATAATGGC

template    AGCGCGCTGGTCGGCACGGCGCAAGTTCAGGCCAACGGTAGCTGGAGCTTTACACCGTCT
query       AGCGCGCTGGTCGGCACGGCGCAAGTTCAGGCCAACGGTAGCTGGAGCTTTACACCGTCT

template    ACCTCGCTGGGCGCGGGCGTCTGGAACCTGACGGCGACAGCAACCGATGCGGCAGGCAAT
query       ACCTCGCTGGGCGCGGGCGTCTGGAACCTGACGGCGACAGCAACCGATGCGGCAGGCAAT

template    ACCAGCGCCGCGTCCGAAATACGCTCGTTTACTATTGATACCACGGCTCCCGCCGCGCCT
query       ACCAGCGCCGCGTCCGAAATACGCTCGTTTACTATTGATACCACGGCTCCCGCCGCGCCT

template    GTTATTGATACGGTCTACGACGGTACGGGCCCCATTACCGGCAATCTGAGTTCAGGGCAG
query       GTTATTGATACGGTCTACGACGGTACGGGCCCCATTACCGGCAATCTGAGTTCAGGACAG

template    ATCACAGACGAGGCGCGCCCTGTCATTAGCGGCACCCGTGAAGCCAACACAACTATTCGT
query       ATCACAGACGAGGCGCGCCCTGTCATTAGCGGCACCCGTGAAACCAACACAACTATTCGT

template    CTCTACGATAACGGCACGCTGCTGGCTGAAATTCCCGCCGACAATAGCAGTAGCTGGCGC
query       CTCTACGATAACGGCACGCTGCTGGCTGAAATTCCCGCCGACAATAGCAGTAGCTGGCGC

template    TACACGCCCGACGCCTCGCTGGCGACGGGCAACCATGTAATTACCGTCATTGCCGTTGAT
query       TACACGCCCGACGCCTCGCTGGCGACGGGCAACCATGTAATTACCGTCATTGCCGTTGAT

template    GCCGCAGGCAACGCCAGCCCCGTTTCGGACAGCGTTAATTTCGTCGTCGATACCACGCCG
query       GCCGCAGGCAACGCCAGCCCCGTTTCGGACAGCGTTAATTTCGTCGTCGATACTACGCCG

template    CCGCTGACGCCGGTAATCACATCAGTCAGTGACGATCAGGCGCCAGGCCTCGGCACGATC
query       CCGCTGACGCCGGTAATCACATCAGTCAGTGACGATCAGGCGCCAGGCCTCGGCACGATC

template    GCGAACGGCCAAAATACCAACGATCCTACGCCAACCTTCAGCGGCACCGCAGAAGCCGGC
query       GCGAACGGCCAAAATACCAACGATCCTACGCCAACCTTCAGCGGCACCGCAGAAGCCGGC

template    GCCACGATCACGCTCTATGAAAATGGTACGGTCATTGGCACGACAACGGCTCAGCCTGAC
query       GCCACGATCACGCTCTATGAAAATGGTACGGTCATTGGCACGACAACGGCTCAGCCTGAC

template    GGCGCGTGGAGCGTCTCCACCTCAACGTTGGCAAGCGGAACGCACGTCATCACCGCCGTC
query       GGCGCGTGGAGCGTCTCCACCTCAACGCTGGCAAGCGGAACGCACGTCATCACCGCCGTC

template    GCCACCGATGCCGCAGGAAACAGCAGCCCGAACAGTACGGCTTTCACCCTGACGGTCGAT
query       GCCACCGATGCCGCAGGAAACAGCAGCCCGAACAGTACGGCTTTCACCCTGACGGTCGAT

template    ACCACCGCGCCGCAAACGCCAATCCTGACGTCCGTGGTGGATGACGTCGCGGGCGGGGTC
query       ACCACCGCGCCGCAAACGCCAATCCTGACGTCCGTGGTGGATGACGTCGCGGGTGGGGTC

template    ACAGGAAATCTCGCTAATGGTCAGATAACCAATGATAACCGCCCCACGCTGAACGGCACT
query       ACAGGAAATCTCGCTAATGGTCAGATAACCAATGATAACCGCCCCACGCTGAACGGCACT

template    GCCGAAGCGGGCAGCGTGGTCAGTATCTATGATGGCGACACTCTGCTTGGCGTCACCTCG
query       GCCGAAGCGGGCAGCGTGGTCAGTATCTATGATGGCGACACTCTGCTTGGCGTCACCTCG

template    GCTAACGCTAGCGGCGCATGGAGCTTCACGCCGACGACAGGGTTAAACGACGGCACGCGC
query       GCTAACGCGAGCGGCGCGTGGAGCTTCACGCCGACGACAGGGTTAAACGACGGCACGCGC

template    ACATTAACAGTGACCGCCACCGACCCAGCAGGCAACGTTAGCCCGGCCACCAGCGGTTTT
query       ACATTAACAGTGACCGCCACCGACCCGGCAGGCAACGTTAGCCCGGCCACCAGCGGTTTT
```

```
template   ACTATCGTGGTCGATACCCTTGCGCCAACGGTTCCGCTTATAACCAGCATCGTTGATGAT
query      ACTATCGTGGTCGATACCCTTGCGCCAACGGTTCCGCTTATAACCAGCATCGTTGATGAT

template   GTCCCGAACAATACCGGCGCCATTGGCAATGGACAATCGACCAATGACACACAGCCGACG
query      GTCCCGAACAACACCGGTGCCATTGGCAATGGACAATCGACCAACGACACACAGCCGACG

template   CTCAACGGTACCGCGGAAGCCAACAGCGCGGTAAGTATCTTCGATAATGGCGCGCTGGTC
query      CTCAACGGTACTGCGGAAGCCAACAGCGCGGTAAGCATCTTCGATAATGGCGCGCTGGTC

template   GCGACCGTGAACGCCAATGCCAGCGGCAACTGGAGCTGGACGCCAACCGCCTCGCTCGGC
query      GCGACCGTGAACGCCAATGCCAGCGGCAACTGGAGCTGGACGCCAACCGCCGCGCTCGGC

template   CAGGGAAGTCATGCCTATAGCGTTAGCGCCGCCGATGCGGCTGGCAACGTTAGCGCCGCT
query      CAGGGAAGTCACGCCTATAGCGTTAGCGCCGCCGATGCGGCTGGCAACGTTAGCGCCGCT

template   TCGCCATCGACAACGATTATCGTGGATACCATTGCGCCCGGCGCGCCCGGTAACCTAGTC
query      TCGCCATCGATAACGATTATCGTGGATACCATTGCGCCCGGCGCGCCCGGCAACCTGGTC

template   ATCAATGCTACCGGTAATCGGGTGACGGGCACCGCGGAAGCAGGCAGCACAGTGACGATT
query      ATCAATGCTACCGGTAATCGCGTGACGGGCACCGCGGAAGCAGGCAGTACAGTGACGATT

template   ACCTCTGAGACTGGTGTGGTACTGGGAACCGCCACCGCCGACGGTACGGGCAGCTTCACC
query      ACCTCTGAGACTGGTGTGGTACTGGGAACCGCCACCGCCGACGGTACAGGCAGCTTCACC

template   GCCACACTCACGCCCGCGCAGACCAATGGTCAGCCGCTACTGGCATTTGCCCAGGATAAA
query      GCCACACTCACGCCCGCGCAGACCAATGGTCAGCCGCTACTGGCATTTGCCCAGGATAAA

template   GCAGGCAACACTGGCATTGCCGCCGGATTTACCGCGCCCGATACGCGCGTGCCGGAAGCG
query      GCAGGCAACACAGGCATTGCCGCCGGATTTACCGCGCCCGATACGCGCGTGCCGGAAGCA

template   CCGATCATCACCAACGTAGTGGATGATGTGGGTATTTATACCGGCGCTATCGCCAACGGT
query      CCGATCATCACCAACGTAGTGGATGATGTGGGTATTTATACCGGCGCTATCGCCAACGGC

template   CAGGTCACTAATGACGCACAACCCACATTGAATGGTACCGCTCAGGCGGGCGCCACGGTG
query      CAGGTCACTAATGACGCACAACCCACATTGAATGGTACCGCTCAGGCGGGCGCCACGGTG

template   AGCATTTATAACAACGGGGCGCTGCTCGGCACCACCACGGCGAACGCCAGCGGGAACTGG
query      AGCATTTATAACAACGGGGCGCTGCTCGGCACCACCACGGCGAACGCCAGCGGAAACTGG

template   AGCTTTACCCCGACAGGCAATTTGACCGAAGGCAGCCACGCCTTCACCGCTACCGCAACT
query      AGCTTTACCCCGACAGGCAATTTGACCGAAGGCAGCCACGCCTTCACCGCCACCGCGACT

template   AACGCCAACGGTACAGGCAGCGTCTCCACCGCCGCGACGGTGATTGTCGATACGCTGGCG
query      AACGCCAACGGTACAGGCAGCGTCTCCACCGCCGCGACGGTGATTGTCGATACGCTGGCG

template   CCCGGTACGCCGTCAGGTACGCTCAGCGCCGATGGCGGTTCACTTTCCGGACAGGCTGAG
query      CCCGGTACGCCGTCAGGTACGCTCAGCGCCGATGGCGGTTCACTTTCCGGGCTGGCAGAG

template   GCAAACAGCACCGTAACCGTAACGCTGGCGGGGGGCGTGACGCTCACCACCACCGCCGGC
query      GCAAACAGCACCGTAACCGTCACGCTGACGGGGGGCGTGACGCTCACCACCACCGCTGGC

template   AGCAACGGCGCATGGTCTCTCACCTTGCCGACAAAACAAATTGAAGGTCAACTCATTAAC
query      AGCAACGGCGCATGGTCTCTCACCTTGCCGACAAAACAAATTGAAGGTCAACTCATTAAC

template   GTGACGGCCACTGACGCTGCGGGTAACGCCTCCGGCACGTTAGGCATTACCGCGCCGATT
query      GTGACGGCGACTGACGCTGCGGGTAACGCCTCTGGCACGTTAGGCATTACCGCGCCGGTT

template   CTGCCGCTGGCGGCAAGGGATAACATAACCAGTCTTGATCTGACCTCTACCGCCGTCACC
query      CTGCCGCTGGCGGCAAGGGATAACATAACCAGTCTTGATCTGACCTCTACCGCCGTCACC

template   AGCACGCAAAACTATTCGGATTACGGCCTGCTGCTGGTTGGCGCGCTTGGCAATGTCGCC
query      AGCACGCAAAACTATTCGGATTACGGCCTGCTGCTGGTTGGCGCGCTTGGCAATGTCGCC

template   TCGGTTTTGGGTAACGATACCGCTCAGGTTGAGTTCACCATTGCTGAAGGTGGTACGGGC
query      TCGGTTTTGGGTAACGATACCGCTCAGGTTGAGTTCACCATTGCTGAAGGTGGTACGGGC

template   GACGTCACCATCGATGCCGCCGCAACGGGAATCGTGCTTTCGCTGCTCAGCACTCAGGAG
query      GACGTCACCATCGATGCCGCCGCAACGGGAATCGTGCTTTCGCTGCTCAGTACTCAGGAG
```

```
template    ATAGTGGTACAGCGCTATGACACCAGCCTCGGCACCTGGACGACGATCGTCAACACCGCC
query       ATTGTGGTACAGCGCTACGACACCAGCCTCGGCGCCTGGACGACGATCGTCAACACCGCC

template    GTTGGCGACTTCGCGAATTTGCTTACCCTGACCGGGAGCGGCGTTACCCTGAACCTGAAC
query       CTTGGCGACTTCGCGAATTTGCTTACCCTGACCGGGAGCGGCGTTACCCTGAACCTGAGC

template    GGACTGGGCGAAGGCCAGTACCGGGTACTCACTTATAACACCAGTCTGCTCGCCACCGGG
query       GGCCTGGGCGAAGGCCAGTACCGGGTACTCACTTATAACACCAGTCTGCTCGCTACCGGG

template    TCATATACCAGCCTGGATGTCGATGTACACCAGACCAGCGCAGGTATTATTAGCGGGCCA
query       TCATATACCAGCCTGGATGTCGATGTACACCAGACCAGCGCAGGTATTATTAGCGGGCCA

template    ACCATCAGTACCGGCAACGTCATGGCTGATGATACCGCGCCGACGGGCACCACGGTCACC
query       ACCATCAGTACCGGCAACGTCATGGCTGATGATACCGCGCCGACGGGCACCACGGTCACC

template    GCCATCACCAACGCCAACGGCGTCAGTACGCCGGTCGGCGCGGGCGGCGTGGATATCCTG
query       GCCATCACCAACGCCAACGGCGTCAGTACGCCGGTCGGCGCGGGCGGCGTGGATATCCTG

template    GGGCAATACGGCACGCTGCACATTAATCAGGATGGCAGTTACACCTACACGCTGACTAAG
query       GGGCAATACGGCACGCTGCACATTAATCAGGATGGCAGTTACACCTACACGCTGACTAAG

template    CCCACGGCGGGATACGGACATAAAGAGAGCTTCACCTACACCATCACCCAGAATGGCGTC
query       CCCACGGCGGGATACGGACATAAAGAGAGCTTCACCTACACCATCACCCAGAATGGCGTC

template    GGTAGCAGCGCCGCGCAACTGGTTATTAATTTGGGTCCCGCGCCTGTACCGGGCAGCGTG
query       GGTAGCAGCGCCGCGCAACTGGTTATTAATTTGGGTCCCGCGCCTGTACCGGGCAGCGTG

template    ATAGCGACAGACAATAACGCCTCGCTGGTCTTTGATACTCACGTTAGCTACGTCAACAAC
query       ATAGCGACAGACAATAACGCCTCGCTGGTCTTTGATACTCACGTTAGCTACGTCAACAAC

template    GGCCCCTCGACACAAAGCGGCGTCACGGTATTAAGCGTCGGACTTGGTAATGTACTGAAC
query       GGCCCCTCGACACAAAGCGGCGTCACGGTATTAAGCGTCGGACTTGGTAATGTACTGAAC

template    GCGAATCTGCTTGATGATATGACTAATCCGATCATCTTTAACGTTGAAGAAGGCGCTACG
query       GCGAATCTGCTTGATGATATGACTAATCCGATCATCTTTAACGTTGAAGAAGGCGCTACG

template    CGAACCATGACGTTACAGGGAACCGTCGGCGGCGTCTCACTGGTTTCCACGTTCGATCTG
query       CGAACCATGACGTTACAGGGAACCGTCGGCGGCGTCTCACTGGTTTCCACGTTCGATCTG

template    TACGTTTATCGCTTCAACGATGCCATTCAACAATATGAGCAGTTCCGGGTGCAAAAGGGC
query       TACGTTTATCGCTTCAACGATGCCATTCAACAATATGAGCAGTTCCGGGTTCAAAAGGGC

template    TGGATTAACACCCTGCTGTTAGCCGGACAGTCCCAGCCGCTGACCCTGACGTTGCCTGGC
query       TGGATTAACACCCTGCTGTTAGCCGGACAGTCCCAGCCGCTGACCCTGACGTTGCCTGGC

template    GGCGAATACTTGTTCGTGCTGAATACCGCCAGCGGCATTAGCGTCCTCACTGGCTATACG
query       GGCGAATACTTGTTCGTGCTGAATACCGCCAGCGGCATTAGCGTCCTCACTGGCTATACG

template    TTGGCGATTTCCCAGGACCACACCTATGCCGTTGACAGTATCACCGCCAACACCACCGGC
query       TTGGCGATTTCCCAGGACCACACCTATGCCGTTGACAGTATCACCGCCAACACCACCGGC

template    AACGTACTGACCAATGATGTCGCCCCTACGGACGCCCTCCTCACTGAAGTAAACGGCGTG
query       AACGTACTGACCAATGATGTCGTCCCTACGGACGCCCTCCTCACTGAAGTAAACGGCGTG

template    GCGATTGCGGCGACCGGCACGACGGAGGTAAATGGGCTGTATGGCTCGCTCATCATTGAC
query       GCGATTGCGGCGACCGGCACAACGGAGGTAAACGGGCTGTATGGCTCGCTCATCATTGAC

template    GCAAGAGGCAACTATACCTACACGCTGAAGAACGGCGTCGGCGCCGACAGCATTAAAACG
query       GCAAGAGGCAACTATACCTACACGCTGAAGAACGGCGTCGGCGCCGACAGCATTAAAACG

template    CCGGACAGCTTTATCTATACGCTCAAAGCGCCAAACGGCGATACCGATACGGCCTCGCTC
query       CCGGACAGCTTTATCTATACGGTCAAAGCGCCAAACGGCGATACCGATACGGCCTCGCTC

template    AATATCACGCCAACCGCCAGGGCGCTGGATGCGATTAATGATGTCAGCGATACCCTTAGC
query       AATATCACGCCAACCGCCAGGGCGCTGGATGCGATTAATGATGTCAGCGATACCCTCAGC

template    GTCGCCACGCTTCAGGATACCGCTGCCTGGCTGGACTCCAGCGTCGGCAGCGCCAGTTGG
query       GTCGCCACGCTTCAGGATACCGCTGCCTGGCTGGACTCCAGCGTCGGCAGCGCCAGTTGG

template    GGGCTACTCGGCAAATCGGGCAGCGGGAGCGGCACCTTTGACGTTGCAACGGGCACCGTA
```

```
query     GGGCTACTCGGCAAATCGGGCAGCGGGAGCGGCACCTTTGACGTTGCAACGGGCACCGTA

template  CTTAAAGGCGCGTCACTGGTCTTTGATGTCTCCACGCTCATTACGCTGGGCAATCTGAAT
query     CTTAAAGGCGCGTCACTGGTTTTTGATGTCTCCACGCTCATTACGCTGGGCAATCTGAAT

template  ATTAGCTGGGCCATTCAGGAGAACGGAACCGTCATACGCAACGGAACCGTCCCGGTGGCG
query     ATTAGCTGGGCCATTCAGGAGAACGGGACCGTCATACGCAACGGAACCGTTCCGGTGGCG

template  AATATCACGCTGGGCAGCGCGACGGTGACCGTCAACCTGAGCGGCCTGGAGCTGGATGCC
query     AATATCACGCTGGGCAGCGCGACGGTGACCGTTAACCTGAGCGGCCTGGAGCTGGATGCC

template  GGAACGTACACGCTTAACTTTACCGGCACCAATACCCTGGCCGGGGCGGCGACAATCACG
query     GGAACGTACACGCTTAACTTTACCGGCACCAATACCCTGGCCGGGGCGGCGACGATCACG

template  CCACGCGTCATCGGCACCACCGTCGATCTGGATAATTTTGAAACGTCCGGAACGCATACC
query     CCACGCGTCATCGGCACCACCGTCGATCTGGATAATTTTGAAACGTCCGGAACGCATACC

template  GTTCTCGGCAATATTTTTGACGGCAGCGACGCGGCGGGGGCGATGGATCAGCTTAATACG
query     GTTCTCGGCAATATTTTTGACGGCAGCGACGCGGCGGGGGCGATGGATCAGCTTAATACG

template  GTGAATACTCGCCTGAGCATTAGCGGGTATAACGGCAGCGCCGCCACGCTGGACGCCGCG
query     GTGAATACCCGCCTGAGCATTAGCGGGTATAACGGCAGCGCCGCCACGCTGGACGCCGCG

template  GCGAATACCACCAGCGCCACGATTCAGGGACATTACGGCACATTGCAAATTAACCTCGAT
query     GCGAATACCACCAGCGCCACGATTCAGGGACATTACGGCACATTGCAAATTAACCTTGAT

template  GGCGCTTACACCTACACCCTGAATAATGGCGTCGCGATGTCGTCCATCACCAGTAAAGAG
query     GGCGCTTACACCTACACCCTGAATAATGGCGTCGCGATGTCGTCCATCACCAGTAAAGAG

template  GTCTTTACCTATCAACTGGATGACAAGATAGGTCATACGGATAGCGCCACATTGACCATT
query     GTCTTTACCTATCAACTGGATGACAAGATGGGTCATACGGATAGCGCCACATTGACCATT

template  GATATGGCGCCGCAAATCGTCAGTACCAACCAAAACGATGTTCTTATCGGCTCCGCCTAT
query     GATATGGCGCCACAAATCGTCAGTACCAACCAAAACGATGTTCTCATCGGCTCCGCCTAT

template  GGCGATACGCTGATTTATCACCTGTTAAACGGCGCGGACGCGACCGGCGGCAACGGCGCC
query     GGCGATACGCTGATTTACCACCTGTTAAACGGCGCGGACGCGACCGGCGGCAACGGCGTC

template  GATCGCTGGCAAAACTTCTCCACCGCGCAGGGCGACAAGATCGATATCCACGAACTGCTG
query     GATCGCTGGCAAAACTTCTCTACCGCGCAGGGCGACAAGATCGATATCCACGAACTGCTG

template  ACCGGCTGGGATCACCAGGCGGCGACGCTAGGTAACTTTGTTCAGGTTCATACCAGCGGC
query     ACCGGCTGGGATCACCAGGCGGCGACGCTGGGTAACTTTGTTCAGGTTCATACCAGCGGC

template  GCCAATACGGTGATATCCGTCGATCGCGACGGCGCCGGCAGCGCGTTTAAATCAACTGAC
query     GCCAATACGGTGATATCCGTCGATCGCGACGGCACCGGCAGCGCGTTTAAATCAACCGAC

template  CTTGTCACTTTGGAGAATGTGCAGCTCACGCTAAATGATCTGTTGCAGAACAACCACCTG
query     CTTGTCACTCTGGAGAATGTGCAGCTCACGCTAAATGATCTGTTGCAGAACAACCACCTG

template  ATAACCGGCGGTTGAT-AAAAAGCCCCGAACGCCACGACGTTCGGGGCAACAAGCGGTGA
query     ATAACCAGCGGTTGATAAAAAAGCCCCGAACGCCACGACGTTCGGGGCAACAAGCGGTGA

template  TATTTAAAAGGGATAAACTATGGGAAGAGTCGCGCCTGTCGCCATCGTACTGGCATTTGC
query     TATTTAAAAGGGATAAACTATGGGAAGAGTCGCGCCTGTCGCCATCGTACTGGCATTTGC

template  TTTGTTTCATCACCAGCCCCGGGGCGCAGAAGCGCTGCCAATGATTACATCCGAAGGATT
query     TTTGTTTCATCACCAGCCCCGGGGCGCAGAAGCGCCGCCAATGATTACATCCGAAGGATT

template  AGCAACGGACCAGATGCTTCCTTCGCTGGATGGCTCCGCCGCTGAGTTGCCGCTCAGCGC
query     AGCAACGGACCAGATGCTTCCTTCGCTGGATGGCTCCGCCGCTGAGTTGCCGCTCAGCGC

template  CGCCGCGCCGGGCAACCTGACGCTCAATGACGCGGTCAATCGCGCCGTTAACTGGCATCC
query     CGCCGCGCCGGGCAACCTGACGCTCAATGACGCGGTCAATCGCGCCGTTAACTGGCATCC

template  TTCTATTCGCGAAGCCGTCGGCAAACTGCTCGCACAGAATGAACAAATAGAGGTCGCCAA
query     TTCTATTCGCGAAGCCATCGGCAAACTGCTCGCACAGAATGAACAAATAGAGGTCGCCAA

template  ATCGAAATATTATCCGCAAGTCAGCGCTGGCGTGAACAATGGGTACAGCAATACCTACAC
query     ATCGAAATATTATCCGCAAGTCAGCGCTGGCGTGAACAATGGGTACAGCAATACCTACAC
```

```
template  CGATCACGGGTATAGCCCCTCGCTGGTGCTGTCGGTATCGCAAATGCTTTATGACTTCGG
query     CGATCACGGGTATAGCCCCTCGCTAGTGCTGTCGGTATCGCAAATGCTTTATGACTTCGG

template  CAAAGTGGCAAGCCAGGTTCGCGCCGAAACCGCAGGCGCAGCACAGCAACAGGCCAACGT
query     CAAAGTGGCAAGCCAGGTTCGCGCCGAAACCGCAGGCGCAGCGCAGCAACAGGCCAATGT

template  GTTGCTCAGTATTGATACCGTCGCGCACGAAACCGCCAACGCCATTGTACAAACGCAGAG
query     GTTGCTCAGTATTGATACCGTCGCGCACGAAACCGCCAACGCCATTGTACAAACGCAGAG

template  CTGGCAGCAAATGGTAGAAGCGGCGGAAGAACAGCTCGTCGCGCTGGACAGCATCGGTAA
query     CTGGCAGCAAATGGTAGAAGCGGCGGAAGAACAGCTCGTCGCGCTGGACAGCATCGGTAA

template  ACTTATCCGGCAGCGCAGCGATGAAGGCGCTACGTCGCTATCTGACGTGGTGCAAACCGA
query     ACTTATCCGGCAGCGCAGCGATGAAGGCGCTACGTCGCTCTCTGACGTGGTGCAAACCGA

template  AGCCAGAATCGAATCCGCCCGCTCGCAACTGGCGCAGTATCAGGCCAATCTCGACAGTGC
query     AGCCAGAATCGAATCCGCCCGCTCGCAACTGGCGCAGTATCAGGCCAATCTCGACAGTGC

template  GAAAGCCTCACTGATGAGCTGGCTGGGCTGGAATTCGCTTAACGGCATCAATAATGACTT
query     GAAAGCCTCACTGATGAGCTGGCTGGGCTGGAATTCGCTTAACGGCATCAATAATGACTT

template  CCCGGCGAAACTCGCTCGCAGCTGTGAGACAGCGACGCCCGAAGATCGGCTGGTGCCCGC
query     CCCGGCGAAACTTGCTCGCAGCTGTGAGACGGCGACTCCCGACGATCGACTGGTGCCCGC

template  GGTACTGGCCGCCTGGGCGCAGGCCAACGTTGCGCGGGCGAATCTGGACTATGCCAGTGC
query     GGTACTGGCCGCCTGGGCGCAGGCCAACGTTGCGCGGGCGAATCTGGACTATGCCAGCGC

template  GCAAATGACGCCGACGATTTCGCTCGAACCTTCTGTACAACATTATCTTAATGACAAATA
query     GCAAATGACGCCGACGATTTCGCTCGAACCTTCTGTACAACATTATCTTAATGATAAATA

template  TCCCAGTCATGAAGTGCTGGATAAAACCCAGTATTCCACCTGGGTGAAAGTTGAGATGCC
query     TCCCAGTCATGAAGTGCTGGATAAAACCCAGTATTCCACCTGGGTAAAAGTTGAGATGCC

template  GCTTTATCAGGGCGGAGGGCTGACTGCCCGACGCAACGCCGCCAGCCATGCGGTAGACGC
query     GCTTTATCAGGGCGGAGGGCTGACTGCCCGACGCAACGCCGCCAGCCATGCGGTAGACGC

template  GGCTCAGTCGACCATTCAGCGCACCCGGCTTGATGTCCGCCAGAAACTGATGGAGGCGCG
query     GGCTCAGTCGACCATTCAGCGCACCCGGCTTGATGTCCGCCAGAAACTGATGGAGGCGCG

template  CAGCCAGGCAATGAGTCTTGCCAGCGCGTTACAAATCCTTCGTCGGCAACAGCAGCTTAG
query     CAGCCAGGCAATGAGTCTTGCCAGCGCGTTACAAATCCTTCGTCGGCAACAGCAGCTTAG

template  CGAACGCACGCGCGAACTGTATCAGCAGCAATACCTTGACCTCGGTTCCCGCCCGTTGCT
query     CGAACGTACGCGCGAACTGTATCAGCAGCAATACCTTGACCTCGGTTCCCGCCCGCTGCT

template  CGACGTGCTTAATGCGGAGCAGGAGGTTTACCAGGCGCGTTTTGCCGAACTGCAAACGGA
query     CGACGTGCTTAATGCGGAGCAGGAAGTTTACCAGGCGCGTTTTGCCGAACTGCAAACGGA

template  AAGCCAGTTGCATCAGTTACAACTGAACTATCTGTATAACACCGGCGCGCTTCGTCAGGC
query     AAGCCAGTTGCATCAGTTGCAACTGAACTGTCTGTATAACACCGGCGCGCTTCGTCAGGC

template  GTTCGCGTTAAATCATCGCAGCATTCAATCCGTGGAGATCCAGCCATGACCCGCGCCGCC
query     GTTCGCGTTAAATCATCGCAGCATTCAATCCGTGGAGATCCAGCCATGACCCGCGCCGCC

template  CCCGATGTAGAAGAGGTACTCAGTGAGCGCGCGCTAAGCCAATGGGCGCAGGCCATCAGC
query     CCCGATGTAGAAGAGGTACTCAGTGAGCGCGCGCTAAGCCAATGGGCGCAGGCCATCAGC

template  CATGTCGCCGGTCATTATCGCGTTGCCTGTTCTCCCGGTTCAATTCAGGCCAACGCCCCG
query     TATGTCGCCGGTCATTATCGCGTTGCCTGTTCTCCCGGTTCAATTCAGGCCAACGCCCCG

template  TGGTTTAGGGGTAAAAGCAGAACGACCGCCTTAACGCAGCTCGCGCGGCAGGCGGGTTTA
query     TGGCTTAGGGGTAAAAGCAGAACGACCGCCTTAACGCAGCTCGCGCGGCAGGCGGGTTTA

template  TCCTTTCATGCGCCGGACATAGACAAAACGGCATTTAGTCAGTGGCGATTGCCGTTGGTT
query     TCCTTTCATGCGCCGGACATAGACAAAACGGCATTTAGTCAGTGGCGATTGCCGTTGGTT

template  GTCGAGCTCCGGGACGGGCAGTTATTGGTCATCGAACATGTTAATGGCGAAGATGCGGTA
query     GTCGAGCTCCGGGACGGGCAGTTATTGGTCATCGAACATGTTAATGGCGAAGAGGCGGTA
```

```
template   GACGTTTTTGTGATTGAAGAAGAGGGTCAGCGTAACCGCCTGACGCTCAGCGAGTTATTG
query      GACGTTTTTGTGATTGAAGAAGAGGGTCAGCGTAACCGCCTGACGCTCAGCGAGTTATTG

template   CCGGAGATCCTCTATGTTGCCGCGCTACGCCCGCTATCGGCGCTCAAGGATAGCCGCGTC
query      CCGGAGATCCTCTATGTTGCCGCGCTACGCCCGCTATCGGCGCTCAAGGATAGCCGCGTC

template   GACCGCTATATCTCCCGCTTTAAACCCGACTGGATGCGCGAGCTGGTTCTGCAGGATATT
query      GACCGCTATATCTCCCGCTTTAAACCCGACTGGATGCGCGAGCTGGTTCTGCAGGATATT

template   CGCCCTTATTTACCGGTAATGGTCGCCGCCTTTCTGATTAACGTGCTGTCTCTGGCCGGG
query      CGCCCTTATTTACCGGTAATGGTCGCCGCCTTTCTGATTAACGTGCTGTCGCTGGCCGGG

template   ATTGTGTTCTCTATGCAGGTTTACGATCGGGTGATCCCCGCTCAGTCATATCCTACCCTG
query      ATTGTGTTCTCTATGCAGGTTTACGATCGGGTGATCCCCGCTCAGTCATATCCTACCCTG

template   TACGTGCTCTCCTTCGGCGTGCTGGTAGCGGTGCTGTTCGGTTTTCTGCTGCGTGAAGCG
query      TACGTGCTCTCCTTCGGCGTGCTGGTAGCGGTGCTGTTCGGTTTTCTGCTGCGTGAAGCG

template   CGTACGCACATTATGGACGTGCTCGGTAAACGCGCCGATATGCGCATTTCCGATCGGGTA
query      CGTACGCACATTATGGACGTGCTCGGTAAACGCGCCGATATGCGCATTTCCGATCGGGTA

template   TTCGGTCATGCGTTAAGGCTGCGCAACAGCGCTATCCCCCGTTCCACCGGCAGTTTTATC
query      TTCGGTCACGCGTTAAGGCTGCGCAACAGCGCTATTCCCCGTTCCACCGGCAGTTTTATC

template   TCGCAGTTACGTGAACTGGAGCAGATCCGCGAGATGATTACCTCTTCAACGCTGGCGACC
query      TCGCAGTTACGCGAACTAGAGCAGATCCGCGAGATGATTACCTCTTCAACGCTGGCGACC

template   ATTGTCGATCTGCCTTTCTTTTTCCTGTTTATGATAGTCTTGGCGATTATTGCCCCACCG
query      ATTGTCGATCTGCCTTTCTTTTTCCTGTTTATGATAGTCCTGGCGATTATTGCCCCACCG

template   CTGGCATGGATAGCGCCCGTGGCGGCTCTGCTGATGATCCTGCCGGGCGTCGCACTGCAA
query      CTGGCATGGATAGCGCCCGTGGCGGCTCTGCTGATGATCCTGCCGGGCGTCGCACTGCAA

template   AAAAAACTGGCTGTCCTCGCCAACCAGGCCGCCCACGAAGCGACGCTACGCAATGCGGTC
query      AAAAAACTGGCTGTCCTCGCCAACCAGGCCGCCCACGAAGCGACGCTACGCAATGCAGTC

template   TTGGTCGAAAGCGTTCAGGGGCTGGAAGATATTAAACTGATGCAGGCGGAGAACCGCTTT
query      TTGGTCGAAAGCGTTCAGGGGCTGGAGGATATTAAACTGATGCAGGCGGAGAACCGCTTT

template   CTTCAGCAGTGGAATAGCTATATCCGTATTACCGGCGAGTCGGGTCTACGCACCCGCAAA
query      CTTCAGCAGTGGAATAGCTATATCCGTATTACCGGCGAGTCGGGTCTACGCACCCGCAAA

template   CTCACGCAAAGGCTTATCAGTTGGGGGATGTCGGTACAAAGTCTGGTGTATGCCGCAGTG
query      CTCACGCAAGGGCTTATCAGTTGGGGGATGTCGGTACAAAGTCTGGTGTATGCCGCAGTG

template   ATTATGTTCGGCGCGCCGATGGTCATTGAAGGCAGTATGACTACCGGCGCCGTGGTCGCC
query      ATTATGTTCGGCGCGCCGATGGTTATTGAAGGCAGTATGACTACCGGCGCCGTGGTCGCC

template   GCGTCGATGCTCGGCTCACGGATGATCGCGCCAATGGCTAACCTGTGCGGCGTGCTGGCG
query      GCGTCGATGCTCGGCTCGCGGATGATCGCGCCAATGGCTAACCTGTGCGGCGTGCTGGCG

template   CGCTGGCAGCAGGTGAAAGCGGCGAAGATGGGTCTCGACAACATTATGCAACTGCCCACT
query      CGCTGGCAGCAGGTGAAAGCGGCGAAGATGGGTCTCGACAACATTATGCAACTGCCCACC

template   GAGACACAGCATGACGATAGCCTGATACACCGCGACATCCTCCACGGGCATTACCTCTTT
query      GAGACACAGCATGACGATAGCCTGATACACCGCGACATCCTCCACGGGCATTACCTCTTT

template   GAAAACGCTCAGTTTCGTTACCATAACGACGATCAACGCATACCGCTGCGCCTCGTGCGT
query      GAAAACGCTCAGTTTCGTTACCATAACGACGATCAACGCATACCGTTGCGCCTCGTGCGT

template   CTGGAGATCATGCCAGGCGAGCGGATTGCGATACTGGGGCGTAACGGTGCGGGTAAATCC
query      CTGGAGATCATGCCAGGCGAGCGGATTGCGATACTGGGGCGTAACGGTGCGGGTAAATCC

template   ACACTTTTACAGGCGATGGCGGGCGGCCTGGAGATGATTCAGGGAGATGCCCGGCTTGAT
query      ACACTTTTACAGGCGATGGCGGGCGGCCTGGAGATGATTCAGGGAGATGCCCGGCTTGAT

template   AATCTCAGCTTGTCGCACATCGATATGGCGGATTTACGCCGCAACATCGGCTTTCTTAGC
query      AATCTCAGCTTGTCGCACATCGATATGGCGGATTTACGCCGCAACATCGGCTTTCTTAGC

template   CAGAACGCGCGGCTTTTCTTCGGCACCCTACGAGAGAACCTGACGCTCGGCGCGCCGCAC
```

```
query     CAGAACGCGCGGCTTTTCTTCGGCACCCTACGAGAGAACCTGACGCTCGGCGCGCCGCAC

template  GCCAACGATGAACAGATTTTTGACGCGCTCGAAGTCAGCGGCGGCGCCGTCTTCGTCAGG
query     GCCAACGATGAACAGATTTTTGACGCGCTCGAAGTCAGCGGCGGCGCCGTCTTCGTCAGG

template  CGGCTGGCAAAAGGGTTGGATCATCCCATTATGGAGGGTGGTAACGGCCTGTCCGGCGGG
query     CGGCTGGCAAAAGGGTTGGATCATCCCATTATGGAGGGTGGTAACGGCCTGTCCGGCGGG

template  CAGCGTCAGTCCCTGTTGCTGGCGAGAATGCTGCTGCGCTCCCCCAATATTGTGCTACTT
query     CAGCGTCAGTCCCTGTTGCTGGCGAGAATGCTGCTGCGCTCCCCCAATATTGTGCTACTT

template  GACGAACCCAGCGCCTCGCTGGACGAGCATACGGAACGAGAGTTTATTCAGCGGTTACAT
query     GACGAACCCAGCGCCTCGCTGGACGAGCATACGGAACGAGAGTTTATTCAGCGGTTACAT

template  CAGTGGCTTGGCAACCGTACCCTGGTCGTCGCAACGCACCGGGTACCAATACTGGAGCTG
query     CAGTGGCTTGGCAATCGAACCCTGGTCGTCGCGACGCACCGGGTACCAATACTGGAGCTG

template  GTTGAGCGTGTCGTTGTCCTGAAAGAAGGACAACTGGTGATGGACGCGCCAAAAGCGCAG
query     GTTGAGCGTGTCGTTGTCCTGAAAGAAGGACAACTGGTGATGGATGCGCCAAAAGCGCAG

template  GCGCTTAACGCGGATCGGATGCAAAGTCACCGTCGGGAGTAGAAAAATGAAAATCAATCA
query     GCGCTTAACGCGGATCGGATGCAAAGTCACCGTCGGGAGTGGAAAAATGAAAATCAATCA

template  GCATGATGCCGCGATGGACGATTTCGATATTCAGCGTGAACGGGCGTTTTCCGGCGCGGG
query     GCATGATGCCGCGATGGACGATCCCGATATTCAGCGTGAACGGGCGTTTTCCGGCGCGGG

template  TCGTATTGTTCTGATCTGCTCACTGTTATTTCTCATTCTCGGCATCTGGGCGTGGTTTGG
query     TCGTATTGTTCTGATCTGCTCACTGTTATTTCTCATTCTCGGCATCTGGGCGTGGTTTGG

template  CCGACTGGATGAGGTTTCCACCGGCAACGGGAAAGTAATCCCCAGTTCACGCGAACAGGT
query     CCGACTGGATGAGGTTTCCACCGGCAACGGGAAAGTGATCCCCAGTTCACGCGAACAGGT

template  TCTGCAGTCGCTGGATGGCGGCATTCTGGCGCAGTTGACGGTGCGGGAAGGCGACAGAGT
query     TCTGCAGTCGCTGGATGGCGGCATTCTGGCACAGTTGACGGTGCGGGAAGGCGACAGAGT

template  TCAGGCTAACCAGATTGTCGCCCAGCTTGATCCGACGCGTCTGGCGTCCAATGTTGGTGA
query     TCAGGCTAACCAGATTGTCGCCCGGCTTGATCCGACGCGTCTGGCGTCCAATGTTGGTGA

template  AAGTGCGGCAAAATATCGCGCTTCACTCGCCTCCAGCGCACGGTTAACCGCGGAAGTCAG
query     AAGTGCGGCAAAATATCGCGCTTCACTCGCCTCCAGCGCACGGTTAACCGCGGAAGTCAA

template  CGACTTACCTCTCGCCTTCCCCGCTGAGCTGAACGGCTGGCCGGATCTGATTGCCGCAGA
query     CGACTTACCTCTCGCCTTCCCCGCTGAGCTGAACGGCTGGCCGGATCTGATTGCCGCAGA

template  GACGCGTCTCTATAAAAGCCGCCGCGCGCAGCTGGCCGATACCGAAGCCGAGCTACGGGA
query     GACGCGTCTCTATAAAAGCCGCCGCGCGCAGCTGGCCGATACCGAAGCCGAGCTACGGGA

template  TGCGCTGGCGTCGGTTAATAAAGAGCTGACCATTACCCAGCGGCTGGAGAAAAGCGGCGC
query     TGCGCTGGCGTCGGTTAATAAAGAGCTGGCCATTACCCAGCGGCTGGAGAAAAGCGGCGC

template  GGCCAGTCATGTTGAAGTGCTACGCCTGCAACGACAAAAAAGCGATTTAGGCTTAAAAAT
query     GGCCAGTCATGTTGAAGTGCTGCGCCTGCAACGACAAAAAAGCGATTTAGGCTTAAAAAT

template  TACCGATCTGCGCTCACAATATTATGTGCAGGCACGCGAAGCGTTATCAAAAGCGAACGC
query     TACCGATCTGCGCTCACAATATTATGTGCAGGCACGCGAAGCGTTATCAAAAGCGAACGC

template  TGAGGTCGATATGCTCTCCGCCATTTTAAAAGGACGCGAGGATTCCGTCACCCGCCTTAC
query     TGAGGTCGATATGCTCTCCGCCATTTTAAAAGGACGCGAGGATTCCGTCACCCGCCTTAC

template  CATACGTTCGCCGGTACGCGGCATTGTTAAAAATATCCAGGTCACGACGATTGGCGGCGT
query     CGTGCGTTCACCGGTACGCGGCATTGTTAAAAATATCCAGGTCACGACGATTGGCGGCGT

template  GATCCCGCCTAACGGTGAGATGATGGAGATAGTGCCGGTAGACGATCGTCTGTTGATTGA
query     GATCCCGCCTAACGGCGAGATGATGGAGATAGTGCCGGTAGACGATCGTCTGCTGATTGA

template  AACCCGCCTTTCGCCGCGTGATATCGCCTTTATTCATCCCGGCCAACGCGCATTGGTTAA
query     AACCCGCCTTTCGCCGCGTGATATCGCCTTTATTCATCCCGGCCAACGCGCATTGGTTAA

template  AATTACTGCTTACGATTACGCCATTTACGGCGGGCTTGACGGCGTGGTGGAGACCATTTC
query     AATTACTGCTTACGATTACGCCATTTACGGCGGGCTTGACGGCGTGGTGGAGACCATTTC
```

```
template   ACCGGATACCATTCAGGATAAAGTGAAACCGGAAATTTTCTACTATCGCGTGTTTATCCG
query      ACCGGATACCATTCAGGATAAAGTGAAACCGGAAATTTTCTACTATCGCGTATTTATCCG

template   CACCCACCAGGACTATCTACAAAATAAATCAGGACGCCGTTTTTCGATTGTTCCAGGCAT
query      CACCCACCAGGACTATCTACAAAATAAATCAGGACGCCGTTTTTCGATTGTTCCAGGCAT

template   GATCGCCACGGTGGATATCAAAACCGGTGAAAAAACCATTGTCGACTATTTAATCAAACC
query      GATCGCCACGGTAGATATCAAAACCGGTGAAAAAACCATTGTCGACTATTTAATCAAACC

template   GTTTAATCGCGCGAAAGAAGCGCTGCGCGAGCGGTAA
query      GTTTAATCGCGCGAAAGAAGCGCTGCGCGAGCGGTAA
```

Input Files: *69M_cons_1.fasta*

Results as text | Results tsv | Hits in genome seqs | SPI sequences

Copyright DTU 2011 / All rights reserved
Center for Genomic Epidemiology, DTU, Kemitorvet, Building 204, 2800 Kgs. Lyngby, Denmark
Contact: Vibeke Dybdahl Hammer, Telephone: +45 3588 6420, E-mail: vdha@food.dtu.dk
Funded by: The Danish Council for Strategic Research
Last modified May 22, 2012 11:08:01 GMT
